# Synthesis of Unsymmetrical Difluoromethylene Bisphosphonates

**DOI:** 10.1021/acs.orglett.3c04211

**Published:** 2024-01-12

**Authors:** Jianyun Guo, Pascal Balić, Vladimir S. Borodkin, Dmitri V. Filippov, Jeroen D. C. Codée

**Affiliations:** † Leiden Institute of Chemistry, Leiden University, Einsteinweg 55, 2333 CC Leiden, Netherlands; ‡ Division of Molecular Cell and Developmental Biology, School of Life Sciences, University of Dundee, Dow Street, DD1 5EH Dundee, U.K.

## Abstract

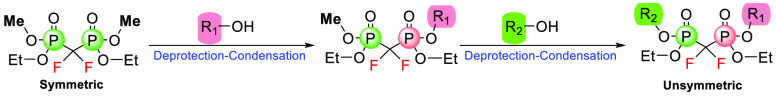

We demonstrate the use of the symmetrical diethyl(dimethyl)difluoromethylene bisphosphonate reagent for the synthesis of terminal and unsymmetrical difluoromethylene bisphosphonates, close analogues of biologically important molecules. The difference in reactivity of the methyl and ethyl groups in the symmetrical diethyl(dimthyl)difluoromethylene bisphosphonate is exploited in a stepwise demethylation–condensation sequence to functionalize either side of the reagent to allow the generation of a series of close bioisosteres of natural pyrophosphate molecules, including ADPr, CDP-glycerol and CDP-ribitol.

Naturally occurring pyrophosphates play important roles as building blocks in numerous vital biological processes.^1^ For example, nicotinamide adenine dinucleotide (NAD^+^, **1**) and flavin adenine dinucleotide (FAD, **2**) act as cofactors in a plethora of enzyme-catalyzed oxidations in living systems.^2^ NAD^+^ is also consumed to install a post-translational modification (PTM), referred to as adenosine diphosphate ribosylation, in which protein nucleophilic side chains are decorated with an adenosine diphosphate ribose (ADPr) moiety (as in **3**). These PTMs play diverse roles in cellular metabolism, signal transduction, and DNA repair.^3^ CDP-glycerol **4** and CDP-ribitol **5** are crucial building blocks for the biosynthesis of bacterial capsular polysaccharides and teichoic acids.^4^ CDP-ribitol **5** also serves as the donor substrate to generate the linkage unit that connects the matriglycan to dystroglycan.^5^ Nucleotide diphosphate (NDP) sugar donors, such as uridine diphosphate (UDP) galactose **6**, are Nature’s building blocks to construct oligo- and polysaccharides and glycoconjugates.^6^ The chemical modification of natural pyrophosphates is important to generate tools for structural studies,^7^ and in drug design and development to generate inhibitors,^8^ for example through the generation of analogues that are (more) stable toward enzymatic and chemical hydrolysis. Various entities have been probed over the years to serve as close analogues of the pyrophosphate moiety,^9^ including bisphosphonates (BPs),^10^ imidodiphosphates (PNPs),^11^ pyrophosphorothiolates,^12^ selenophosphates,^13^ boranophosphates,^14^ and phosphonoacetates^15^ (Figure 1B). Among these, the difluoromethylene bisphosphonate (P-CF_2_-P) analogues stand out,^16^ because they closely resemble the natural pyrophosphates, in terms of p*K*_a_-value, as well as bond angles and lengths.^17^ Furthermore, P-CF_2_-P linked analogues have a very similar polarity and size as their natural congeners.^18^ Several methods have been reported for synthesizing terminal difluoro phosphonates as pyrophosphate or triphosphate analogs, such as dicyclohexylcarbodiimide or other dehydrating-agent-mediated condensations, or the electrophilic phosphorylation of nucleosides by cyclotriphosphate and nucleophilic substitution of leaving groups using difluoromethylenebisphosphonate tetrabutylammonium salts.^9a,16d,19^ The latter approach has also been applied in a limited number of examples generating unsymmetrical difluoromethylene bisphosphonates.^8b,11c,20^ These reactions proceed with relatively poor yield, require the installation of leaving groups on the substrates, which may lead to side reactions, and generate charged intermediates which are challenging to purify.

**Figure 1:**
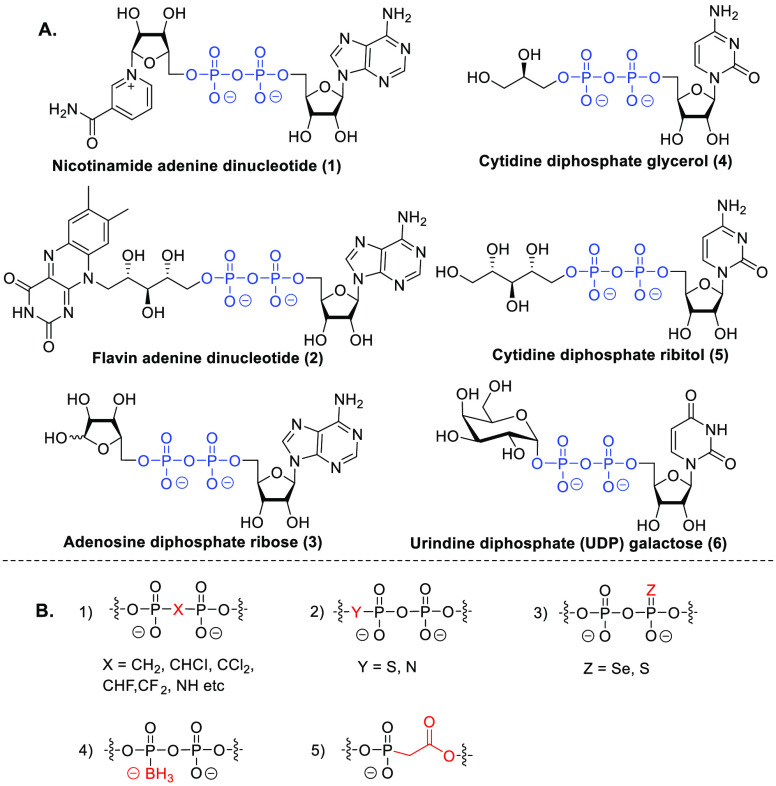
(A) Examples of natural unsymmetrical pyrophosphate molecules. (B) Examples are previously reported stabilized pyrophosphate mimics.

Here, we present a synthetic strategy to generate difluoromethylene bisphosphonate analogues of various natural products using a desymmetrization strategy of diethyl(dimethyl)difluoromethylene bisphosphonate **7**. We hypothesized that reagent **7** (Scheme 1) could be orthogonally deprotected to set the stage for one or two consecutive condensation reactions through P(V) condensation chemistry. We reasoned that cleavage of a single methyl ester in **7** could be selectively achieved because the bisfluoromethylene bisphosphonate monoanion is a better leaving group than the bisfluoromethylene bisphosphonate dianion, which would be generated after S_N_2 displacement of the second methyl ester. After condensation with the alcohol of choice, intermediate **9** is obtained. Hereafter, the remaining methyl ester may be cleaved selectively, leaving the larger ethyl esters unscathed and setting the stage for the second condensation on the opposite side of the reagent, providing unsymmetric difluoromethylene bisphosphonates **10**.

**Scheme 1:**
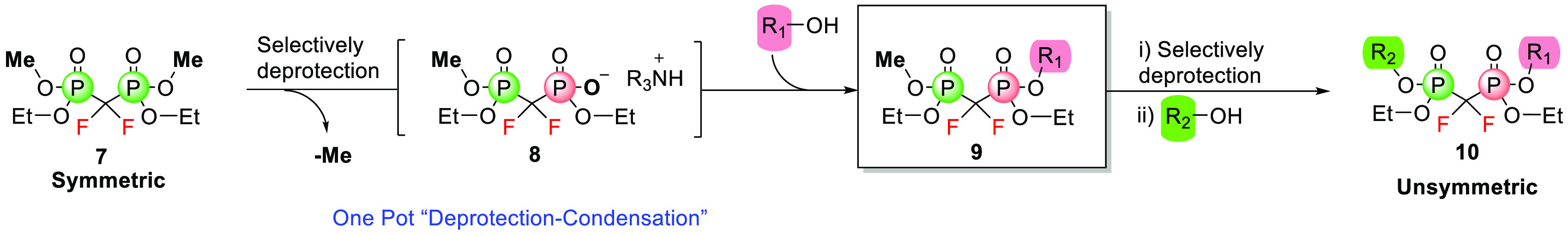
Strategy for the Synthesis of Unsymmetric Difluoromethylene Bisphosphonates

The required symmetrical diethyl(dimethyl)difluoromethylene bisphosphonate **7** was generated from commercially available tetraethyl methylenebisphosphonate **11**, which was fluorinated using *N*-fluorobenzenesulfonimide to obtain tetraethyl difluoromethylene bisphosphonate **12** in a 71% yield (Scheme 2).^21^ Two ethyl groups of tetraester **12** were selectively removed using refluxing morpholine to furnish the bismorpholinium salt **13** in 84% yield,^22^ which was converted to the corresponding acid **14** using Dowex ion-exchange resin. Esterification of acid **14** using trimethyl orthoformate provided the desired symmetrical difluoromethylene bisphosphonate **7** in 87% yield.^23^ The reagent could be readily generated on an 18 g scale.

**Scheme 2:**
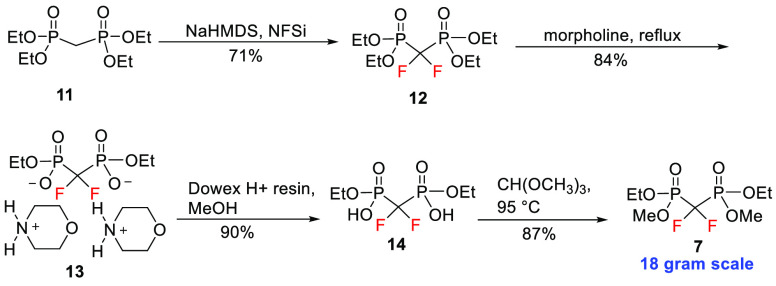
Synthesis of Symmetrical Diethyl(dimethyl)difluoromethylene Bisphosphonate **7**

With reagent **7** in hand, first the synthesis of the P-CF_2_-P linked ADPr analogue **21** was undertaken (Scheme 3A). ADP ribosylation is a PTM that plays a vital role in regulating various biological processes,^24^ and stable ADPr analogs are valuable tools to study the enzymes involved in ADP ribosylation as well as ADPr hydrolysis.^25^ To investigate the strategy proposed in Scheme 1, optimization of the selective mono-*O*-dealkylation conditions was required. While treatment of **7** with thiophenol (1 equiv) and triethylamine (1.5 equiv) provided a mixture of the desired monomethyl product and fully demethylated diethyl ester, the use of tetrabutylammonium benzoate (1 equiv) cleanly provided the desired mono tetrabutylammonium salt **15** (Scheme 3A). The use of ACN as a solvent provided more selectivity than DMF (Supporting Information). We have previously found^10^ 3-nitro-1,2,4-triazol-1-yl-tris(pyrrolidin-1-yl)phosphonium hexafluorophosphate (PyNTP, Wada’s reagent)^26^ to be an effective reagent for the condensation of methylene bisphosphonates and alcohols, and we therefore examined PyNTP as a condensation agent for our CF_2_-substrates. Using this reagent in the condensation of adenosine **16** with the tetrabutylammonium salt **15** of the difluoromethylene bisphosphonate reagent provided adenosine bisphosphonate **17** in 65% yield over 2 steps. It is worth noting that the tetrabutylammonium salt can be used directly for the condensation reaction in a one-pot fashion without the need for conversion to the corresponding acid and isolation and purification. Next, the remaining methyl ester in compound **17** was removed to allow for coupling with protected ribose **18**,^27^ resulting in the formation of protected ADPr analogue **19**. Purification by silica gel column chromatography of this product proved difficult, as the compound is highly polar with a polarity similar to that of the tetrabutylammonium hexafluorophosphate. Gratifyingly, size-exclusion chromatography (LH-20, MeOH/DCM 1:1) provided pure **19** in 77% yield. Deprotection of **19** was achieved by removal of the ethyl esters using thiophenol in MeCN/TEA,^10^ desilylation with hydrogen fluoride in pyridine, and finally, cleavage of the acetyl esters and adenine benzoyl groups using aqueous ammonia providing ADPr analogue **21**. However, partial cleavage of the ribose- and adenosine phosphonate linkages was observed, and the resulting side products could not be separated from the target compound **21**.^28^ We therefore purified partially deprotected ADPr after the desilylation step using preparative high-performance liquid chromatography (HPLC) to afford compound **20** in 48% yield. Subsequent deacylation then provided ADPr isostere **21** in 85% yield after isolation by gel filtration.

**Scheme 3:**
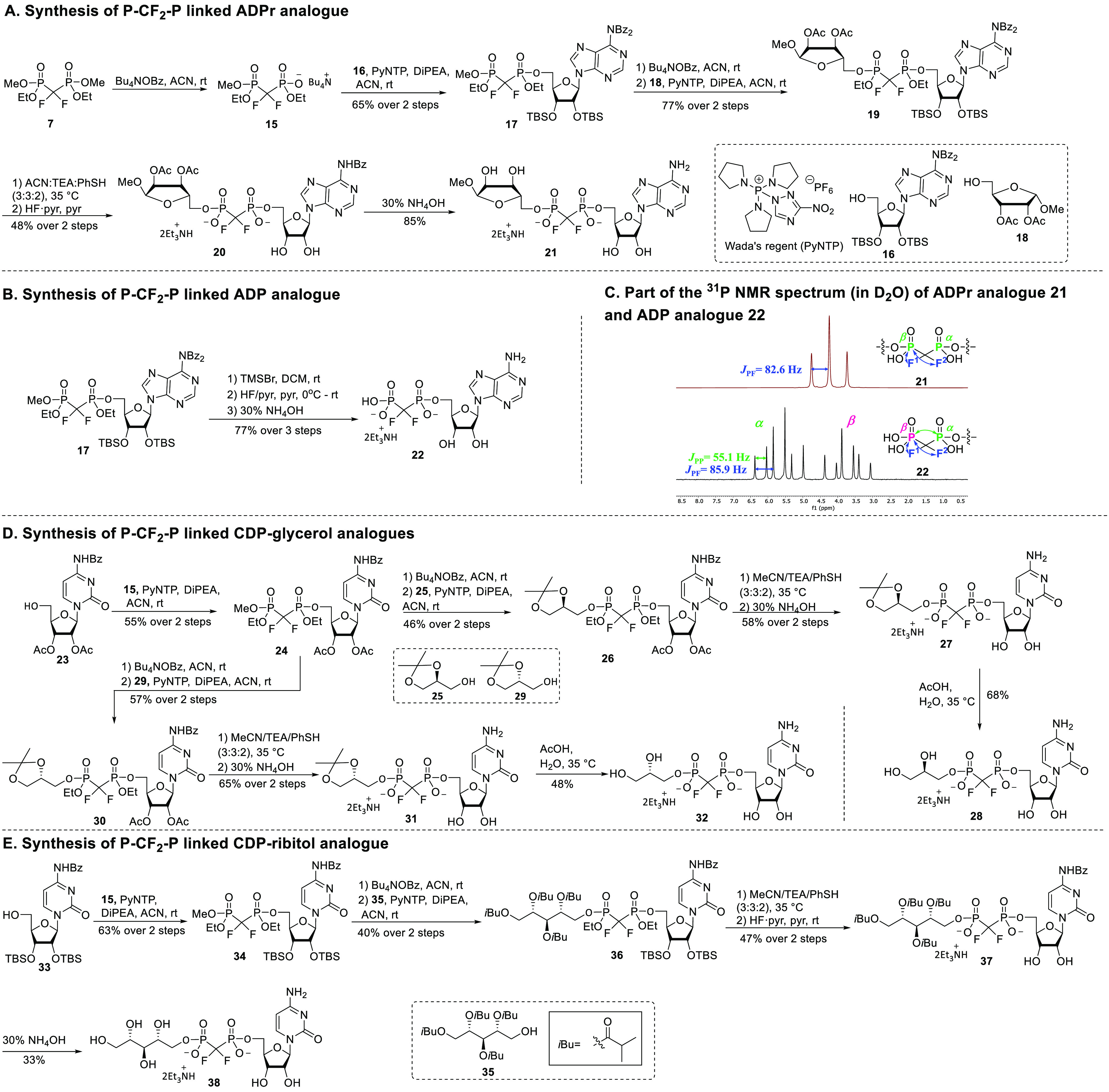
Synthesis of P-CF_2_-P Linked Analogues

Compound **17** could also be used as a precursor to generate the P-CF_2_-P analogue of adenosine diphosphate (ADP). To this end, we explored the deprotection of the methyl and ethyl phosphonate esters using bromotrimethylsilane (TMSBr),^29^ a commonly employed reagent for the deprotection of alkyl phosphate esters. Using 20 equiv of TMSBr, incomplete conversion was observed, even after an extended reaction time (7 days). However, by treating **17** with 20 equiv of TMSBr, 30 equiv of dry pyridine, and prolonging the reaction time to 2 weeks, full conversion was achieved. Finally, desilylation and deacylation afforded the ADP analogue **22** in 77% yield.

The ^31^P NMR of CF_2_-ADP **22** and CF_2_-ADPr **21** exhibited notable differences (See Scheme 3C). While the ^31^P NMR spectrum of **22** reveals a coupling between the two phosphorus atoms (*J*_PP_ = 55.1 Hz) and a coupling of the two fluorine atoms with both the α-phosphorus (α-P) and β-phosphorus (β-P) atoms (*J*_PF_ = 85.9 Hz), leading to two triplet of doublets, the ^31^P NMR of ADPr analogue **21** shows an apparent triplet. Apparently, the two phosphonates in **21** are magnetically very similar because of their similar substituents, which leads to the disappearance of the P–P coupling, generating a relatively simple apparent triplet in the spectrum.

Next, we evaluated the methodology in the synthesis of CDP-glycerol (CDP-Gro) and CDP-ribitol (CDP-Rib) analogues, which may be used to inhibit the enzymes that synthesize wall teichoic acids or aid in structural studies of these enzymes (Scheme 3D).^4^ CDP-glycerol is also used for the assembly of poly(glycosylglycerol phosphate) capsule polymers in Gram-negative pathogens, which represent crucial virulence factors of various pathogenic Gram-negative bacteria.^30^ Cytidine **23** was prepared via a three-step procedure (see Supporting Information), and devised such that only a single deprotection step is required to unmask the nucleoside. It was subjected to a condensation reaction with tetrabutyl ammonium phosphonate **15** to give compound **24** in 55% yield over two steps. After the demethylation of **24**, the resulting salt was condensed with commercially available (*S*)-solketal **25** under the agency of PyNTP to give the protected CDP-glycerol **26** in a 46% yield. The acyl and ethyl protecting groups in compound **26** were efficiently removed in a two-step process: first, removal of the ethyl groups was achieved with thiophenol in a mixture of MeCN/TEA, followed by deacylation using aqueous ammonia. Purification of the resulting crude product via preparative HPLC afforded intermediate **27** in 58% yield over 2 steps, of which the isopropylidene acetal was removed to provide the P-CF_2_-P analogue of CDP-Gro **28**. In an analogous fashion, we generated the diastereomeric counterpart starting from (*R*)-solketal **29**. It has been reported that some biosynthesis enzymes can actually accept both diastereoisomeric CDP-Gro substrates, incorporating either the *sn*-3 or *sn*-1 glycerolphosphates in poly(glycosylglycerol phosphate) polymers *in vitro*.^30b^ Both CF_2_ CDP-Gro enantiomers may therefore be relevant as potential inhibitors of these enzymes. The assembly of the *sn*-1 CF_2_ CDP-Gro **32** proceeded with similar yield as its *sn*-3 counterpart **28**.

Finally, we assembled CF_2_ CDP-Rib **38** as is shown in Scheme 3E. In line with the approach for the CDP-Gro analogue we combined cytidine building block **24** with ribitol **35** to enable a two-step deprotection (*i.e*., removal of the ethyl esters followed by cleavage of all remaining acyl groups), but this led to difficulties in purification and we therefore switched to the use of a cytidine building block carrying TBS-ethers. As noted in the synthesis of ADPr analogue **21**, the lipophilicity of these groups can be beneficial during HPLC purification. Hence, to assemble CDP ribitol analogue **38**, building block **33** was combined with **15** under the action of PyNTP to give **34** in 63% yield. Demethylation and coupling with **35** then gave fully protected CDP-ribitol. After removal of the ethyl groups, the TBS-groups were removed with HF-pyridine, followed by HPLC purification, to deliver pure **37** in 47% yield. Ammonia treatment of **37** then delivered the CDP ribitol analogue **38**.

In conclusion, we have described an efficient orthogonal deprotection–condensation strategy using diethyl(dimethyl) difluoromethylene bisphosphonate to synthesize unsymmetric difluoromethylene bisphosphonates, which can serve as close analogues to naturally occurring pyrophosphate-containing molecules. Symmetrical difluoromethylene bisphosphonate **7** allows for a stepwise functionalization sequence in which one methyl can be selectively removed to attach the first alcohol coupling partner, after which the second methyl can be removed to connect a second alcohol. We found Wada’s reagents to perform well in the construction of the difluorophosphonate ester linkages. The first synthesis of ADPr, CDP-glycerol, and CDP-ribitol difluoromethylene bisphosphonate analogues demonstrates the applicability of the devised methodology, making these molecules available for structural and biochemical studies.

## Data Availability

The data underlying this study are available in the published article and its Supporting Information.

## Supporting Information Available

Experimental details, ^1^H and ^13^C NMR spectra for all new compounds. (PDF)
