# Supplementary material for: Synthesis of Unsymmetrical Difluoromethylene Bisphosphonates
Source: Org Lett. 2024 Jan 12;26(3):739–44. doi: 10.1021/acs.orglett.3c04211 (PMC10825822; doi:10.1021/acs.orglett.3c04211)
Supplement: Supplementary file 1 — ol3c04211_si_001.pdf [file ol3c04211_si_001.pdf]

Supporting Information (SI)

Synthesis of Unsymmetrical Difluoromethylene Bisphosphonates

Jianyun Guo,[†] Pascal Balić,[†] Vladimir S. Borodkin,[‡] Dmitri V. Filippov,*[,†] and Jeroen D. C. Codée*[,†]

†Leiden Institute of Chemistry, Leiden University, Einsteinweg 55, 2333 CC Leiden, Netherlands

‡ Division of Molecular Cell and Developmental Biology, School of Life Sciences, University of Dundee, Dow Street, DD1 5EH Dundee, UK

*Email: jcodee@chem.leidenuniv.nl; filippov@lic.leidenuniv.nl

Contents

General Information **2**

Optimization Conditions for the Demethylation of Compound 7 .... **3**

Experimental Procedures **4-17**

NMR Spectroscopic Data **18-82**

Reference **83**

Experimental part

General Information

All chemicals were commercial grade and used as received and all moisture sensitive reactions were performed under an argon or nitrogen atmosphere, at ambient temperature (21°C), unless stated otherwise. For TLC analysis were used aluminium sheets (Merck , TLC silica gel 60 F_{254}), sprayed with a solution of H_2SO_4 (20%) in EtOH or with a solution of $(NH_4)_6Mo_7O_{24}$•$4H_2O$ (25 g/L) and $(NH_4)_4Ce(SO_4)_4$•$2H_2O$ (10g/L) in 10% aqueous H_2SO_4 or with a solution of $KMnO_4$ (2%) and K_2CO_3 (1%) in H_2O and then heated at ~150°C. For column chromatography was used 60Å silica gel (40-63 µm, SD Screening Devices). NMR spectra (^{1}H, ^{13}C, ^{19}F and ^{31}P) were recorded with a Bruker AV-400liq, Bruker AV-500 or a Bruker AV-600. Chemical shifts (δ) are given in ppm relative to tetramethylsilane as internal standard (^{1}H NMR in $CDCl_3$) or the residual signal of the deuterated solvent. Chemical shifts for ^{31}P spectra are indirectly referenced to H_3PO_4 (0.00 ppm) according to the IUPAC method. ^{31}P NMR spectra measured to monitor reactions were made by charging a NMR tube with an aliquot of the reaction mixture and fitting the tube with an acetone-d_6 capillary. Coupling constants (*J*) are given in Hz. All ^{13}C NMR and ^{31}P NMR spectra are proton decoupled. NMR peak assignments were made using COSY and HSQC experiments. LC/MS analysis was performed on a Surveyor HPLC system (Thermo Finnigan) equipped with a C_{18} column (Gemini, 4.6 mm x 50 mm, 5 µm particle size, Phenomenex), coupled to a LCQ Adventage Max (Thermo Finnigan) ion-trap spectrometer (ESI+). The applied buffers were H_2O, MeCN and 1% aqueous TFA. High resolution mass spectra were recorded by direct injection on a mass spectrometer (Thermo Finnigan LTQ Orbitrap) equipped with an electrospray ion source in positive ion mode (source voltage 3.5 kV, sheath gas flow 10, capillary temperature 275°C) with resolution R = 60000 at m/z 400 (mass range m/z= 150-4000) and dioctyl phthalate (m/z= 391.28428)as a lock mass. Size exclusion chromatography (SEC) was performed by constant elution (1 ml/min) with an aqueous NH_4OAc (0.15 M) and 10% MeCN buffer system over HW-40-S resin (16x 600 mm) from TOYOPEARL. Purification by preparative high pressure liquid chromatography (HPLC) was carried out on a Gilson-preparative-system equipped with a Phenomenex-Gemini-NX C18 column (5µm, 10x250 mm) using Buffer A (25 mM NH_4OAc in water) and Buffer B (MeCN). Optical rotation measurements ($[a]_D^{20}$) were performed on an Anton Paar Modular Circular Polarimeter MCP 100/150 with a concentration of 0.001 mg/mL.

Optimization conditions for the demethylation of compound 7

Figure SI-1. Optimization of the demethylation of compound **7**, monitored by ^{31}P NMR spectrometry. A: ^{31}P NMR spectrum (202 MHz, in $CDCl_3$) of compound **7**. B: ^{31}P NMR spectrum (202 MHz, in $CDCl_3$) of compound **14**. C: Overnight treatment of the reaction mixture with PhSH (1.0 eq) and TEA (1.5 eq) resulted in non-selective deprotection (monitored by ^{31}P NMR spectrometry (202 MHz, in $CDCl_3$)). D and E: Overnight treatment of the reaction mixture with Bu_4NOBz (1.0 eq) in either MeCN (D, monitored by ^{31}P NMR spectrometry (202 MHz, in $CDCl_3$)) or DMF (E, monitored by ^{31}P NMR spectrometry (122 MHz, in acetone-d_6 capillary)) at ambient temperature resulted in selective deprotection.

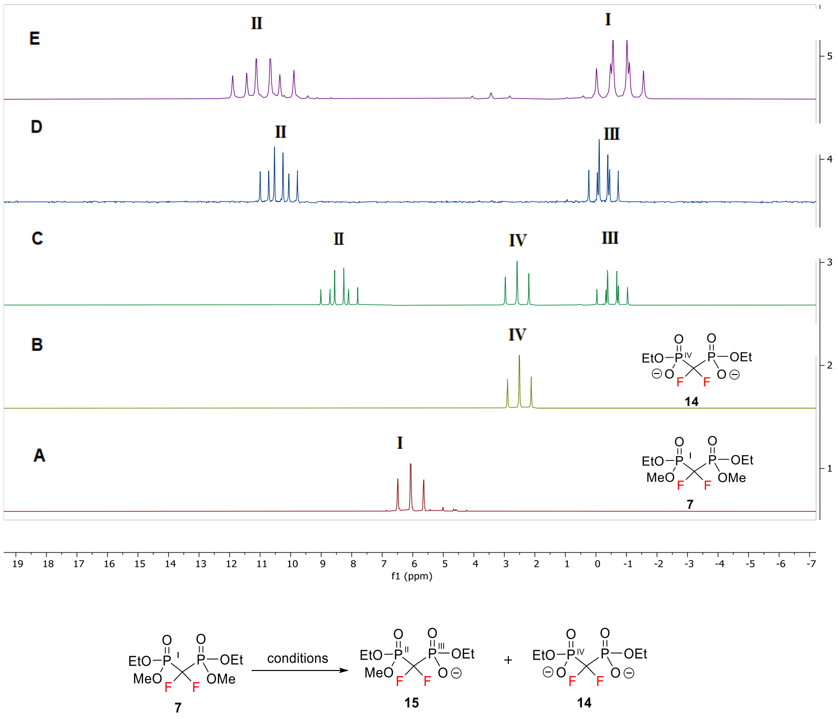

Experimental Procedures and Characterizations of Compounds

Tetraethyl difluoromethylenediphosphonate (12)

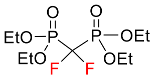

12

Tetraethyl methylenediphosphonate, (5 g, 17.3 mmol) was added in a flask under argon with a reflux condenser attached. NaHMDS (5.71 mL, 5.71 mmol of a 57.1 mL, 1.0 M solution (57.1 mmol) in THF) was added and the mixture was stirred for approximately 2 minutes. An anhydrous THF solution of N-fluorobenzenesulfonimide (NFSI) (5.19 ml, 5.19 mmol of a 51.9 mL, 1.0 M solution (51.9 mmol) in THF) was added and stirring was continued for approximately 2 minutes. Additional aliquots of NaHMDS (57.1 mL, 57.1 mmol) and NFSI (9.5 g in 51.9 mL THF, 51.9 mmol) were all added as described above. The reaction mixture was allowed to stir for 1h, then cooled to room temperature and the suspension was filtered. The residue was washed with hexane (20 mL) and the combined filtrate was concentrated in vacuo to give an oil. The oil was co-evaporated with hexane (30 mL) in vacuo to give an amber oil. The compound was purified with flash column chromatography, eluting with 10% acetone in dichloromethane (Rf= 0.25), to give **12** as a colorless oil (4.0 g, 12.3 mmol,71%). Spectroscopic data were in accord with those reported previously.[1] **^{1}H NMR** (400 MHz, $CDCl_3$): δ 4.40 – 4.22 (m, 8H), 1.37 (d, *J* = 8.0 Hz, 12H). ^{13}C NMR (101 MHz, D_2O): δ 120.7, 118.9, 118.0, 117.0, 116.1, 115.2, 114.2, 113.3, 65.5, 65.4, 65.4, 16.4. **^{31}P NMR** (162 MHz, $CDCl_3$): δ 1.64 (t, $J_{P\text{-}F}$ = 85.9 Hz). **^{19}F NMR** (471 MHz, $CDCl_3$): δ -121.85 (t, $J_{P\text{-}F}$ = 84.8 Hz). **HRMS (ESI) M/Z**: Calcd for $C_9H_{21}F_2O_6P_2$ 325.0775 $[M+Na]^+$; found 325.0778.

Morpholinium difluoromethylenebis(ethyl phosphonate) (13)

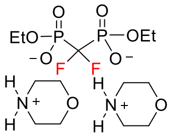

13

Morpholinium difluoromethylenebis(ethyl phosphonate) **12** (35 g, 79 mmol) was dissolved in in methanol (200 mL), Dowex 50W-X8 resin (30 g) was added and the resulting mixture stirred at rt for 4 h. The solution was filtered and the filtrate was concentrated under reduced pressure, giving the symmetrical P,P’-diethyl difluoromethylene bisphosphonic bismorpholinium salt **13** (19.2 g, 71.1 mmol, 90%) as a colorless oil: **^{1}H NMR** (400 MHz, $CDCl_3$): δ 4.11 – 4.06 (m, 4H), 3.94 – 3.87 (m, 8H), 3.17 – 3.09 (m, 8H), 1.28 (t, *J* = 7.1 Hz, 6H). **^{13}C NMR** (101 MHz, $CDCl_3$): δ 65.0, 62.9, 40.6, 16.4. **^{31}P NMR** (162 MHz, $CDCl_3$): δ 2.76 (t, $J_{P\text{-}F}$ = 76.1 Hz). **^{19}F NMR** (376 MHz, $CDCl_3$): δ -121.71 (t, $J_{P\text{-}F}$ = 75.2 Hz). **HRMS** (ESI) M/Z: Calcd for $C_5H_{13}F_2O_6P_2$ 269.0149 $[M+H]^+$; found 269.0155.

P,P'-diethyl difluoromethylene bisphosphonic acid (14)

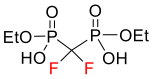

14

Morpholinium difluoromethylenebis(ethyl phosphonate) **13** (35 g, 79 mmol) was dissolved in in methanol (200 mL), Dowex 50W-X8 resin (30 g) was added and the resulting mixture stirred at rt for 4 h. The solution was filtered and the filtrate was concentrated under reduced pressure, giving the symmetrical P,P’-diethyl difluoromethylene bisphosphonic acid **14** (19.2 g, 71.1 mmol, 90%) as a colorless oil. **^{1}H NMR** (400 MHz, $CDCl_3$): δ 6.68 (s, 4H), 4.39 – 4.36 (m, 4H), 1.43 (t, *J* = 7.0 Hz, 6H). **^{13}C NMR** (101 MHz, $CDCl_3$): δ 66.3, 16.5. **^{31}P NMR** (162 MHz, $CDCl_3$): δ 1.90 (t, $J_{P\text{-}F}$ = 84.2 Hz). **^{19}F NMR** (376 MHz, $CDCl_3$): δ -126.41 (t, $J_{P\text{-}F}$ = 86.4 Hz). **HRMS** (ESI) M/Z: $[M+H]^+$: Calcd for $C_5H_{13}F_2O_6P_2$ 269.0149; found 269.0157.

Diethyl(dimethyl) difluoromethylene bisphosphonate (7)

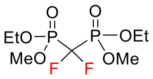

7

A solution of P,P'-diethyl difluoromethylenebisphosphonic acid **13** (19 g, 71 mmol) in trimethyl orthoformate (200 mL) was heated to reflux in an oil-bath for 16 h. The reaction mixture was cooled to room temperature and concentrated under vacuum. Purification by silica gel column chromatography (Rf= 0.3, 100% EtOAc) yielded compound **7** as a colorless oil (18.2 g, 61.8 mmol, 87%). *A mixture of diastereoisomers:* ^{1}**H NMR** (300 MHz, $CDCl_3$): δ 4.43 – 4.28 (m, 4H), 4.02 – 3.90 (m, 6H), 1.41 (t, J = 7.1 Hz, 6H). ^{13}**C NMR** (75 MHz, $CDCl_3$): δ 65.7, 65.72, 55.3, 16.5, 16.4, 16.4. ^{31}**P NMR** (121 MHz, $CDCl_3$): δ 4.63, 4.62 (ABX, $J_{P\text{-}F}$ = 85.9 Hz). ^{19}**F NMR** (376 MHz, $CDCl_3$): δ -121.31, 121.33 (ABX, $J_{P\text{-}F}$ = 86.4 Hz). **HRMS** (ESI) M/Z: $[M+H]^+$: Calcd for $C_7H_{17}F_2O_6P_2$ 297.0462; found 297.0468.

N,N-dibenzoyl-2,3-*bis*-O-tert-butyldimethylsilyladenosine (16)

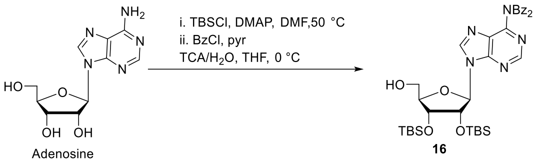

Adenosine (15 g, 56 mmol) was co-evaporated with anhydrous pyridine three times, dissolved in anhydrous dimethylformamide (84 mL), and heated to 50 °C in an oil-bath. Imidazole (19 g, 280 mmol) and tert-butyldimethylsilyl chloride (42.2 g, 280 mmol) were added consecutively and the mixture was stirred overnight. The reaction mixture was quenched by the addition of H_2O (41 mL), diluted with Et_2O (200 mL), and washed with H_2O (100 mL). The aqueous layer was extracted twice with Et_2O (2 x 100 mL), and the resulting organic layers were combined, washed with water and brine, dried over anhydrous MgSO4, filtered, and concentrated *in vacuo*. The crude product was co-evaporated with toluene three times, placed under a nitrogen atmosphere and dissolved in pyridine (110 ml), whereafter benzoyl chloride (13 ml g, 112 mmol) was added and the mixture stirred at room temperature for 5 hours. After analysis by TLC showed complete consumption of starting material, the reaction was quenched with sat. aq$NaHCO_3$ (150 mL) at 0 °C, diluted with EA (200 mL) and washed with water (150 mL) and brine (150 mL). The organic layer was dried over MgSO4, filtered, and concentrated *in vacuo*. The crude product was taken up in THF (600 mL), and aqueous TCA (318 g in 150 mL H_2O) was added at 0 °C. After stirring for 3 h at 0 °C, the reaction was quenched with sat. aq $NaHCO_3$ and diluted with dichloromethane (500 mL). The water layer was back-extracted twice with dichloromethane (200 ml). The combined organic layers were dried over anhydrous Na_2SO_4 and concentrated. The residue was purified by flash column chromatography (R_f = 0.3, PE/EA 3:1) to give **16** as a white solid (28.1 g, 43.1 mmol, 77%). ^{1}**H NMR** (300 MHz, $CDCl_3$): δ 8.65 (s, 1H), 8.11 (s, 1H), 7.88 – 7.84 (m, 4H), 7.52 – 7.46 (m, 2H), 7.38 – 7.32 (m, 4H), 5.85 (d, J = 12.0 Hz, 1H), 4.97 (dd, J = 6.0, 3.0 Hz, 1H), 4.32 (d, J = 3.0 Hz, 1H), 4.19 (t, J = 3.0 Hz, 1H), 3.95 (dd, J = 9.0, 3.0 Hz, 1H), 3.71 (t, J = 12.0 Hz, 1H), 0.95 (s, 9H), 0.75 (s, 9H), 0.13 (s, 3H), 0.11 (s, 3H), -0.15 (s, 3H), -0.71 (s, 3H). ^{13}**C NMR** (75 MHz, $CDCl_3$): δ 172.1, 153.0, 152.0, 151.6, 145.3, 134.0, 133.1, 130.2, 129.6, 128.9, 128.5, 91.3, 89.7, 74.1, 73.9, 63.0, 25.9, 25.8, 18.2, 17.9, -4.4, -4.4, -4.5, -5.7. **HRMS** (ESI) M/Z: $[M+H]^+$: Calcd for $C_{36}H_{50}N_5O_6Si_2$ 704.3294; found 704.3291. $[\alpha]^{20}_{D}$ = +20 ° (c = 0.001, $CHCl_3$)

Compound 17

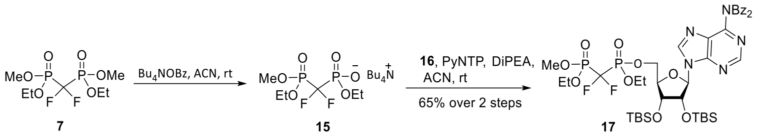

Compound **7** (820 mg, 2.77 mmol) was co-evaporated with toluene and dissolved in dry ACN (9.0 ml), whereupon tetrabutylammonium benzoate (1.0 g, 2.77 mmol) was added under nitrogen and the resulting mixture was cstirred overnight. Compound **16** (2.9 g, 4.16 mmol),dried prior to use by evaporation with toluene, DIPEA (4.7 ml, 27.7 mmol), and PyNTP (4.1 g, 8.31 mmol) were added consecutively. After 2 hours, the reaction was complete as indicated by TLC analysis. The reaction mixture was poured into DCM and washed with phosphate buffer (pH = 7). The water layer was extracted with DCM. The combined organics were dried over Na_2SO4, filtered and evaporated *in vacuo*. The crude product was purified by flash column chromatography over silica gel (R_f = 0.2, PE/EA 2:1) and then by size exclusion (LH-20) (DCM/MeOH 1:1) to obtain **17** as a yellow solid (178 mg, 1.8 mmol, 65%). *A mixture of diastereoisomers.* **^{1}H NMR** (300 MHz, $CDCl_3$): δ 8.65 (d, *J* = 1.4 Hz, 1H), 8.37 – 8.32 (m, 1H), 7.86 – 7.83 (m, 4H), 7.50 – 7.44 (m, 2H), 7.36 – 7.29 (m, 4H), 6.09 – 6.03 (m, 1H), 4.82 – 4.72 (m, 1H), 4.66 – 4.58 (m, 1H), 4.48 – 4.28 (m, 6H), 3.97 – 3.91 (m, 3H), 1.40 – 1.32 (m, 6H), 0.93 (s, 9H), 0.76 (s, 9H), 0.13 – 0.10 (m, 6H), -0.05 (s, 3H), -0.36 (s, 3H). **^{13}C NMR** (75 MHz, $CDCl_3$): δ 172.2, 153.0, 152.9, 152.2, 152.1, 151.9, 144.2, 144.0, 134.1, 132.9, 129.5, 128.7, 88.8, 88.8, 88.6, 88.5, 83.5, 75.0, 74.8, 74.7, 72.0, 71.9, 66.9, 66.7, 66.3, 66.1, 65.9, 55.4, 25.8, 25.6, 18.0, 17.8, 16.4, -4.4, -4.4, -4.6, -4.8, -5.1, -5.2. **^{31}P NMR** (202 MHz, $CDCl_3$): δ 5.35 - 4.36 (m). **^{19}F NMR** (471 MHz, $CDCl_3$): δ -113.02 – -122.85 (m). **HRMS** (ESI) M/Z: $[M+H]^+$: Calcd for $C_{42}H_{62}F_2N_5O_{11}P_2Si_2$ 968.3422; found 968.3430.

Fully protected CF_2-ADPr analogue (19)

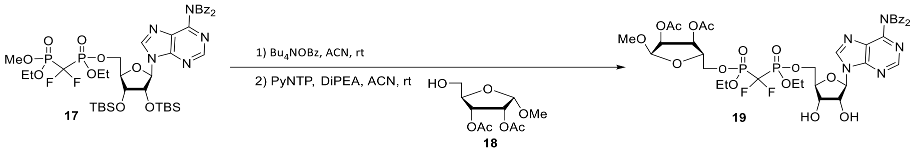

Compound **16** (200 mg, 0.21 mmol) was co-evaporated with toluene and dissolved in dry ACN (0.7 ml), whereafter tetrabutylammonium benzoate (74 mg, 0.21 mmol) was added under nitrogen and stirred overnight. Compound **18** (77 mg, 0.31 mmol), dried by evaporation with toluene prior to use, DIPEA (0.35 ml, 2.06 mmol), and PyNTP (308 mg g, 0.62 mmol) were added successively. After 2 hours, the reaction was complete as indicated by TLC analysis. The reaction mixture was poured into DCM and washed with phosphate buffer (pH = 7). The water layer was extracted with DCM. The combined organics were dried over Na_2SO4, filtered and evaporated *in vacuo*. The crude product was purified by flash column chromatography over silica gel (R_f = 0.3, PE/EA 1:1) and then by size exclusion (LH-20) (DCM/MeOH 1:1) to obtain **19** (188 mg, 0.16 mmol, 77%) as a yellow solid. *A mixture of diastereoisomers.* **^{1}H NMR** (400 MHz, $CDCl_3$): δ 8.85 – 8.60 (m, 1H), 8.42 – 8.26 (m, 1H), 8.02 (dd, *J* = 7.2, 1.6 Hz, 1H), 7.87 – 7.78 (m, 3H), 7.33 (t, *J* = 7.8 Hz, 5H), 6.08 (ddd, *J* = 15.5, 5.6, 3.3 Hz, 1H), 5.31 – 5.17 (m, 1H), 5.05 – 4.89 (m, 1H), 4.83 – 4.69 (m, 1H), 4.67 – 4.56 (m, 1H), 4.55 – 4.43 (m, 3H), 4.43 – 4.18 (m, 7H), 3.43 – 3.38 (m, 3H), 2.13 – 2.07 (m, 6H), 1.43 – 1.30 (m, 6H), 0.95 – 0.88 (m, 9H), 0.79 – 0.72 (m, 9H), 0.14 – 0.07 (m, 6H),

-0.01 – -0.09 (m, 3H), -0.26 – -0.31 (m, 3H), -0.36 – -0.40 (m, 3H). **^{13}C NMR** (101 MHz, $CDCl_3$): δ 172.2, 170.6, 170.5, 169.9, 169.8, 164.6, 153.0, 152.9, 152.8, 152.26, 152.2, 151.9, 151.7, 149.6, 144.3, 144.0, 134.1, 133.9, 132.9, 132.8, 129.5, 128.9, 128.9, 128.7, 128.3, 128.2, 127.9, 127.9, 101.8, 101.8, 101.7, 88.7, 88.7, 88.6, 88.5, 83.7, 83.6, 83.6, 83.3, 80.36, 76.8, 75.2, 75.1, 75.0, 74.9, 72.0, 71.8, 71.0, 70.7, 70.9, 70.6, 69.9, 69.7, 69.6, 69.6, 67.8, 67.6, 67.1, 67.1, 67.0, 66.9, 66.5, 66.5, 66.3, 66.3, 66.2, 66.1, 66.1, 66.0, 55.7, 55.7, 25.8, 25.7, 25.7, 20.9, 20.6, 18.1, 17.8, 17.8, 16.4, -4.4, -4.4, -4.6, -4.7, -4.7, -5.0, -5.1. **^{31}P NMR** (162 MHz, $CDCl_3$): δ 5.24 – 2.44 (m). **^{19}F NMR** (471 MHz, $CDCl_3$): δ -120.51 – -122.67 (m). **HRMS** (ESI) M/Z: $[M+H]^+$: Calcd for $C_{51}H_{73}F_2N_5O_{17}P_2Si_2$ 1184.4056; found 1184.4019.

Compound 20

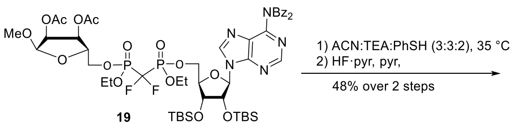

Full protected ADPr analogue **19** (125 mg, 0.11 mmol) was dissolved in MeCN: TEA: PhSH (3:3:2, 1 ml) and stirred for 6 hours at 35 °C. Reaction progression was monitored by ^{31}P NMR and LC-MS, which indicated complete consumption of the starting material. The solution was concentrated in vacuo. The residue was dissolved in pyridine and cooled to 0 °C, after which HF/pyridine (70%, 86 µL 3.3 mmol) was added and stirred overnight at rt. The resulting solution was quenched with sat. aq $NaHCO_3$ and concentrated. The resulting mixture was diluted with water then acidified to pH 6-7 using acetic acid. The mixture was washed with DCM (3x) to remove excess thiophenol and pyridine. The water layer was concentrated in vacuo then purified by HPLC to give compound **20** as a white solid (40.5 mg, 40 µmol, 48% over 2 steps). **^{1}H NMR** (400 MHz, D_2O): δ 8.79 (s, 1H), 8.72 (s, 1H), 7.98 – 7.96 (m, 2H), 7.69 – 7.64 (m, 1H), 7.57 – 7.53 (m, 2H), 6.25 (d, *J* = 5.5 Hz, 1H), 5.24 (dd, *J* = 6.7, 2.4 Hz, 1H), 5.14 (d, *J* = 4.6 Hz, 1H), 5.02 (dd, *J* = 6.7, 4.6 Hz, 1H), 4.55 – 4.53 (m, 1H), 4.38 – 4.36 (m, 1H), 4.33 – 4.28 (m, 3H), 4.16 – 4.14 (m, 2H), 3.34 (s, 3H), 2.02 (s, 3H), 1.99 (s, 3H). **^{13}C NMR** (101 MHz, D_2O): δ 154.3, 151.1, 148.9, 118.55, 103.2, 86.9, 84.1, 83.4, 74.3, 70.8, 70.3, 69.6, 66.0, 65.4, 55.4. **^{31}P NMR** (162 MHz, D_2O): δ 3.52 (ABX, J_{P-F} = 82.6 Hz). **^{19}F NMR** (471 MHz, D_2O): δ -118.84 (t, J_{P-F} = 80.1 Hz,). **HRMS** (ESI) M/Z: $[M+H]^+$: Calcd for $C_{28}H_{34}F_2N_5O_{16}P_2$ 796.1438; found 796.1437.

CF_2-ADPr analogue (21)

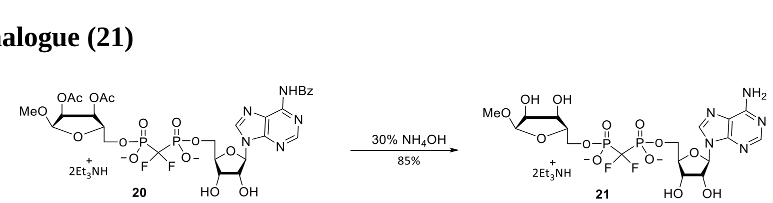

Compound **20** (40.5 mg, 40 µmol) was dissolved in aqueous ammonia (30 wt%, 5 mL) and stirred overnight. The excess ammonia was removed by stirring under vacuum. The crude product was purified by size-exclusion chromatography (HW40-column, 0.15 M aqueous NH_4OAc in water) followed by lyophilization to yield CF_2-ADPr analogue **21** (26.2 mg, 32 µmol, 85%) as a white powder. **^{1}H NMR** (400 MHz, D_2O): δ 8.42 (s, 1H), 8.09 (s, 1H), 6.04 (d, *J* = 5.7 Hz, 1H), 4.88 – 4.85 (m, 1H), 4.69 (t, *J* = 5.5 Hz, 1H), 4.48 (dd, *J* = 5.1, 3.6 Hz, 1H), 4.33 – 4.31 (m, 1H), 4.26 – 4.24 (m, 2H), 4.12 – 4.09 (m, 3H), 4.06 – 4.03 (m, 2H), 3.34 (s, 3H). **^{13}C NMR** (101 MHz, D_2O): δ 160.2, 155.3, 152.6, 148.8, 139.6, 120.9, 119.2, 118.36, 117.5, 116.5, 103.1, 86.7, 83.9, 83.8,

83.8, 83.4, 83.3, 83.3, 74.1, 70.7, 70.2, 69.6, 65.9, 65.4, 55.3. **^{31}P NMR** (162 MHz, D_2O): δ 4.24 (t, $J_{P\text{-}F}$ = 82.6 Hz,). **^{19}F NMR** (471 MHz, D_2O): δ -118.83 (t, $J_{P\text{-}F}$ = 84.8 Hz,). **HRMS** (ESI) M/Z: $[M+H]^+$: Calcd for $C_{17}H_{26}F_2N_5O_{13}P_2$ 608.0964; found 608.0967.

CF_2-ADP analogue (22)

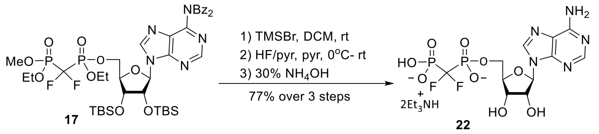

Compound **17** (200 mg, 0.21 mmol) was co-evaporated with toluene and dissolved in dry DCM (10 ml), whereupon pyridine (169 µL, 2.1 mmol) and trimethylsilyl bromide (0.45 ml, 4.2 mmol) were added under nitrogen and the reaction mixture was stirred for 2 weeks. After analysis by LC-MS showed complete consumption of starting material, pyridine (338 µL, 4.2 mmol) was added. The reaction was concentrated *in vacuo* to give a yellow colored oil. The oil was dissolved in pyridine (10 ml),cooled to °C and HF/pyridine (70%, 109 µL, 4.2 mmol) was added. The reaction mixture was warmed to rt and stirred for 24 h until LC-MS showed complete consumption of starting material. The reaction was diluted with water (10 ml), quenched with sat. aq $NaHCO_3$ (2ml) and washed with DCM (3x 10ml). Ammonia solution (30%) (5 ml) was added and the reaction was stirred overnight. The excess ammonia was removed by stirring under vacuum. The crude product was purified by size-exclusion chromatography (HW40-column, 0.15 M aqueous NH4OAc in water) followed by lyophilization to yield ADP analogue **22** (77.0 mg, 0.16 mmol, 77%) as a white powder. Spectroscopic data were in accord with those reported previously.[2] **^{1}H NMR** (400 MHz, D_2O): δ 8.48 (s, 1H), 8.16 (s, 1H), 6.07 (d, *J* = 4.0 Hz, 1H), 4.71 – 4.68 (m, 1H), 4.53 – 4.51 (m, 1H), 4.36 – 4.33 (m, 1H), 4.31 – 4.29 (m, 2H). **^{13}C NMR** (101 MHz, D_2O): δ 155.2, 152.4, 148.8, 139.9, 118.4, 86.8, 83.9, 83.8, 74.3, 70.1, 65.3, 65.2. **^{31}P NMR** (162 MHz, D_2O): δ 6.04 (dt, $J_{P\text{-}F}$ = 80.1 Hz, $J_{P\text{-}P}$ = 55.1 Hz, α-P), 3.89 (dt, $J_{P\text{-}F}$ = 77.8 Hz, $J_{P\text{-}P}$ = 53.5 Hz, *β*-P). **^{19}F NMR** (471 MHz, D_2O): δ -118.88 (t, $J_{P\text{-}F}$ = 84.8 Hz,). **HRMS** (ESI) M/Z: $[M+H]^+$: Calcd for $C_{11}H_{16}F_2N_5O_9P_2$ 462.0385; found 462.0396.

***N*^{4}-benzoyl-2', 3'-di-*O*-acetyl cytidine (23)**

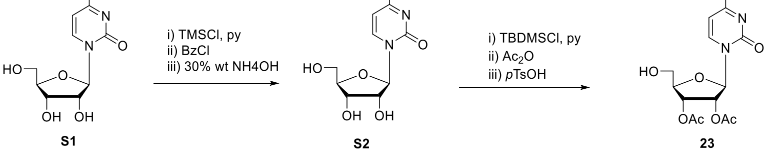

Cytidine (5.0 g, 20.5 mmol) was co-evaporated with pyridine three times and suspended in anhydrous pyridine (102 ml). Trimethylsilyl chloride (23.4 ml, 184 mmol) was slowly added and the mixture stirred for 2h, then BzCl (11.9 ml, 102.5 mmol) was added dropwise and the mixture was stirred for an additional 2h. The reaction mixture was cooled to 0 °C and quenched with water (20 ml) and warmed to room temperature. Aqueous NH_4OH (30 wt%,40 ml) was added and the reaction mixture was concentrated in vacuo to give a white solid. The solid was partitioned between H_2O (280 ml) and EtOAc (80 ml), the organic layer was discarded, and the aqueous layer cooled to 0 °C. The formed white crystals were collected by filtration, rinsed with ice-cold Et_2O anddried under high vacuum at 60 °C to give 4-*N*-benzoyl-*β*-D-cytidine **S2** (6.2 g, 17.8 mmol,

87%). N^4-benzoyl cytidine (6.0 g, 17.3 mmol) was dissolved in dry pyridine (30 ml), after evaporation with pyridine, and *tert*-butyldimethylsilyl chloride (50 wt% in toluene, 12.0 mL, 34.4 mmol) was added and the reaction mixture was stirred overnight at room temperature. Then, acetic anhydride was added and the reaction mixture was stirred for 2 hours. After TLC analysis indicated complete acetylation, the reaction mixture was concentrated*in* vacuo and the residue was redissolved in EtOAc (170 ml) and washed with sat. aq $NaHCO_3$ (50ml), 5% Citric acid (50ml), and H_2O (50ml). The organic layer was dried over MgSO4, filtered and evaporated *in vacuo* to give a yellow oil. The residue was dissolved in 4:1 MeCN/H_2O (170 mL, v/v) and excess *p*-TsOH (3.46 g, 28.4 mmol) was added after which the reaction mixture was stirred overnight at room temperature. The reaction mixture was concentrated and taken up in EtOAc, washed with sat. aq $NaHCO_3$ and water. The organic layer was dried over $MgSO_4$, filtered and evaporated *in vacuo*. The compound was purified with silica gel column chromatography DCM/MeOH (100/0 - 93/7 (Rf=0.35)) to yield **23** (5.9 g, 13.7 mmol, 80%). Spectroscopic data were in accord with those reported previously.[3] **^{1}H NMR** (300 MHz, $CDCl_3$): δ 8.77 (s, 1H), 8.21 (d, *J* = 7.4 Hz, 1H), 7.89 (d, *J* = 7.6 Hz, 2H), 7.67 – 7.46 (m, 4H), 6.08 (d, *J* = 4.9 Hz, 1H), 5.63 (t, *J* = 5.2 Hz, 1H), 5.52 (t, *J* = 5.0 Hz, 1H), 4.32 – 4.25 (m, 1H), 4.04 (d, *J* = 12.2 Hz, 1H), 3.87 (d, *J* = 12.3 Hz, 1H), 3.09 (s, 1H), 2.11 (d, *J* = 5.6 Hz, 6H).

Compound 24

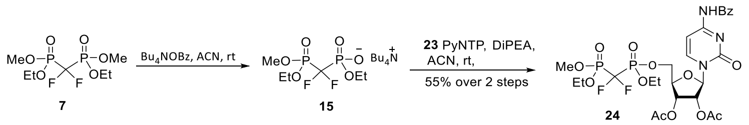

Compound **7** (200 mg, 0.68 mmol) was co-evaporated with toluene and dissolved in dry ACN (3ml),.Tetrabutylammonium benzoate (247 mg, 0.68 mmol) was added under nitrogen and the mixture was stirred overnight, whereafter the reaction was complete as indicated by ^{31}P NMR. N^4-benzoyl-2', 3'-di-*O*-acetyl cytidine **23** (440 mg, 1.02 mmol), dried prior to use by co-evaporation with toluene, DIPEA (0.79 ml, 10.0 eq)and PyNTP (1.02 g, 2.04 mmol) were added. After 2 h, the reaction was complete as indicated by TLC-analysis. The reaction mixture was poured into DCM and washed with phosphate buffer (pH = 7). The water layer was extracted with DCM. The combined organic was dried over Na_2SO4 and evaporated in vacuo. Purification by flash column chromatography (R_f = 0.24, 100% EA) followed by size exclusion (LH-20) (DCM/MeOH 1:1) furnished **24** as a pale yellow solid (258 mg, 0.37 mmol, 55%). *A mixture of diastereoisomers.* **^{1}H NMR** (400 MHz, $CDCl_3$) δ 8.77 (s, 1H), 8.27 – 8.11 (m, 1H), 7.90 (d, *J* = 7.6 Hz, 2H), 7.66 – 7.47 (m, 4H), 6.36 – 6.31 (m, 1H), 5.53 – 5.36 (m, 2H), 4.67 – 4.53 (m, 2H), 4.50 – 4.31 (m, 5H), 4.10 – 3.94 (m, 3H), 2.14 – 2.08 (m, 6H), 1.50 – 1.36 (m, 6H). ^{13}C NMR (101 MHz, $CDCl_3$) δ 169.7, 169.6, 169.5, 133.3, 129.1, 127.6, 87.8, 87.4, 80.8, 77.4, 77.1, 76.8, 73.7, 70.0, 69.7, 66.5, 29.3, 20.6, 20.5, 16.4. **^{31}P NMR** (162 MHz, $CDCl_3$): δ 6.25 – 2.07 (m). **^{19}F NMR** (471 MHz, $CDCl_3$): δ -120.81 – -123.19 (m). **HRMS** (ESI) M/Z: $[M+H]^+$: Calcd for $C_{26}H_{34}F_2N_3O_{13}P_2$ 696.1529; found 696.1549.

Fully protected sn-3 CF_2 CDP-Gro analogue (26)

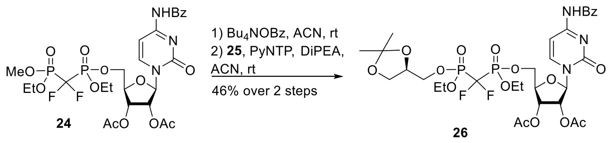

Compound **24** (430mg, 0.62 mmol) was co-evaporated with toluene,dissolved in dry ACN and tetrabutylammonium benzoate (225 mg, 0.62 mmol) was added under nitrogen and the mixture stirred overnight. After reaction completion, as indicated by ^{31}P NMR, (*S*)-solketal **25** (245 mg, 1.86 mmol), dried prior to addition by co-evaporating with toluene, DiPEA (1.0 ml, 6.2 mmol), and PyNTP (1.02 g, 2.04 mmol) were added successively. After 2 h, the reaction was complete as indicated by TLC-analysis. The reaction mixture was poured into DCM and washed with phosphate buffer (pH = 7). The water layer was extracted with DCM and the combined organics were dried over Na_2SO_4 and evaporated *in vacuo*. The crude product was purified by flash column chromatography over silica gel (R_f = 0.3, 100% EA) and then by size exclusion (LH-20) (DCM/MeOH 1:1) to obtain **26** (226 mg, 0.29 mmol, 46%) as a pale yellow solid. *A mixture of diastereoisomers.* **^{1}H NMR** (400 MHz, $CDCl_3$): δ 8.83 (s, 1H), 8.26 – 8.06 (m, 1H), 7.95 – 7.84 (m, 2H), 7.68 – 7.45 (m, 4H), 6.37 – 6.27 (m, 1H), 5.54 – 5.36 (m, 2H), 4.70 – 4.51 (m, 2H), 4.51 – 4.20 (m, 8H), 4.16 – 4.06 (m, 1H), 3.94 – 3.81 (m, 1H), 2.14 – 2.07 (m, 6H), 1.49 – 1.38 (m, 9H), 1.36 (s, 3H). **^{13}C NMR** (101 MHz, $CDCl_3$): δ 169.6, 169.5, 169.5, 169.3, 169.3, 169.3, 162.5, 144.6, 133.2, 129.0, 127.6, 110.0, 109.9, 87.8, 87.5, 80.7, 80.6, 77.4, 77.1, 76.7, 68.5, 66.6, 66.5, 66.4, 66.4, 66.1, 66.0, 65.8, 65.8, 65.7, 26.7, 26.6, 25.2, 20.5, 20.4, 16.3. **^{31}P NMR** (162 MHz, $CDCl_3$): δ 5.42 – 2.29 (m). **^{19}F NMR** (471 MHz, $CDCl_3$): δ -120.56 – -123.13 (m). **HRMS** (ESI) M/Z: $[M+H]^+$: Calcd for $C_{31}H_{42}F_2N_3O_{15}P_2$ 796.2087; found 796.2060.

Compound 27

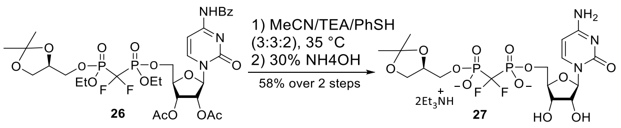

Fully protected *sn*-3 CF_2 CDP-Gro analogue **26** (30 mg, 0.11 mmol) was dissolved in MeCN: TEA: PhSH (3:3:2, 1 ml) and stirred for 6 hours at 35 ℃. Reaction progression was monitored by ^{31}P NMR and LC-MS, which indicated complete consumption of the starting material. The solution was concentrated in vacuo. The residue was dissolved in conc. ammonia (30wt%, 1.5 ml) and stirred overnight. The reaction mixture was concentrated in vacuo. The resulting mixture was diluted with water then acidified to pH 6 -7 using acetic acid and poured into a separation funnel. The mixture was washed with DCM (3x) to remove excess thiophenol. The water layer was concentrated in vacuo and purified by HPLC to give compound **27** as a white powder (12 mg, 21 μmol, 58% over 2 steps). **^{1}H NMR** (400 MHz, D_2O): δ 7.97 (d, *J* = 8.0 Hz, 1H), 6.08 (d, *J* = 8.0 Hz, 1H), 5.94 (d, *J* = 4.0 Hz, 1H), 4.40 – 4.34 (m, 1H), 4.32 – 4.21 (m, 4H), 4.13 – 4.09 (m, 1H), 4.07 – 3.96 (m, 1H), 3.84 (dd, *J* = 8.0, 4.0 Hz, 1H), 1.41 (s, 3H), 1.34 (s, 3H). **^{13}C NMR** (101 MHz, D_2O): δ 180.6, 165.2, 156.5, 141.8, 110.1, 96.3, 89.1, 82.7, 74.8, 74.1, 69.1, 66.4, 65.2, 64.8, 25.4, 24.1, 22.7. **^{31}P NMR** (162 MHz, D_2O): δ 3.47, 3.44 (ABX, J_{P-F} = 82.6 Hz). **^{19}F NMR** (376 MHz, D_2O): δ -118.88 (t, J_{P-F} = 82.7 Hz). **HRMS** (ESI) M/Z: $[M+H]^+$: Calcd for $C_{16}H_{26}F_2N_3O_{12}P_2$ 552.0954; found 552.0963.

sn-3 CF_2-CDP-Gro analogue (28)

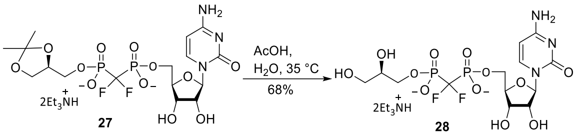

Compound **27** (12mg, 21 μmol) was dissolved in water (0.5 ml) and acetic acid (0.2 ml) was added. The reaction mixture was stirred at 35 °C for 6 hours. After analysis by LC-MS showed complete consumption of the starting material, the mixture was concentrated in vacuo. The compound was purified by size-exclusion chromatography (HW40-column, 0.15 M aqueous NH_4OAc in water) followed by lyophilization to yield *sn*-3 CF_2-CDP-Gro **28** (8.8 mg, 0.29 mmol, 68%) as a white powder. **^{1}H NMR** (400 MHz, D_2O): δ 8.09 (d, J = 8.0 Hz, 1H), 6.15 (d, J = 8.0 Hz, 1H), 5.93 (d, J = 4.0 Hz, 1H), 4.35 – 4.22 (m, 5H), 4.08 – 4.03 (m, 1H), 4.01 – 3.95 (m, 1H), 3.88 – 3.83 (m, 1H), 3.64 (dd, J = 12.0, 4.0 Hz, 1H), 3.56 (dd, J = 12.0, 8.0 Hz, 1H). **^{13}C NMR** (101 MHz, D_2O): δ 162.5, 152.9, 142.9, 121.8, 120.9, 119.1, 116.6, 95.8, 89.3, 83.1, 83.0, 83.0, 74.3, 70.9, 70.8, 70.8, 69.1, 67.2, 67.1, 67.1, 64.8, 61.9. **^{31}P NMR** (162 MHz, D_2O): δ 3.47, 3.44 (ABX, $J_{P\text{-}F}$ = 82.6 Hz). **^{19}F NMR** (471 MHz, D_2O): δ -119.17 (t, $J_{P\text{-}F}$ = 84.8 Hz). **HRMS** (ESI) M/Z: $[M+H]^+$: Calcd for $C_{13}H_{22}F_2N_3O_{12}P_2$ 512.0641; found 512.0654. $[\alpha]^{20}_D$ = +10 ° (c = 0.001, H_2O).

Fully protected sn-1 CF_2-CDP-Gro analogue (30)

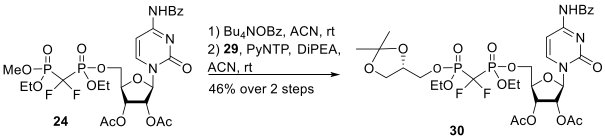

Compound **24** (150 mg, 0.22 mmol) was co-evaporated with toluene dissolved in dry ACN, and tetrabutylammonium benzoate (78 mg, 0.22 mmol) was added under nitrogen and the mixture stirred overnight. The reaction was complete as indicated by ^{31}P NMR. *R*-solketal **29** (87 mg, 0.66 mmol) was added after being dried by evaporation with toluene together with DiPEA (0.37 ml, 2.2 mmol), and PyNTP (330 mg, 2.04 mmol). After 2 h, the reaction was complete as indicated by TLC-analysis. The reaction mixture was poured into DCM and washed with pH=7 phosphate buffer. The water layer was extracted with DCM. The combined organic was dried over Na_2SO_4 and evaporated in vacuo. The crude product was purified by flash column chromatography over silica gel (Rf = 0.3, 100% EA) and then by size exclusion (LH-20) (DCM/MeOH 1:1) to obtain **30** (279 mg, 0.35 mmol, 57%) as a yellow solid. *A mixture of diastereoisomers.* **^{1}H NMR** (400 MHz, $CDCl_3$): δ 8.93 (s, 1H), 8.26 – 8.06 (m, 1H), 7.96 – 7.83 (m, 2H), 7.67 – 7.43 (m, 4H), 6.33 – 6.27 (m, 1H), 5.52 – 5.33 (m, 2H), 4.67 – 4.49 (m, 2H), 4.46 – 4.16 (m, 7H), 4.13 – 3.96 (m, 1H), 3.90 – 3.79 (m, 1H), 2.12 – 2.03 (m, 6H), 1.47 – 1.36 (m, 9H), 1.34 (s, 3H). **^{13}C NMR** (101 MHz, $CDCl_3$): δ 169.7, 169.6, 169.5, 169.4, 169.4, 162.6, 144.8, 133.3, 129.1, 127.7, 117.8, 116.0, 110.1, 110.09, 110.0, 87.9, 87.6, 80.8, 80.7, 80.7, 77.4, 77.1, 76.8, 74.2, 74.1, 74.1, 74.1, 74.0, 73.7, 73.7, 70.0, 69.9, 69.7, 69.0, 69.0, 68.9, 68.9, 68.6, 68.5, 68.5, 66.7, 66.6, 66.6, 66.5, 66.5, 66.4, 66.3, 66.3, 66.2, 66.1, 65.9, 65.9, 65.9, 65.7, 26.8, 26.7, 26.7, 26.6, 25.3, 25.3, 20.6, 20.5, 16.6, 16.5, 16.5, 16.4. **^{31}P NMR** (162 MHz, $CDCl_3$): δ 5.41 – 2.29 (m). **^{19}F NMR** (471 MHz, $CDCl_3$): δ -

120.95 – -123.42 (m). **HRMS** (ESI) M/Z: $[M+H]^+$: Calcd for $C_{31}H_{42}F_2N_3O_{15}P_2$ 796.2053; found 796.2057.

Compound 31

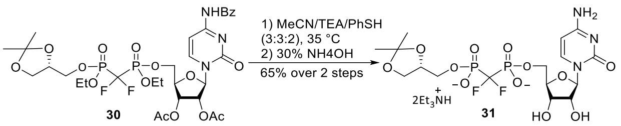

Fully protected *sn*-1 CF_2 CDP-Gro analogue **30** (88 mg, 0.11 mmol) was dissolved in MeCN: TEA: PhSH (3:3:2, 2 ml) and stirred for 6 hours at 35 °C. Reaction progression was monitored by ^{31}P NMR and LC-MS, which indicated complete consumption of the starting material. The solution was concentrated *in vacuo* redissolved in aqueous ammonia (30, 3 ml) and stirred overnight. The reaction mixture was concentrated in vacuo. The resulting mixture was diluted with water then acidified to pH 6 -7 using acetic acid and poured into a separation funnel. The mixture was washed with DCM (3x) to remove excess thiophenol. The water layer was concentrated *in vacuo*. The residue was purified by HPLC to give compound **31** as a white powder (41 mg, 72 µmol, 65% over 2 steps). **^{1}H NMR** (400 MHz, D_2O): δ 7.90 (d, *J* = 8.0 Hz, 1H), 6.03 (d, *J* = 8.0 Hz, 1H), 5.91 (d, *J* = 4.0 Hz, 1H), 4.38 – 4.31 (m, 1H), 4.31 – 4.15 (m, 5H), 4.10 – 4.05 (m, 1H), 4.05 – 3.99 (m, 1H), 3.99 – 3.91 (m, 1H), 3.81 (dd, *J* = 8.0, 4.0 Hz, 1H), 1.37 (s, 3H), 1.31 (s, 3H). **^{13}C NMR** (101 MHz, D_2O): δ 180.6, 165.3, 156.6, 141.8, 121.2, 119.2, 117.2, 110.1, 96.3, 89.1, 82.7, 82.7, 82.7, 74.8, 74.8, 74.7, 74.15, 69.1, 66.3, 65.2, 64.8, 25.4, 24.1, 22.7. **^{31}P NMR** (162 MHz, D_2O): δ 4.11, 4.08 (ABX, $J_{P\text{-}F}$ = 84.8 Hz). **^{19}F NMR** (376 MHz, D_2O): δ -119.01 (t, $J_{P\text{-}F}$ = 82.7 Hz). **HRMS** (ESI) M/Z: $[M+H]^+$: Calcd for $C_{16}H_{26}F_2N_3O_{12}P_2$ 552.0954; found 552.0961. $[\alpha]^{20}_D$ = +16 ^{o} (c = 0.001, H_2O).

sn-1 CF_2 CDP-Gro analogue (32)

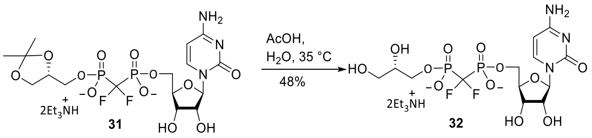

Compound **31** (41mg, 72 µmol) was dissolved in water (0.5 ml) and acetic acid (0.2 ml) was added. The reaction mixture was stirred at 35 °C for 6 hours. After analysis by LC-MS showed complete consumption of the starting material, the mixture was concentrated in vacuo. The compound was purified by size-exclusion chromatography (HW40-column, 0.15 M aqueous NH_4OAc in water) followed by lyophilization to yield *sn*-1 CF_2 CDP-Gro analogue **32** (18 mg, 35 µmol, 48%) as a white powder. **^{1}H NMR** (400 MHz, D_2O): δ 8.05 (d, *J* = 8.0 Hz, 1H), 6.13 (d, *J* = 8.0 Hz, 1H), 5.93 (d, *J* = 4.0 Hz, 1H), 4.36 – 4.22 (m, 5H), 4.10 – 4.03 (m, 1H), 4.01 – 3.96 (m, 1H), 3.89 – 3.83 (m, 1H), 3.64 (dd, *J* = 12.0, 4.0 Hz, 1H), 3.56 (dd, *J* = 12.0, 8.0 Hz, 1H). **^{13}C NMR** (101 MHz, D_2O): δ 163.1, 153.7, 142.6, 121.8, 120.8, 119.1, 117.4, 95.9, 89.2, 82.9, 82.9, 82.9, 74.2, 70.8, 70.8, 70.7, 69.0, 67.1, 67.1, 67.0, 64.8, 64.7, 61.9. **^{31}P NMR** (162 MHz, D_2O): δ 3.66, 3.64 (ABX, $J_{P\text{-}F}$ = 82.6 Hz). **^{19}F NMR** (471 MHz, D_2O): δ -119.13 (t, $J_{P\text{-}F}$ = 80.1 Hz). **HRMS** (ESI) M/Z: $[M+H]^+$: Calcd for $C_{13}H_{22}F_2N_3O_{12}P_2$ 512.0641; found 512.0652.

6-N-benzoyl-2,3-*bis*-O-tert-butyldimethylsilylcytidine (33)

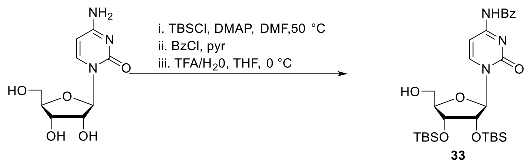

Commercially available cytidine (10 g, 41 mmol) was co-evaporated thrice with anhydrous pyridine , dissolved in anhydrous dimethylformamide (80 mL) and heated to 50 °C in an oil-bath, whereupon imidazole (14 g, 205 mmo) and *tert*-butyldimethylsilyl chloride (31 g, 205 mmol) were added consecutively and the resulting mixture stirred overnight. The reaction mixture was quenched by the addition of H_2O (30 mL), diluted with Et_2O (200 mL), and washed with H_2O (100 mL). The aqueous layer was back-extracted twice with Et_2O, and the resulting organic layers were combined, washed with water and brine, dried over MgSO4, filtered, and concentrated *in vacuo*. The crude product was co-evaporated with toluene three times, placed under a nitrogen atmosphere and dissolved in pyridine (110 ml), whereupon benzoyl chloride (28.8 g, 205 mmol) was added and the mixture was stirred at room temperature for 5 hours. After analysis by TLC showed complete consumption of the starting material, the reaction was quenched with sat. aq $NaHCO_3$ at 0 °C, diluted with EA and washed with water and brine. The organic layer was dried over MgSO4, filtered, and concentrated *in vacuo*. The crude product was taken up in THF (400 mL), and aqueous TCA (212 g in 100 mL H_2O) was added at 0 °C. After stirring for 3 h at 0 °C, the reaction was quenched with sat. aq $NaHCO_3$ and diluted with dichloromethane (500 mL). The water layer was back-extracted twice with dichloromethane (200 ml). The combined organic layers were dried over Na_2SO_4 and concentrated. The residue was purified by flash column chromatography (R_f = 0.2, PE/EA 3:1) to give title nucleoside **33** as a white solid (18.7 g, 31.8 mmol, 77%). Spectral data was in accordance with literary precedence.[4] **^{1}H NMR** (300 MHz, $CDCl_3$): δ 8.84 (s, 1H), 8.12 (d, *J* = 6.0 Hz, 1H), 7.96 – 7.86 (m, 2H), 7.66 – 7.44 (m, 4H), 5.48 (d, *J* = 6.0 Hz, 1H), 4.73 (t, *J* = 6.0 Hz, 1H), 4.19 (dd, *J* = 9.0, 3.0 Hz, 1H), 4.01 (dd, *J* = 12.0, 3.0 Hz, 1H), 3.74 (d, *J* = 12.0 Hz, 1H), 0.09 – 0.06 (m, 9H), 0.04 (s, 3H).

Conpound (34)

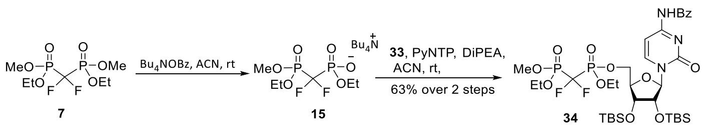

Compound **7** (100 mg, 0.34 mmol) was co-evaporated with toluene,dissolved in dry ACN (11.5 ml) and treated with tetrabutylammonium benzoate (124 mg, 0.34 mmol) under a nitrogen atmosphere and stirred overnight. The reaction was complete as indicated by ^{31}P NMR. Compound **33** (358 mg, 0.51 mmol) was added after being dried by evaporation with toluene, DiPEA (0.58 ml, 3.4 mmol eq) and PyNTP (509 mg, 1.02 mmol) were added. After 2 h, the reaction was complete as indicated by TLC-analysis and was poured into DCM and washed with phosphate buffer (pH = 7). The water layer was extracted with DCM and the combined organics were dried over Na_2SO_4 and evaporated *in vacuo*. The crude product was purified by flash column chromatography over silica gel (Rf = 0.3, PE/EA 1:1,) and then by size exclusion (LH-20) (DCM/MeOH 1:1) to obtain **34** (178 mg, 0.21 mmol, 63% over 2 steps) as a yellow solid. *A mixture of diastereoisomers.* **^{1}H NMR** (400 MHz, $CDCl_3$): δ 8.67 (s, 1H), 8.39 – 8.18 (m, 1H),

7.95 – 7.81 (m, 2H), 7.65 – 7.46 (m, 4H), 5.85 – 5.77 (m, 1H), 4.78 – 4.65 (m, 1H), 1.49 – 1.39 (m, 6H), 0.91 (s, 5H), 0.89 (s, 8H), 0.27 – 0.20 (m, 3H), 0.17 – 0.11 (m, 3H), 0.11 – 0.03 (m, 6H). **^{13}C NMR** (101 MHz, $CDCl_3$): δ 162.5, 145.0, 133.2, 129.1, 127.6, 118.8, 117.8, 116.4, 116.1, 91.5, 81.2, 81.0, 77.4, 77.1, 76.8, 75.7, 69.7, 69.6, 66.7, 66.6, 66.4, 66.1, 66.0, 66.0, 65.8, 65.6, 55.6, 55.6, 29.8, 26.0, 25.9, 18.1, 18.1, 16.6, 16.5, 16.5, -4.0, -4.1, -5.0, -5.0, -5.0. **^{31}P NMR** (162 MHz, $CDCl_3$): δ 5.66 –2.45 (m). **^{19}F NMR** (376 MHz, $CDCl_3$): δ -119.99 – -122.63. **HRMS** (ESI) M/Z: $[M+H]^+$: Calcd for $C_{34}H_{58}F_2N_3O_{11}P_2Si_2$ 840.3047; found 840.3061.

Synthesis of compound 35

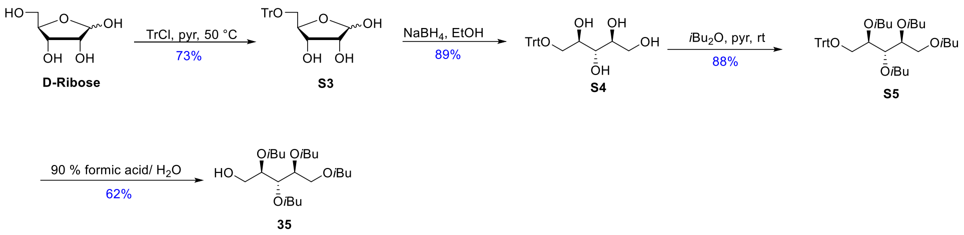

5-*O*-trityl-D-ribitol (S4)

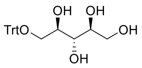
S4

D-Ribose (5.1g, 34 mmol) was coevaporated with toluene three times under nitrogen and dissolved in pyridine (110 ml), trityl chloride (9.4g, 34 mmol) was added and the reaction mixture was heated to 50 °C in an oil-bath for 6 h whereupon the solution became homogenous, the reaction mixture the flask was cooled to room temperature and the pyridine was removed under reduced pressure. The resulting oil was subsequently redissolved in DCM, washed with water, The aqueous layer was extracted with DCM and the combined organic layers were dried over Na_2SO_4 and concentrated in vacuo. The residue was again taken up in DCM, and added dropwise with stirring to a mixture of hexane (18 mL) and DCM (2 mL), the white crystals were collected by filtration and washed with hexane and water. Crystals were dried under a high vacuum at 50 °C to yield 5-*O*-triphenylchloromethyl-D-ribose (9.6g, 24.4 mmol, 73%). 5-*O*-triphenylchloromethyl-D-ribose (3g, 7.6 mmol) was dissolved in anhydrous ethanol and cooled to 0 °C, sodium tetrahydroborate (1.73g, 45.6 mmol) was slowly added. The reaction mixture was warmed to rt and stirred overnight. After analysis by TLC showed complete consumption of the starting material, the reaction mixture was quenched with acetic acid, and concentrated *in vacuo*. The residue was diluted in EtOAc and washed with sat. aq $NaHCO_3$, water, and brine. The organic layer was dried over anhydrous MgSO4, filtered, and concentrated in vacuo, and the crude product was purified by flash column chromatography (R_f = 0.3, DCM/MeOH 10:1) to give a white solid (2.7 g, 6.8 mmol, 89%). **^{1}H NMR** (400 MHz, $CDCl_3$): δ 7.45 – 7.38 (m, 6H), 7.30 – 7.24 (m, 6H), 7.23 – 7.18 (m, 3H), 3.85 – 3.78 (m, 1H), 3.75 – 3.60 (m, 5H), 3.47 – 3.41 (m, 2H), 3.39 (d, J = 4.0 Hz, 1H), 3.37 – 3.33 (m, 1H), 3.30 (dd, J = 12.0, 8.0 Hz, 1H). **^{13}C NMR** (101 MHz, $CDCl_3$): δ 143.5, 128.66, 128.1, 127.3, 87.4, 73.2, 72.8, 71.6, 65.2, 63.3. **HRMS** (ESI) M/Z: $[M+Na]^+$: Calcd for $C_{24}H_{26}O_5Na$ 417.1672; found 417.1676.

1,2,3,4-tetra-O-isobutyryl-5-O-trityl-D-ribitol (S5).

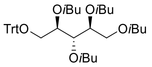
S5

5-*O*-trityl-D-ribitol **16** (3.5 g, 9.0 mmol) was co-evaporated with toluene three times, placed under a nitrogen atmosphere and dissolved in pyridine (36 ml). 4-Dimethylaminopyridine (4.95 g, 40.5 mmol) and isobutyric anhydride (9 ml, 54

mmol) were added and the mixture was stirred overnight at rt. The reaction mixture was concentrated *in vacuo* and taken up in DCM. After washing with 5% citric acid and water, the organic phase was dried over MgSO4 and concentrated *in vacuo*. The crude product was purified by flash column chromatography (R_f = 0.2, PE/EA 20:1) to give **S5** as a colorless oil (4.8 g, 7.9 mmol, 88%).**^{1}H NMR** (400 MHz, $CDCl_3$): δ 7.46 – 7.37 (m, 6H), 7.36 – 7.21 (m, 9H), 5.48 – 5.36 (m, 2H), 5.34 – 5.26 (m, 1H), 4.35 (dd, *J* = 12.0, 4.0 Hz, 1H), 4.15 (dd, *J* = 12.0, 8.0 Hz, 1H), 3.32 (dd, *J* = 8.0, 4.0 Hz, 1H), 3.23 (dd, *J* = 12.0, 8.0 Hz, 1H), 2.75 – 2.60 (m, 1H), 2.59 – 2.47 (m, 1H), 2.49 – 2.34 (m, 2H), 1.27 (d, *J* = 7.1 Hz, 3H), 1.25 (d, *J* = 8.0 Hz, 3H), 1.17 – 1.10 (m, 12H), 1.07 (d, *J* = 8.0 Hz, 3H), 1.04 (d, *J* = 8.0 Hz, 3H). **^{13}C NMR** (101 MHz, $CDCl_3$): δ 176.6, 176.0, 175.7, 175.1, 144.7, 144.0, 143.9, 143.7, 143.5, 128.7, 127.9, 127.1, 86.7, 70.7, 69.6, 69.4, 61.9, 61.90, 34.2, 33.9, 33.9, 33.9, 19.0, 19.0, 18.9, 18.9, 18.8, 18.7. **HRMS** (ESI) M/Z: $[M+Na]^+$: Calcd for $C_{40}H_{50}O_9Na$ 697.3347; found 697.3364.

1,2,3,4-tetra-O-isobutyryl-D-ribitol (35).

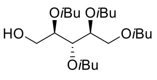

35

p-Toluenesulfonic acid (1.2 g, 6.4 mmol), dissolved in MeOH-DCM (1:1, 6 mL), was added dropwise over 1 h to a solution of **S5** (2.0 g, 3.2 mmol) in MeOH-DCM (1:1, 20 mL). The reaction mixture was stirred at rt for 3 h until complete conversion was confirmed by TLC analysis. Hereafter, solid $NaHCO_3$ was added, the turbid mixture filtered and the filtrate evaporated *in vacuo*. The residue was purified by flash column chromatography (Rf= 0.26, PE-EA, 3:1) to give compound **35** as a colorless oil (867 mg, 1.98 mmol, 62 %). **^{1}H NMR** (400 MHz, $CDCl_3$): δ 5.41 – 5.31 (m, 2H), 5.09 – 5.04 (m, 1H), 4.39 (dd, *J* = 12.0, 4.0 Hz, 1H), 4.20 – 4.11 (m, 1H), 3.83 (d, *J* = 16.0 Hz, 1H), 3.72 – 3.62 (m, 1H), 2.69 – 2.44 (m, 4H), 1.70 (s, 1H), 1.22 – 1.11 (m, 24H). **^{13}C NMR** (101 MHz, $CDCl_3$): δ 176.7, 176.5, 176.1, 175.9, 72.4, 69.7, 61.9, 61.2, 34.1, 19.0. **HRMS** (ESI) M/Z: $[M+Na]^+$: Calcd for $C_{21}H_{36}O_9Na$ 455.2251; found 455.2260.

Fully protected CF_2 CDP-Ribitol analogue (36)

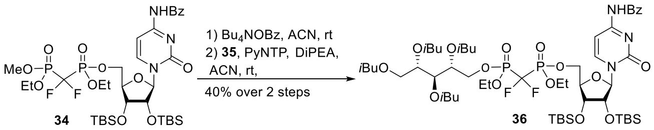

Compound **34** (400 mg, 0.48 mmol) was co-evaporated with toluene dissolved in dry ACN (2.0 ml) and treated with tetrabutylammonium benzoate (175 mg, 0.48 mmol) under a nitrogen atmosphere and stirred overnight. Compound **35** (311 mg, 0.72 mmol), dried by evaporation with toluene, DiPEA (816 µl, 4.8 mmol) and PyNTP (719 mg, 1.44 mmol) were added successively. After 2 h, the reaction was complete as indicated by TLC-analysis. The reaction mixture was poured into DCM and washed with phosphate buffer (pH = 7). The water layer was extracted with DCM. The combined organics were dried over Na_2SO_4 and evaporated *in vacuo*. The crude product was purified by flash column chromatography over silica gel (Rf = 0.24, PE/EA 2:1) and then by size exclusion (LH-20) (DCM/MeOH 1:1) to obtain **36** (282 mg, 0.23 mmol, 48%) as a colorless oil. *A mixture of diastereoisomers.* **^{1}H NMR** (400 MHz, $CDCl_3$): δ 8.72 (s, 1H), 8.34 – 8.17 (m, 1H), 7.94 – 7.84 (m, 2H), 7.64 – 7.44 (m, 3H), 5.83 – 5.74 (m, 1H), 5.42 – 5.25 (m, 3H), 4.77 – 4.47 (m, 2H), 4.47 – 4.25 (m, 8H), 4.25 – 4.19 (m, 1H), 4.17 – 4.09 (m, 1H), 4.07 – 3.91 (m, 1H), 2.68 – 2.45 (m, 4H), 1.48 – 1.34 (m, 6H), 1.22 – 1.07 (m, 24H), 0.93 – 0.88 (m, 9H), 0.90 –

0.85 (m, 9H), 0.26 – 0.18 (m, 3H), 0.16 – 0.08 (m, 3H), 0.09 – 0.02 (m, 6H). **^{13}C NMR** (101 MHz, $CDCl_3$): δ 176.5, 176.5, 175.9, 175.9, 175.8, 175.8, 175.2, 175.2, 175.1, 162.3, 144.9, 133.2, 129.1, 127.6, 119.4, 118.0, 116.1, 91.6, 91.4, 81.2, 80.9, 80.9, 77.4, 77.1, 76.8, 75.7, 75.7, 69.8, 69.8, 69.7, 69.7, 69.6, 69.5, 69.5, 69.4, 69.4, 68.6, 68.6, 66.8, 66.7, 66.7, 66.6, 66.5, 66.5, 66.4, 66.4, 66.3, 66.2, 66.2, 65.9, 65.7, 61.7, 61.6, 34.0, 34.0, 34.0, 33.8, 25.9, 25.9, 18.9, 18.9, 18.8, 18.8, 18.1, 18.0, 16.6, 16.5, 16.5, 16.4, 16.4, 16.3, -4.0, -4.1, -4.15, -4.1, -5.0, -5.0, -5.1. **^{31}P NMR** (162 MHz, $CDCl_3$): δ 5.33 – 2.01 (m). **^{19}F NMR** (376 MHz, $CDCl_3$): δ -120.30 – -123.12 (m). **HRMS** (ESI) M/Z: $[M+H]^+$: Calcd for $C_{54}H_{90}F_2N_3O_{19}P_2Si_2$ 1240.5144; found 1240.5144.

Compound 37

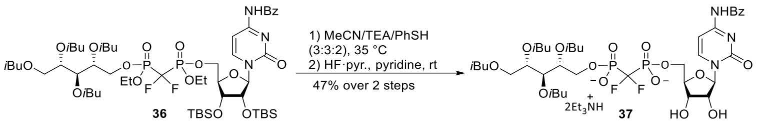

Full protected CDP-ribitol analogue **36** (55 mg, 0.11 mmol) was dissolved in MeCN: TEA: PhSH (3:3:2, 0.4 ml) and stirred for 6 hours at 35 °C. Reaction progression was monitored by ^{31}P NMR and LC-MS, which indicated complete consumption of the starting material. The solution was concentrated in vacuo. The residue was dissolved in pyridine and cooled to 0 °C, after which HF●pyridine (70 wt%, 31 µL, 1. 2 mmol) was added and stirred overnight at rt. The resulting solution was quenched with sat. aq $NaHCO_3$ and concentrated. The resulting mixture was diluted with water then acidified to pH 6-7 using acetic acid. The mixture was washed with DCM (3x) to remove excess thiophenol and pyridine. The water layer was concentrated and purified by HPLC to give compound **37** as a white powder (20 mg, 51 µmol, 47% over 2 steps). **^{1}H NMR** (400 MHz, D_2O): δ 8.57 (d, J = 8.0 Hz, 1H), 7.92 – 7.85 (m, 2H), 7.70 – 7.61 (m, 1H), 7.58 – 7.48 (m, 3H), 5.95 (d, J = 4.0 Hz, 1H), 5.31 – 5.19 (m, 3H), 4.46 – 4.39 (m, 1H), 4.37 – 4.22 (m, 6H), 4.20 – 4.12 (m, 1H), 4.08 (dd, J = 12.0, 4.0 Hz, 1H), 2.66 – 2.38 (m, 4H), 1.11 – 0.97 (m, 24H). **^{13}C NMR** (101 MHz, D_2O): δ 179.2, 178.8, 178.3, 177.7, 169.3, 156.7, 145.9, 133.6, 132.5, 128.9, 128.0, 98.4, 90.7, 82.5, 74.8, 71.2, 69.7, 68.9, 68.1, 64.0, 63.6, 61.5, 33.9, 33.8, 33.8, 33.7, 18.1, 17.9, 17.9. **^{31}P NMR** (162 MHz, D_2O): δ δ 3.47 (dt, J_{P-F} = 84.2 Hz, J_{P-P}= 55.1Hz, α-P), 2.77(dt, J_{P-F} = 84.2 Hz, J_{P-P}= 55.1Hz, β-P). **^{19}F NMR** (376 MHz, D_2O): δ -118.87, 118.89 (ABX, J_{P-F} = 82.7 Hz). **HRMS** (ESI) M/Z: $[M+H]^+$: Calcd for $C_{38}H_{54}F_2N_3O_{19}P_2$ 956.2789; found 956.2797.

CF_2 CDP-Ribitol analogue(38)

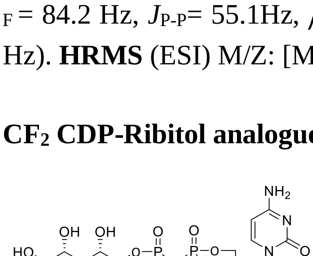

Compound **37** (18 mg, 0.019 mmol) was dissolved in aqueous ammonia (30 wt%, 2 mL) and stirred overnight. The excess ammonia was removed by stirring under vacuum. The crude product was purified by size-exclusion chromatography (HW40-column, 0.15 M aqueous NH_4OAc in water) followed by lyophilization to yield CF_2 CDP-ribitol analogue **38** (10.0 mg, 6.3 µmol, 33%) as a white powder. **^{1}H NMR** (400 MHz, D_2O): δ 8.08 (d, J = 8.0 Hz, 1H), 6.15 (d, J = 8.0 Hz, 1H), 5.93 (d, J = 4.0 Hz, 1H), 4.38 – 4.16 (m, 6H), 4.13 – 4.06 (m, 1H), 3.91 – 3.80 (m, 2H), 3.76 (dd, J = 12.0, 4.0 Hz, 1H), 3.74 – 3.69 (m, 1H), 3.60 (dd, J = 12.0, 8.0 Hz, 1H). **^{13}C NMR** (101 MHz, D_2O): δ 162.7, 153.2, 142.8, 123.3, 121.8, 120.8, 119.1, 117.4, 95.9, 89.2, 83.0, 83.0, 74.3, 72.0, 71.6, 70.9, 70.9, 69.1, 67.4, 67.3, 64.8, 64.8, 62.2. **^{31}P NMR** (162 MHz, D_2O): δ 3.78，3.72 (ABX, J_{P-F} = 82.6 Hz). **^{19}F NMR** (471 MHz, D_2O): δ -119.21 (t,

J_{P-F} = 84.8 Hz,). **HRMS** (ESI) M/Z: $[M+H]^+$: Calcd for $C_{15}H_{26}F_2N_3O_{14}P_2$ 572.0852; found 572.0867.

NMR Spectra

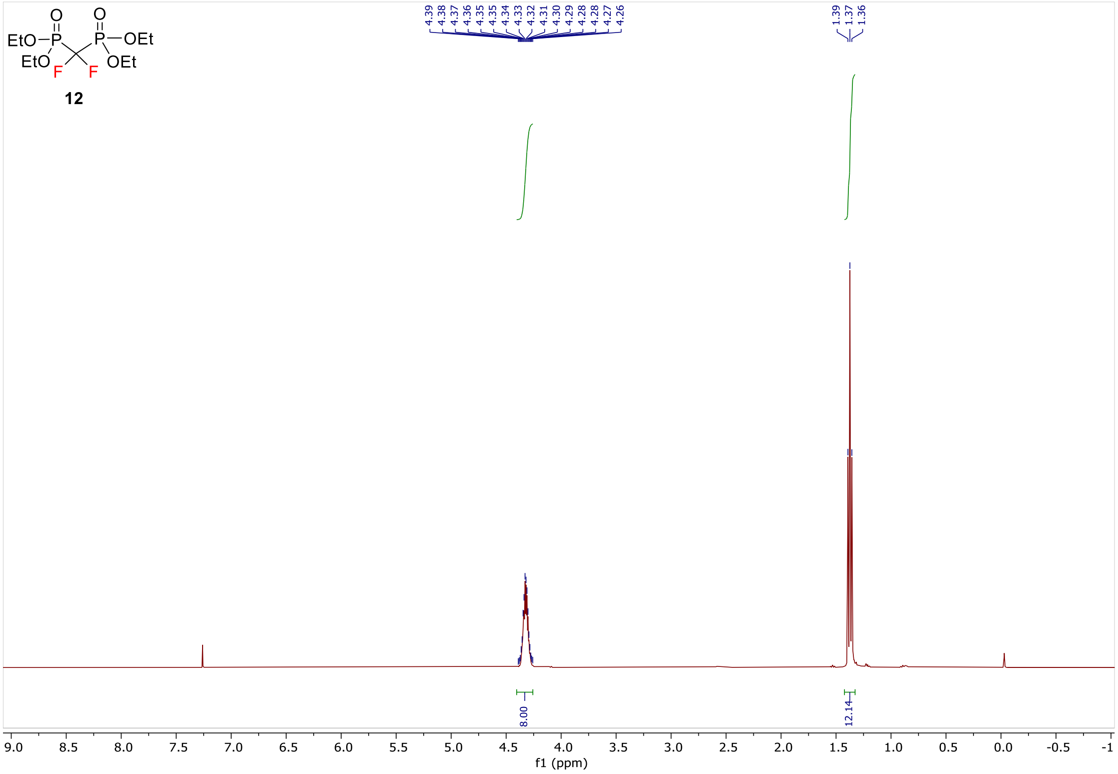

^{1}H NMR (400 MHz, $CDCl_3$) of compound **12**

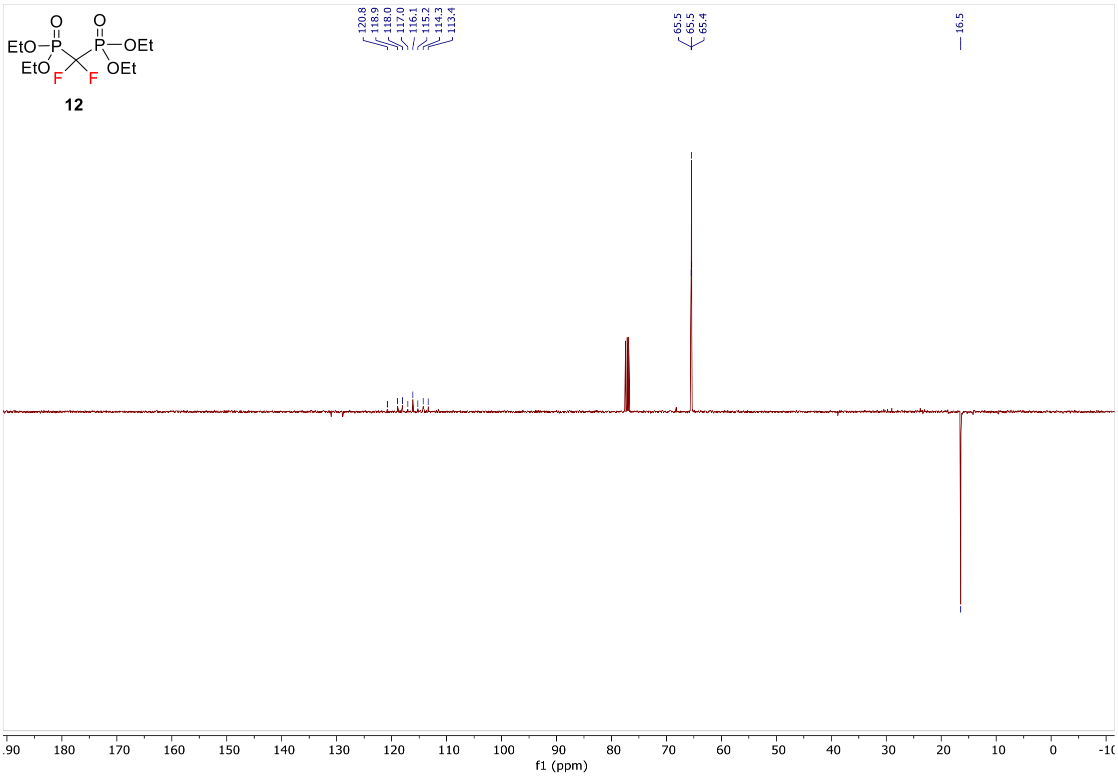

^{13}C NMR (101 MHz, $CDCl_3$) of compound **12**

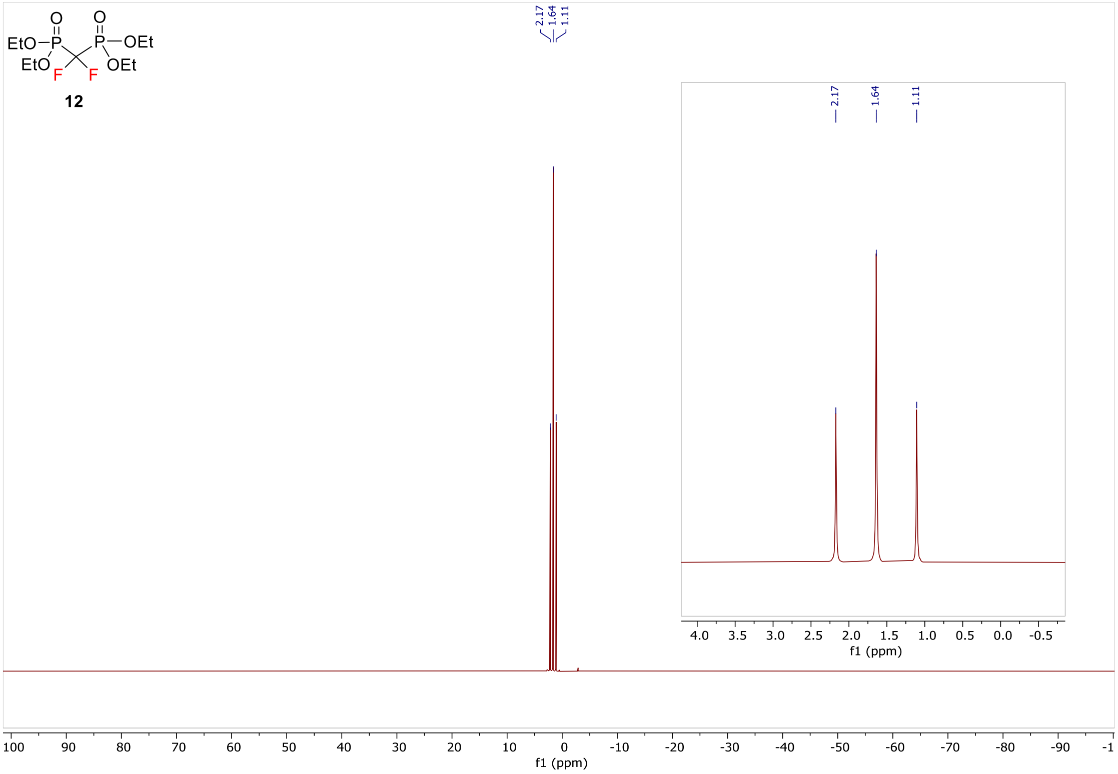

^{31}P NMR (162 MHz, $CDCl_3$) of compound **12**

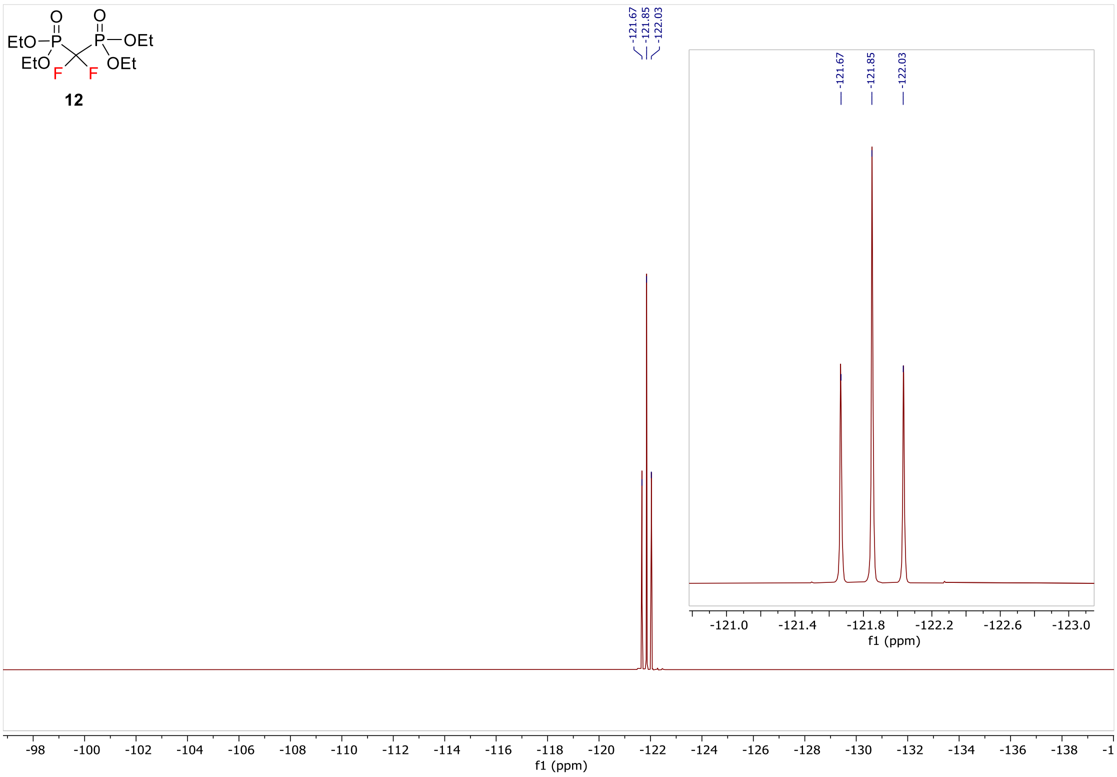

^{19}F NMR (471 MHz, $CDCl_3$) of compound **12**

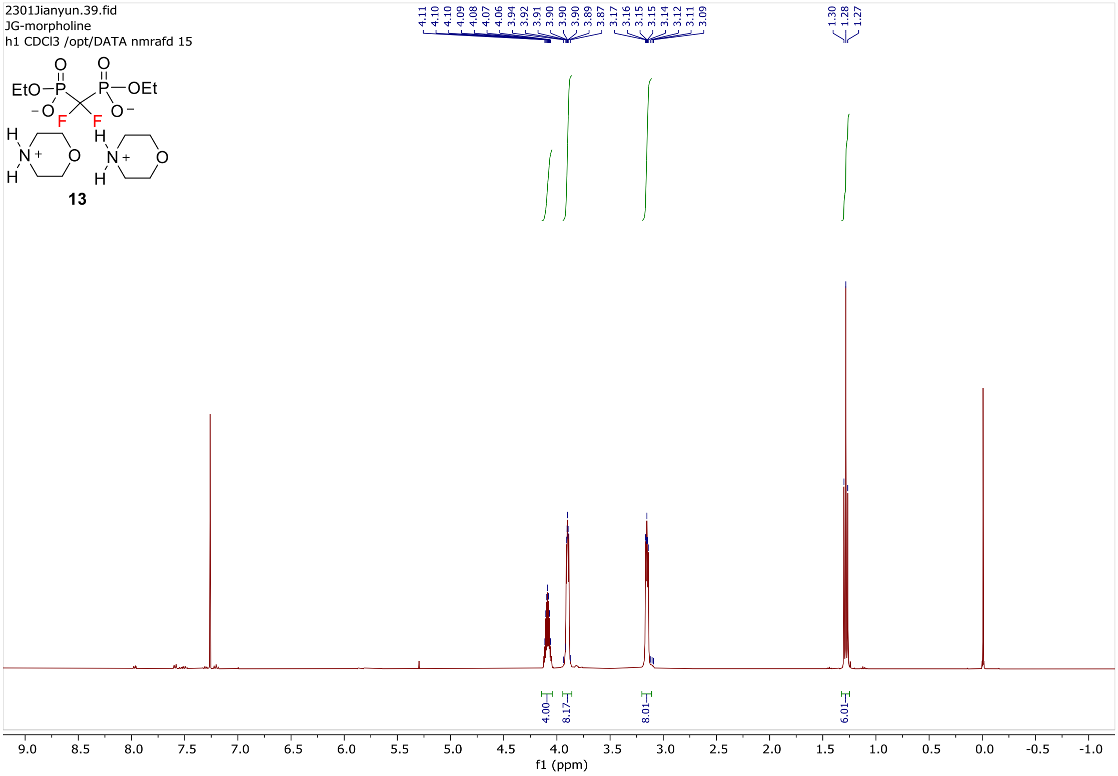

^{1}H NMR (400 MHz, $CDCl_3$) of compound **13**

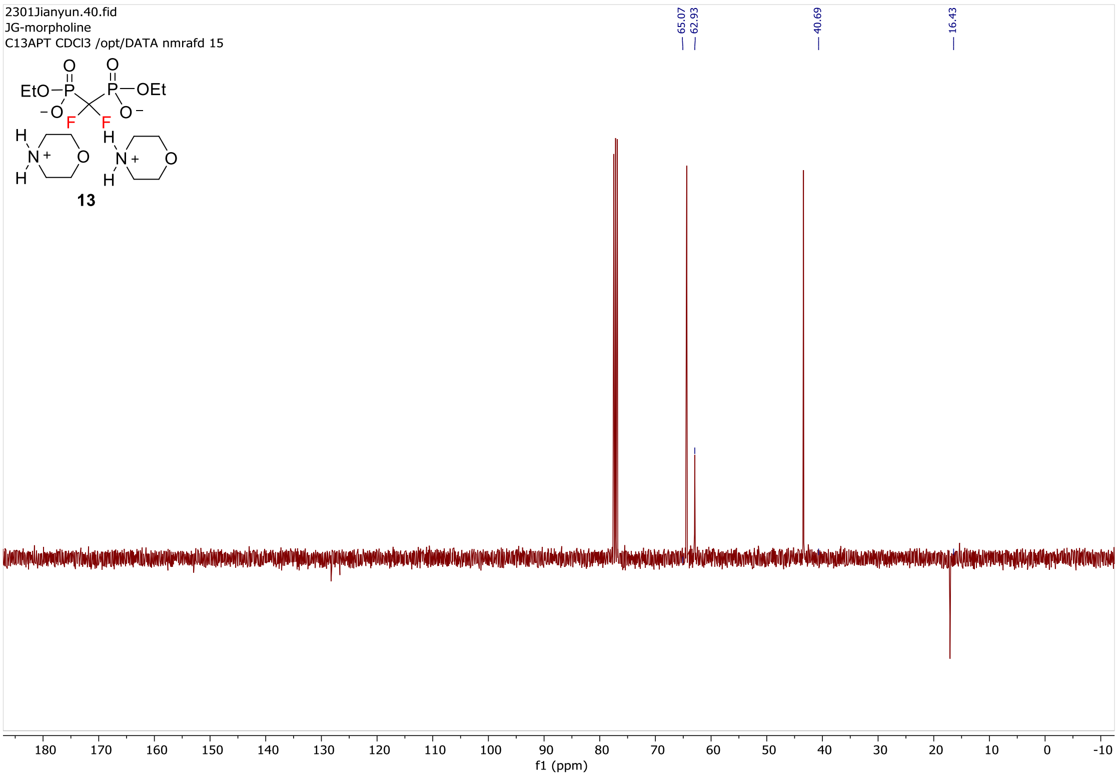

^{13}C NMR (101 MHz, $CDCl_3$) of compound **13**

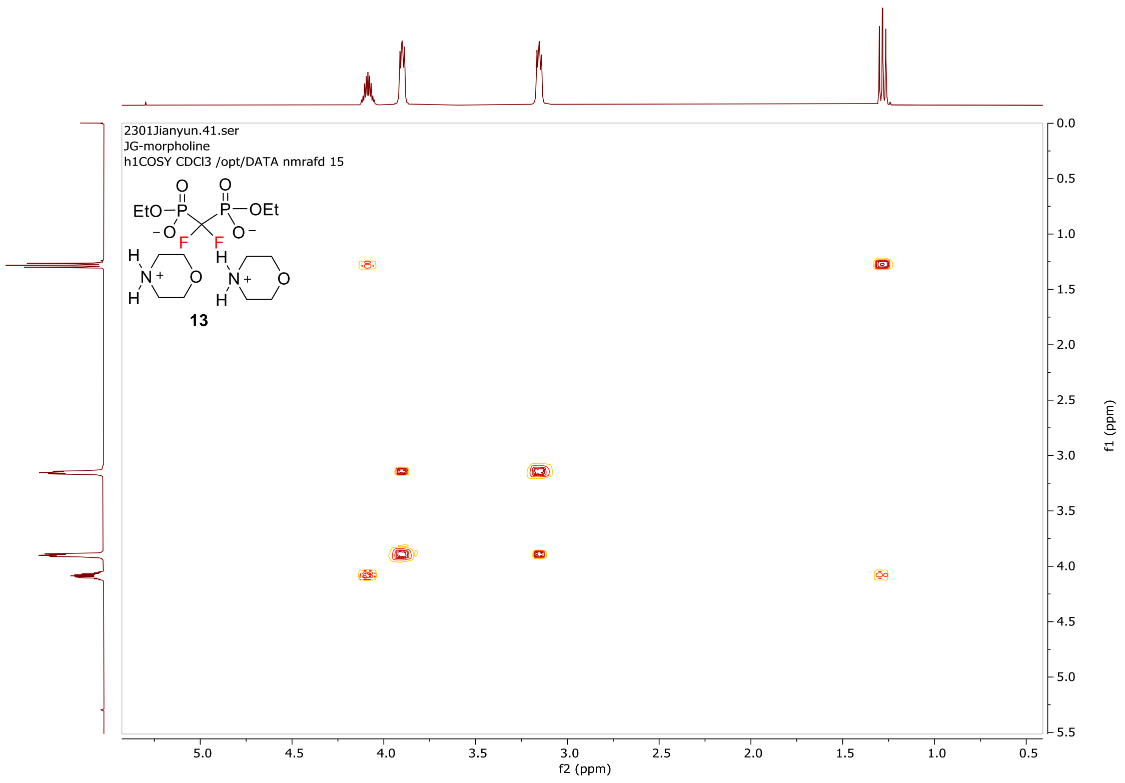

^{1}H-^{1}H COSY (101 MHz, $CDCl_3$) of compound **13**

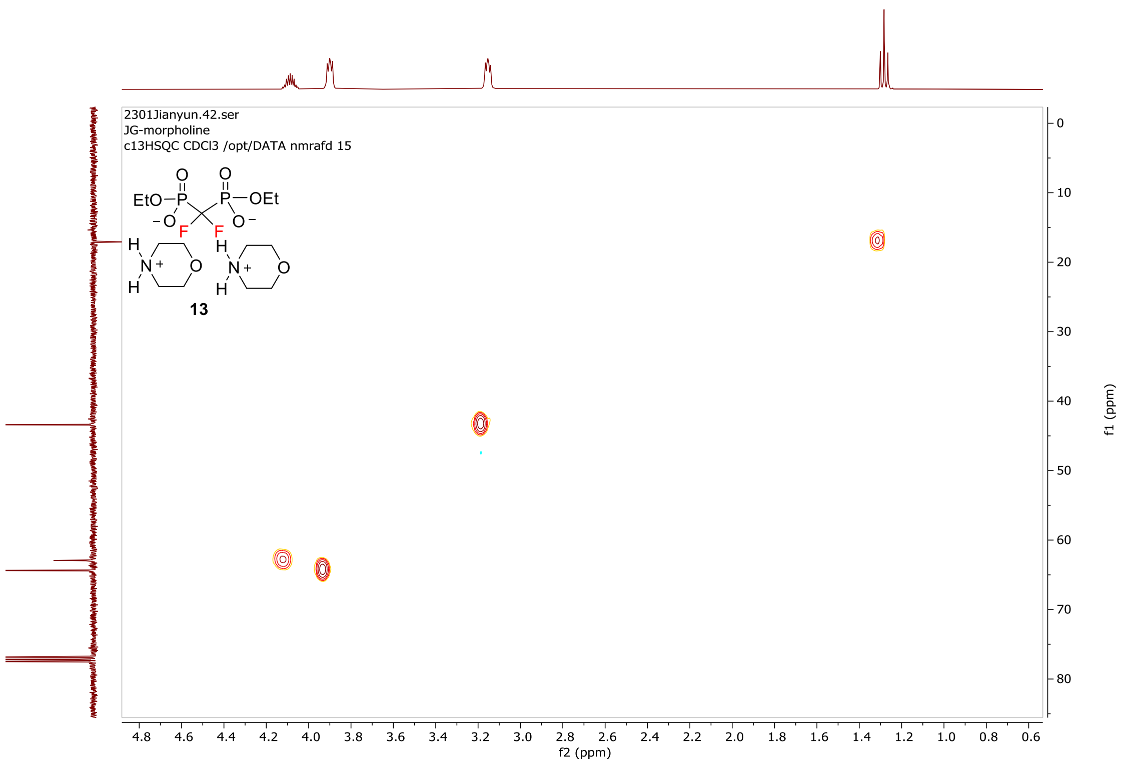

^{1}H-^{13}C HSQC (400 MHz, $CDCl_3$) of compound **13**

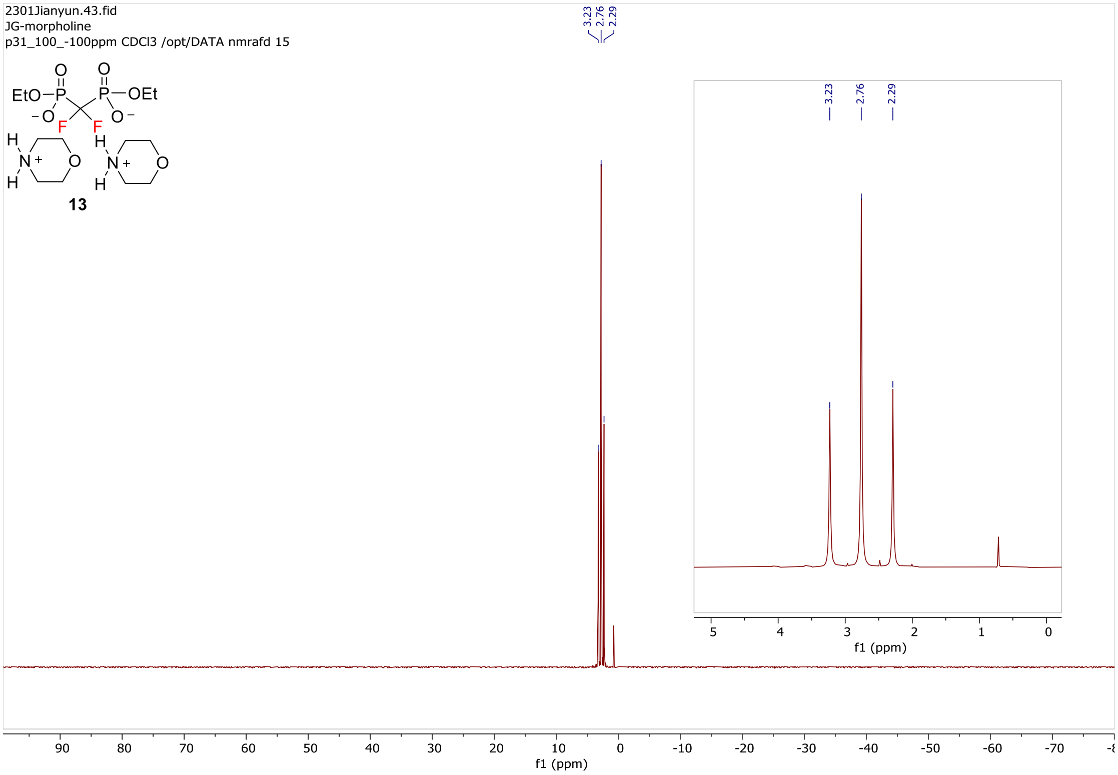

^{31}P NMR (162 MHz, $CDCl_3$) of compound **13**

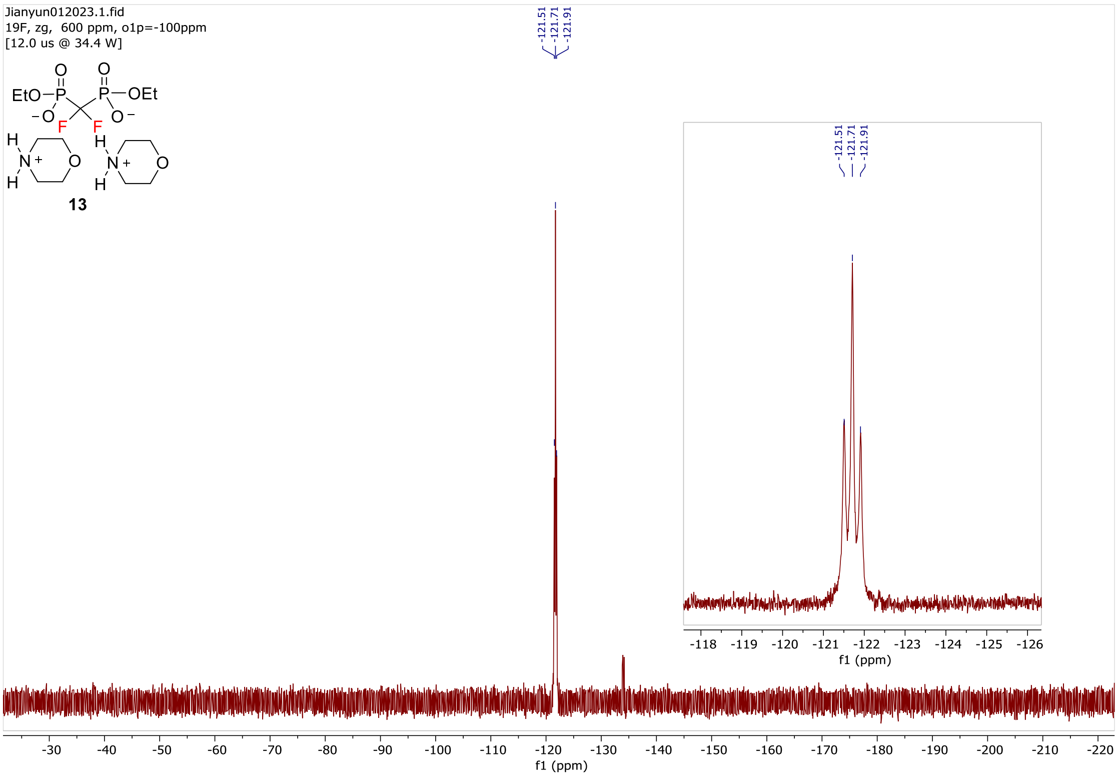

^{19}F NMR (376 MHz, $CDCl_3$) of compound **13**

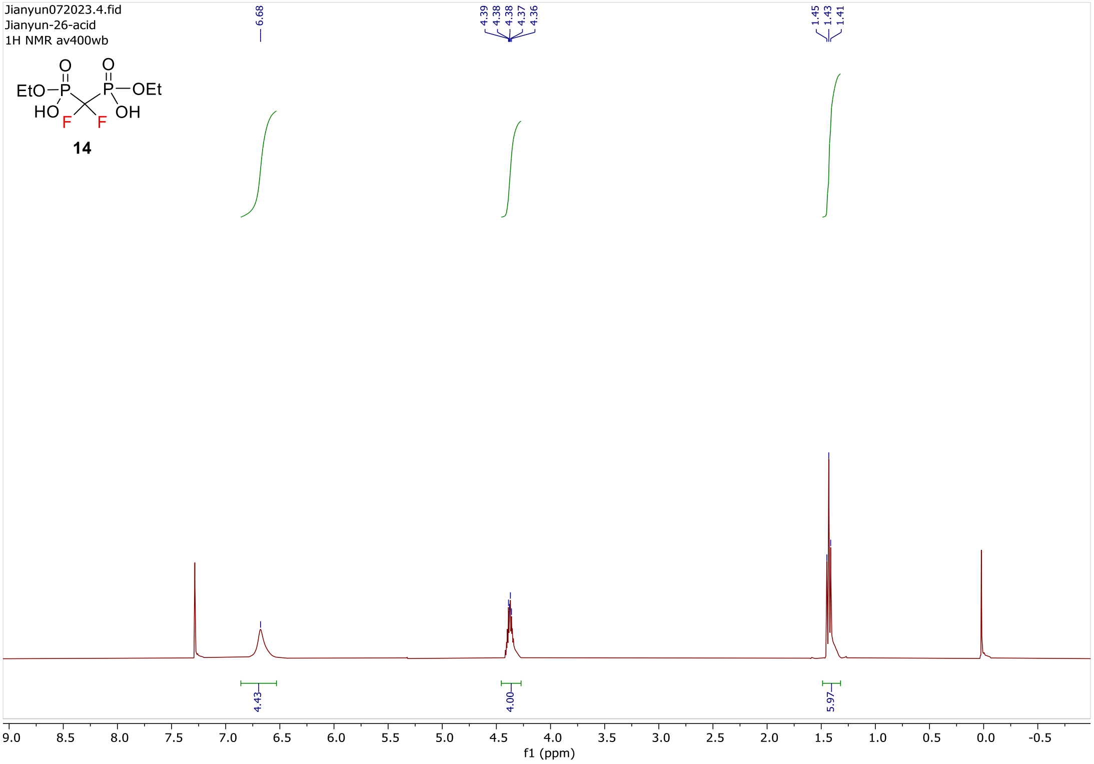

^{1}H NMR (400 MHz, $CDCl_3$) of compound **14**

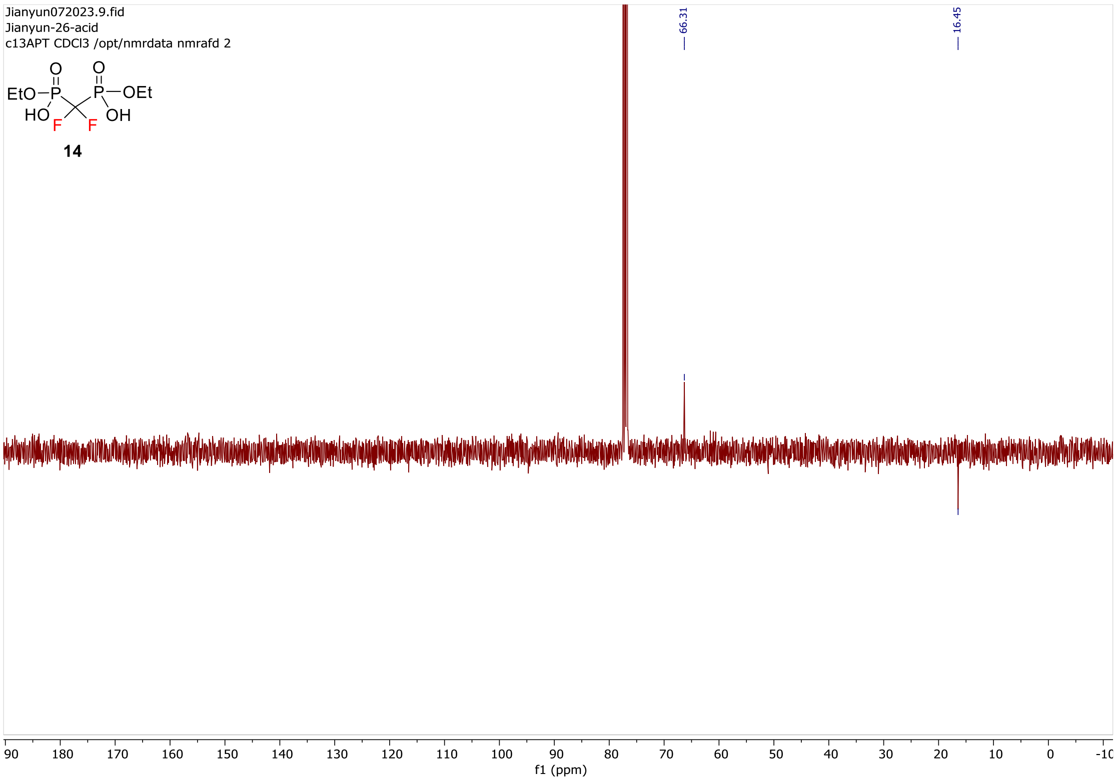

^{13}C NMR (101 MHz, $CDCl_3$) of compound **14**

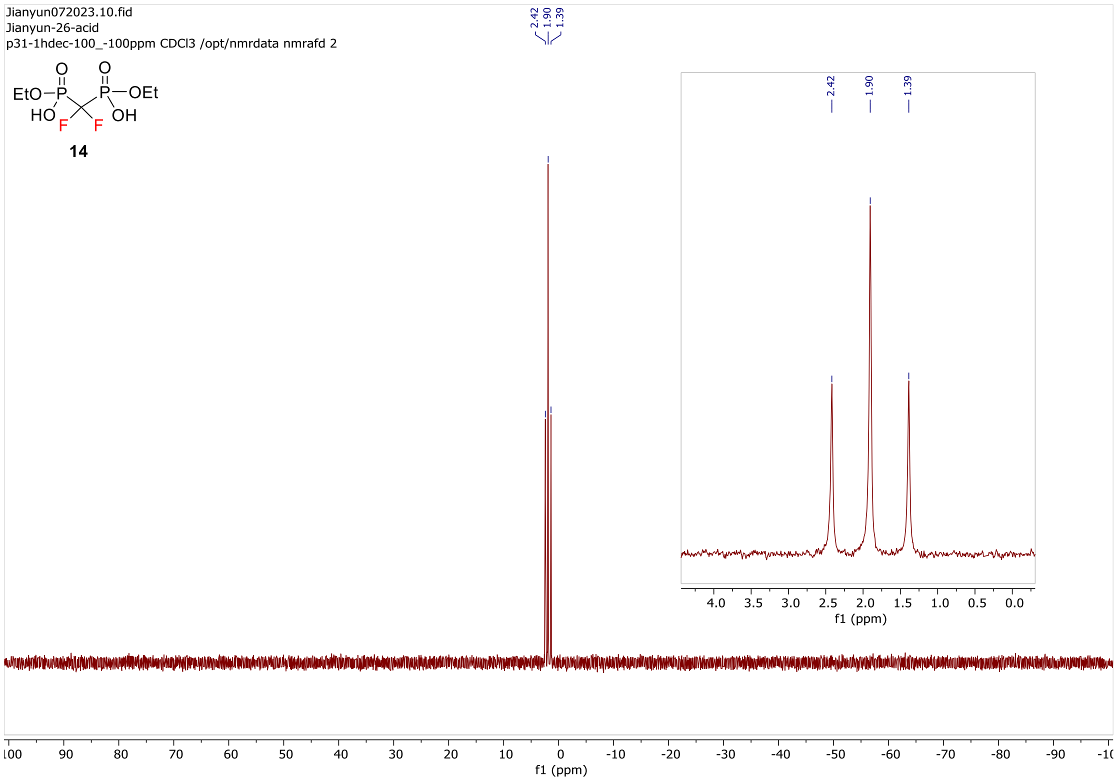

^{31}P NMR (162 MHz, $CDCl_3$) of compound **14**

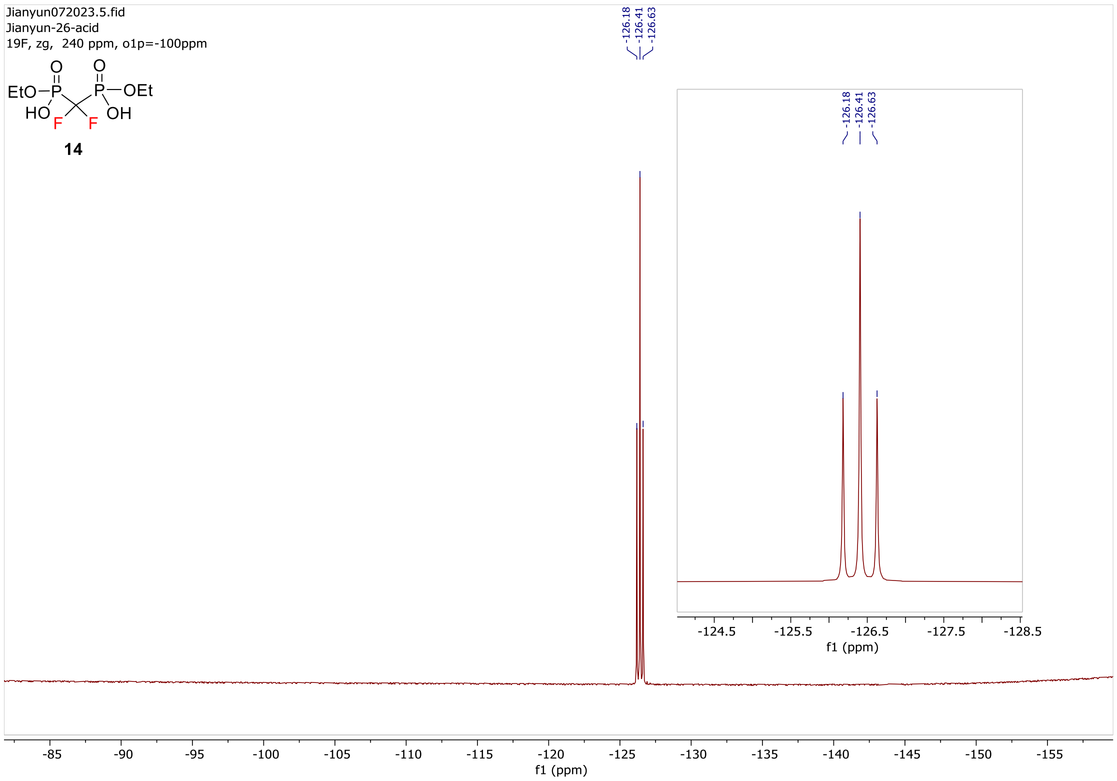

^{19}F NMR (376 MHz, $CDCl_3$) of compound **14**

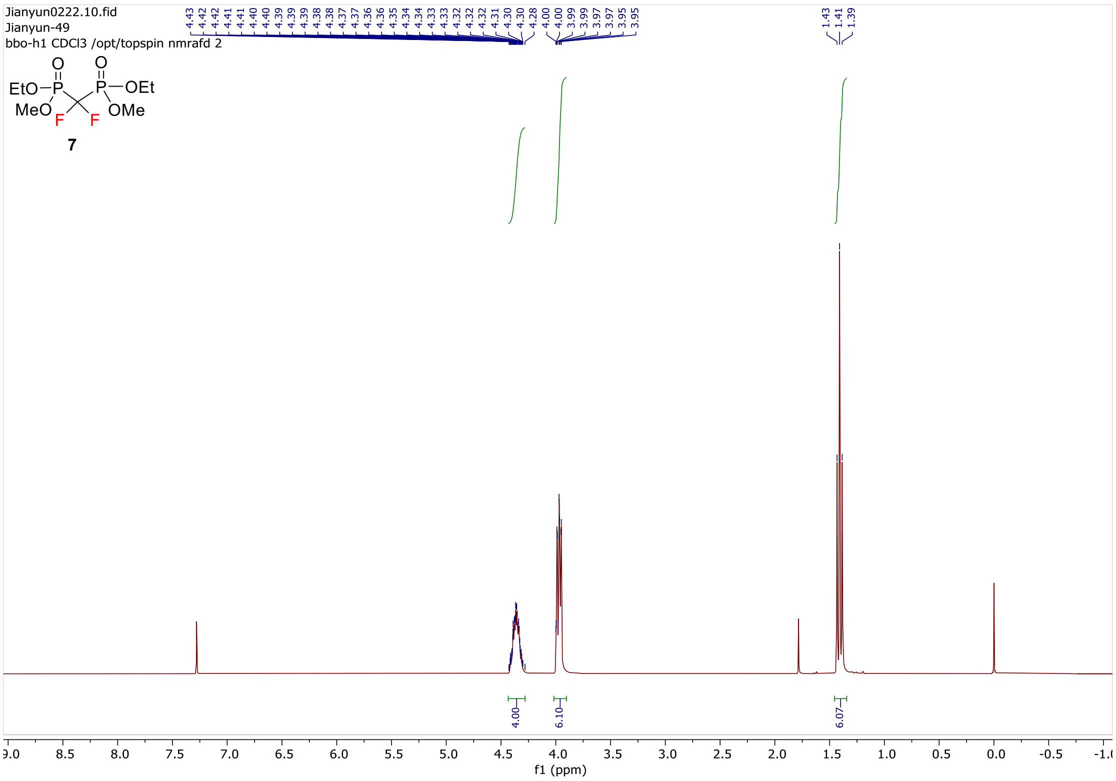

^{1}H NMR (400 MHz, $CDCl_3$) of compound **7**

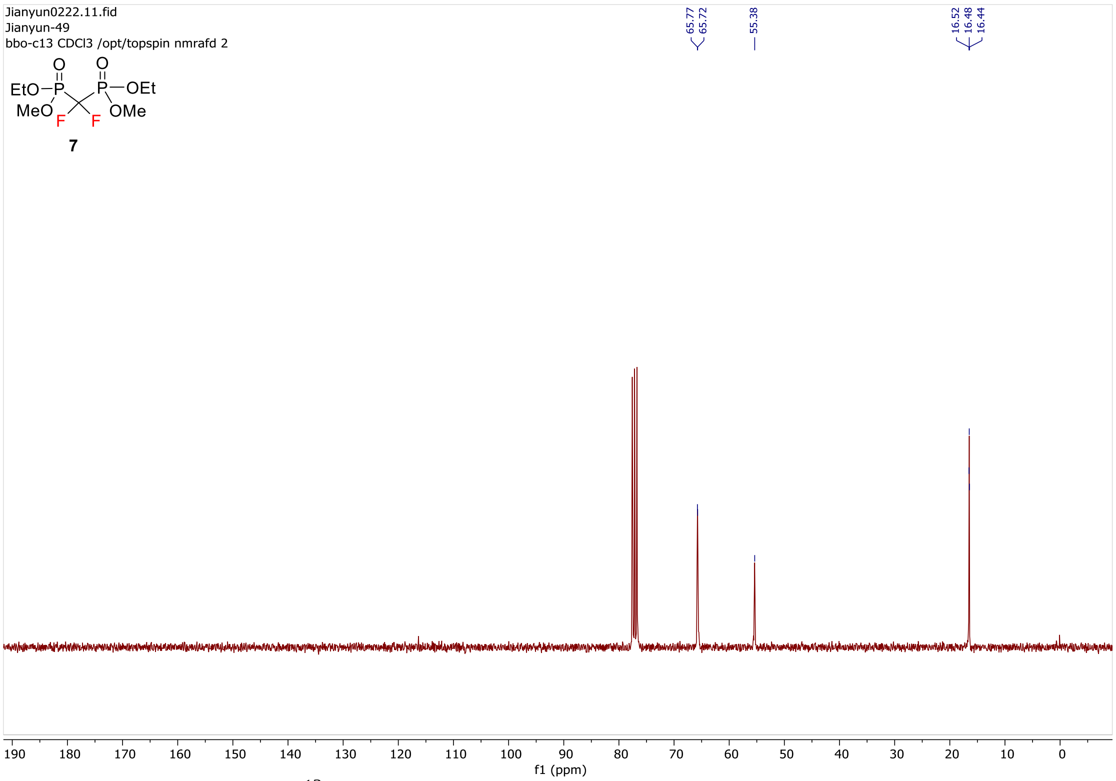

^{13}C NMR (101 MHz, $CDCl_3$) of compound **7**

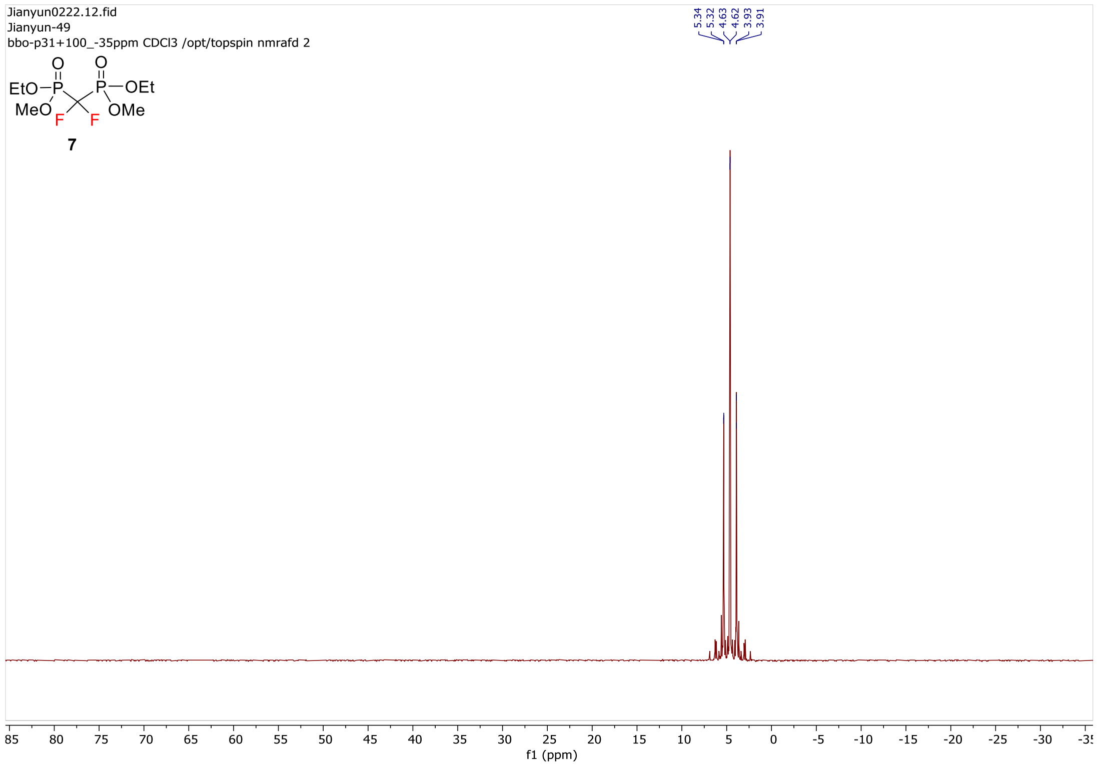

^{31}P NMR (162 MHz, CDCl$_3$) of compound **7**

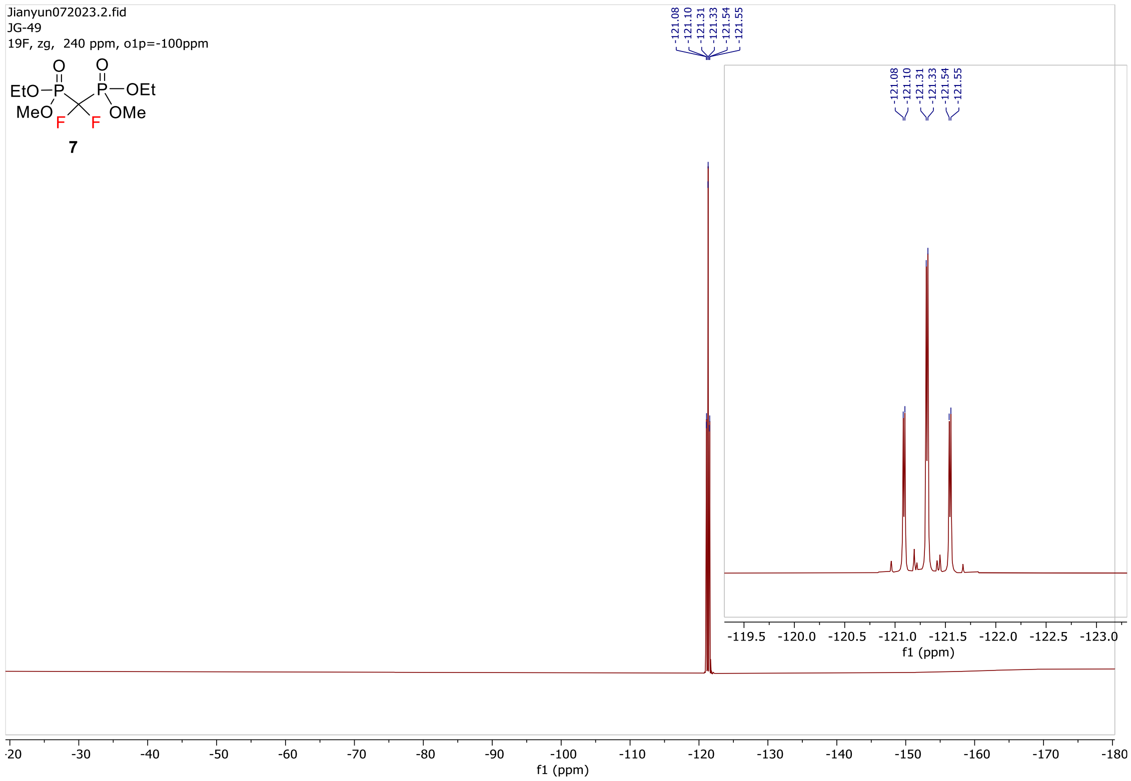

^{19}F NMR (376 MHz, CDCl$_3$) of compound **7**

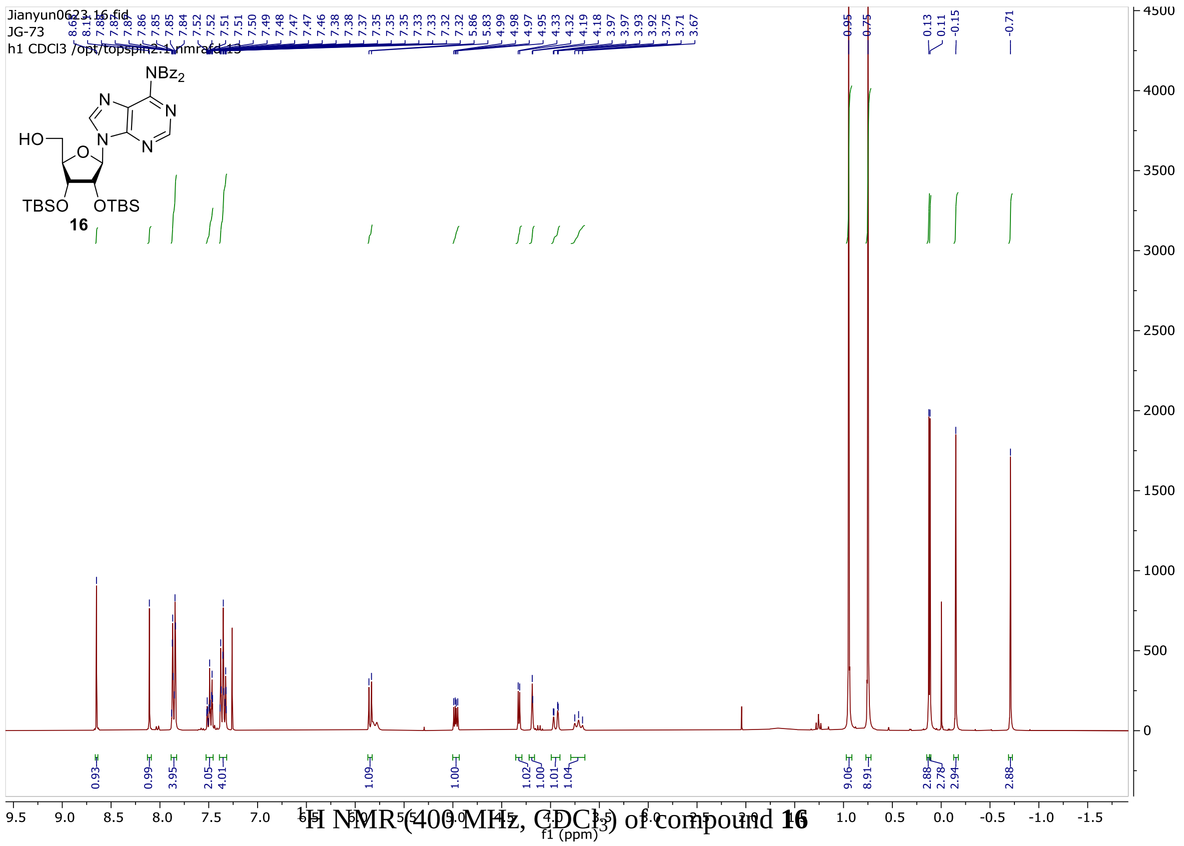

^{1}H NMR (400 MHz, CDCl$_3$) of compound **16**

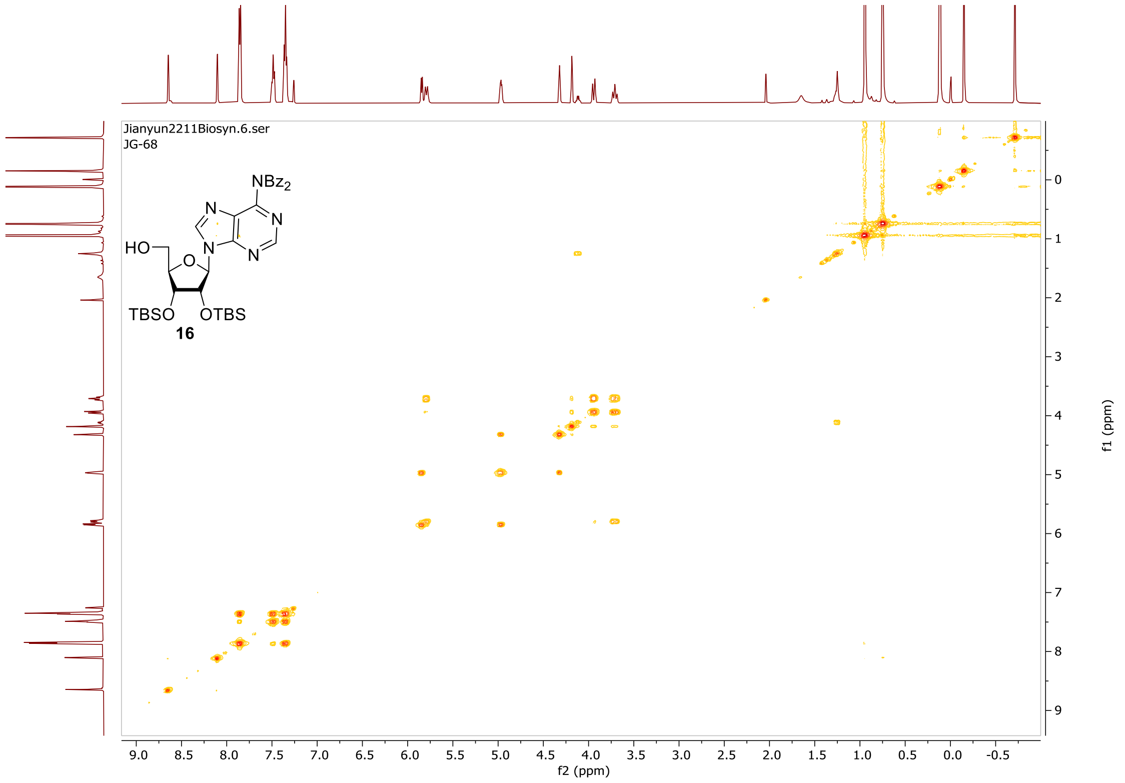

^{1}H - ^{1}H COSY (101 MHz, CDCl$_3$) of compound **16**

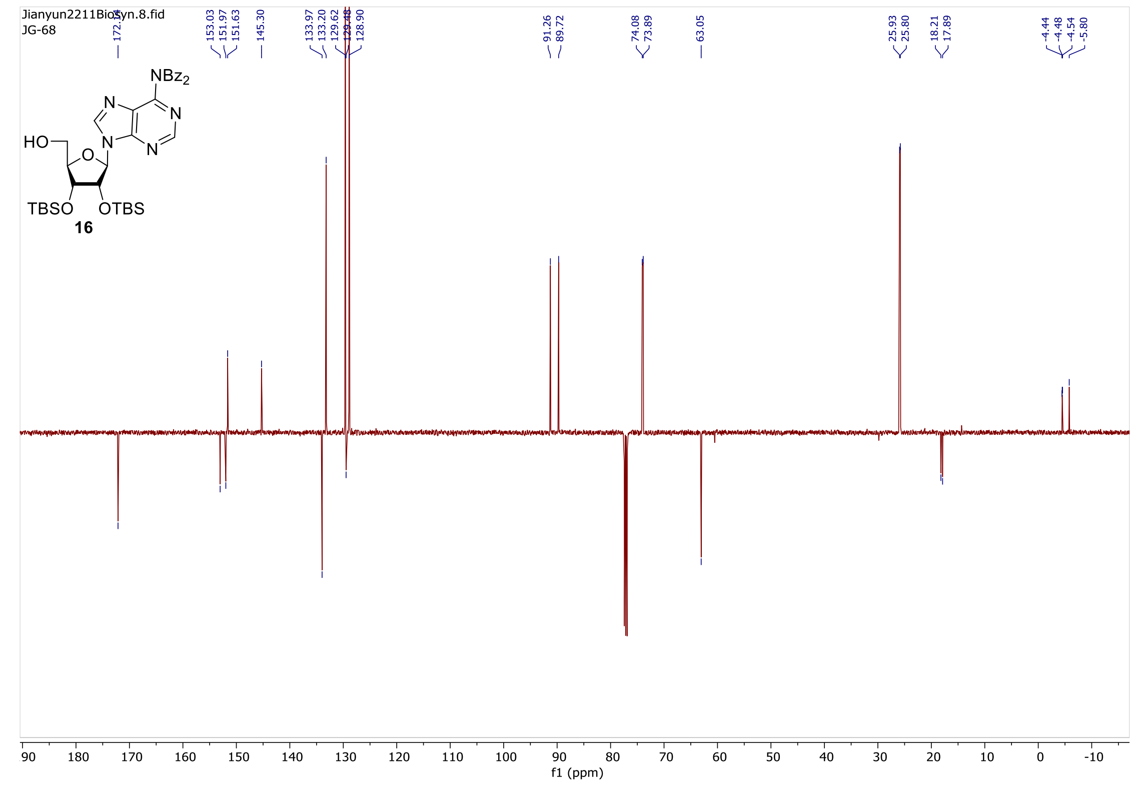

^{13}C NMR (101 MHz, CDCl$_3$) of compound **16**

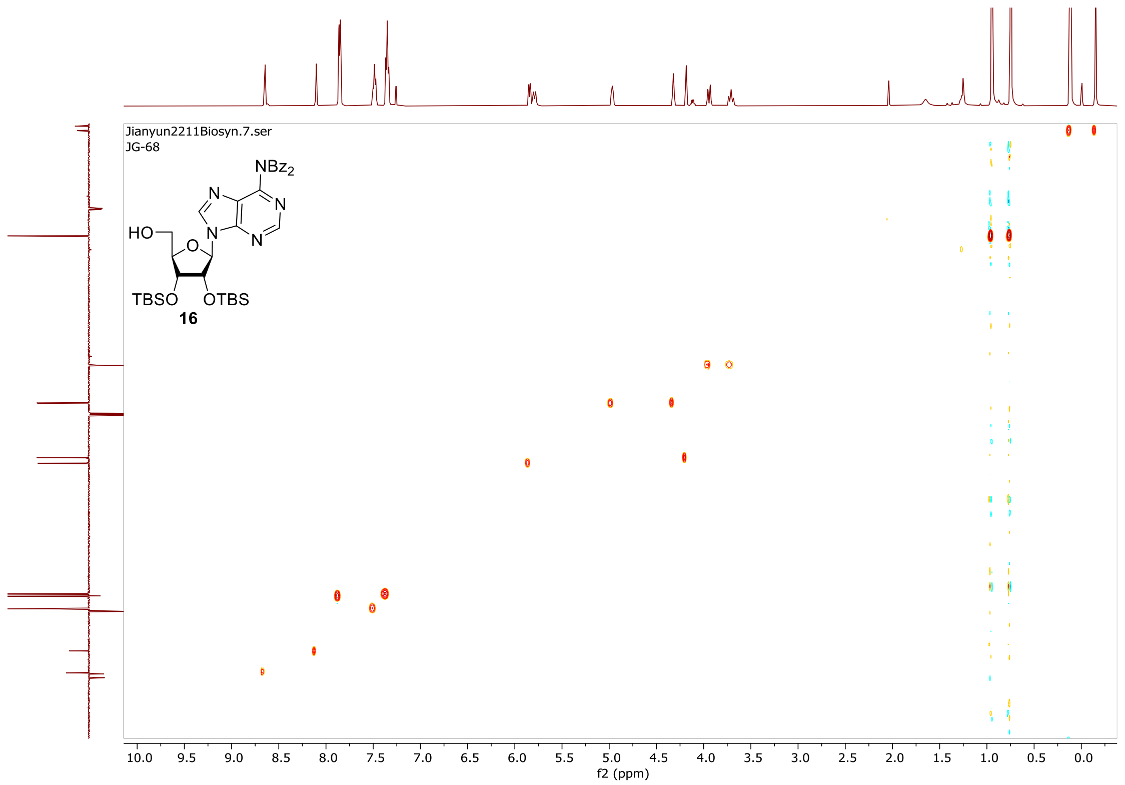

^{1}H - ^{13}C HSQC (400 MHz, CDCl$_3$) of compound **16**

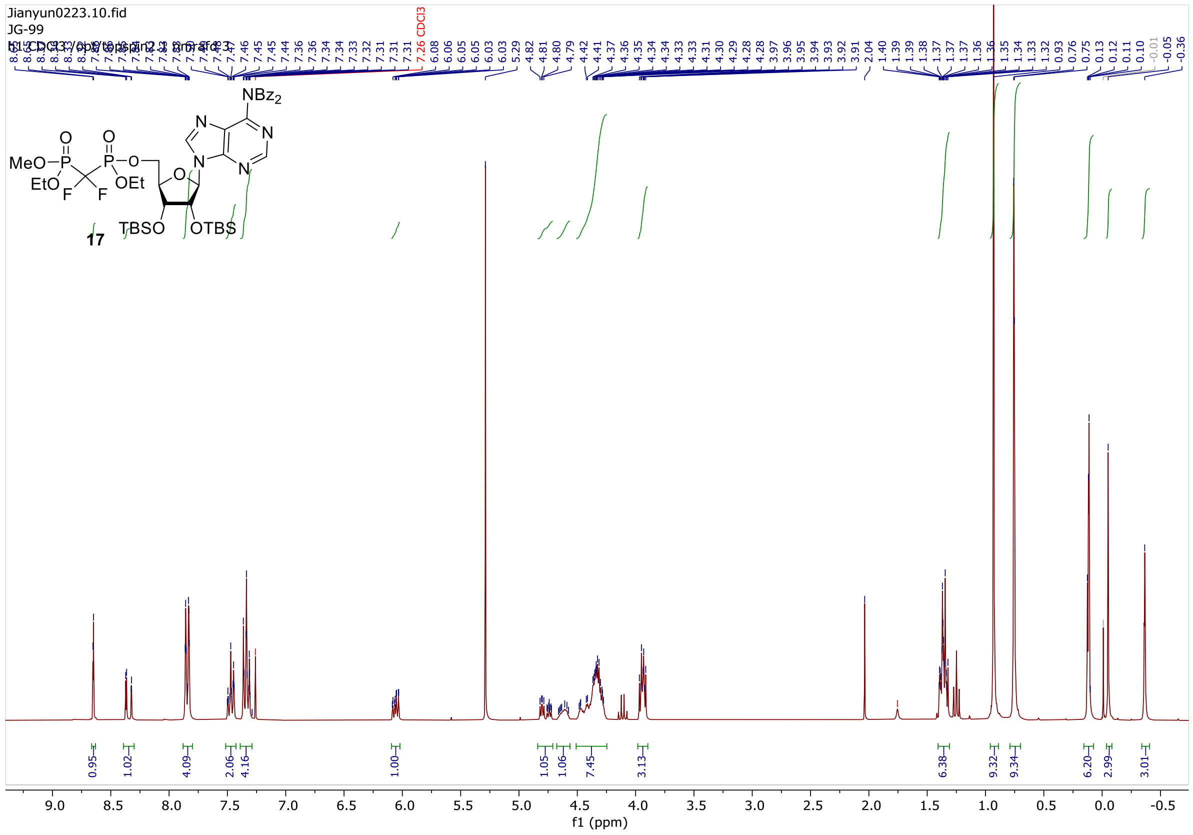

^{1}H NMR (400 MHz, CDCl$_3$) of compound **17**

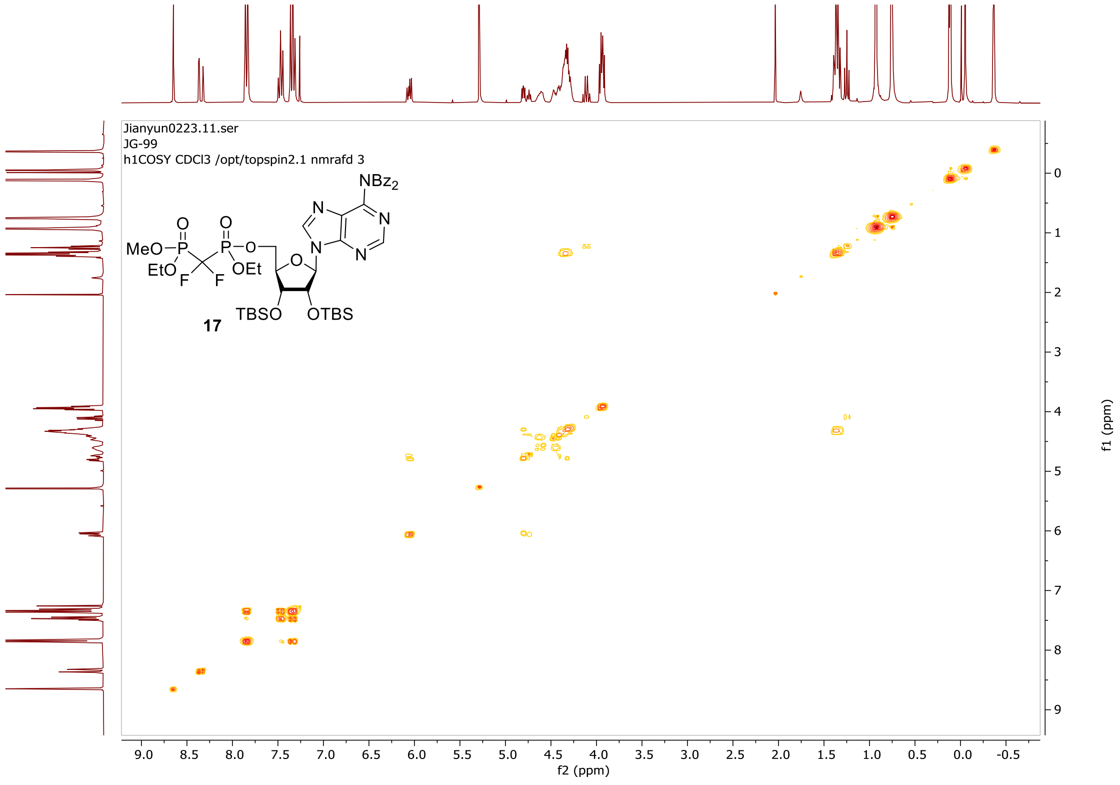

^{1}H - ^{1}H COSY (400 MHz, CDCl$_3$) of compound **17**

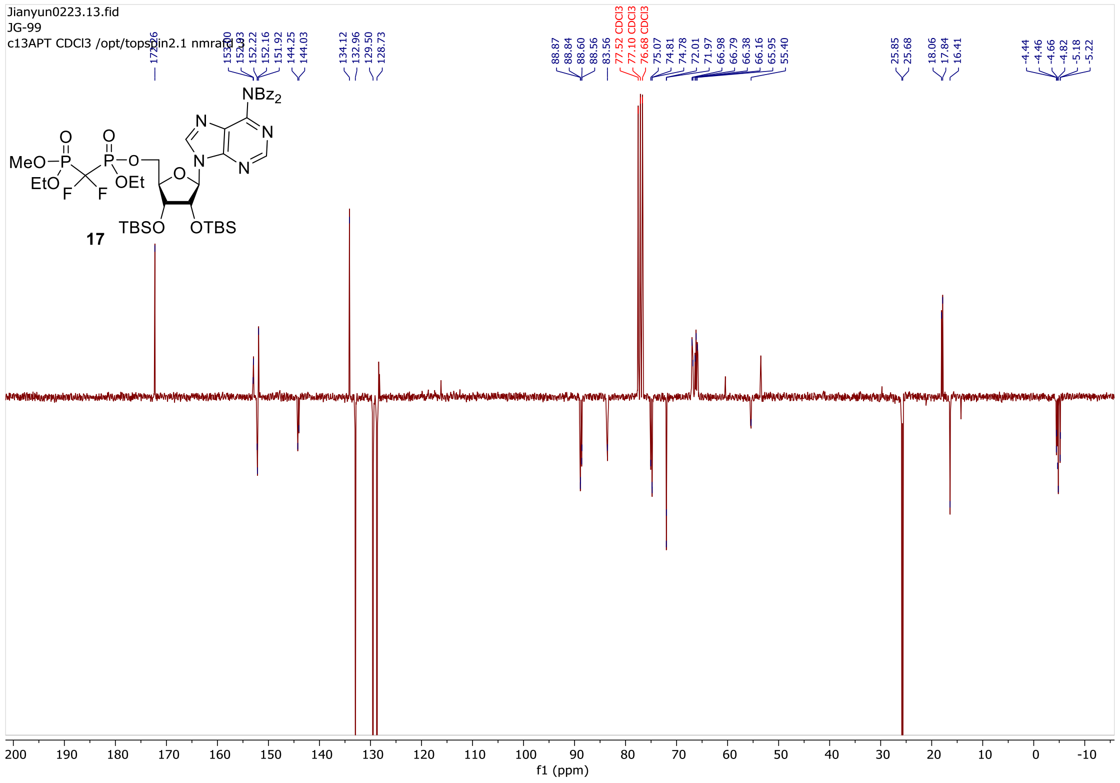

^{13}C NMR (101 MHz, $CDCl_3$) of compound **17**

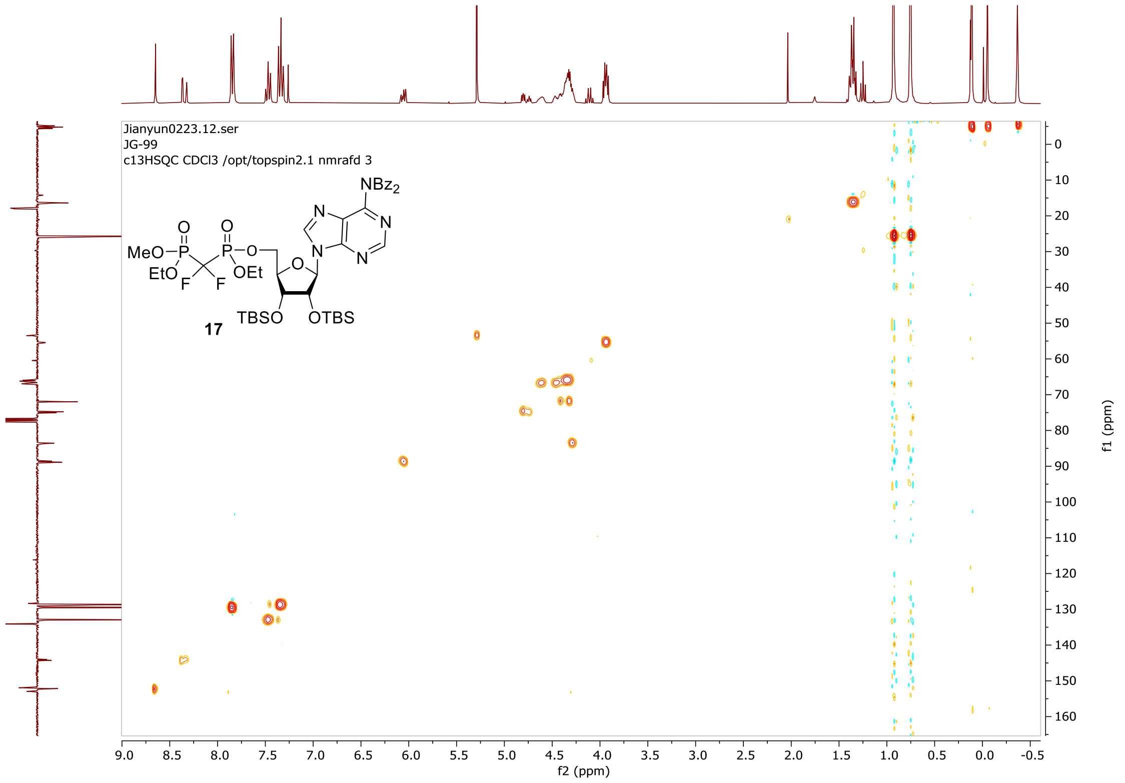

^{1}H - ^{13}C HSQC (400 MHz, $CDCl_3$) of compound **17**

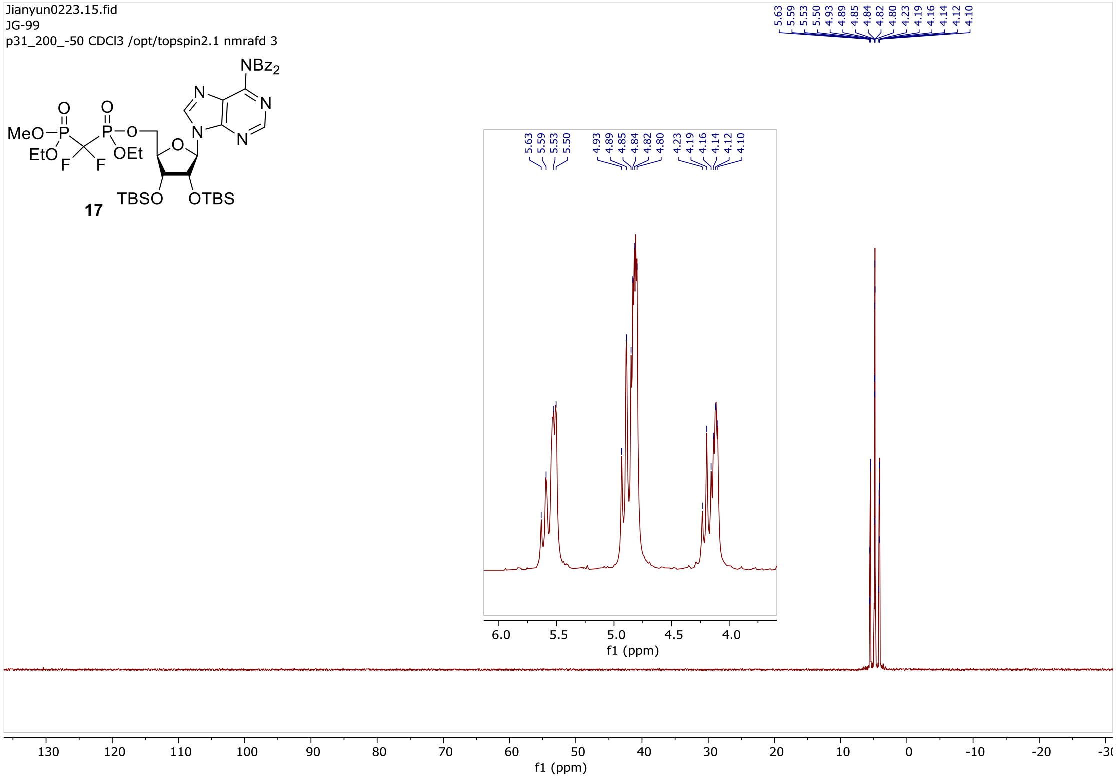

^{31}P NMR (162 MHz, CDCl$_3$) of compound **17**

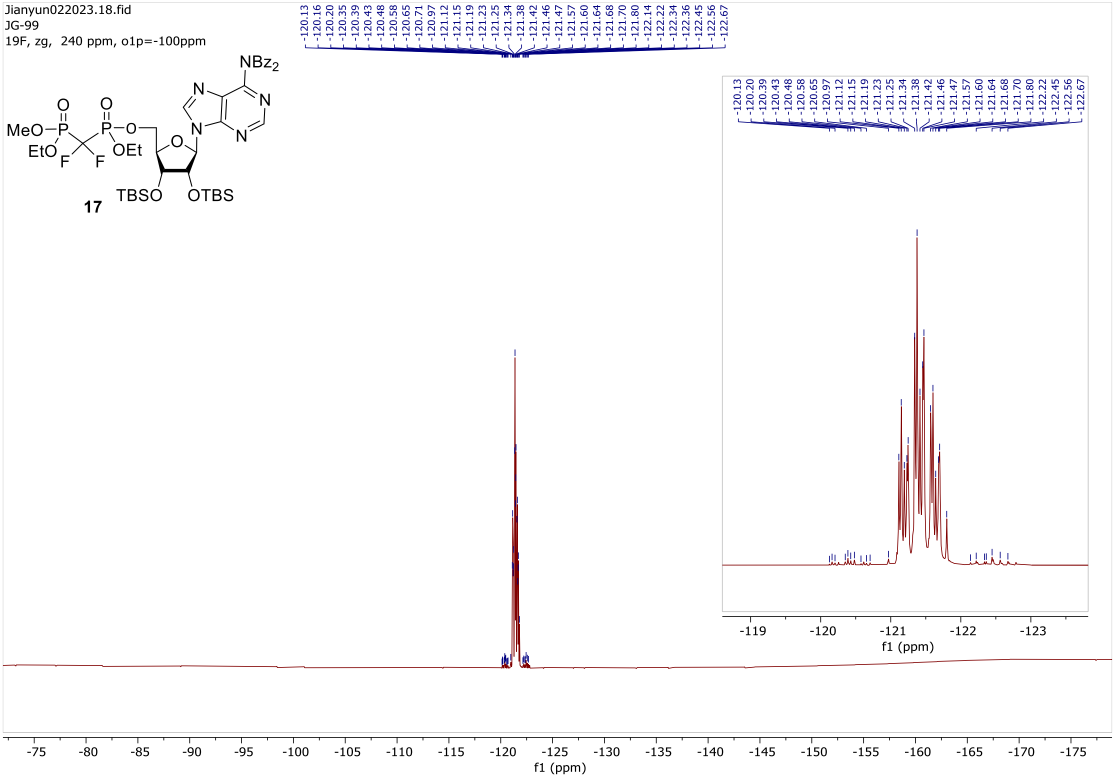

^{19}F NMR (376 MHz, CDCl$_3$) of compound **17**

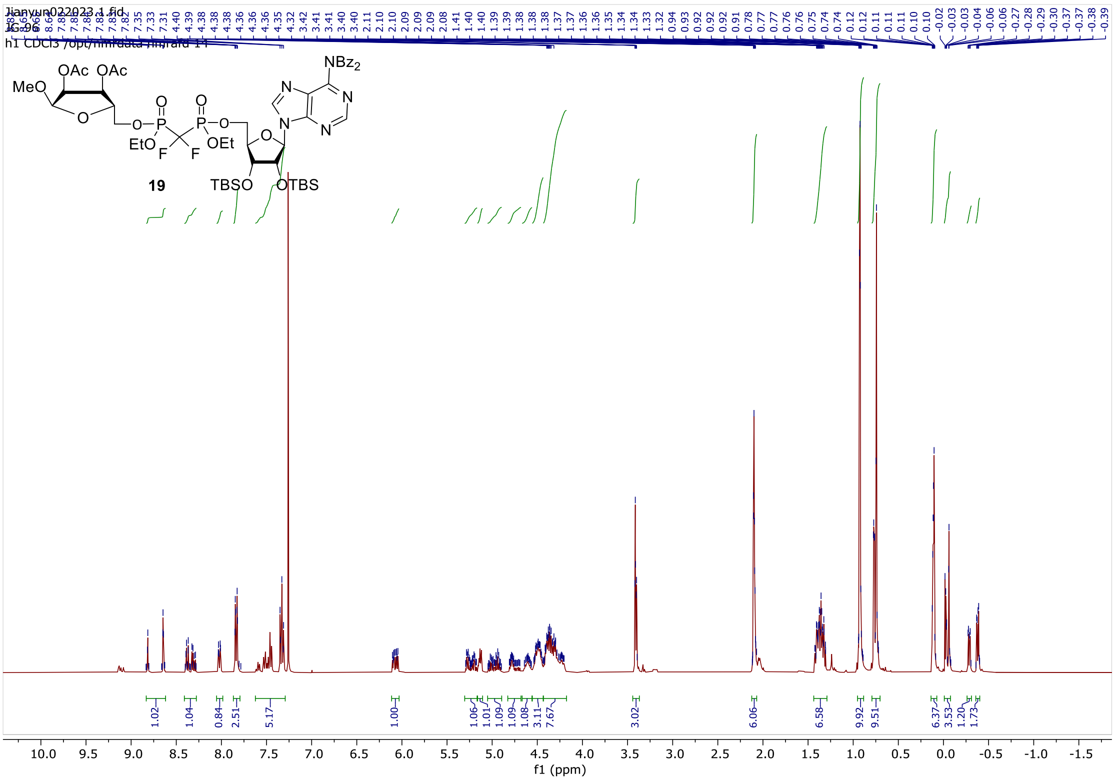

^{1}H NMR (400 MHz, $CDCl_3$) of compound **19**

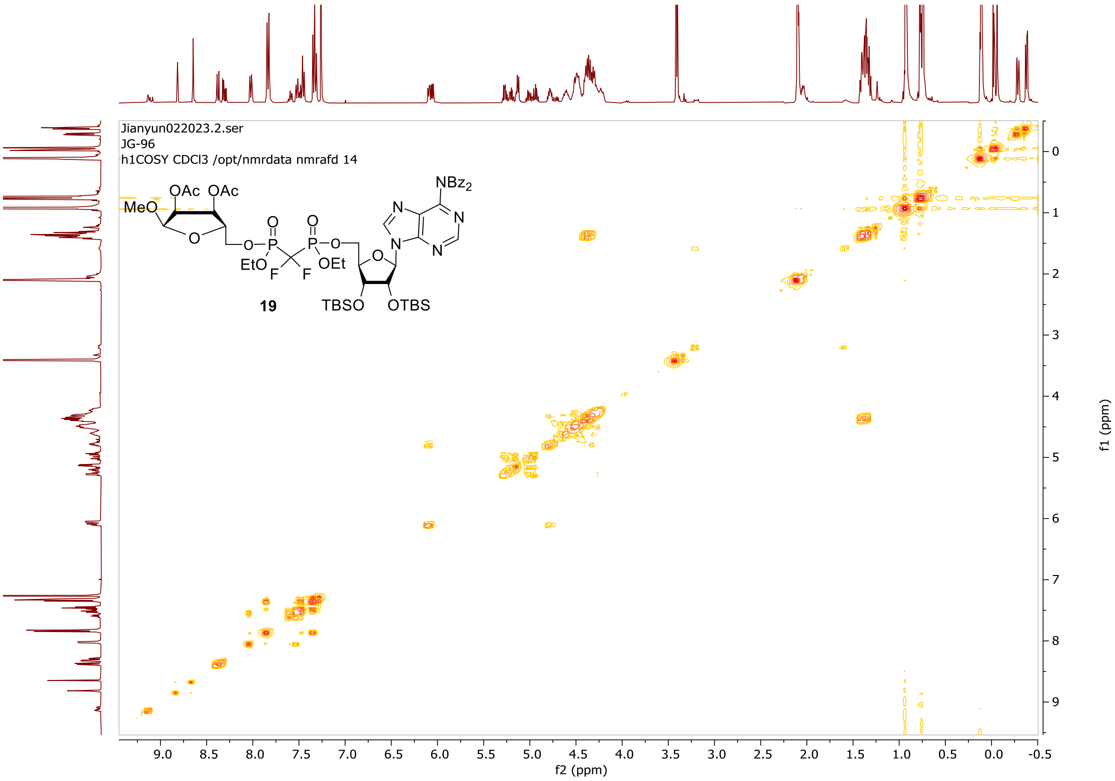

^{1}H-^{1}H COSY (101 MHz, $CDCl_3$) of compound **19**

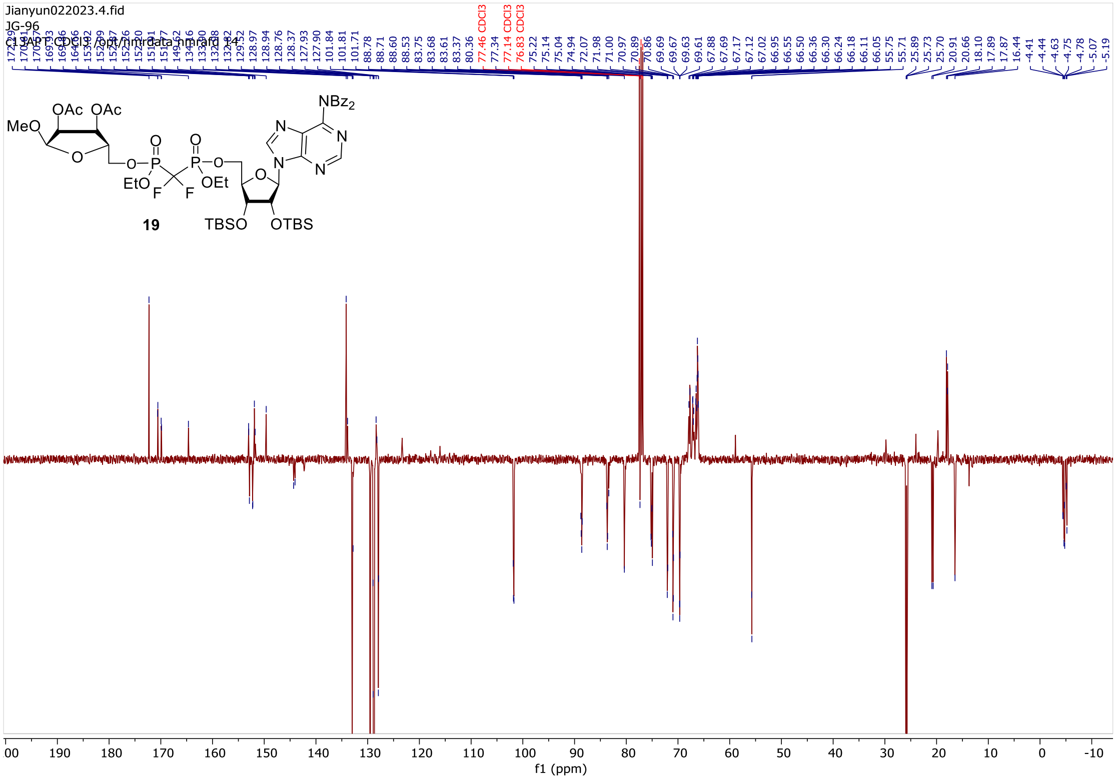

^{13}C NMR (101 MHz, $CDCl_3$) of compound **19**

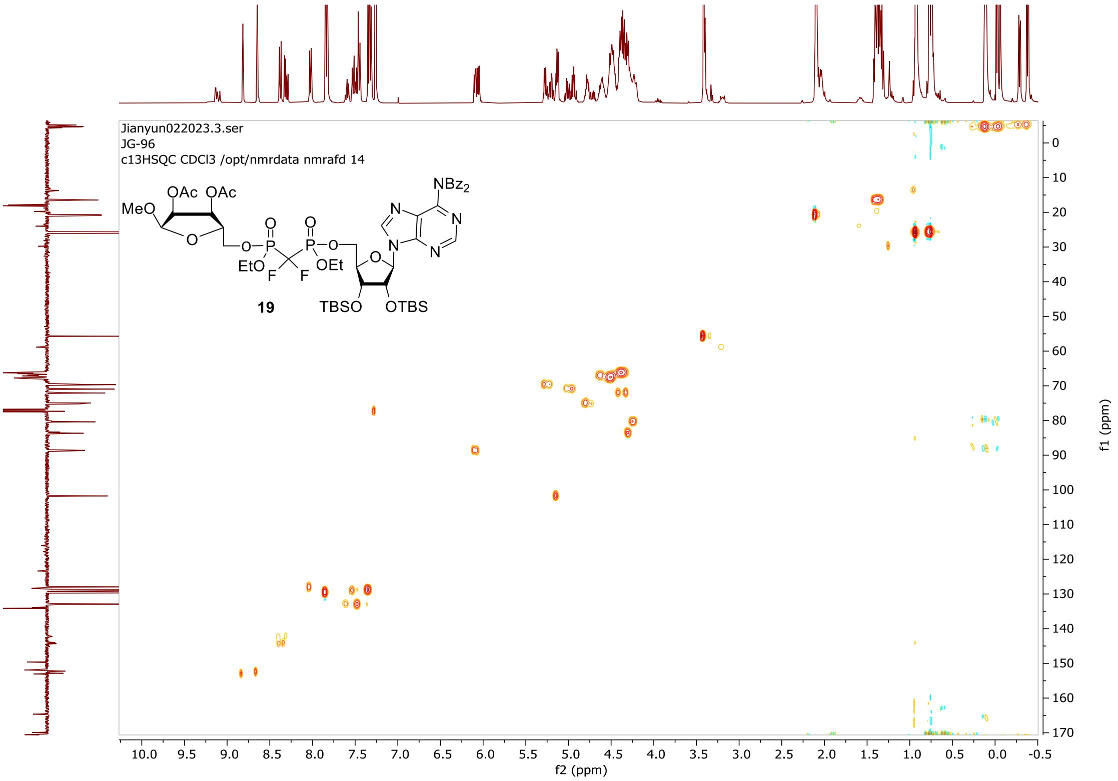

^{1}H - ^{13}C HSQC (400 MHz, $CDCl_3$) of compound **19**

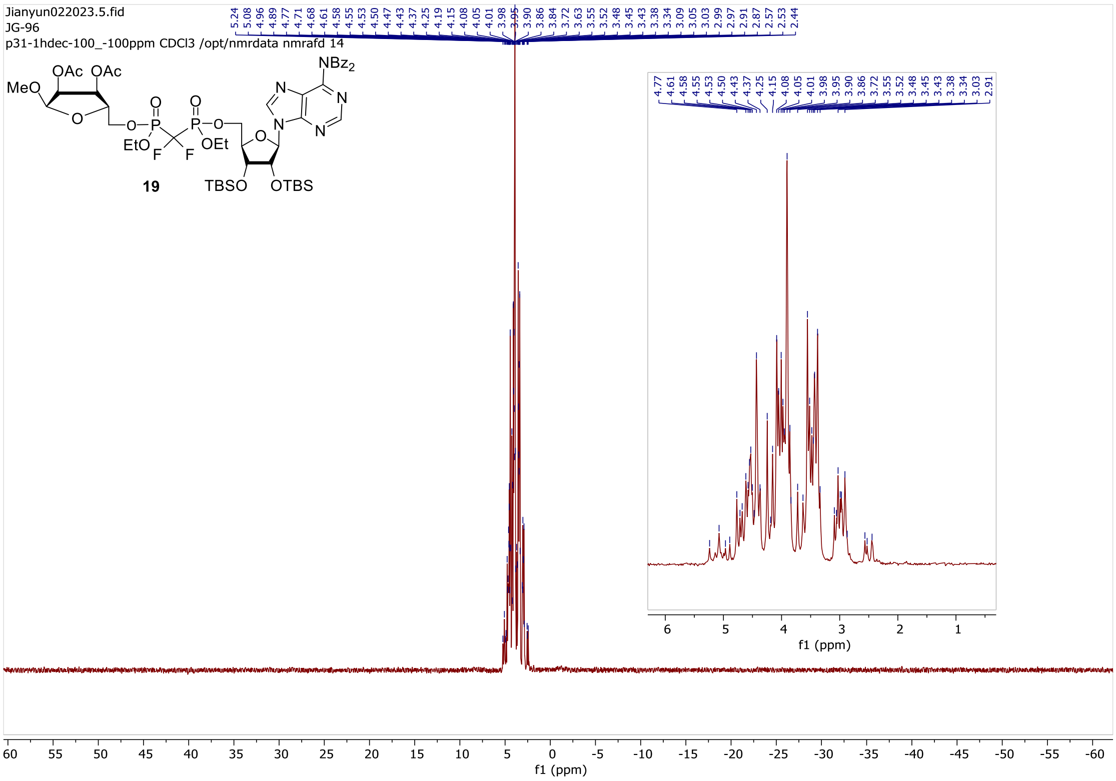

^{31}P NMR (162 MHz, $CDCl_3$) of compound **19**

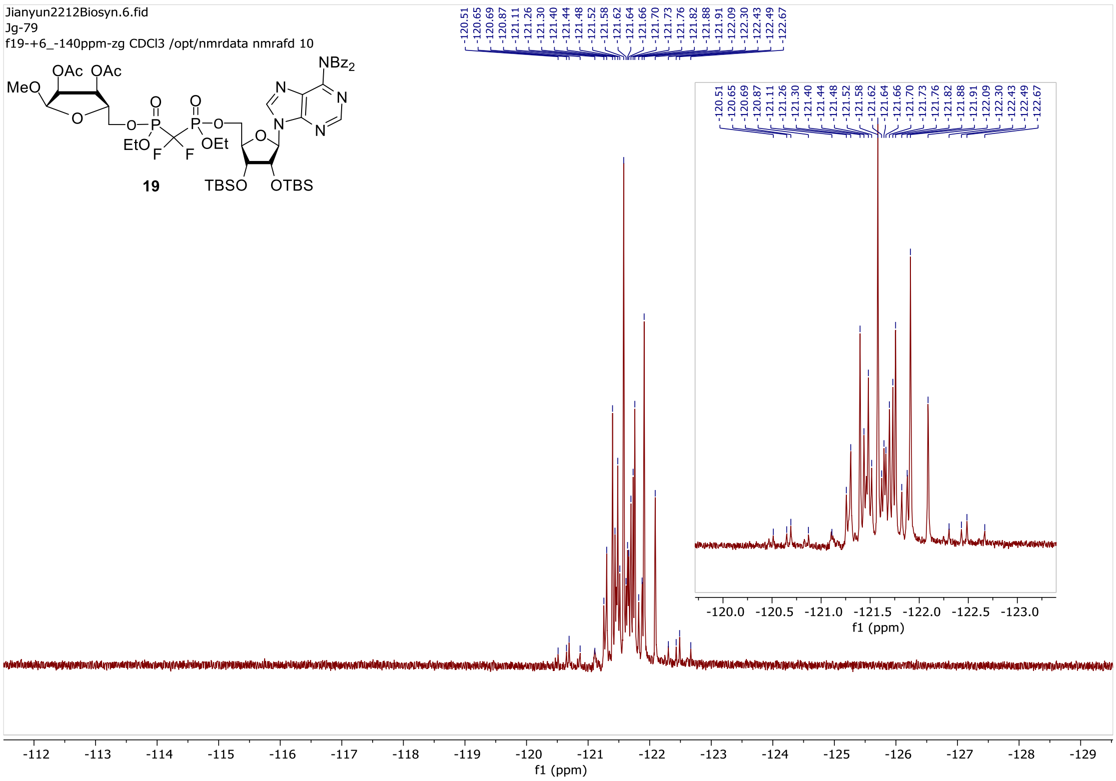

^{19}F NMR (376 MHz, $CDCl_3$) of compound **19**

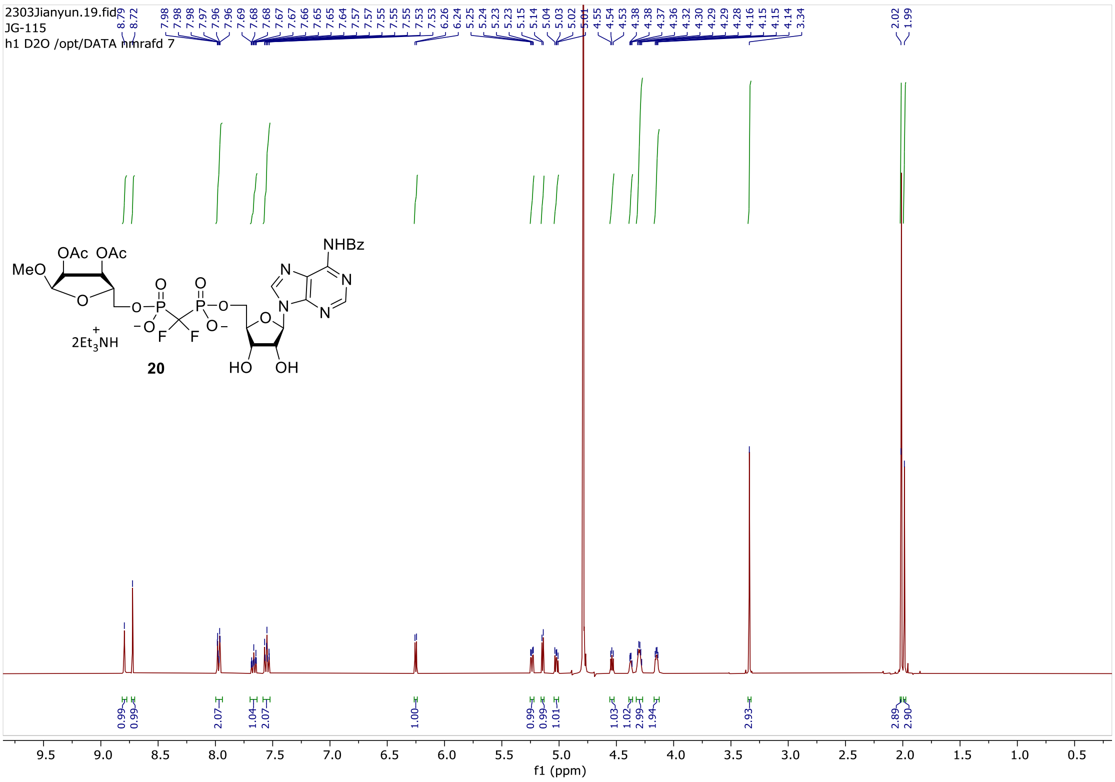

^{1}H NMR (400 MHz, $CDCl_3$) of compound **20**

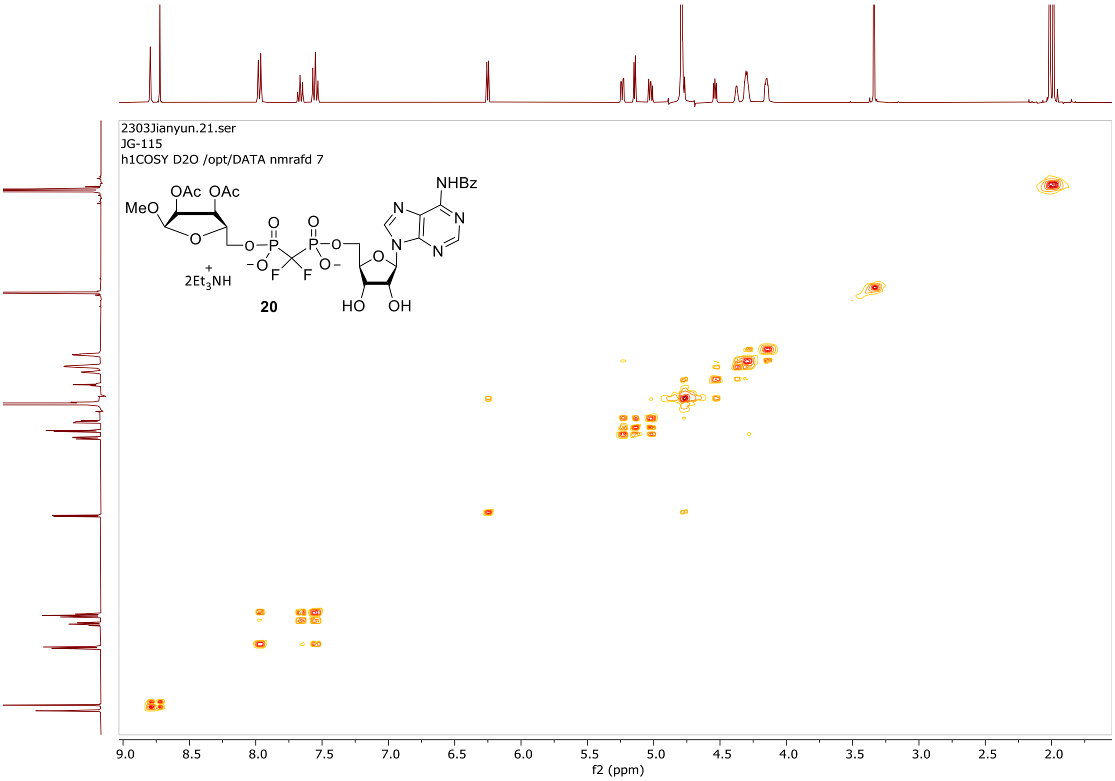

^{1}H - ^{1}H COSY (101 MHz, $CDCl_3$) of compound **20**

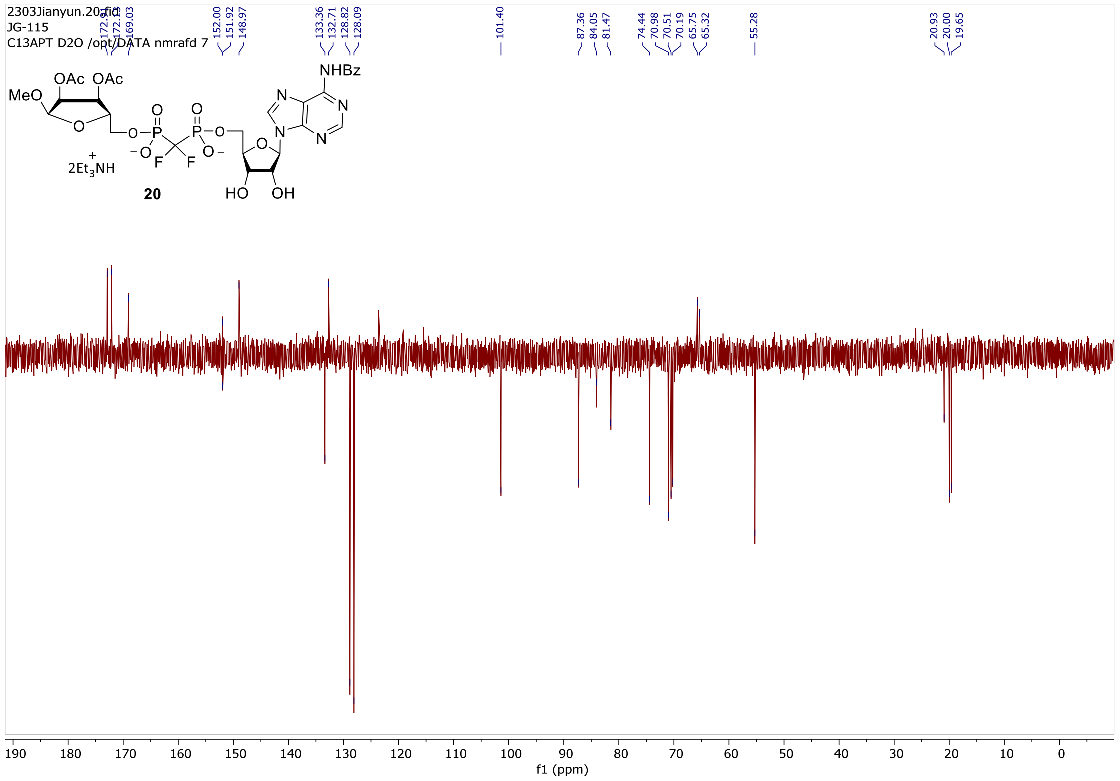

^{13}C NMR (101 MHz, CDCl$_3$) of compound **20**

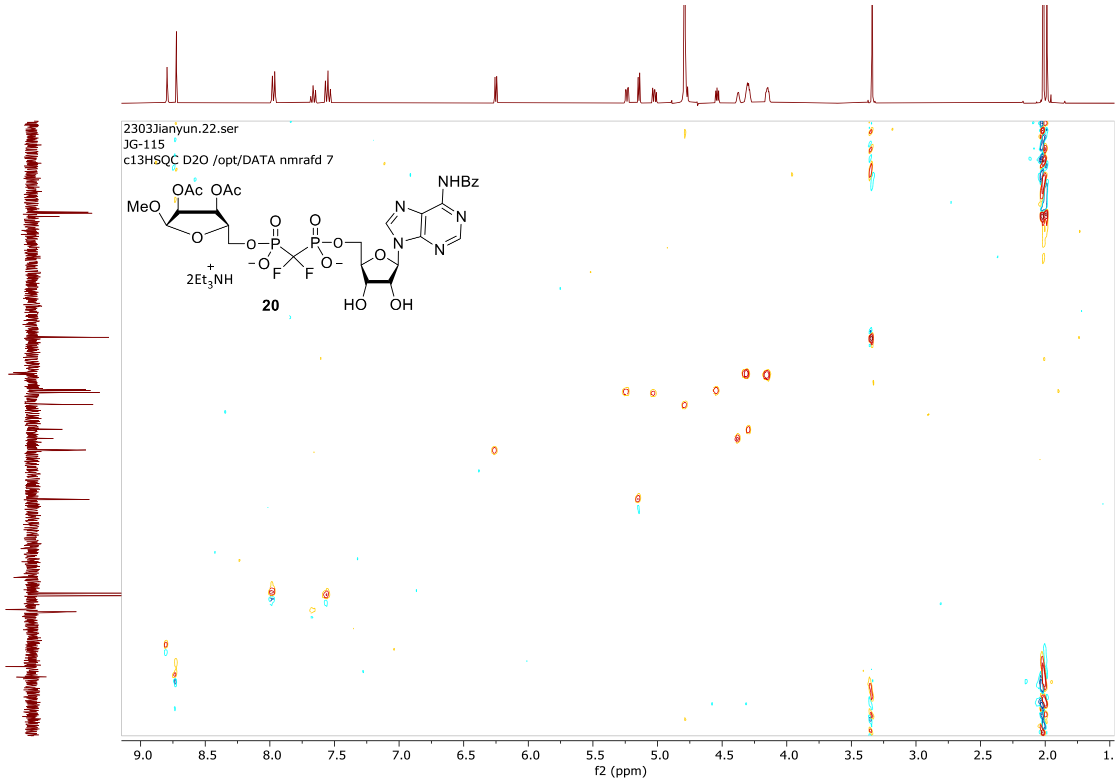

^{1}H - ^{13}C HSQC (400 MHz, CDCl$_3$) of compound **20**

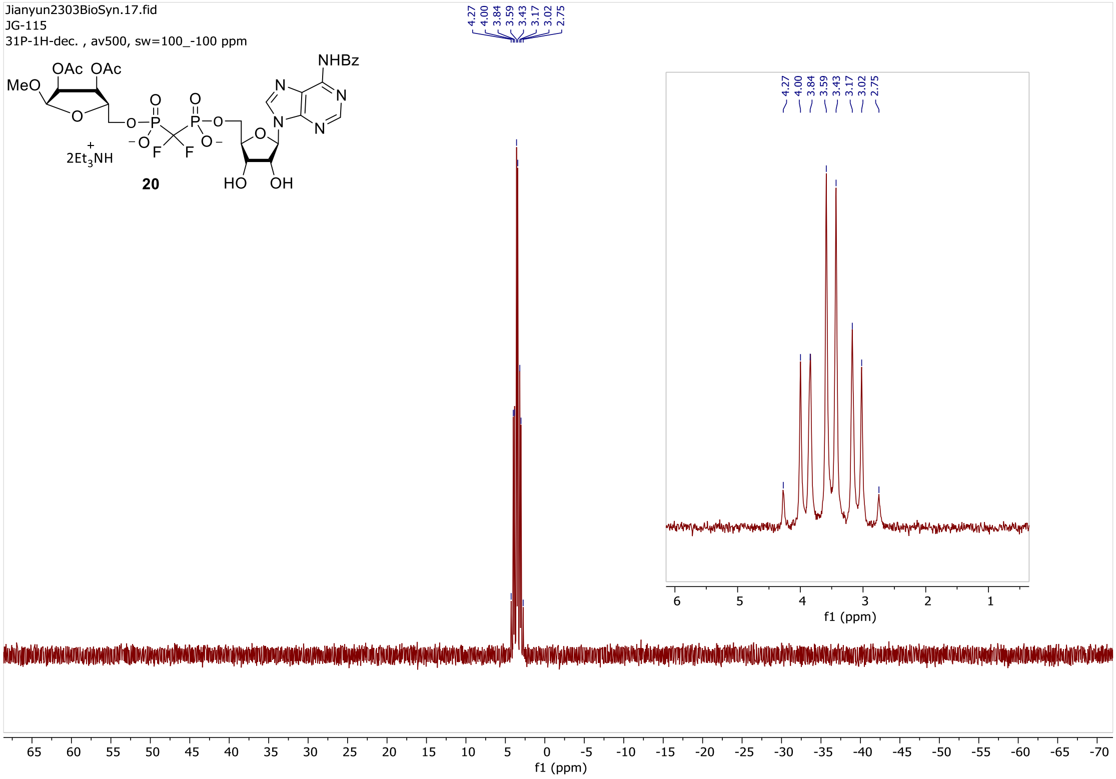

^{31}P NMR (162 MHz, CDCl$_3$) of compound **20**

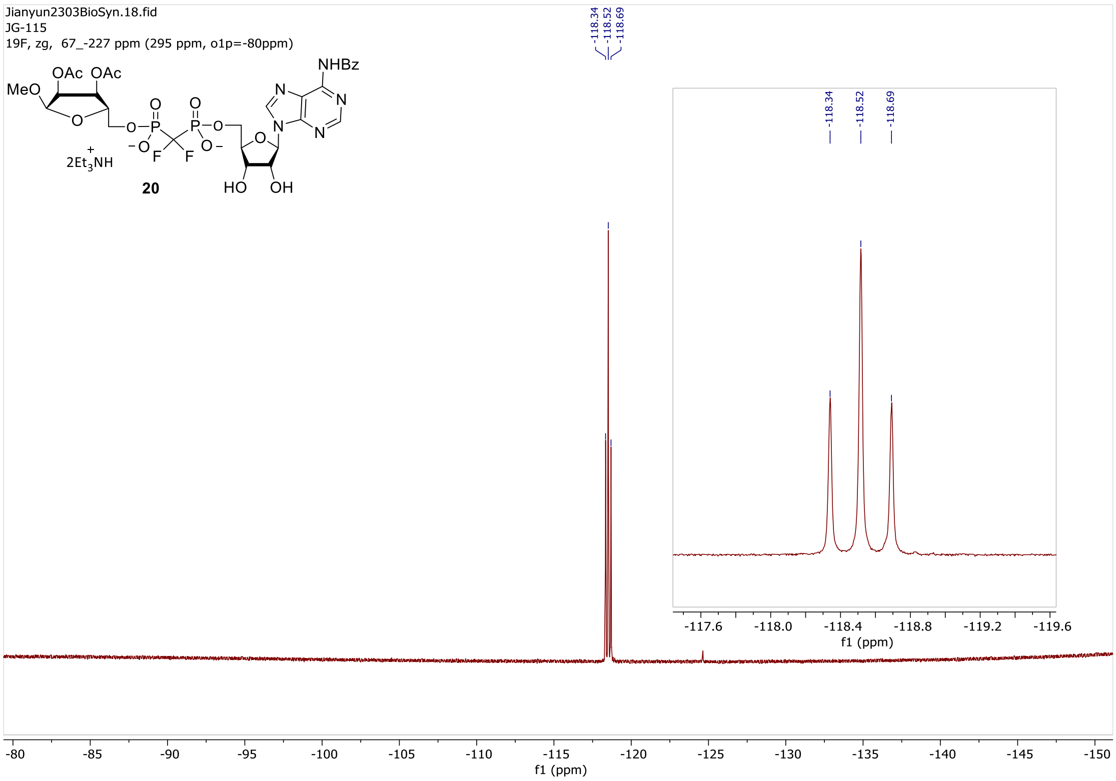

^{19}F NMR (471 MHz, CDCl$_3$) of compound **20**

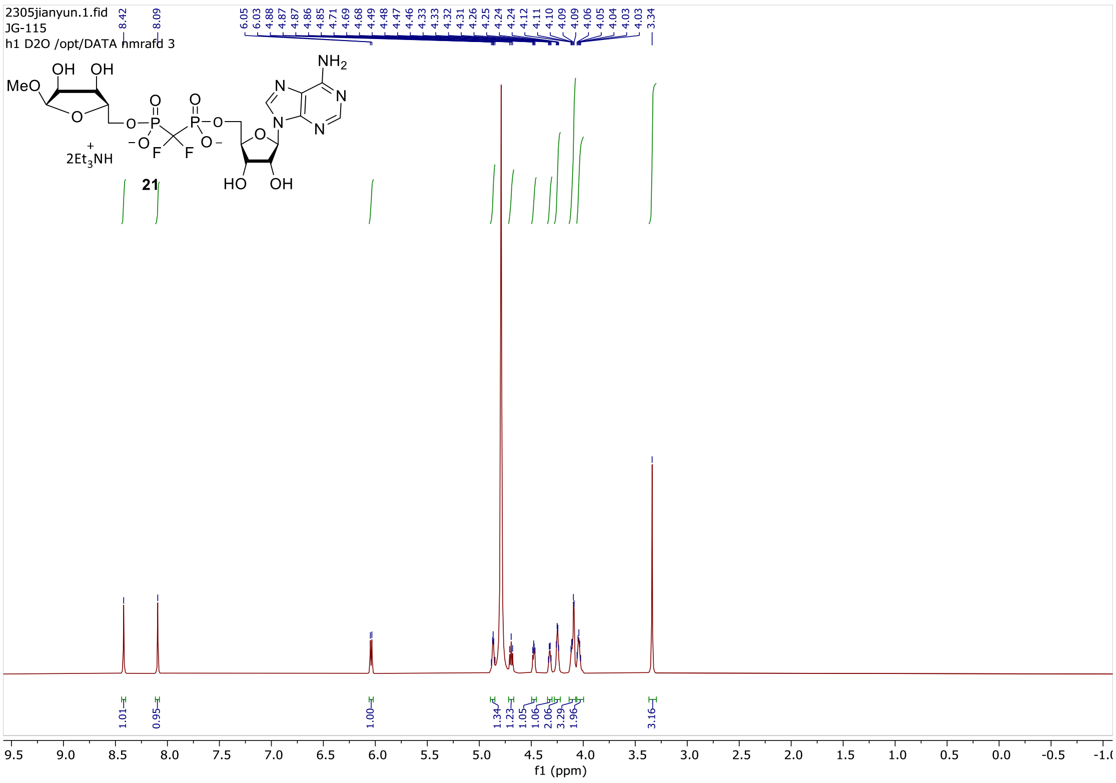

^{1}H NMR (400 MHz, $CDCl_3$) of compound **21**

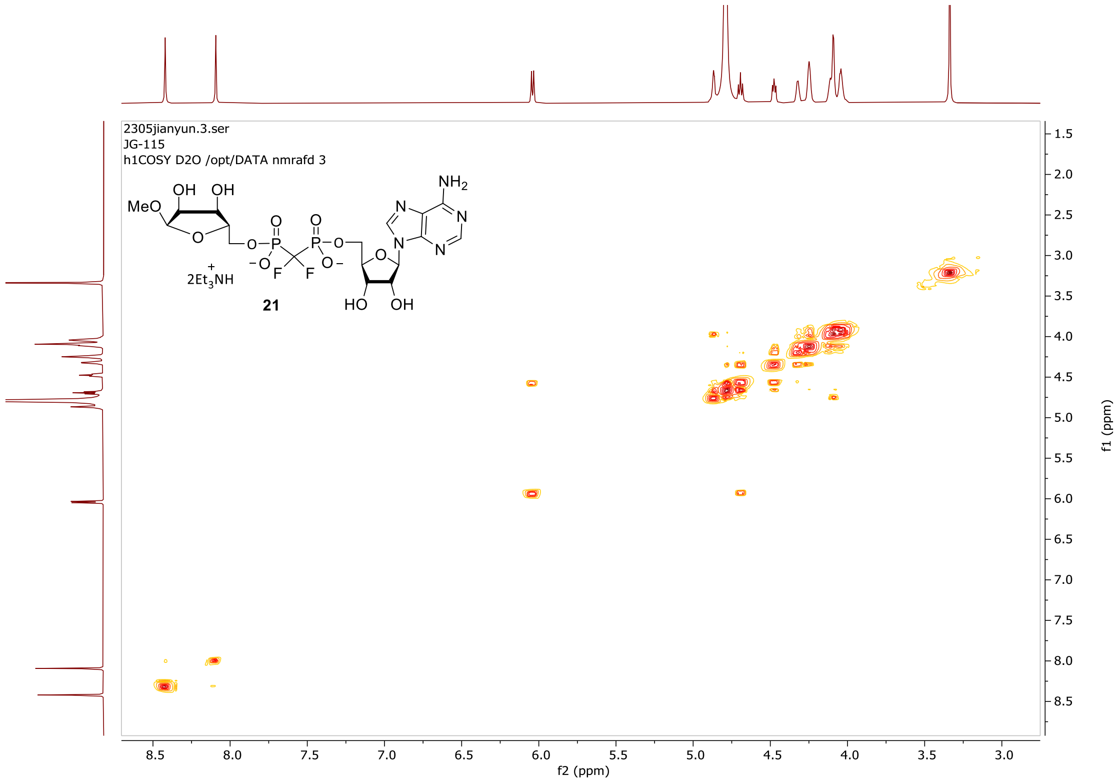

^{1}H - ^{1}H COSY (101 MHz, $CDCl_3$) of compound **21**

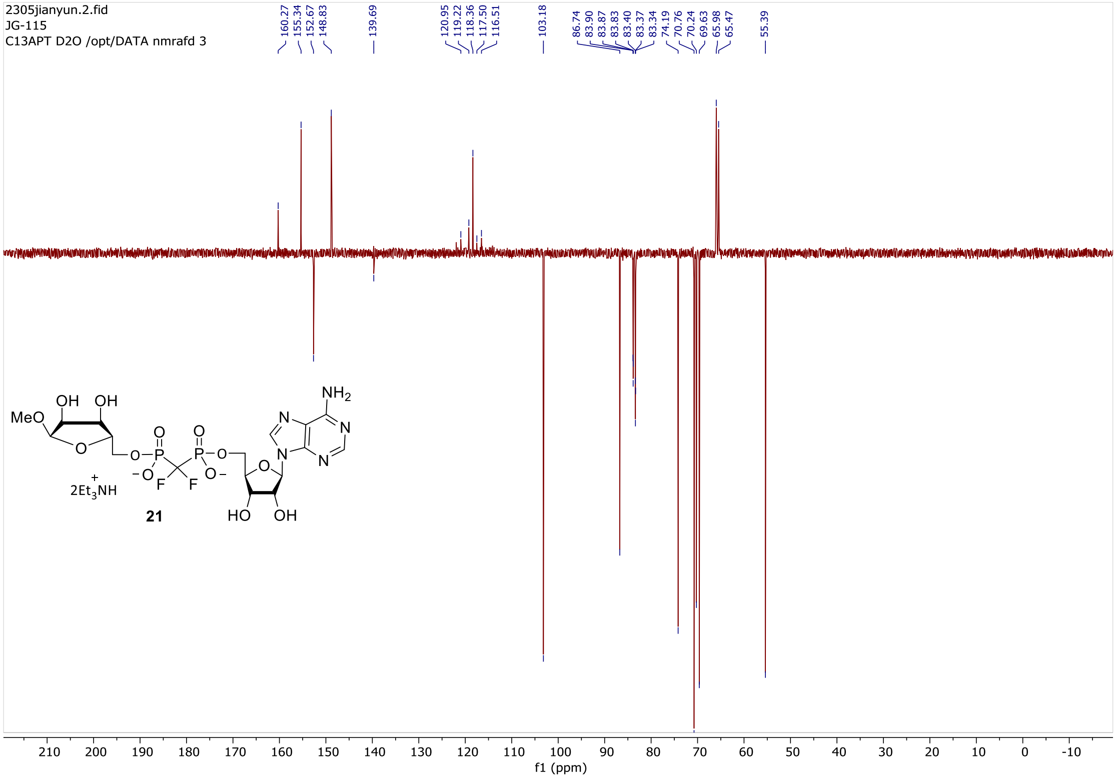

^{13}C NMR (101 MHz, CDCl$_3$) of compound **21**

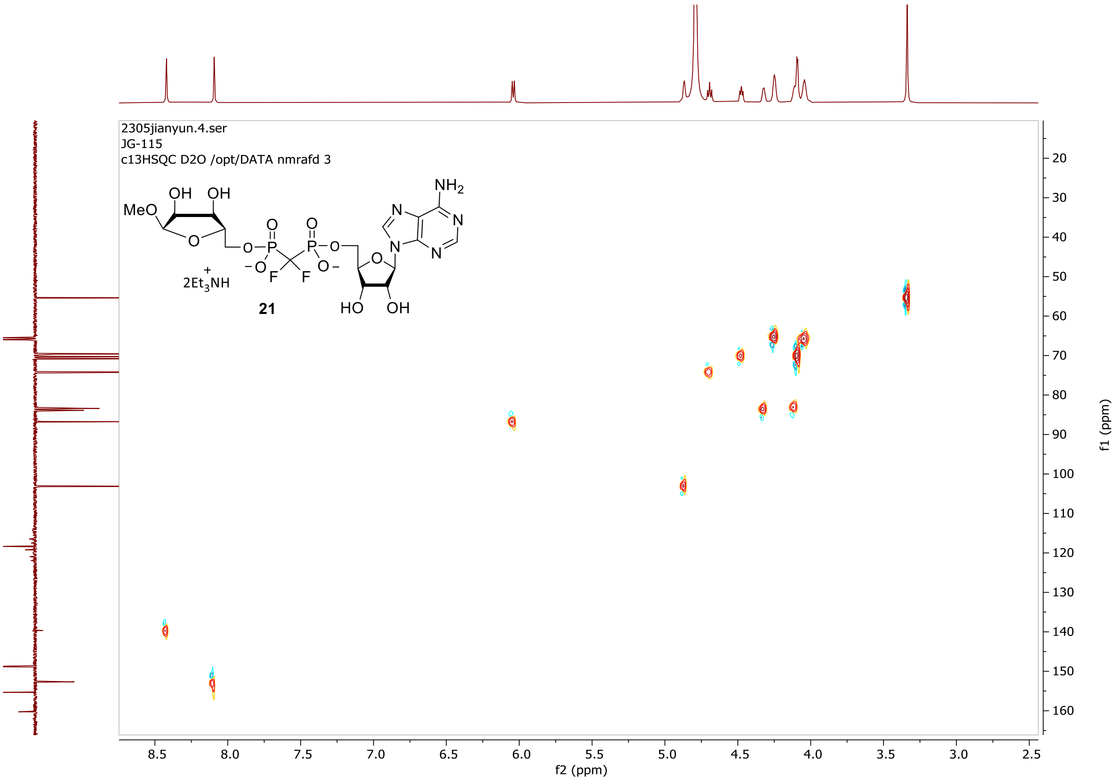

^{1}H - ^{13}C HSQC (400 MHz, CDCl$_3$) of compound **21**

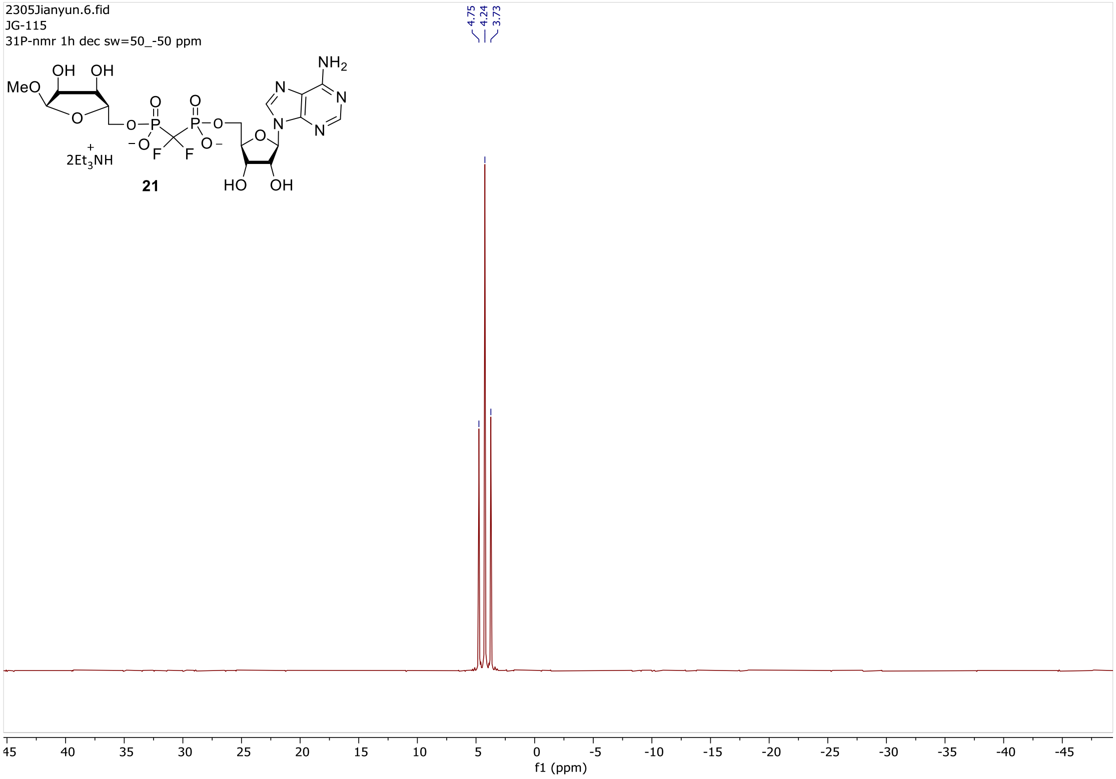

^{31}P NMR (162 MHz, $CDCl_3$) of compound **21**

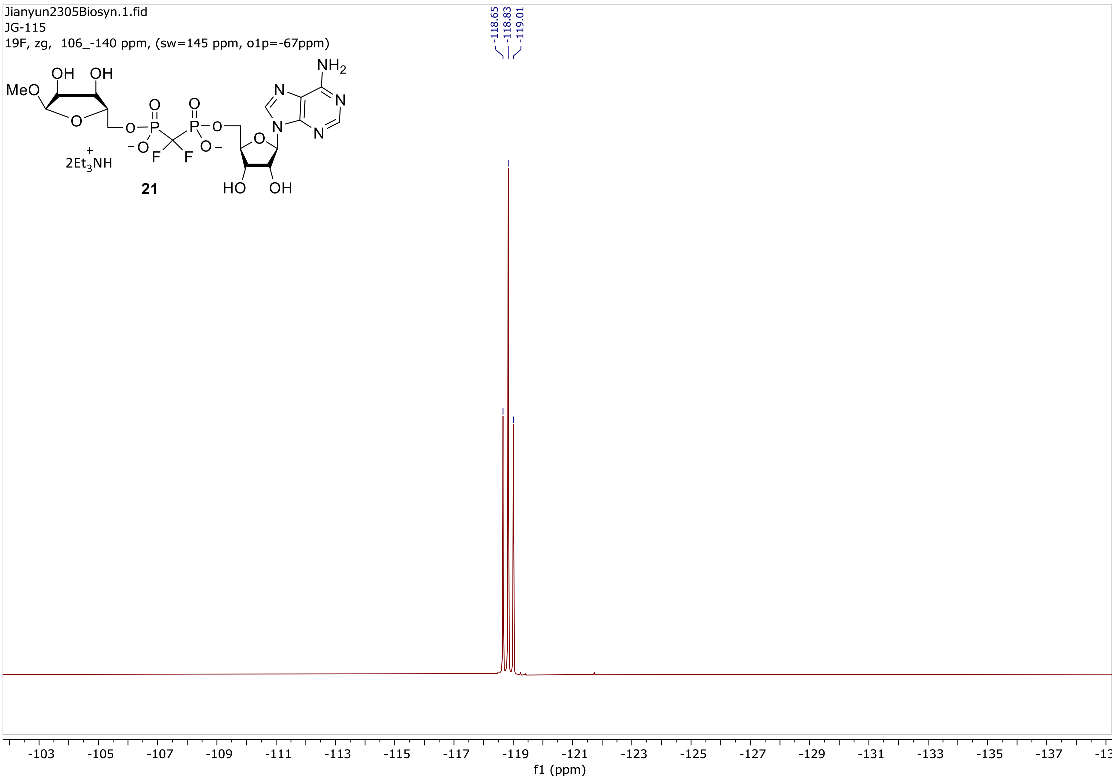

^{19}F NMR (471 MHz, $CDCl_3$) of compound **21**

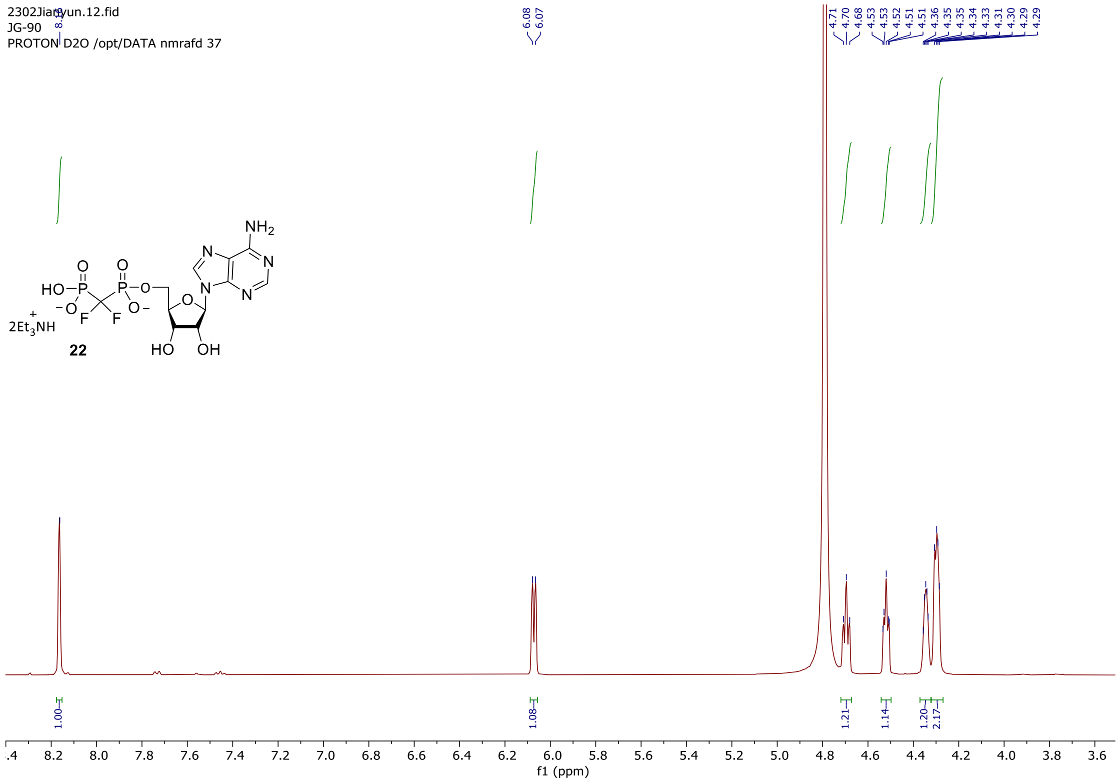

^{1}H NMR (400 MHz, $CDCl_3$) of compound **22**

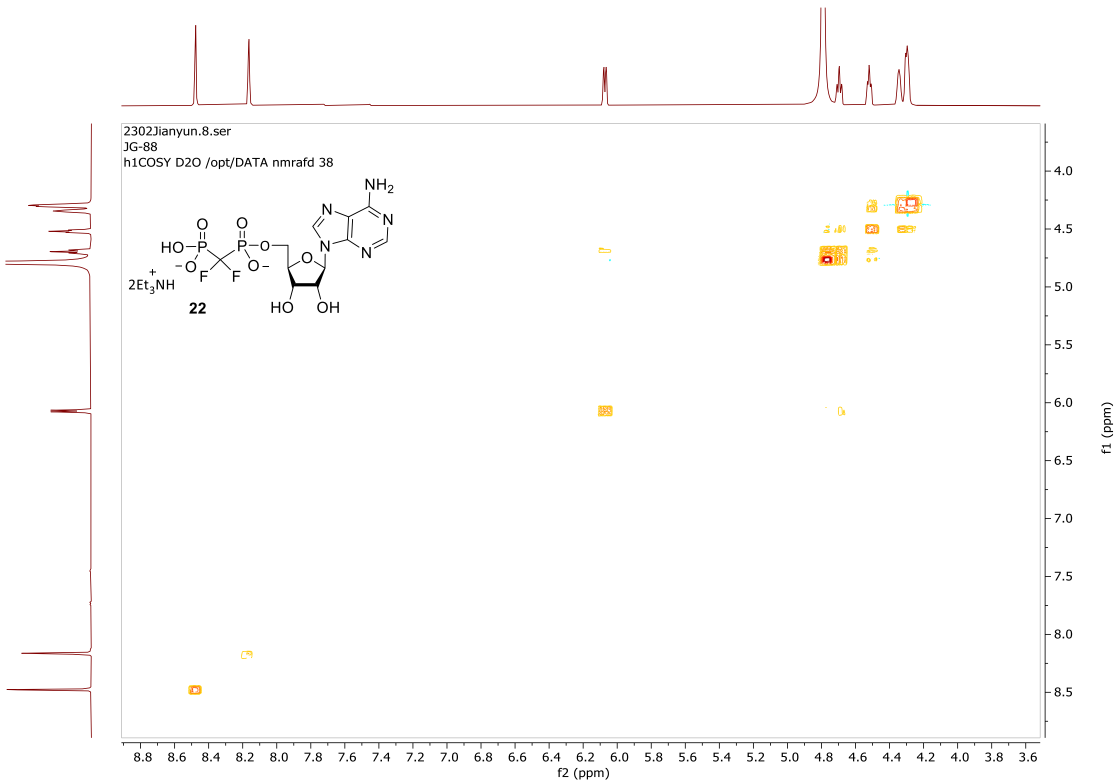

^{1}H - ^{1}H COSY (101 MHz, $CDCl_3$) of compound **22**

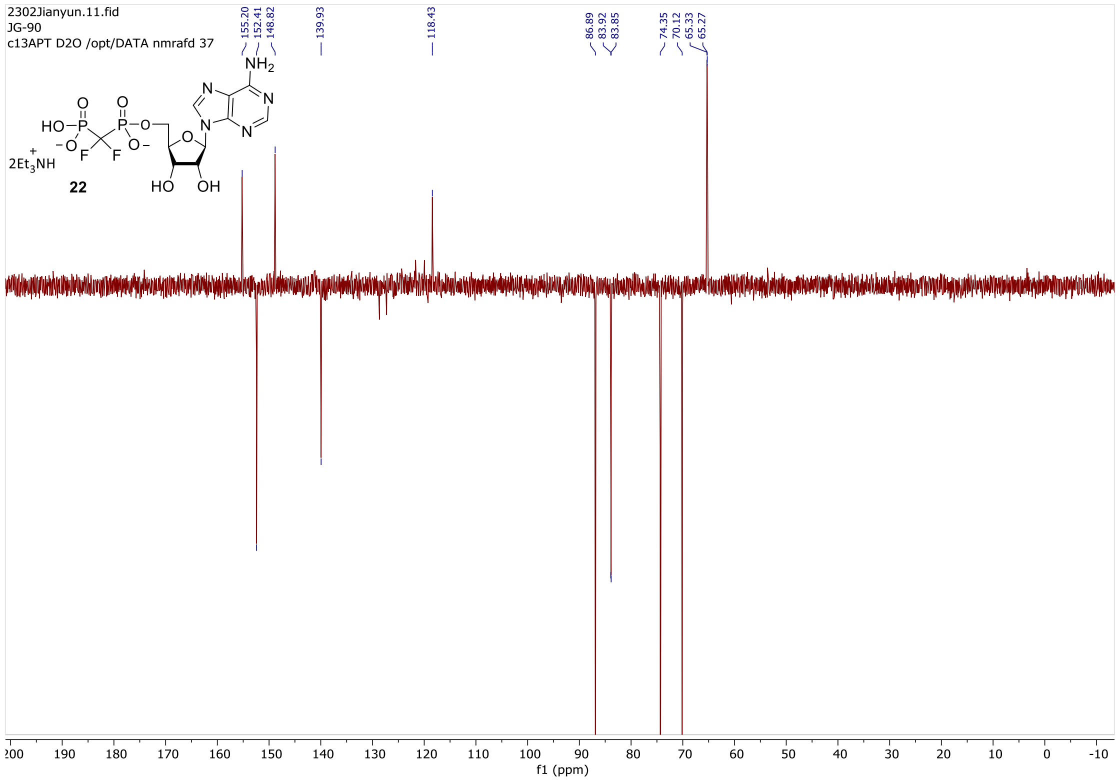

^{13}C NMR (101 MHz, $CDCl_3$) of compound **22**

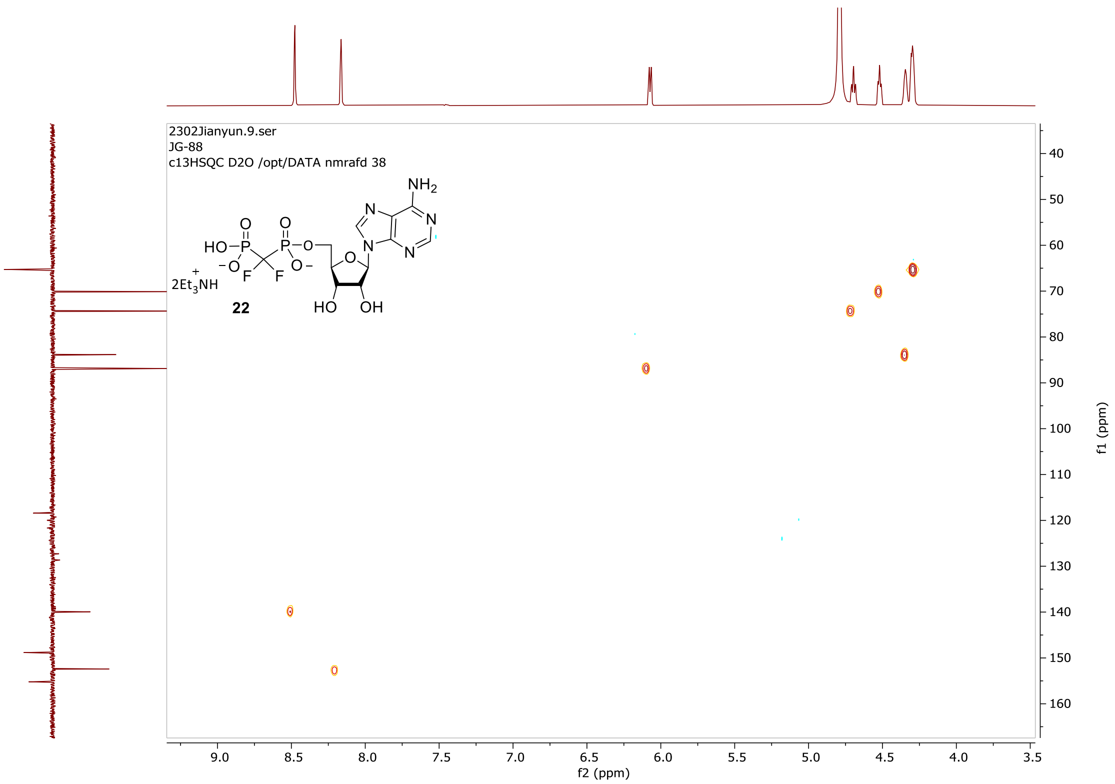

^{1}H - ^{13}C HSQC (400 MHz, $CDCl_3$) of compound **22**

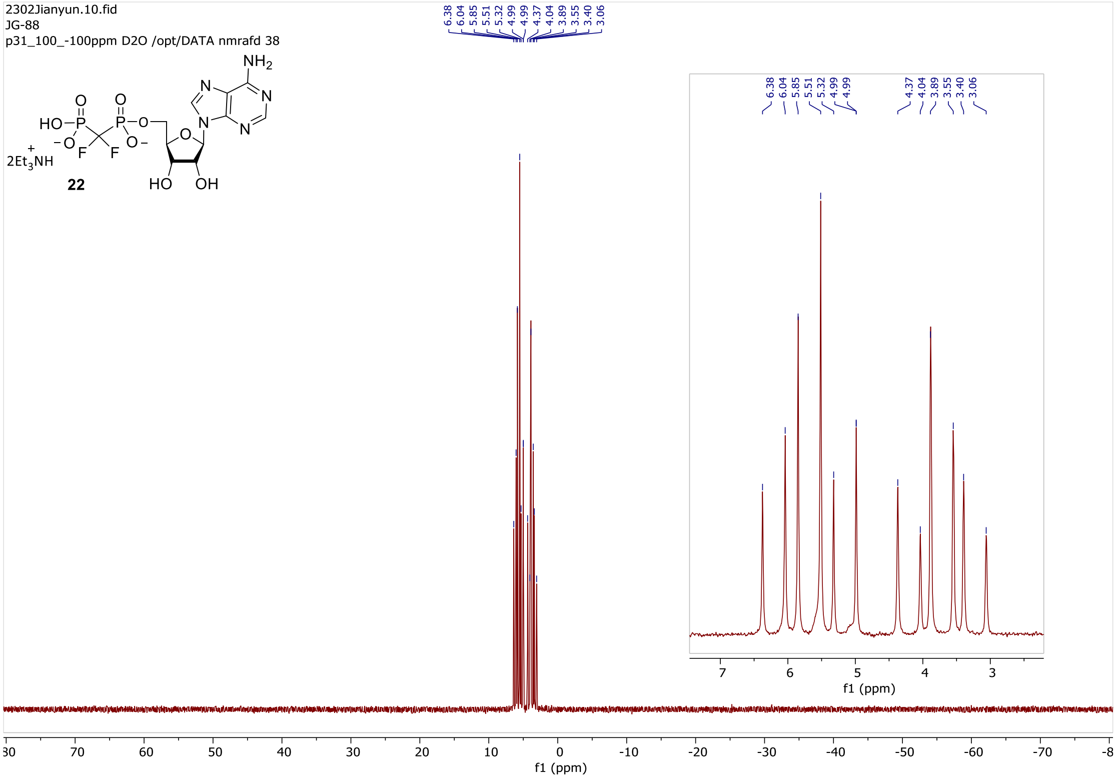

^{31}P NMR (162 MHz, $CDCl_3$) of compound **22**

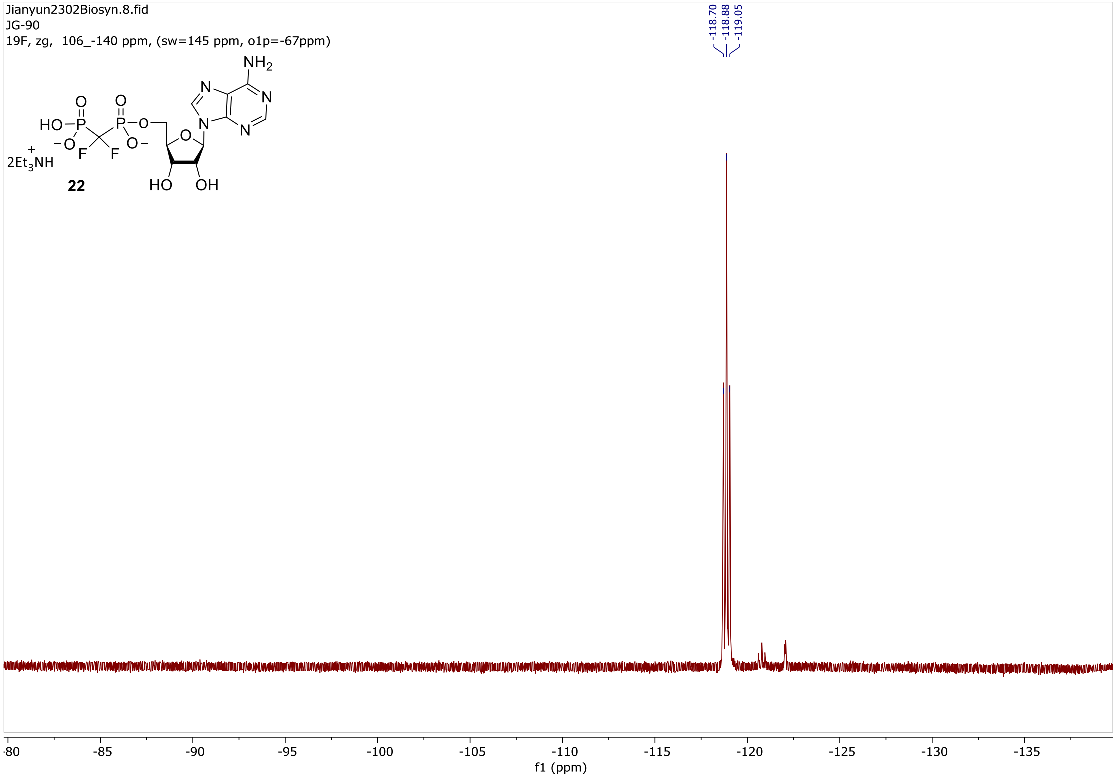

^{19}F NMR (471 MHz, $CDCl_3$) of compound **22**

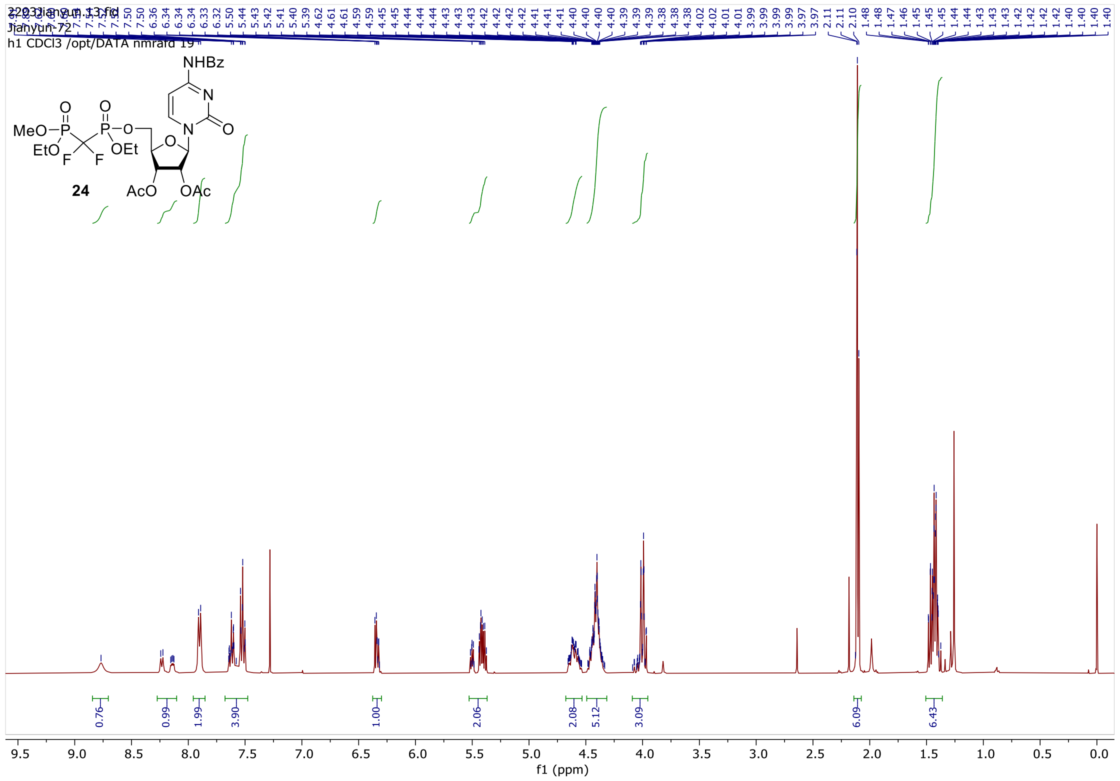

^{1}H NMR (400 MHz, $CDCl_3$) of compound **24**

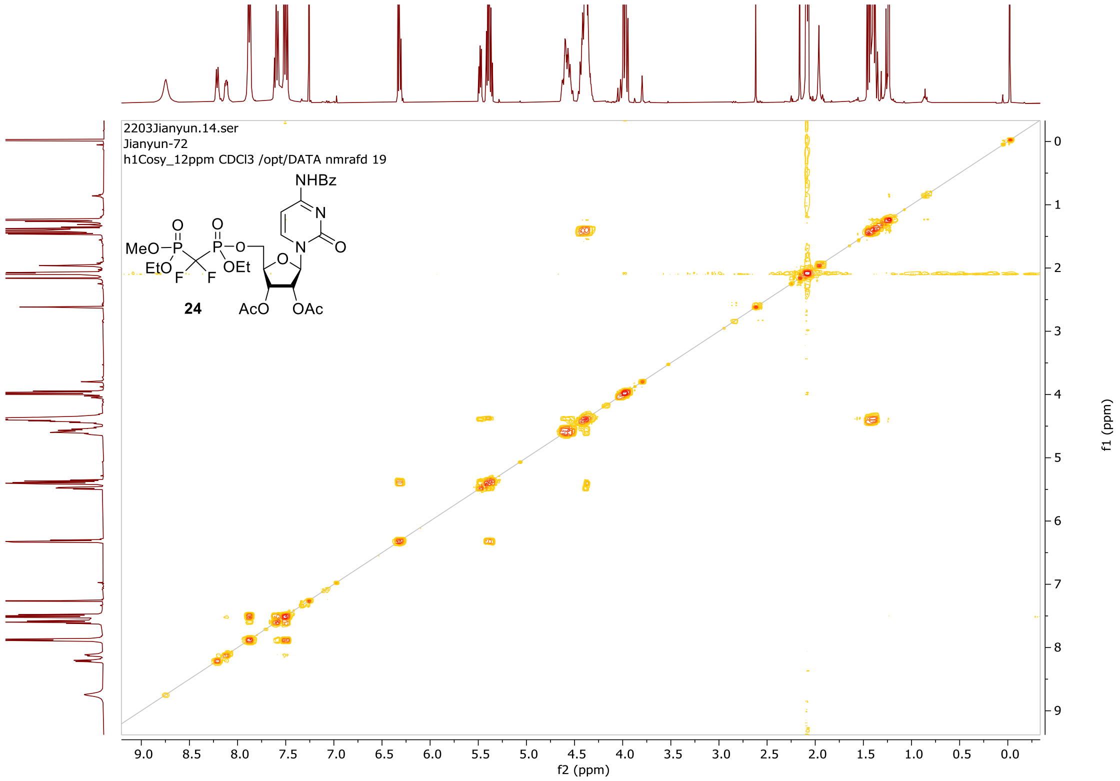

^{1}H - ^{1}H COSY (101 MHz, $CDCl_3$) of compound **24**

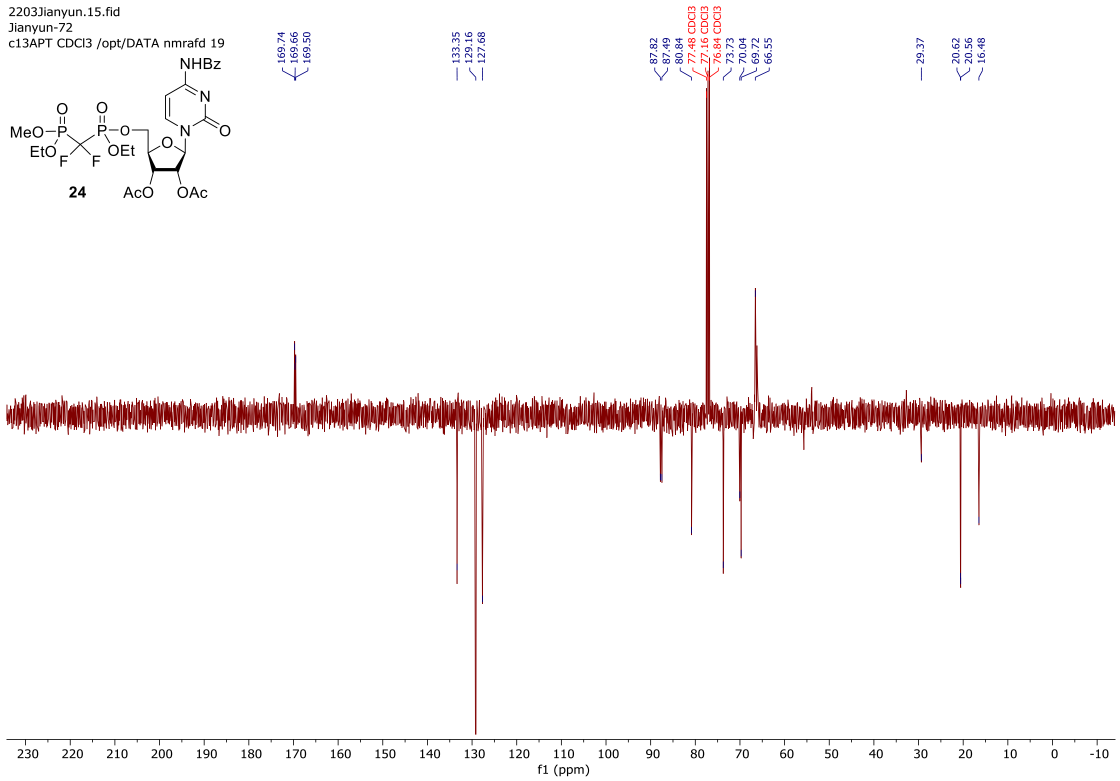

^{13}C NMR (101 MHz, $CDCl_3$) of compound **24**

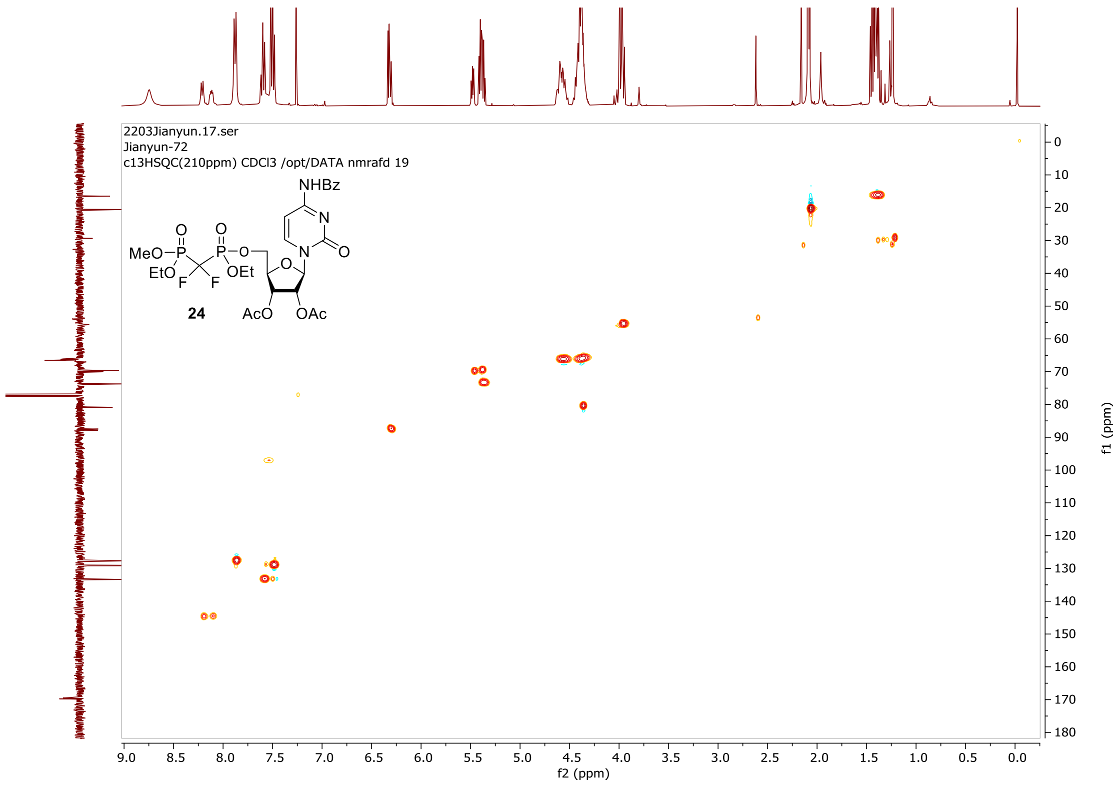

^{1}H - ^{13}C HSQC (400 MHz, $CDCl_3$) of compound **24**

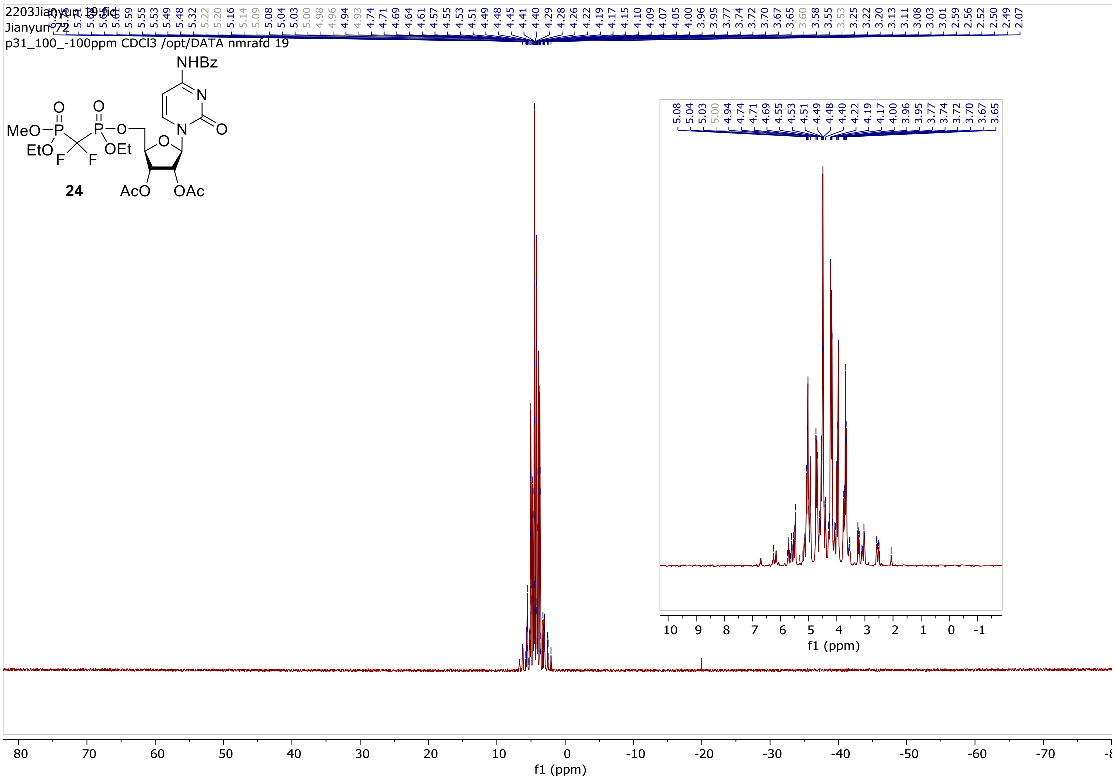

^{31}P NMR (162 MHz, $CDCl_3$) of compound **24**

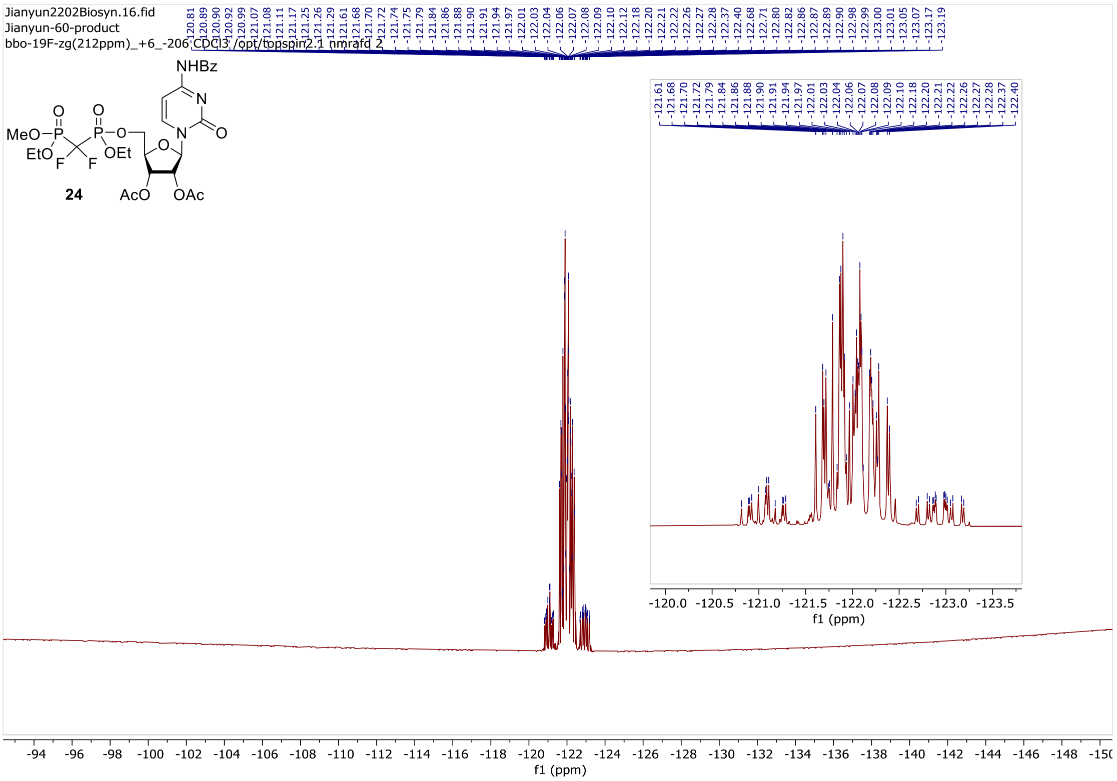

^{19}F NMR (471 MHz, $CDCl_3$) of compound **24**

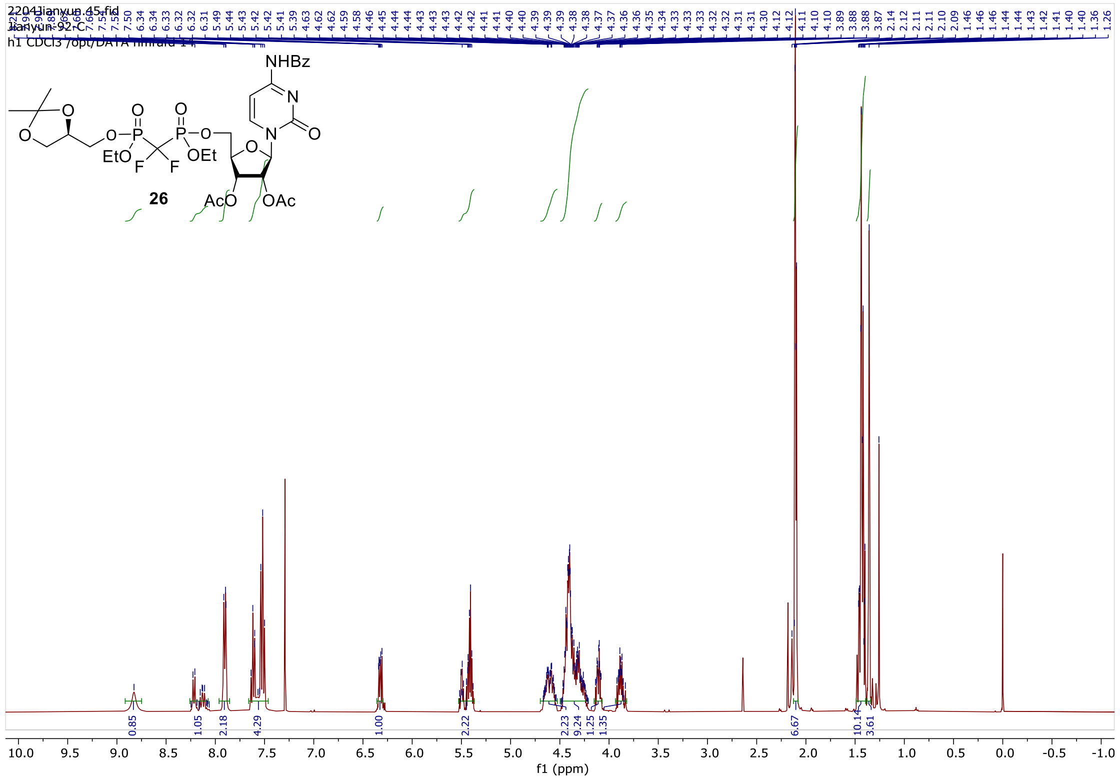

^{1}H NMR (400 MHz, $CDCl_3$) of compound **26**

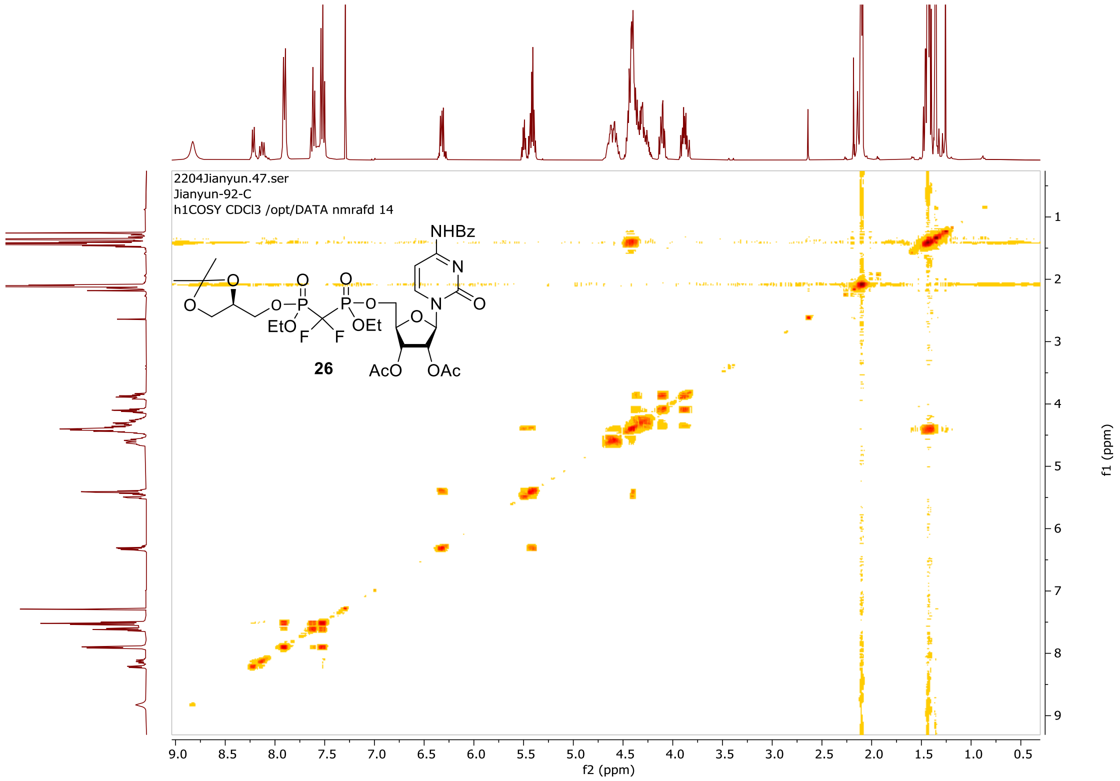

^{1}H - ^{1}H COSY (101 MHz, $CDCl_3$) of compound **26**

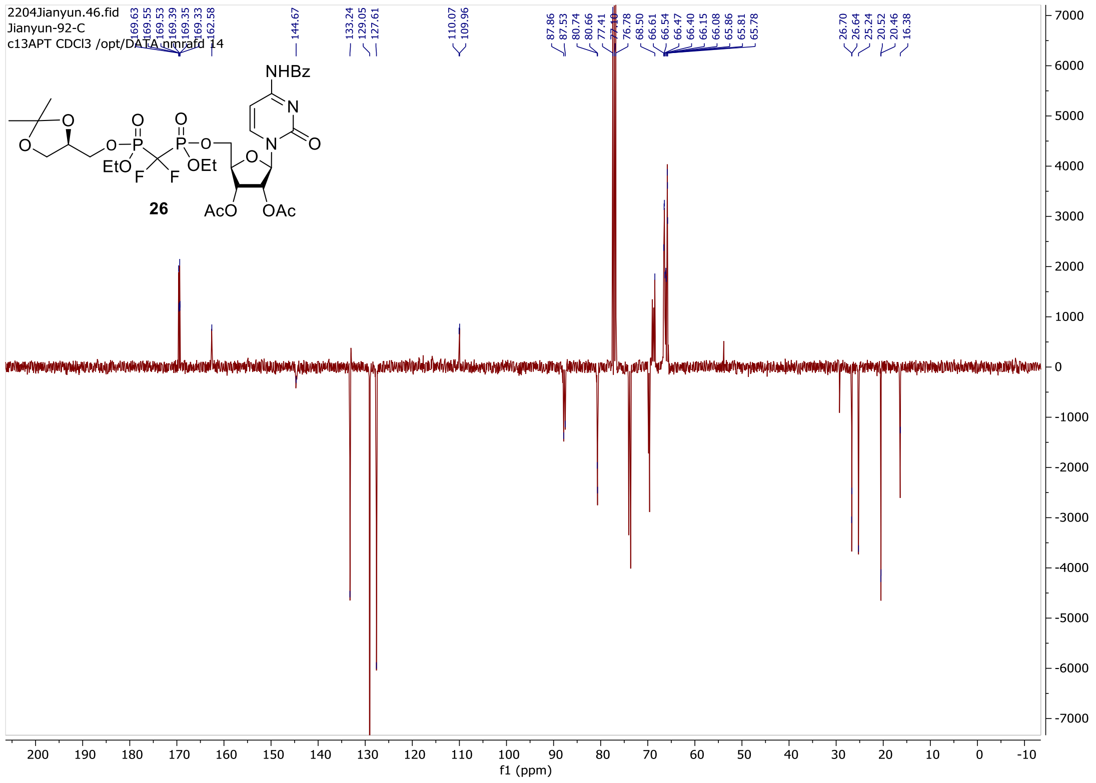

^{13}C NMR (101 MHz, $CDCl_3$) of compound **26**

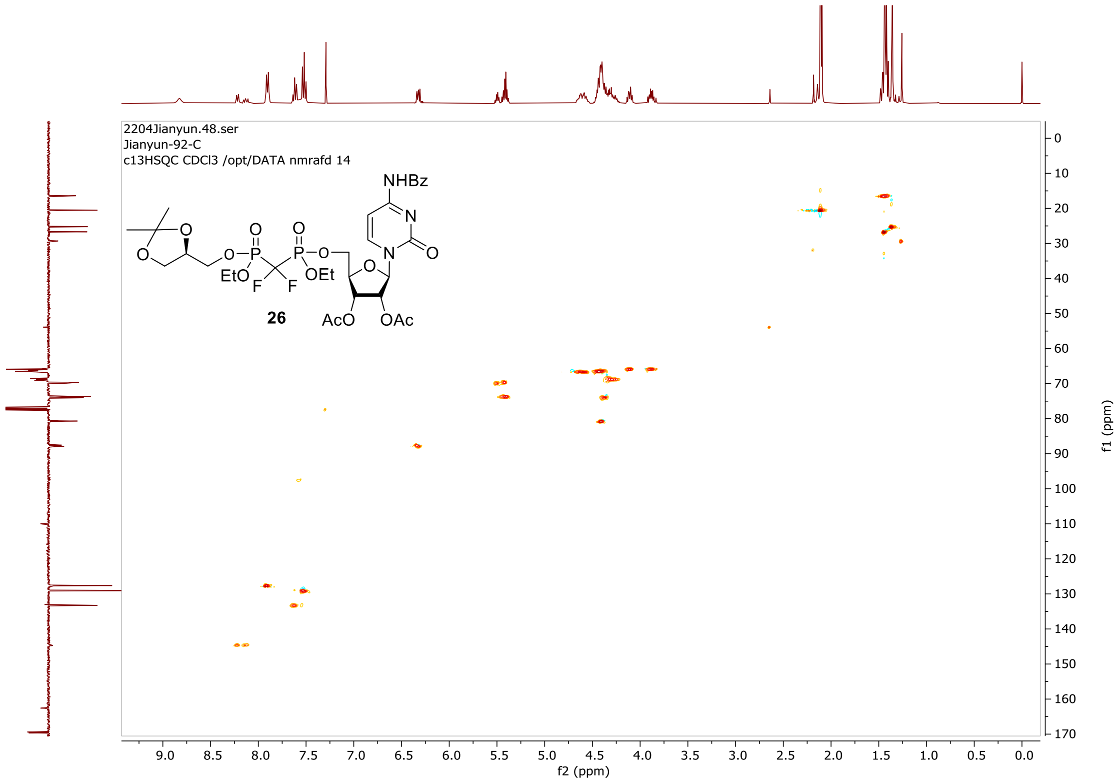

^{1}H - ^{13}C HSQC (400 MHz, $CDCl_3$) of compound **26**

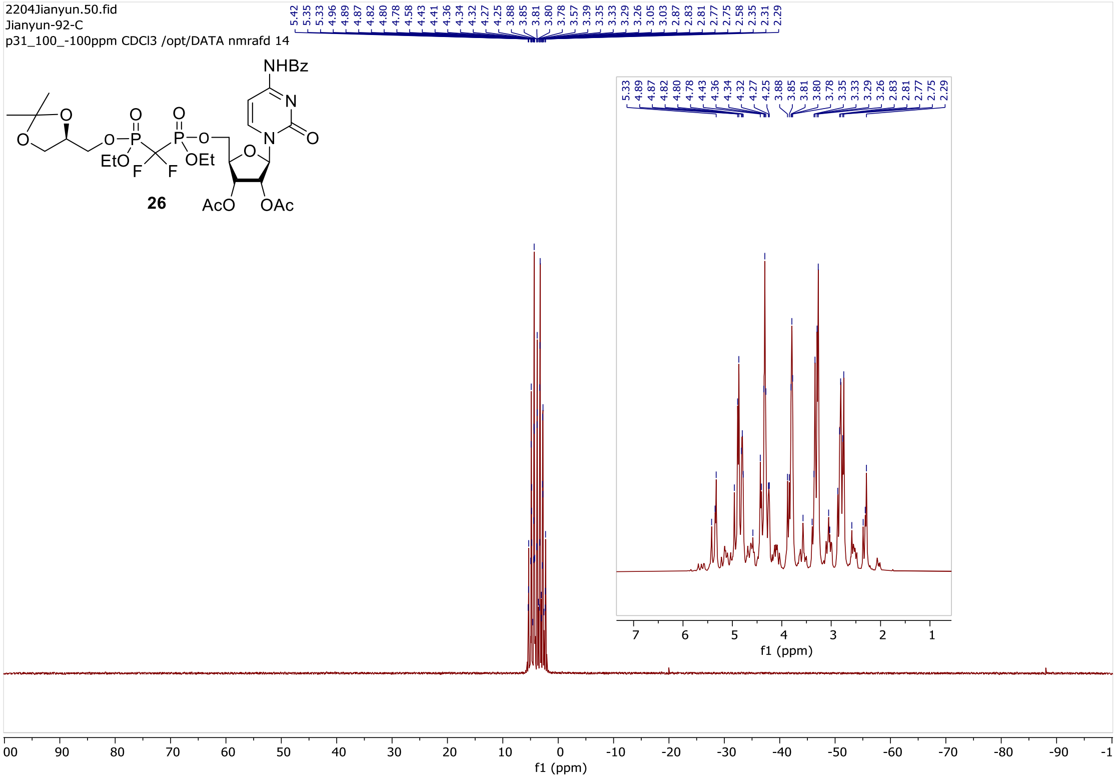

^{31}P NMR (162 MHz, $CDCl_3$) of compound **26**

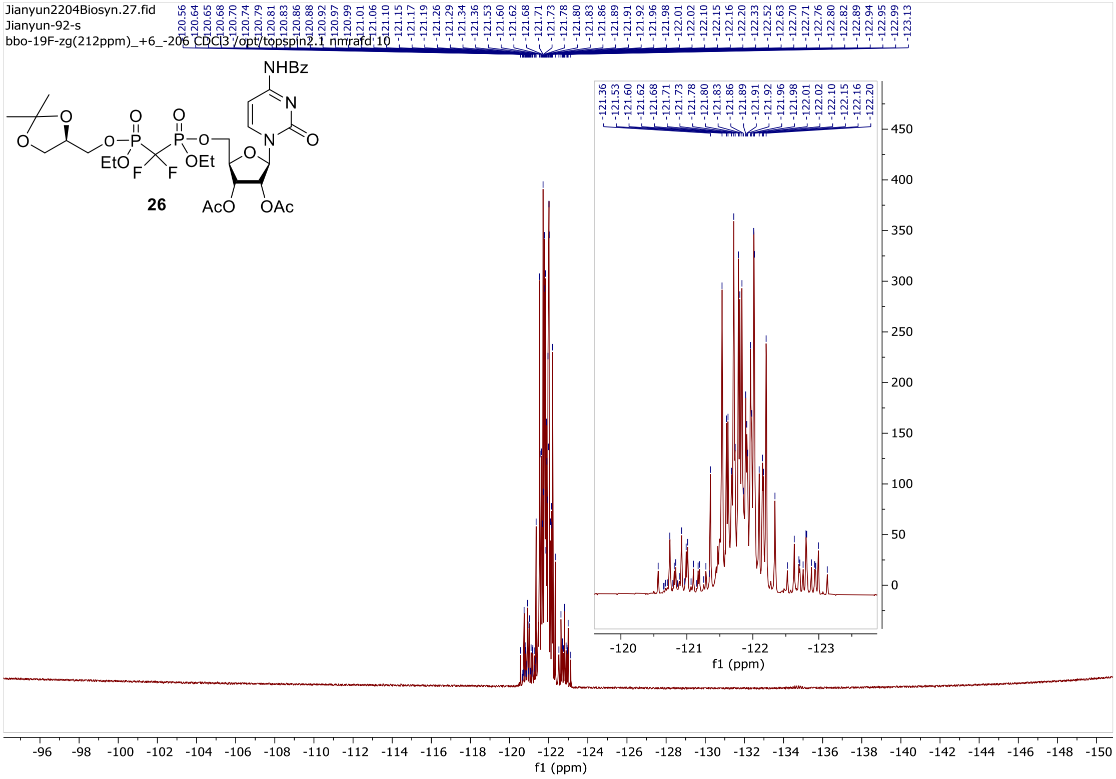

^{19}F NMR (376 MHz, $CDCl_3$) of compound **26**

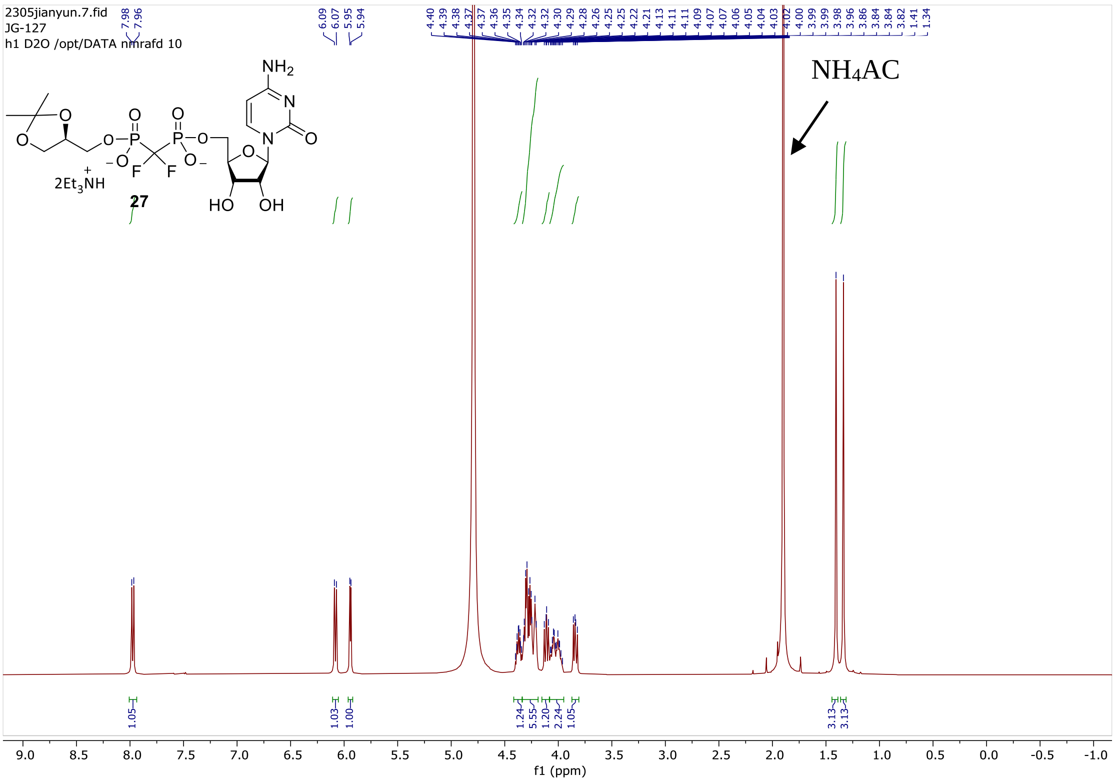

^{1}H NMR (400 MHz, CDCl$_3$) of compound **27**

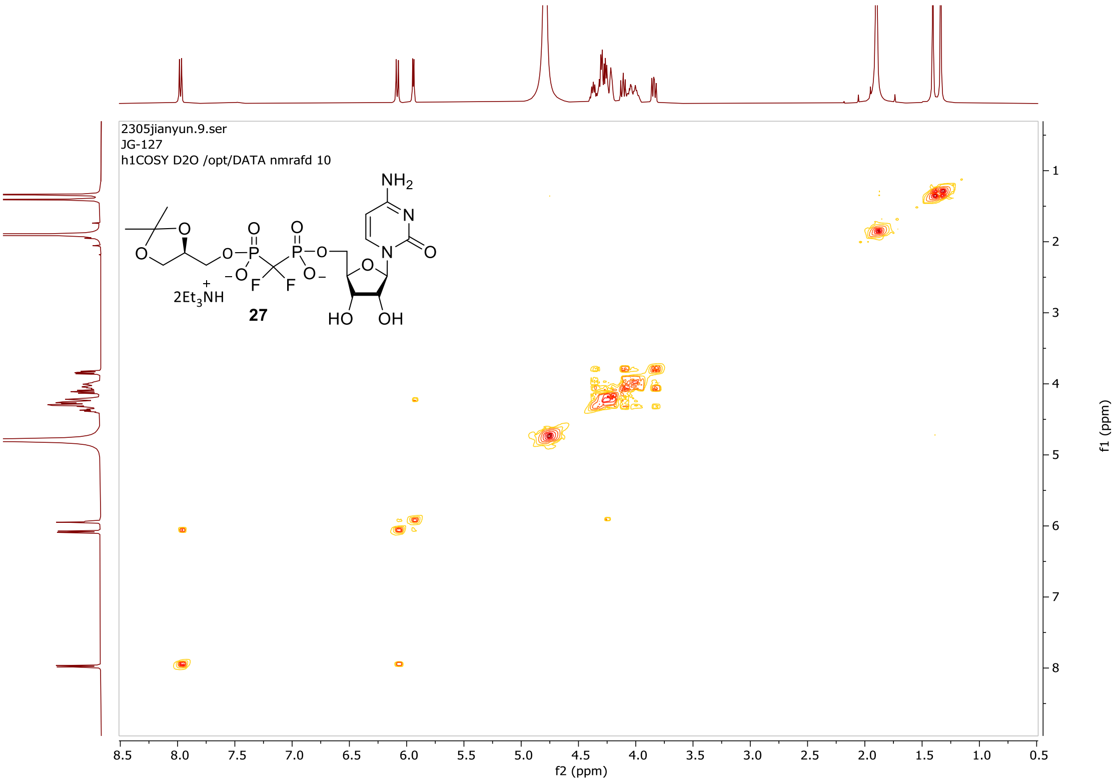

^{1}H - ^{1}H COSY (101 MHz, CDCl$_3$) of compound **27**

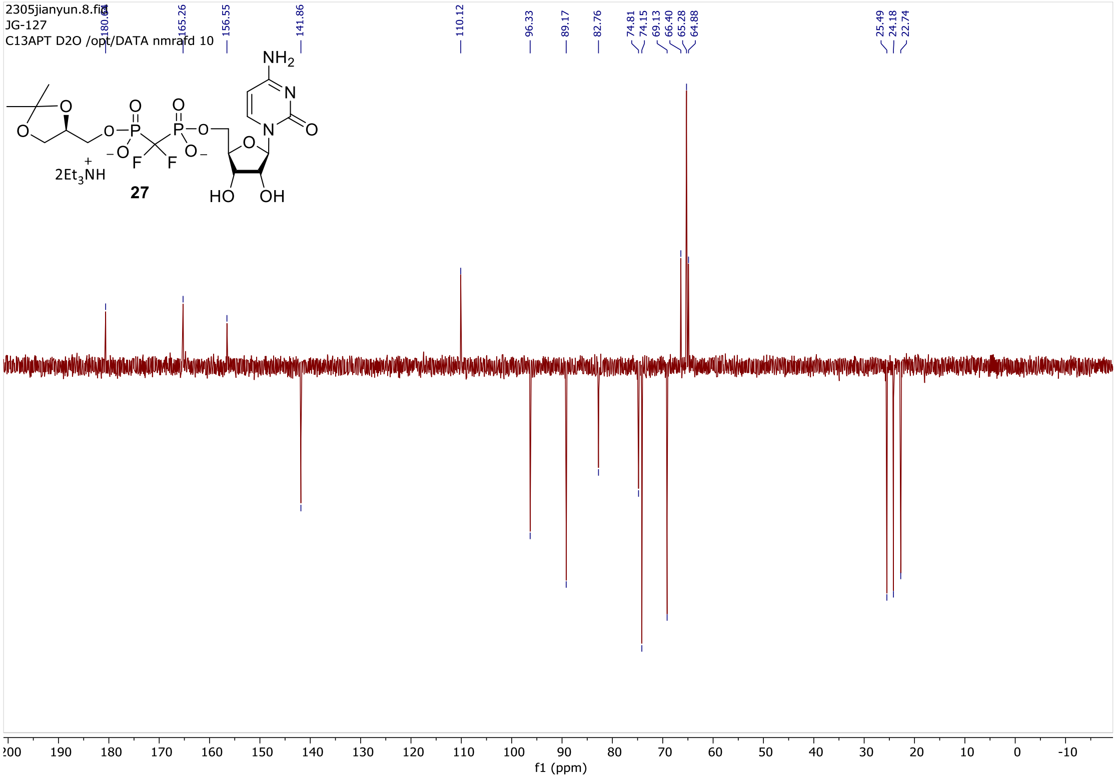

^{13}C NMR (101 MHz, $CDCl_3$) of compound **27**

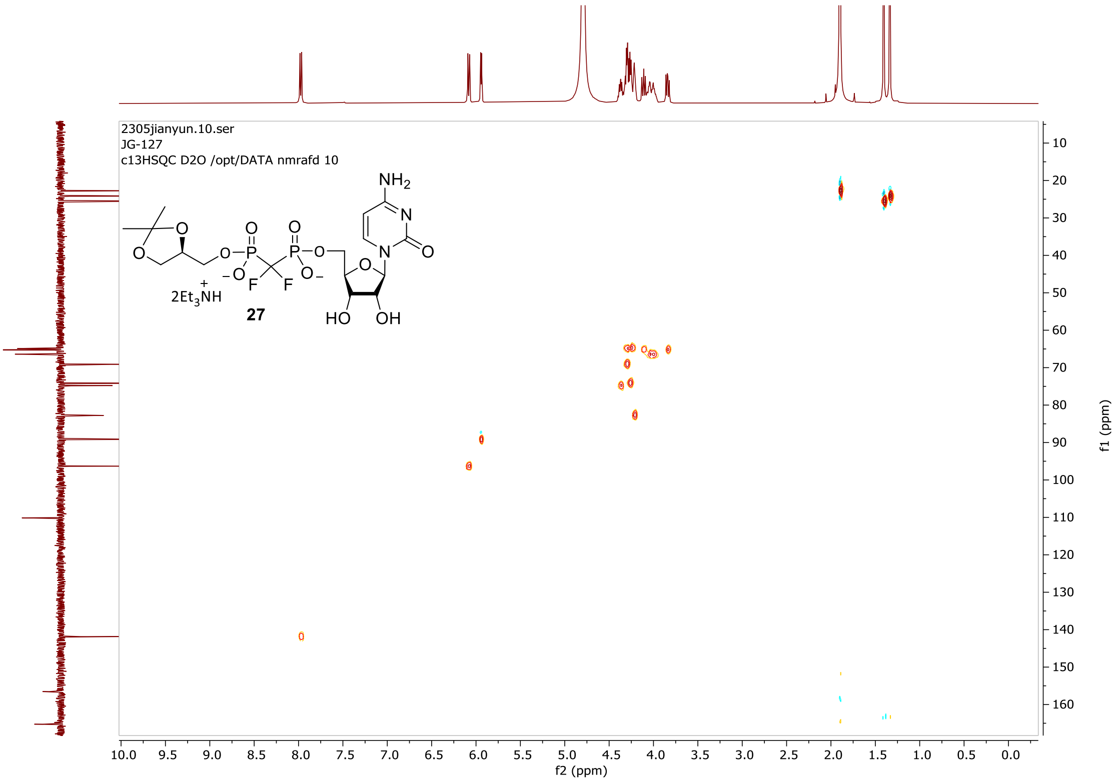

^{1}H - ^{13}C HSQC (400 MHz, $CDCl_3$) of compound **27**

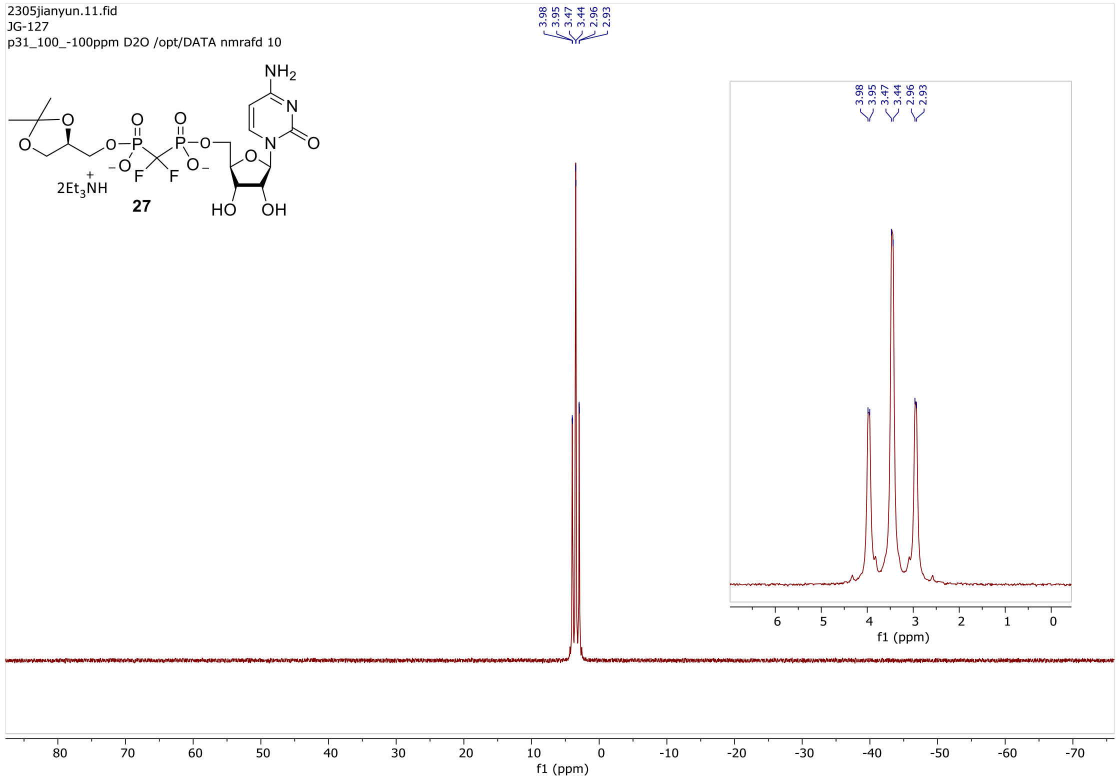

^{31}P NMR (162 MHz, $CDCl_3$) of compound **27**

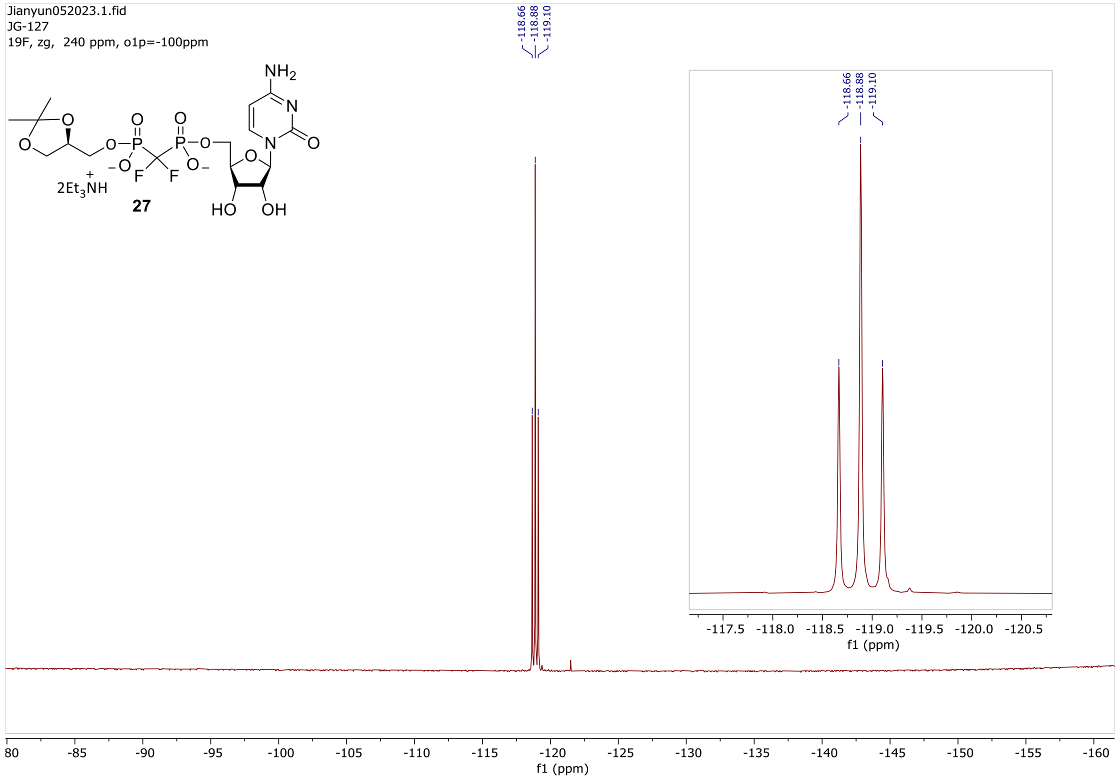

^{19}F NMR (376 MHz, $CDCl_3$) of compound **27**

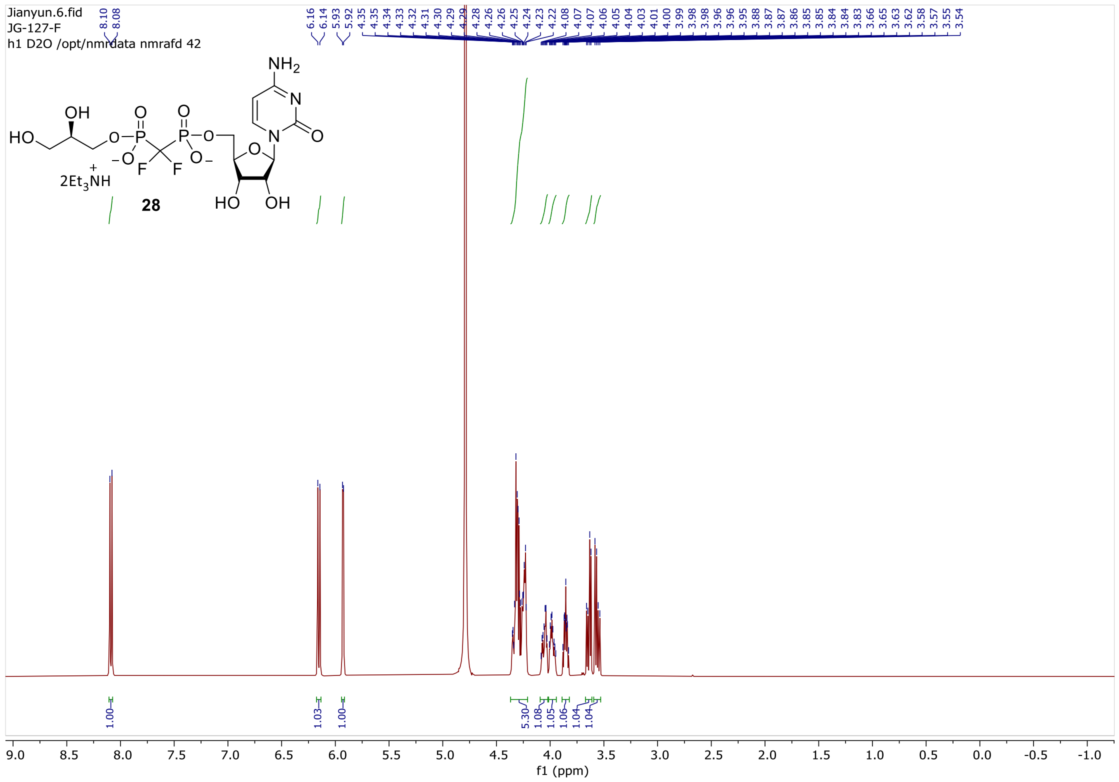

^1H NMR (400 MHz, $CDCl_3$) of compound **28**

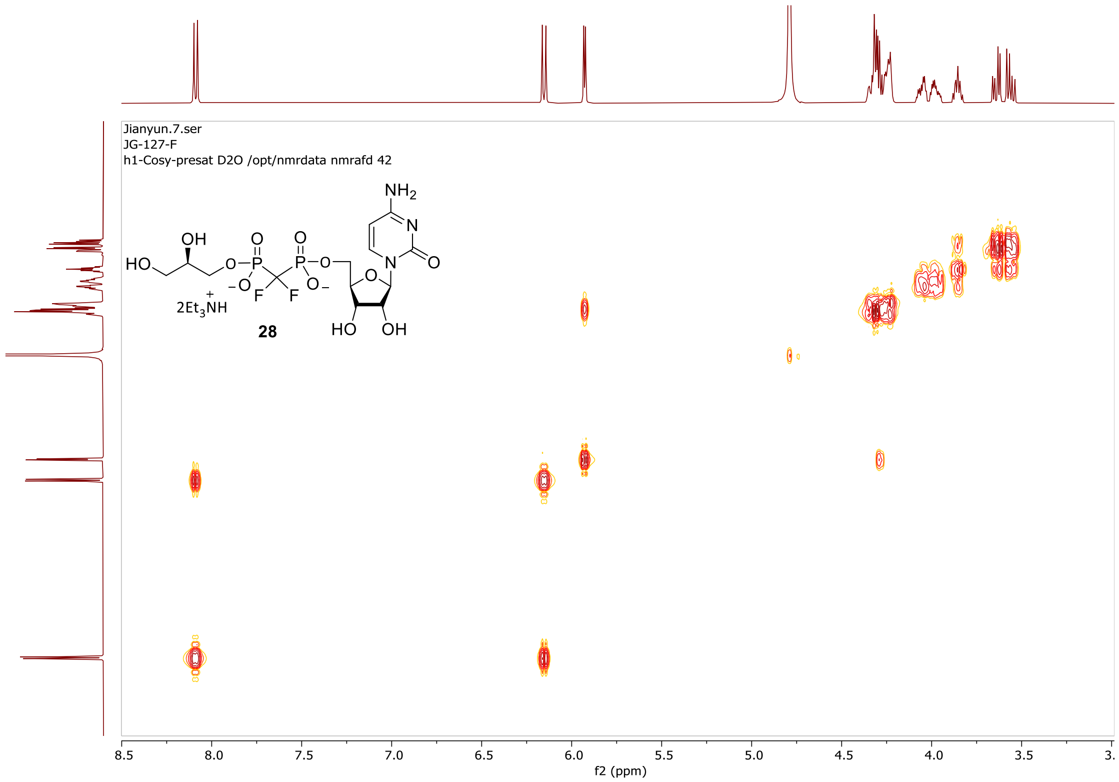

^1H - ^1H COSY (101 MHz, $CDCl_3$) of compound **28**

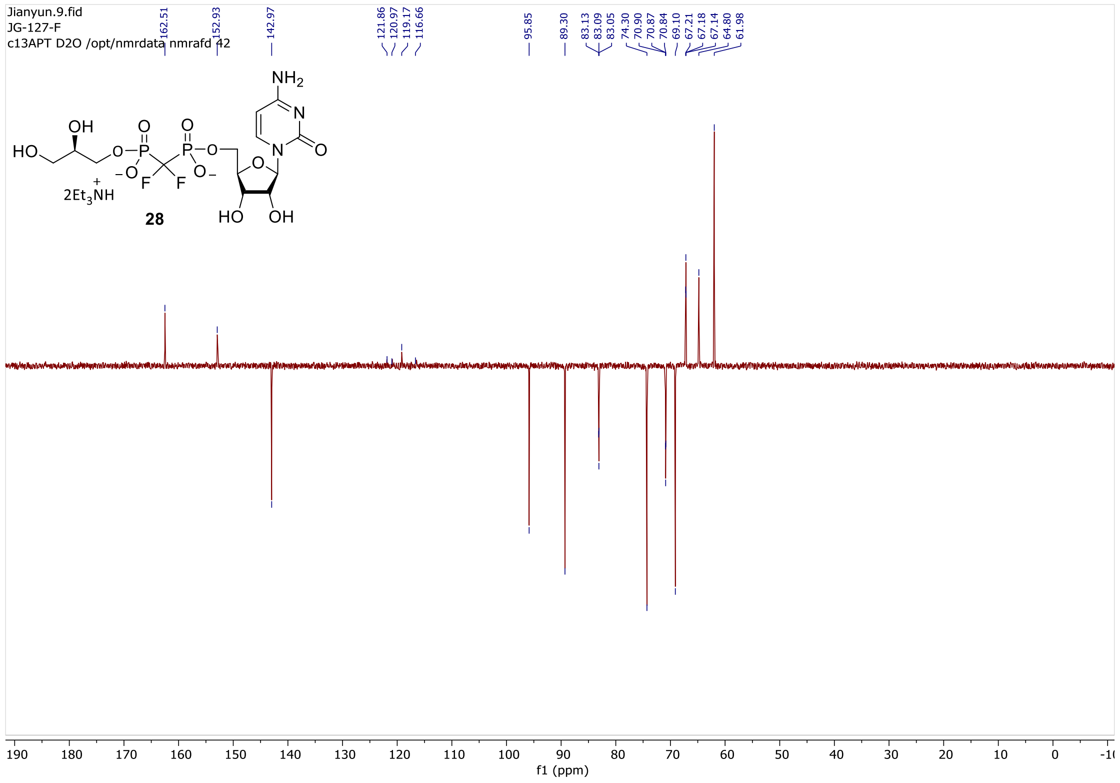

^{13}C NMR (101 MHz, $CDCl_3$) of compound **28**

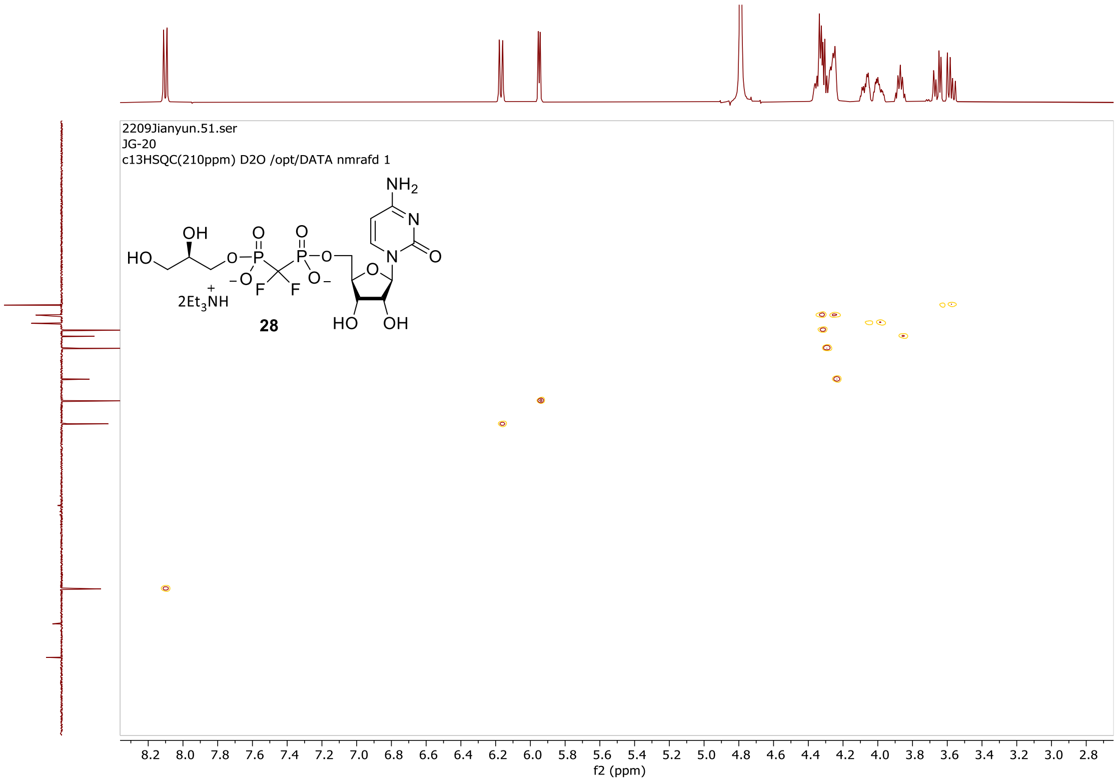

^{1}H - ^{13}C HSQC (400 MHz, $CDCl_3$) of compound **28**

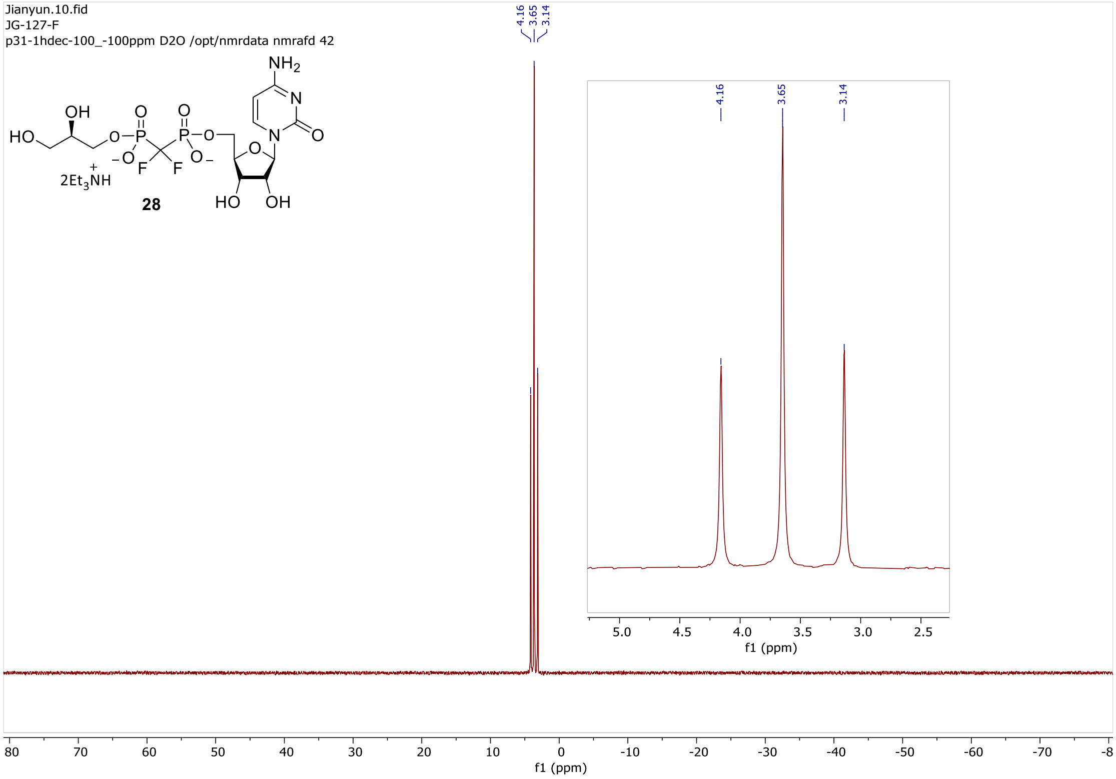

^{31}P NMR (162 MHz, $CDCl_3$) of compound **28**

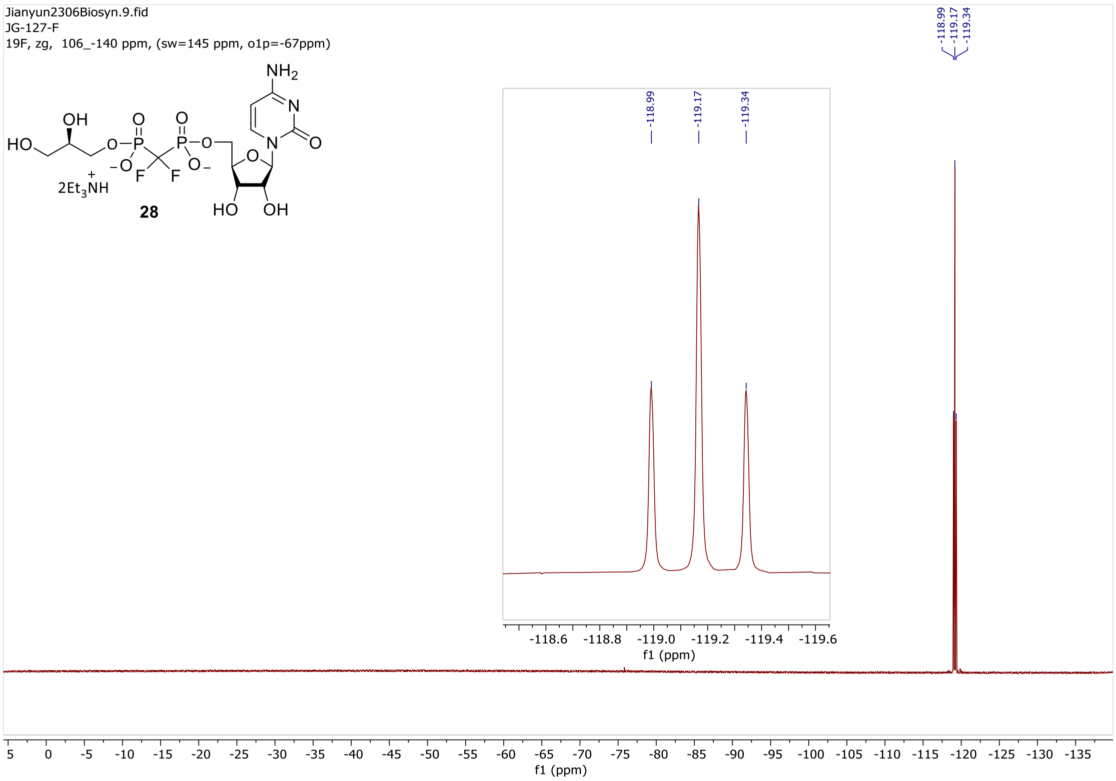

^{19}F NMR (471 MHz, $CDCl_3$) of compound **28**

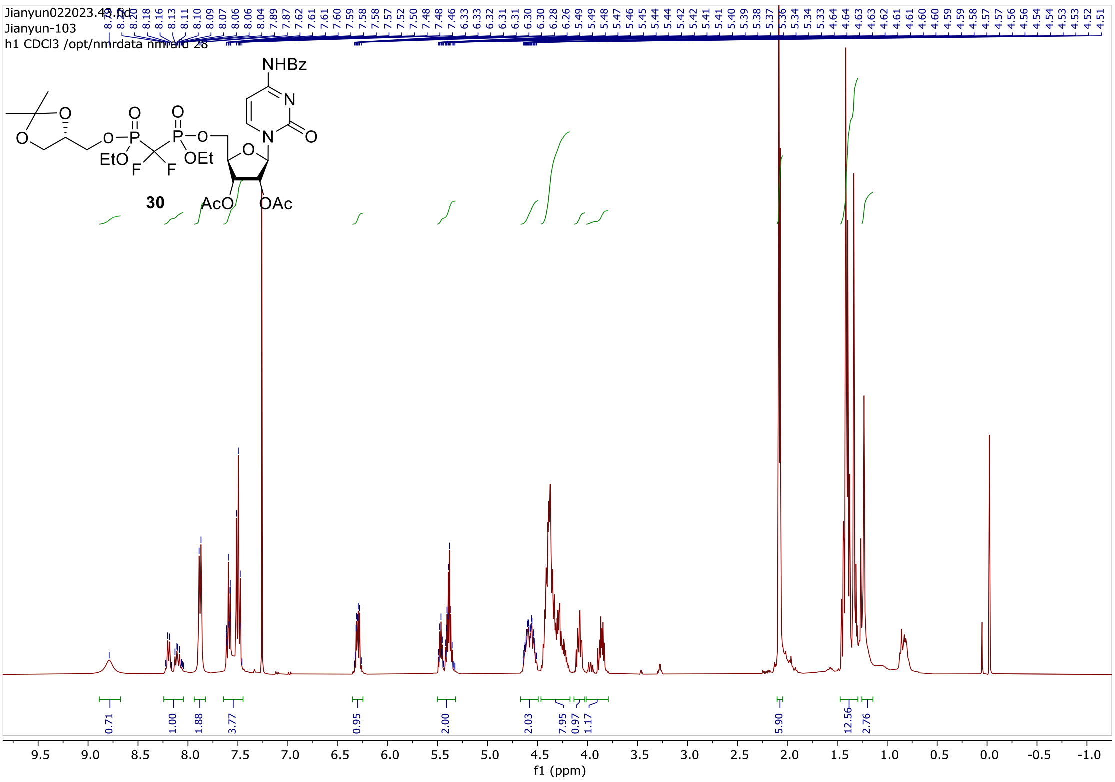

^{1}H NMR (400 MHz, $CDCl_3$) of compound **30**

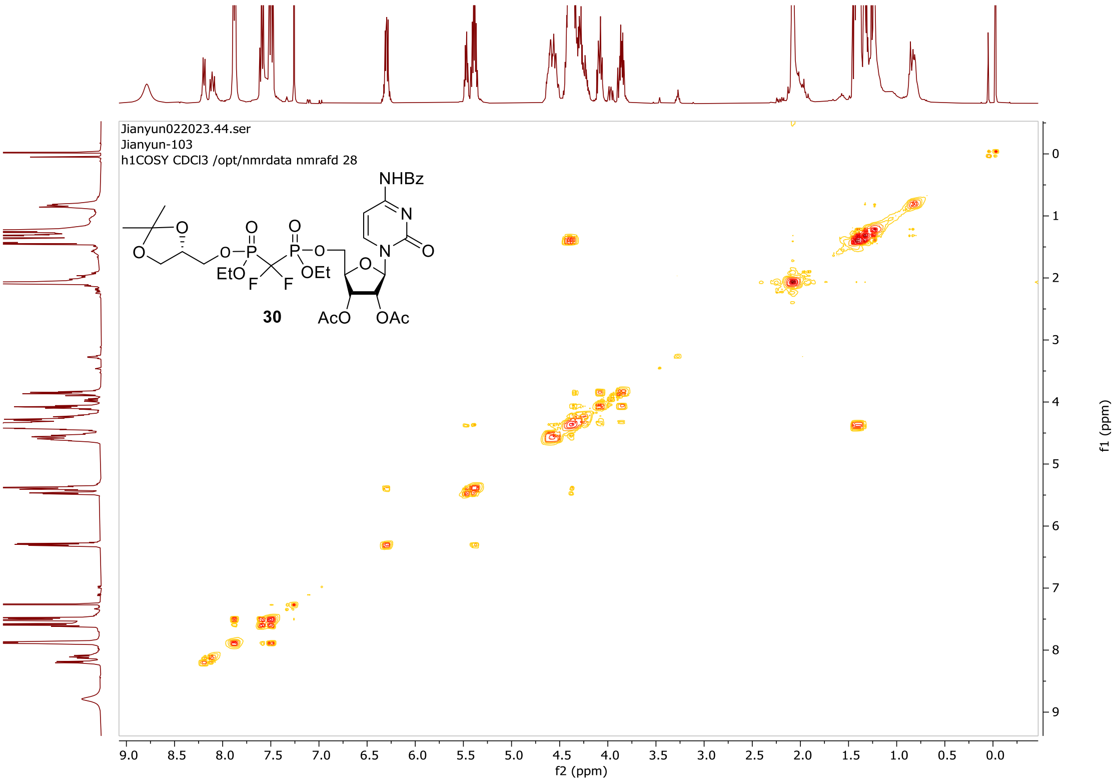

^{1}H - ^{1}H COSY (101 MHz, $CDCl_3$) of compound **30**

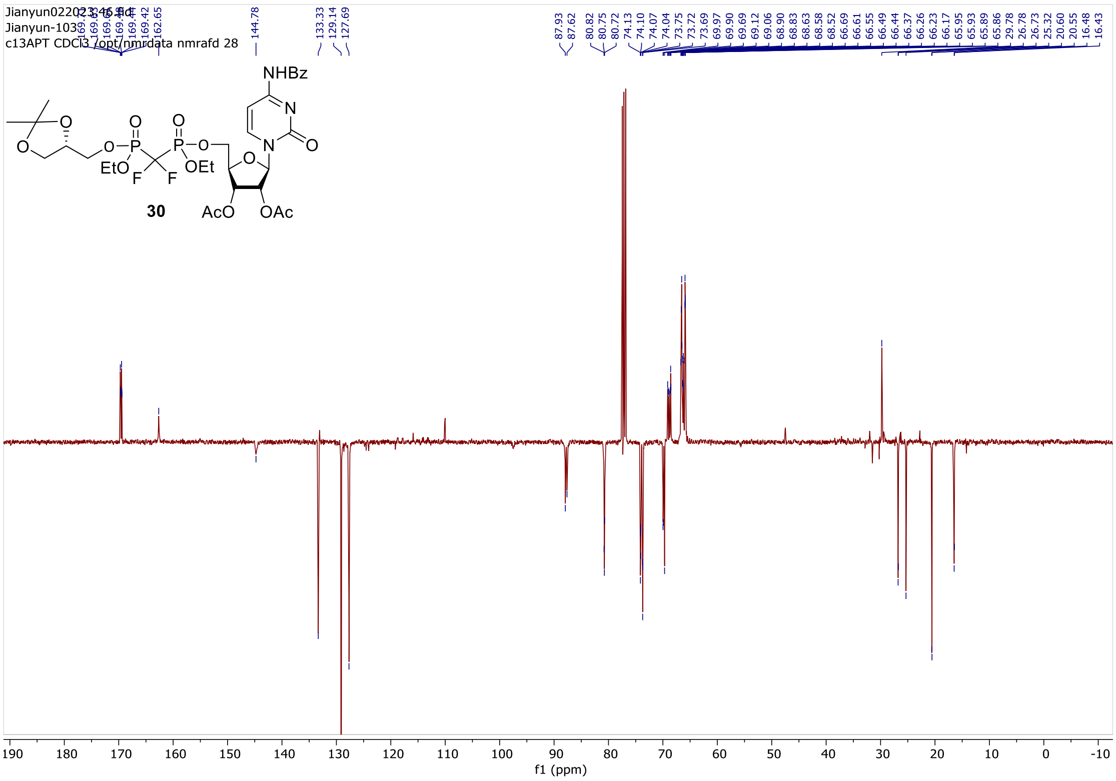

^{13}C NMR (101 MHz, $CDCl_3$) of compound **30**

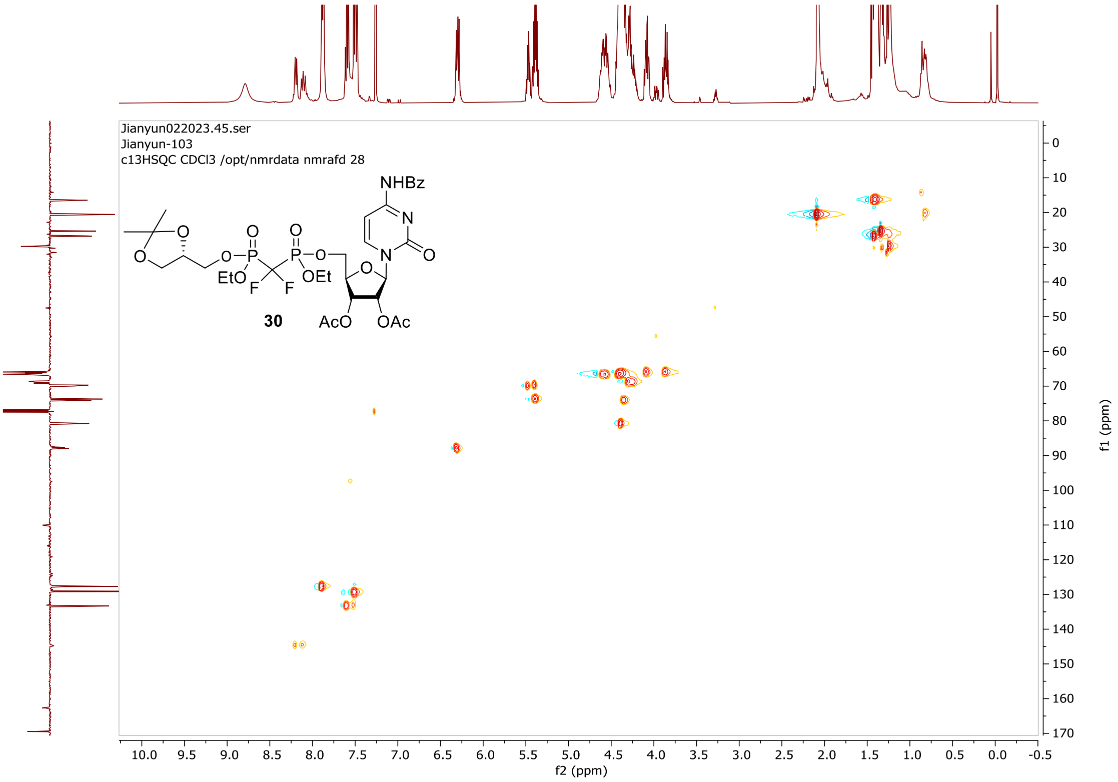

^{1}H - ^{13}C HSQC (400 MHz, $CDCl_3$) of compound 3**0**

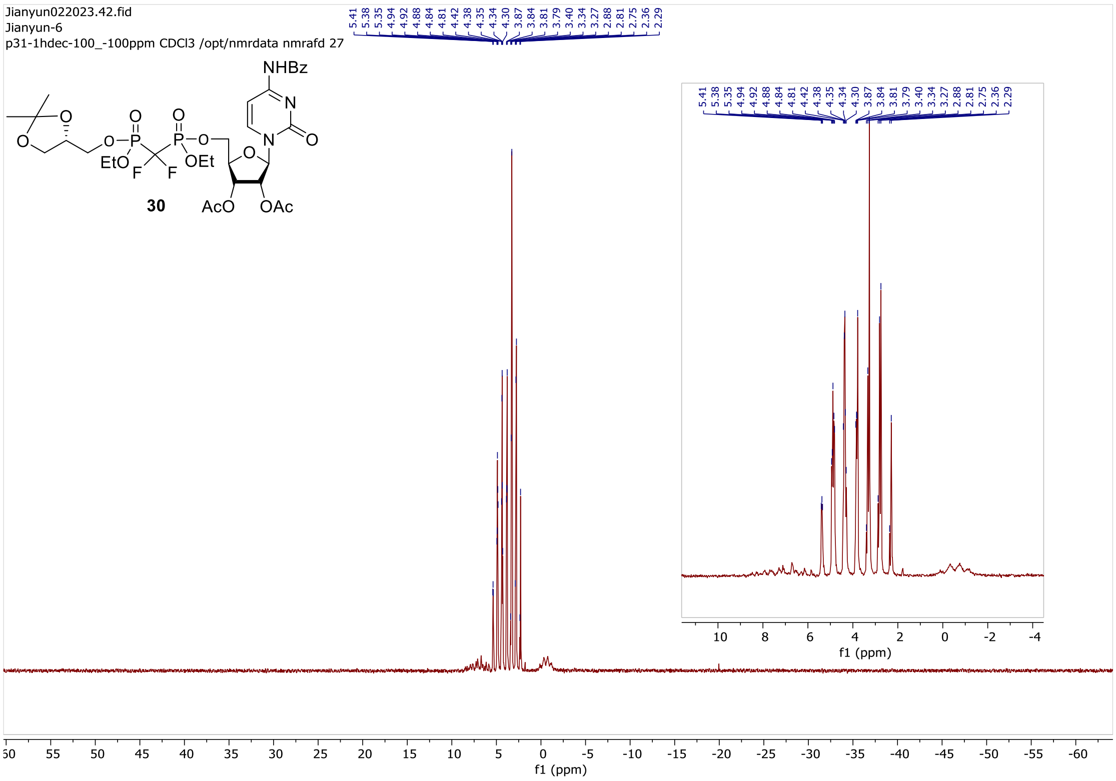

^{31}P NMR (162 MHz, $CDCl_3$) of compound **30**

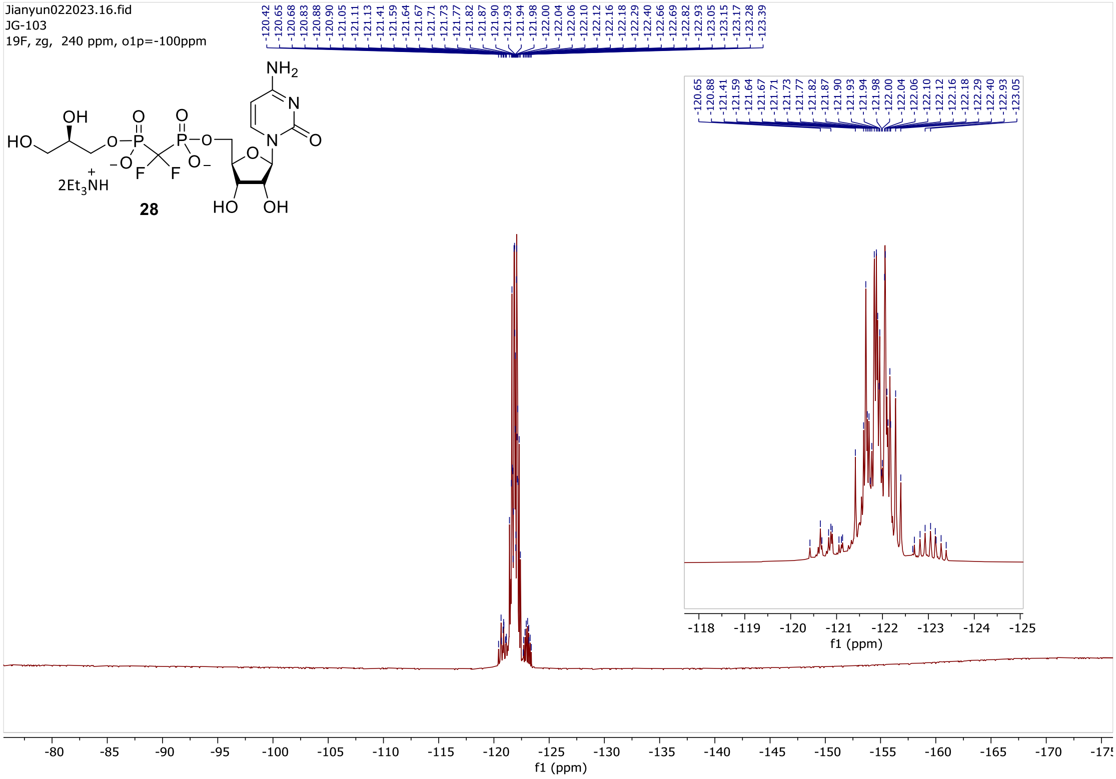

^{19}F NMR (376 MHz, $CDCl_3$) of compound **30**

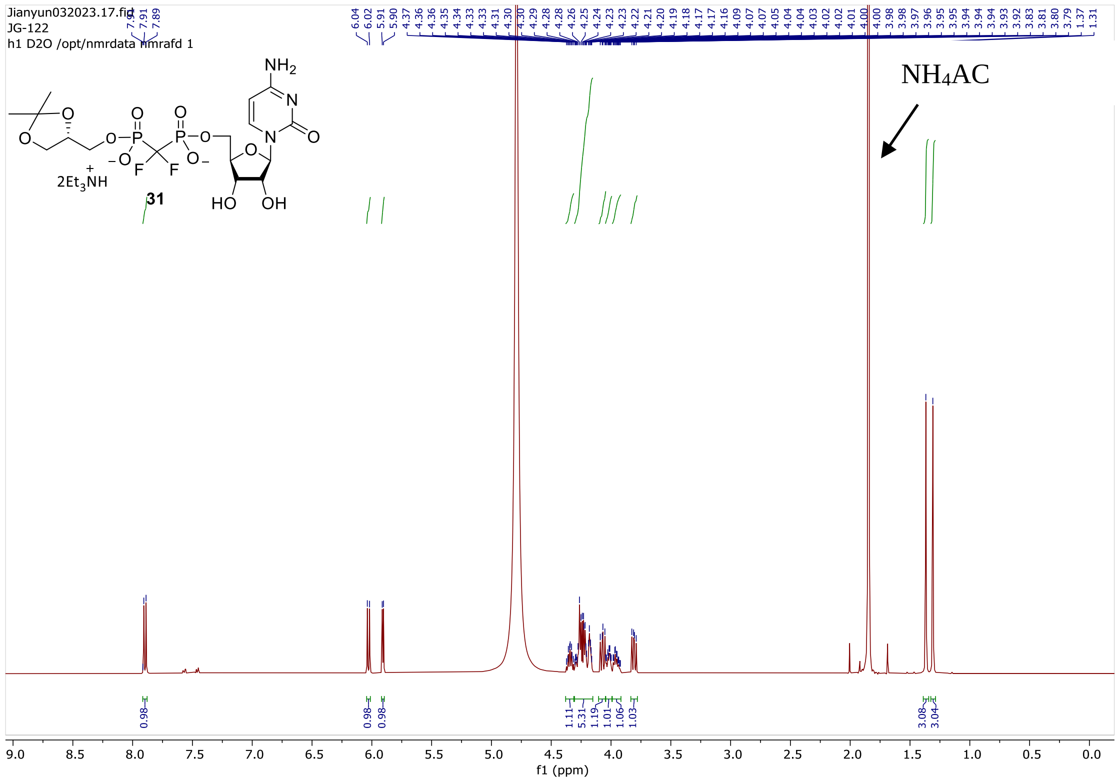

^{1}H NMR (400 MHz, $CDCl_3$) of compound **31**

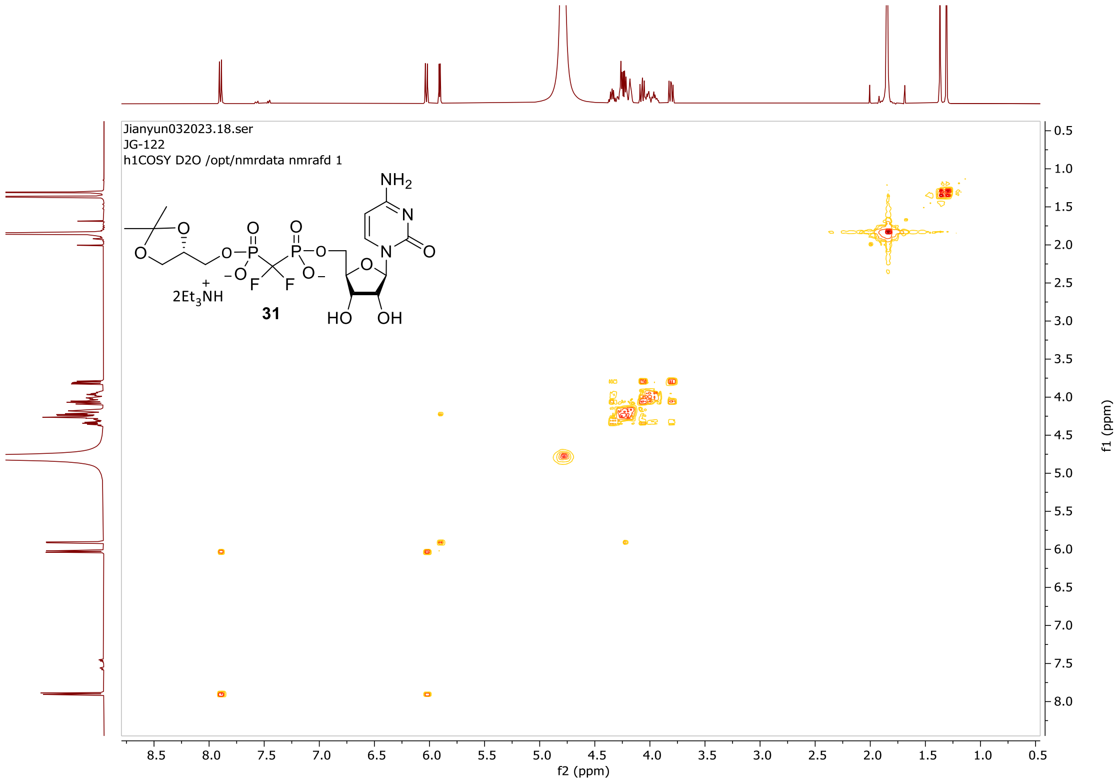

^{1}H - ^{1}H COSY (101 MHz, $CDCl_3$) of compound **31**

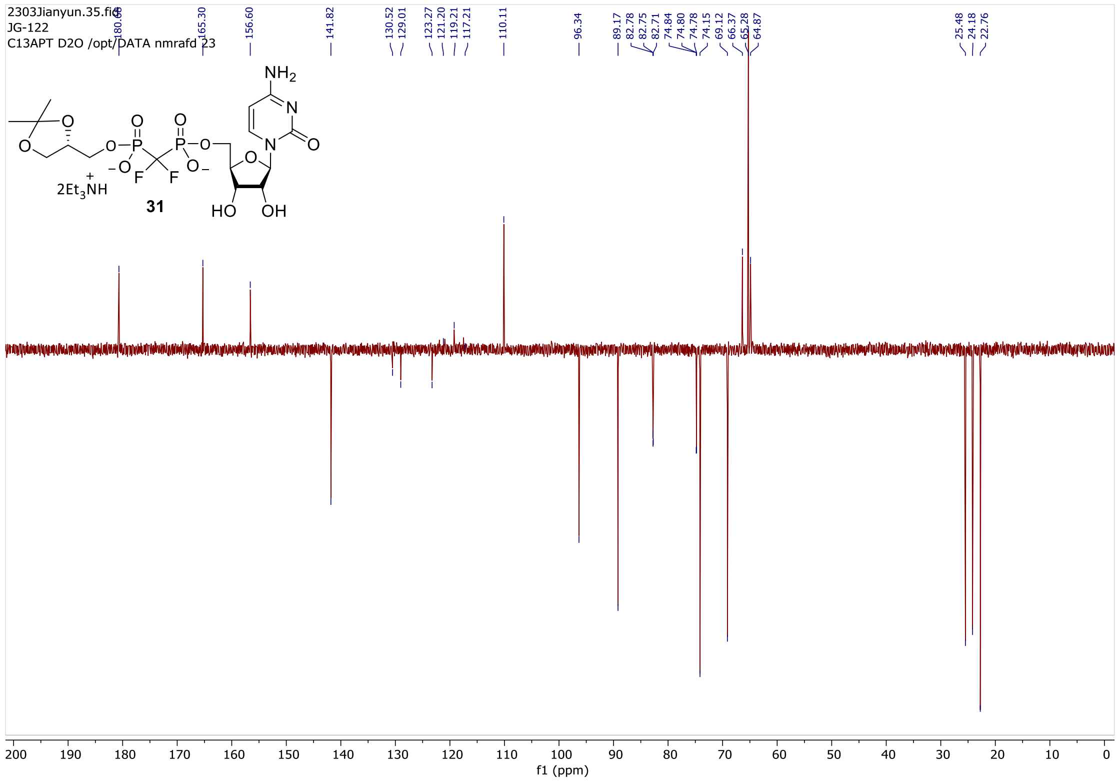

^{13}C NMR (101 MHz, $CDCl_3$) of compound **31**

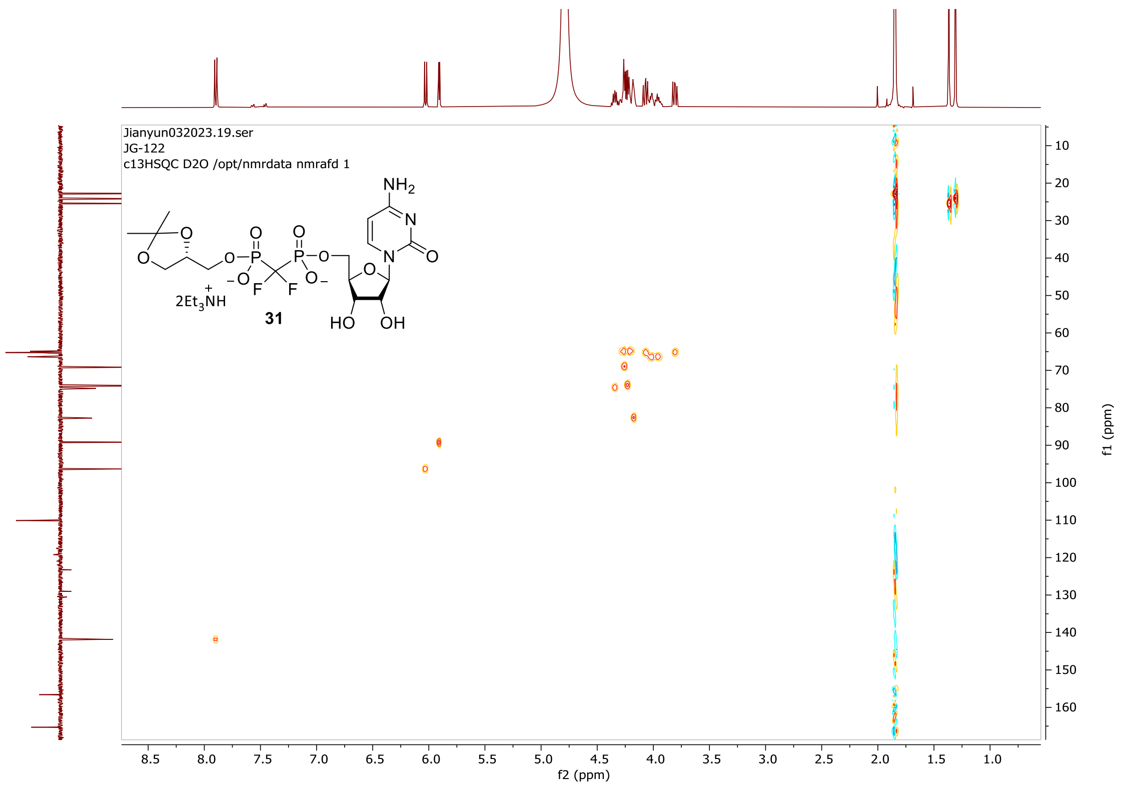

^{1}H - ^{13}C HSQC (400 MHz, $CDCl_3$) of compound **31**

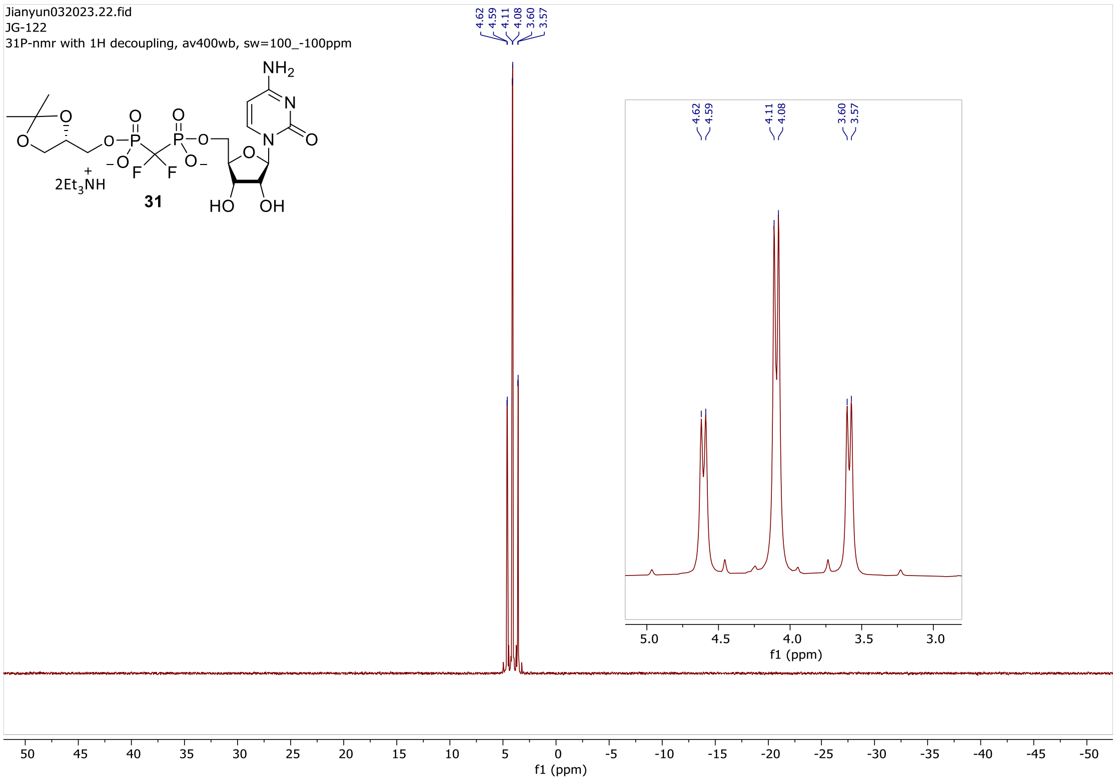

^{31}P NMR (162 MHz, $CDCl_3$) of compound **31**

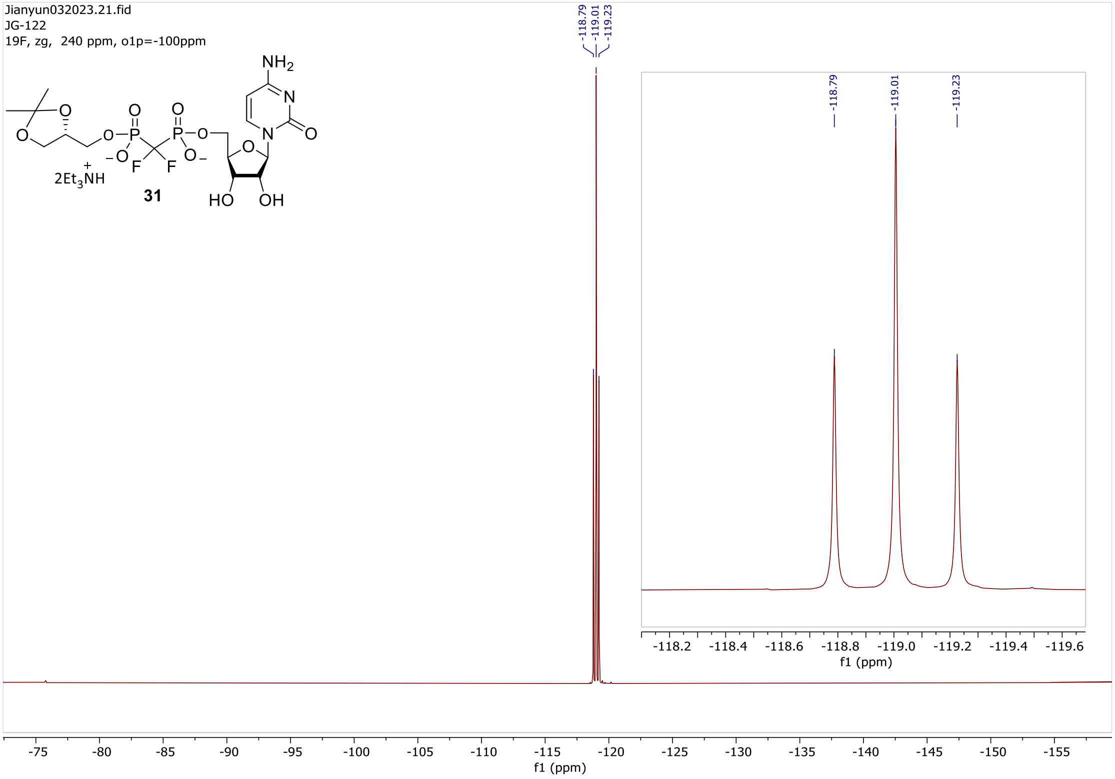

^{19}F NMR (376 MHz, $CDCl_3$) of compound **31**

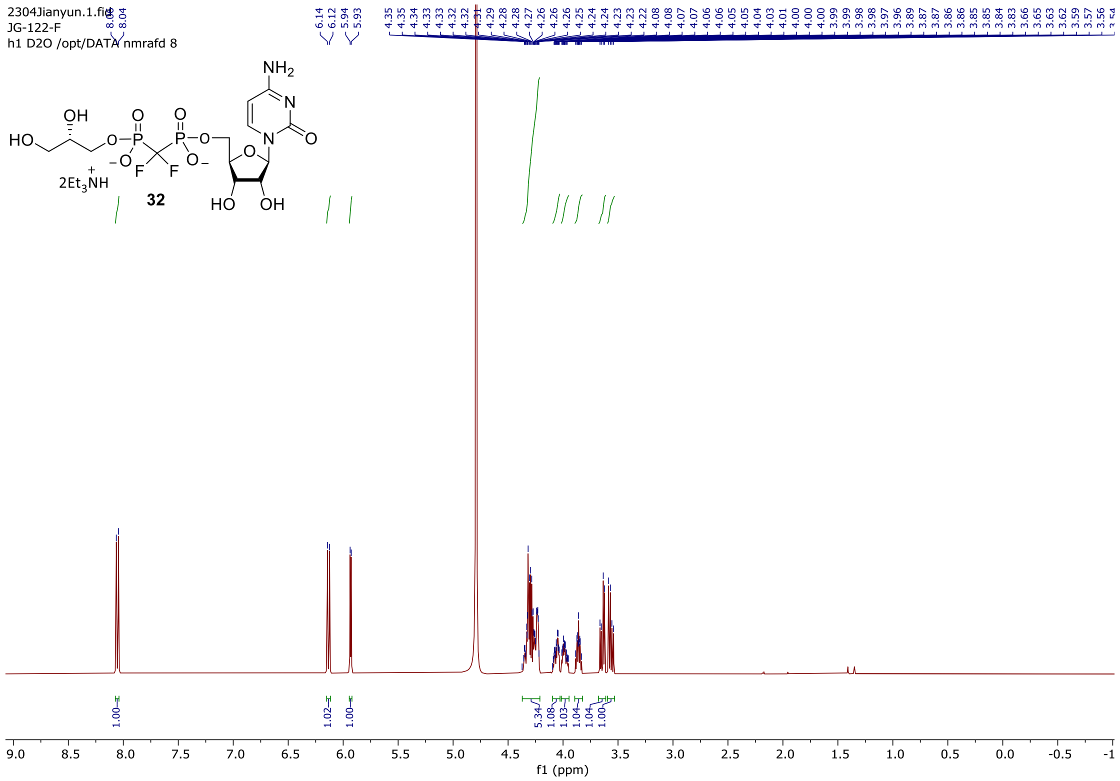

^{1}H NMR (400 MHz, $CDCl_3$) of compound **32**

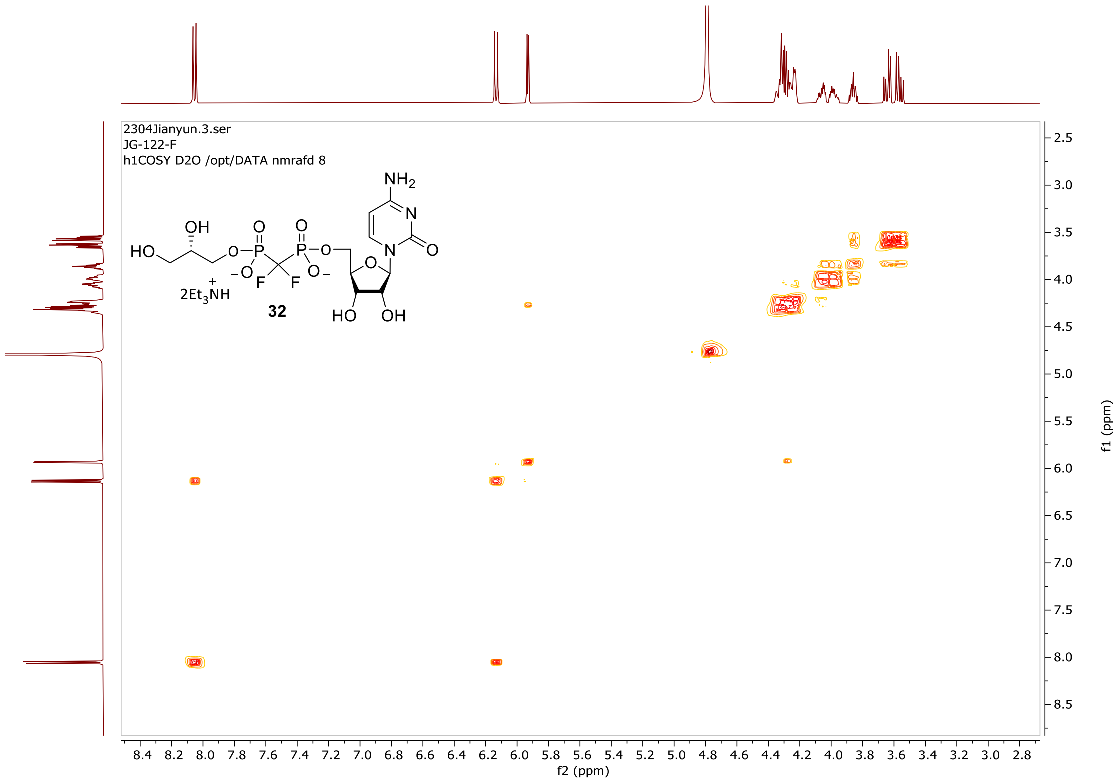

^{1}H - ^{1}H COSY (101 MHz, $CDCl_3$) of compound **32**

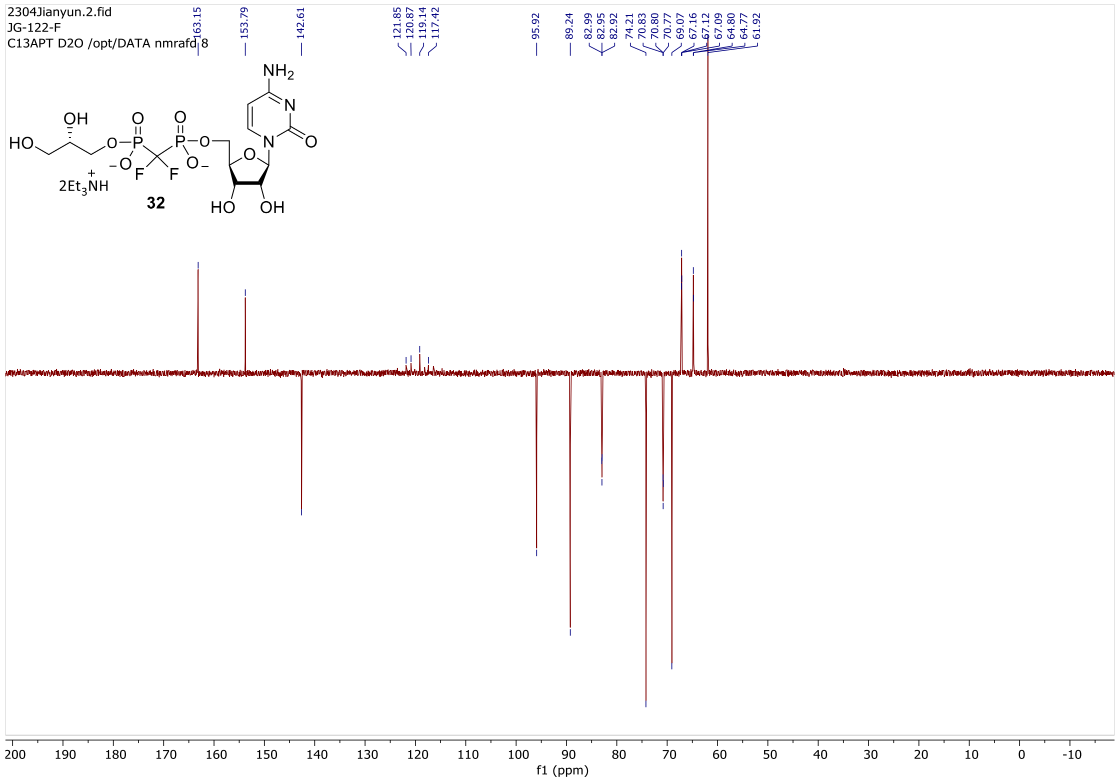

^{13}C NMR (101 MHz, $CDCl_3$) of compound **32**

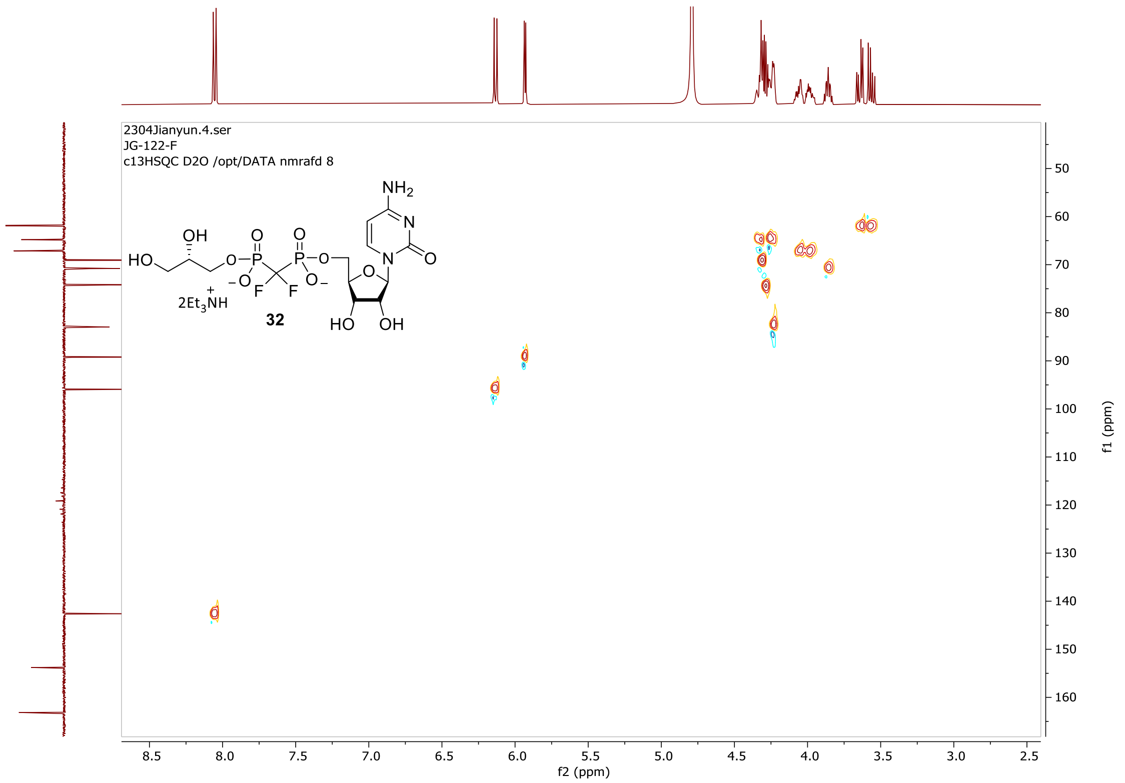

^{1}H - ^{13}C HSQC (400 MHz, $CDCl_3$) of compound **32**

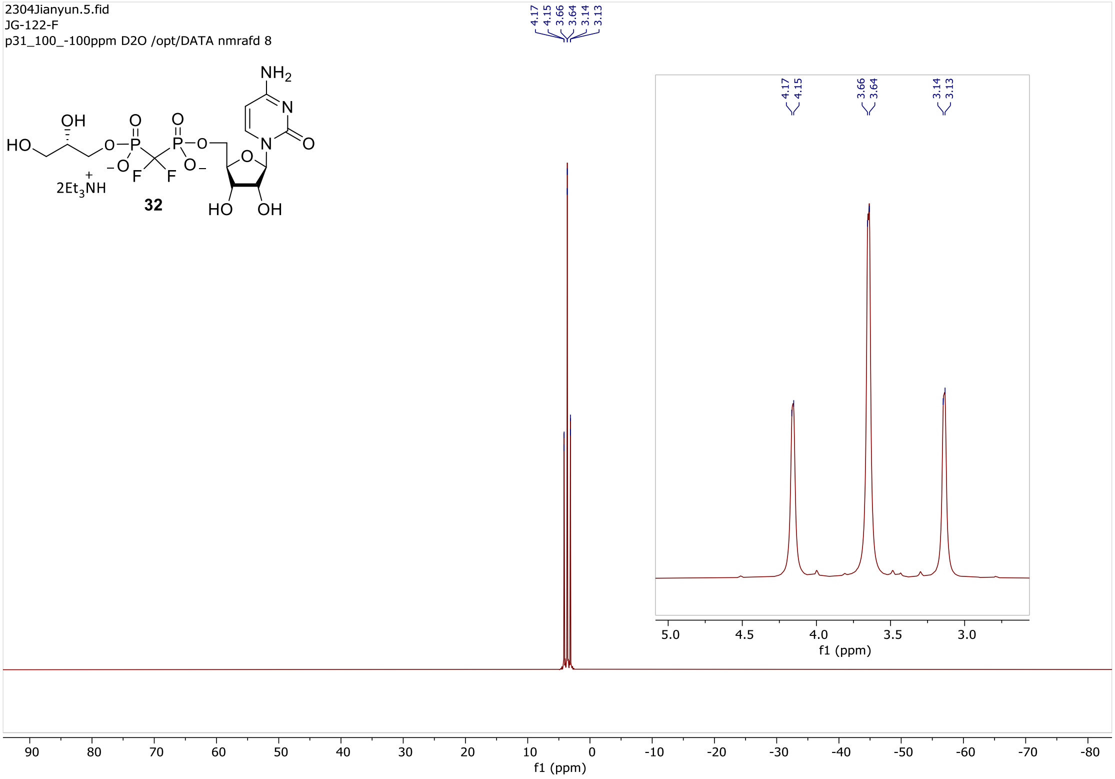

^{31}P NMR (162 MHz, $CDCl_3$) of compound **32**

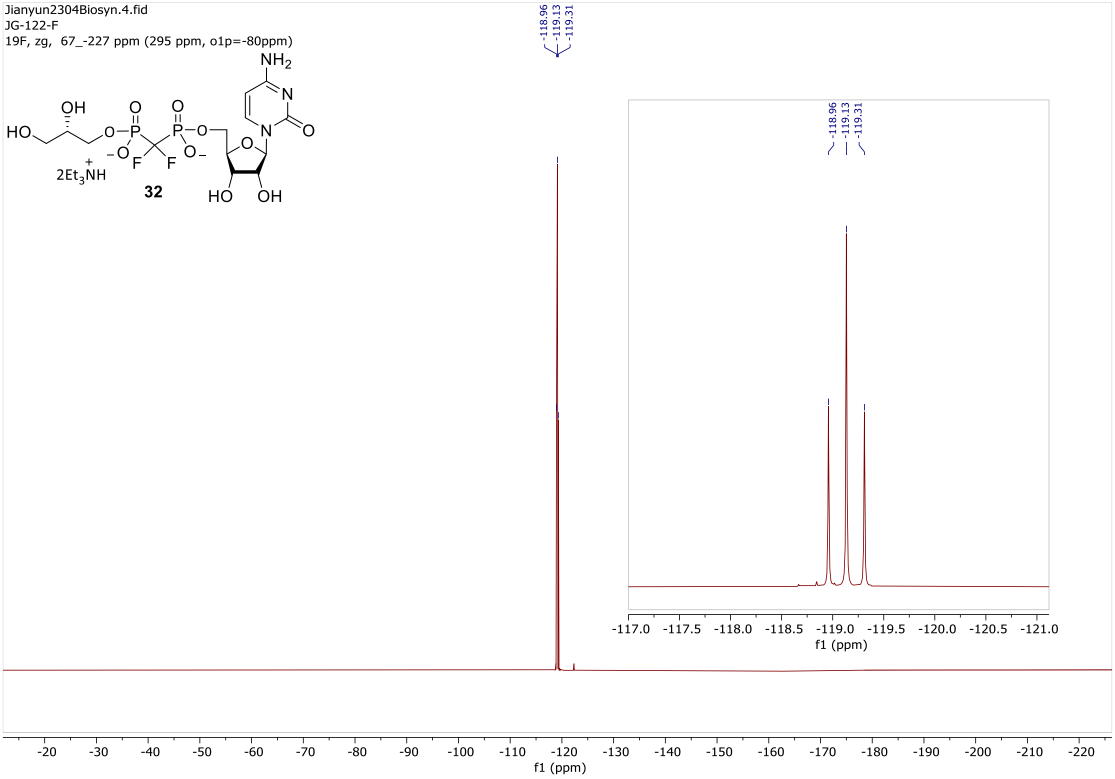

^{19}F NMR (471 MHz, $CDCl_3$) of compound **32**

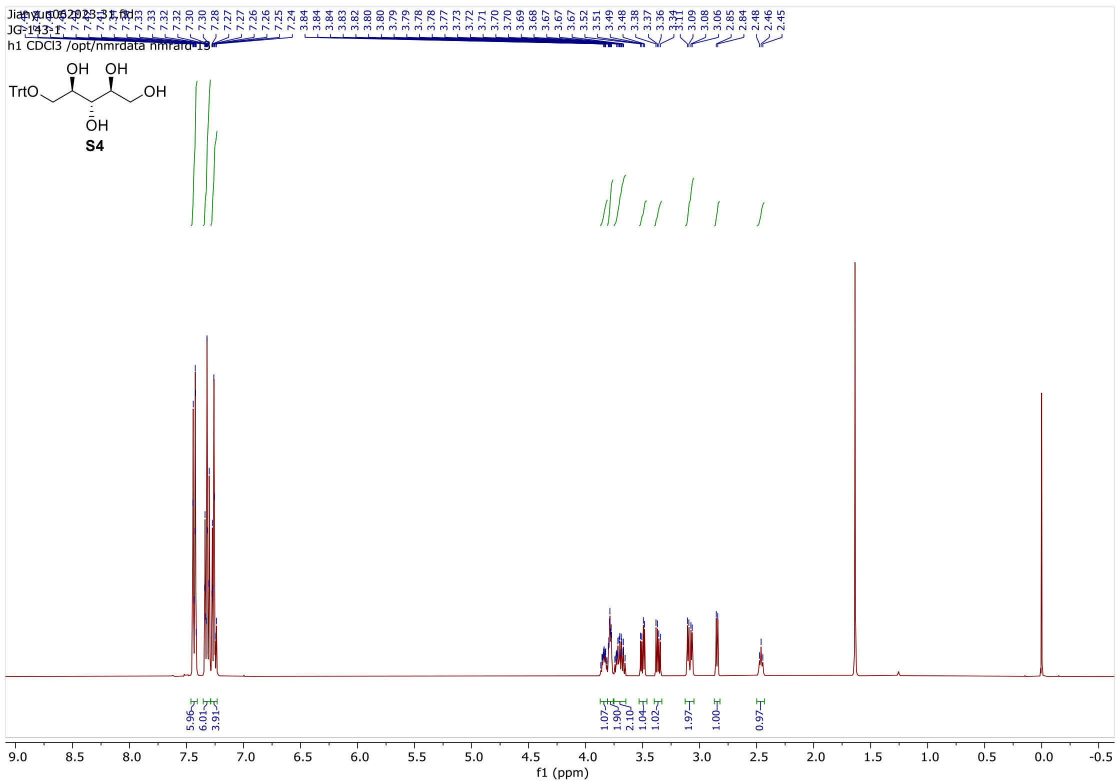

^{1}H NMR (400 MHz, $CDCl_3$) of compound **S4**

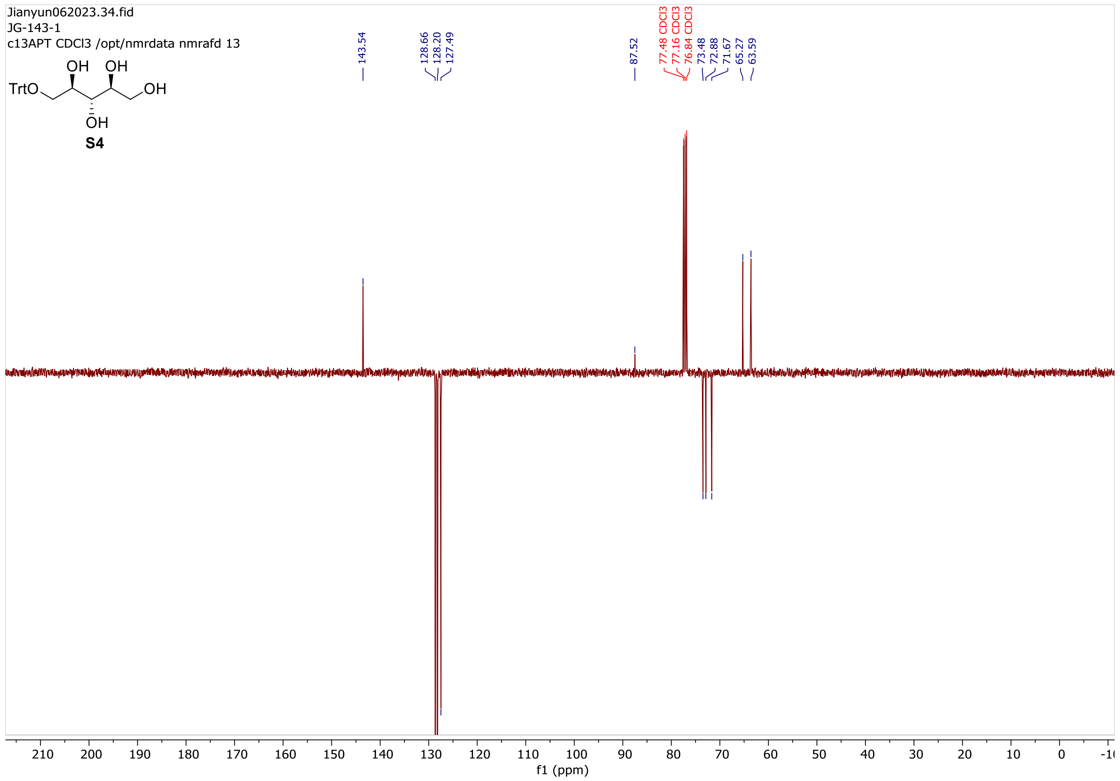

^{13}C NMR (101 MHz, $CDCl_3$) of compound **S4**

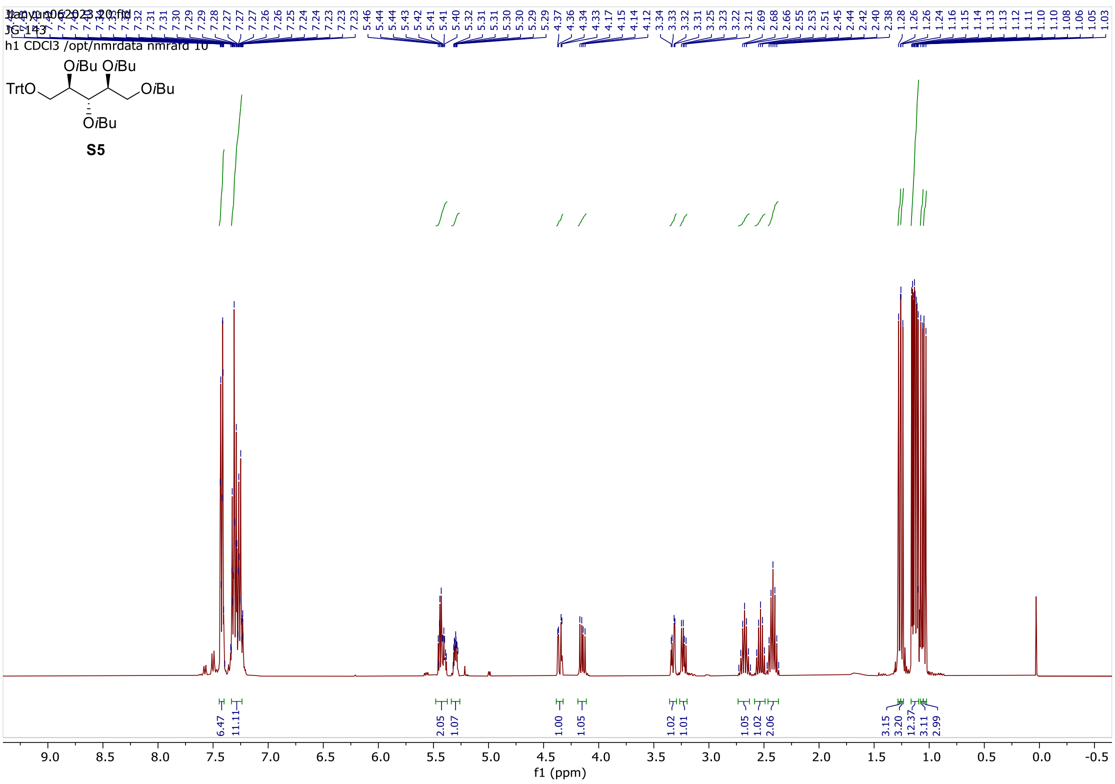

^{1}H NMR (400 MHz, CDCl$_3$) of compound **S5**

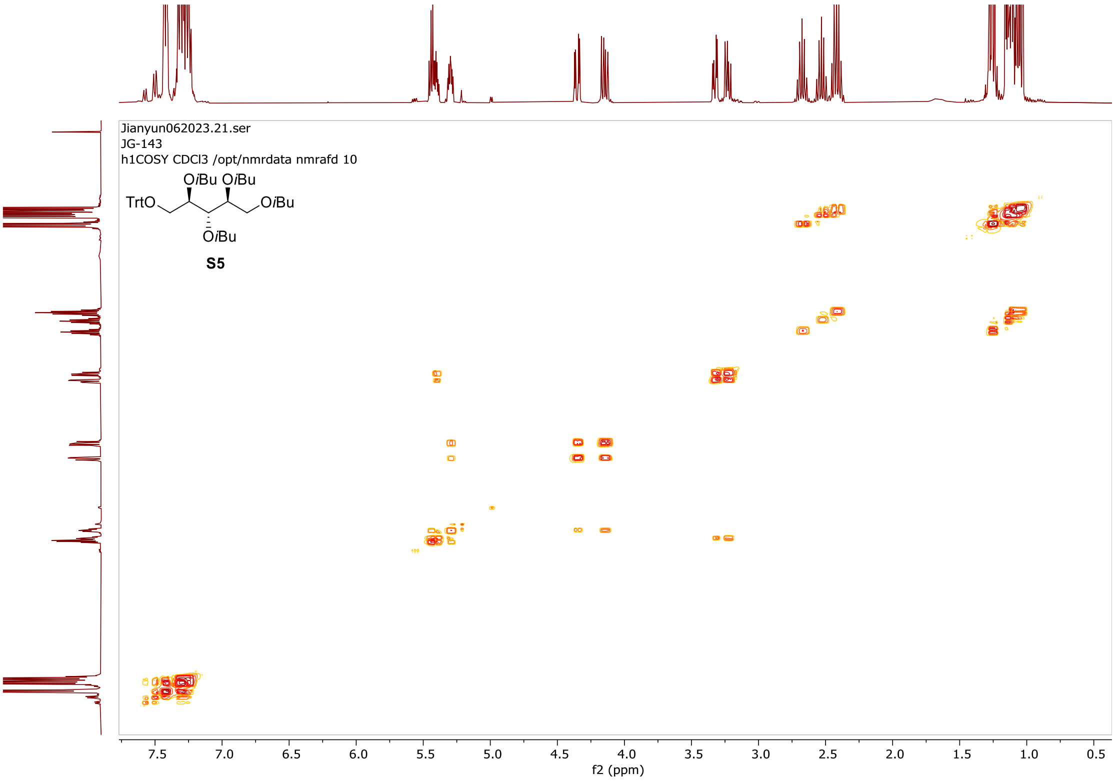

^{1}H - ^{1}H COSY (101 MHz, CDCl$_3$) of compound **S5**

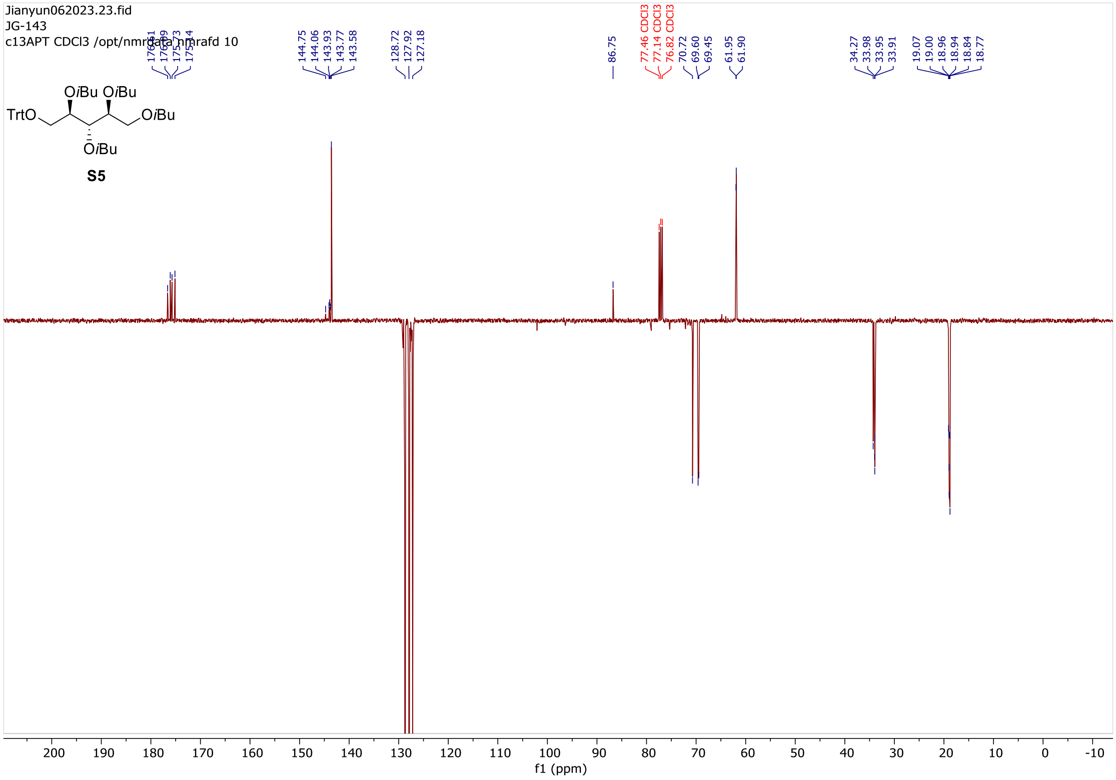

^{13}C NMR (101 MHz, $CDCl_3$) of compound **S5**

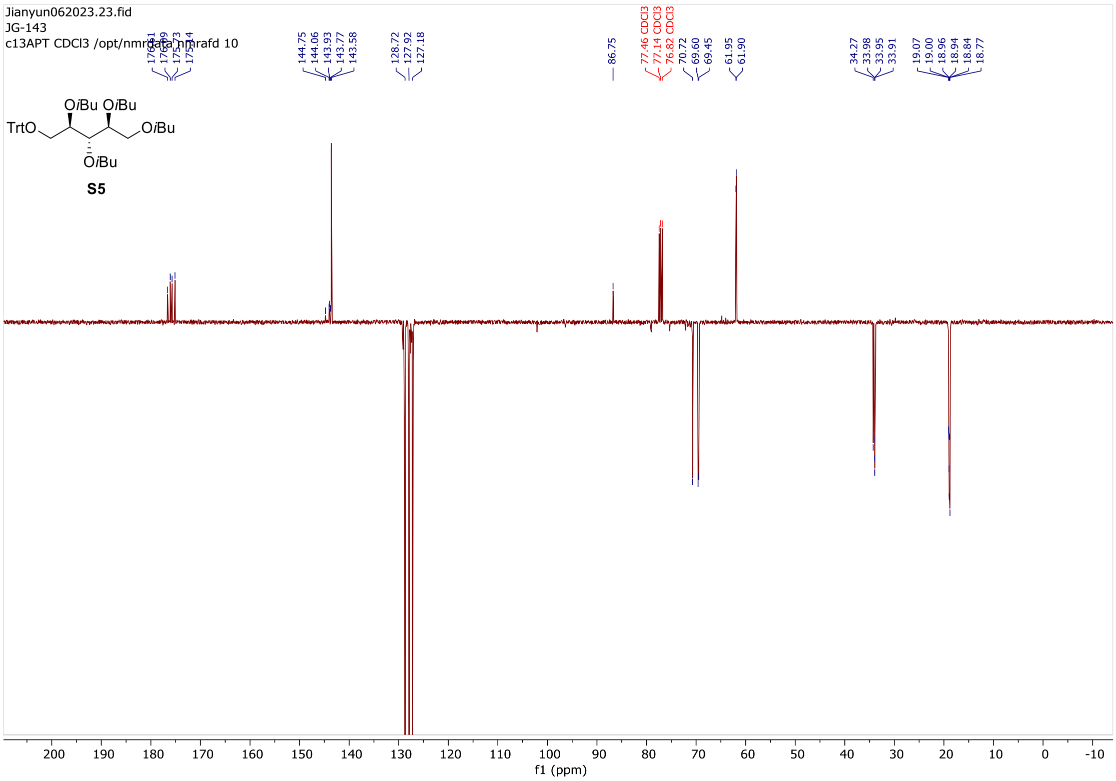

^{1}H - ^{13}C HSQC (400 MHz, $CDCl_3$) of compound **S5**

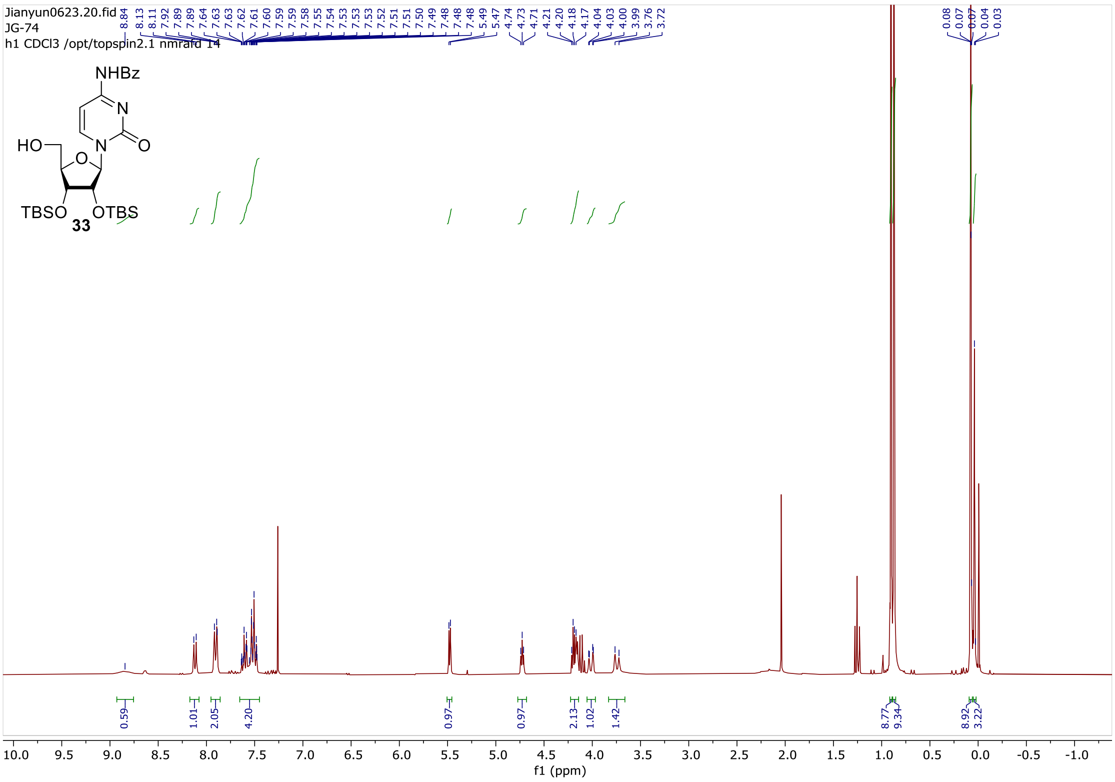

^{1}H NMR (400 MHz, $CDCl_3$) of compound **33**

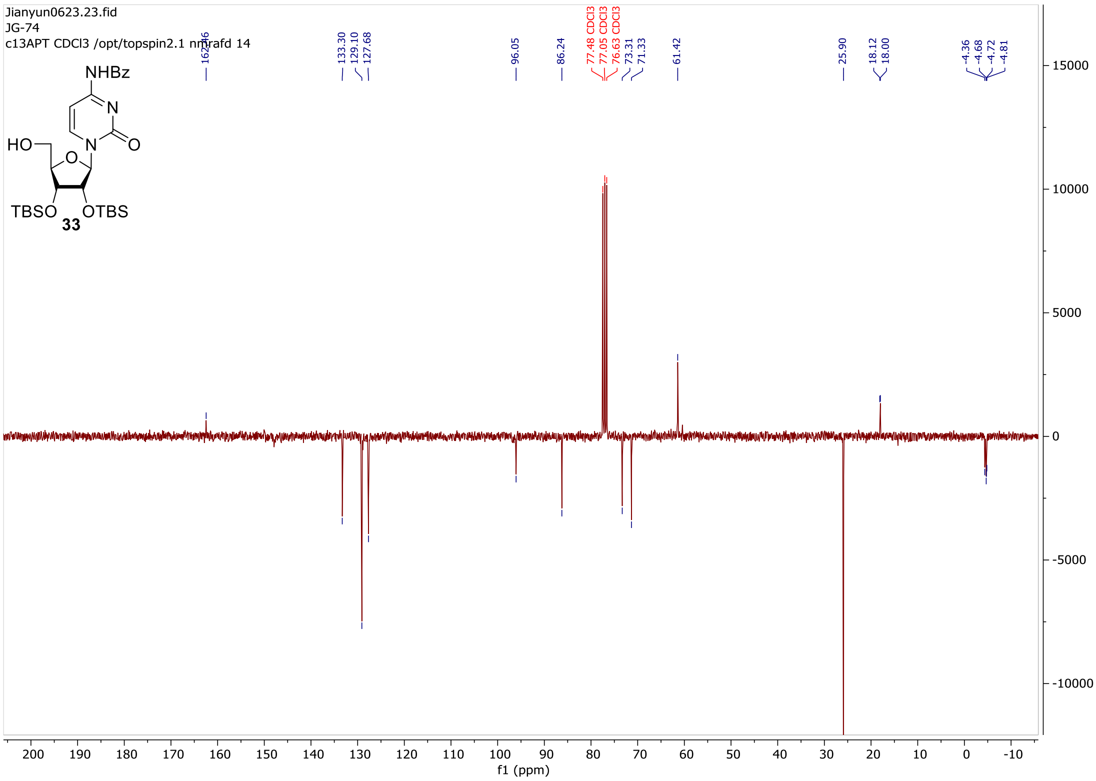

^{13}C NMR (101 MHz, $CDCl_3$) of compound **33**

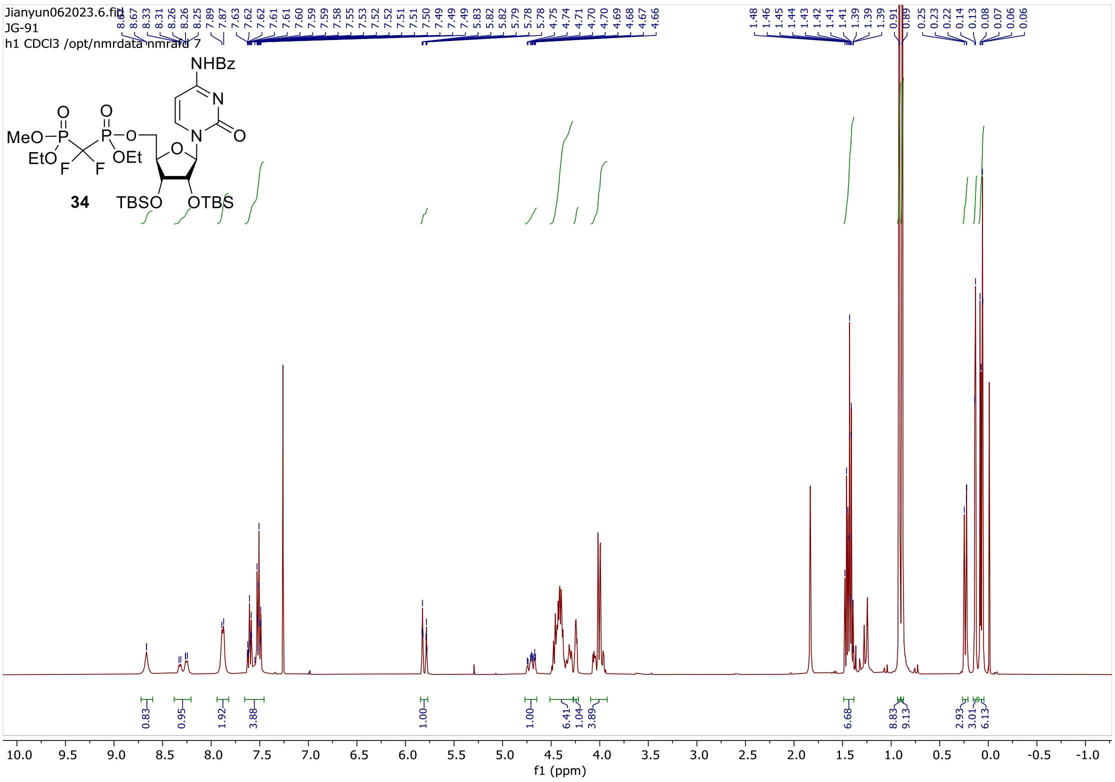

^{1}H NMR (400 MHz, $CDCl_3$) of compound **34**

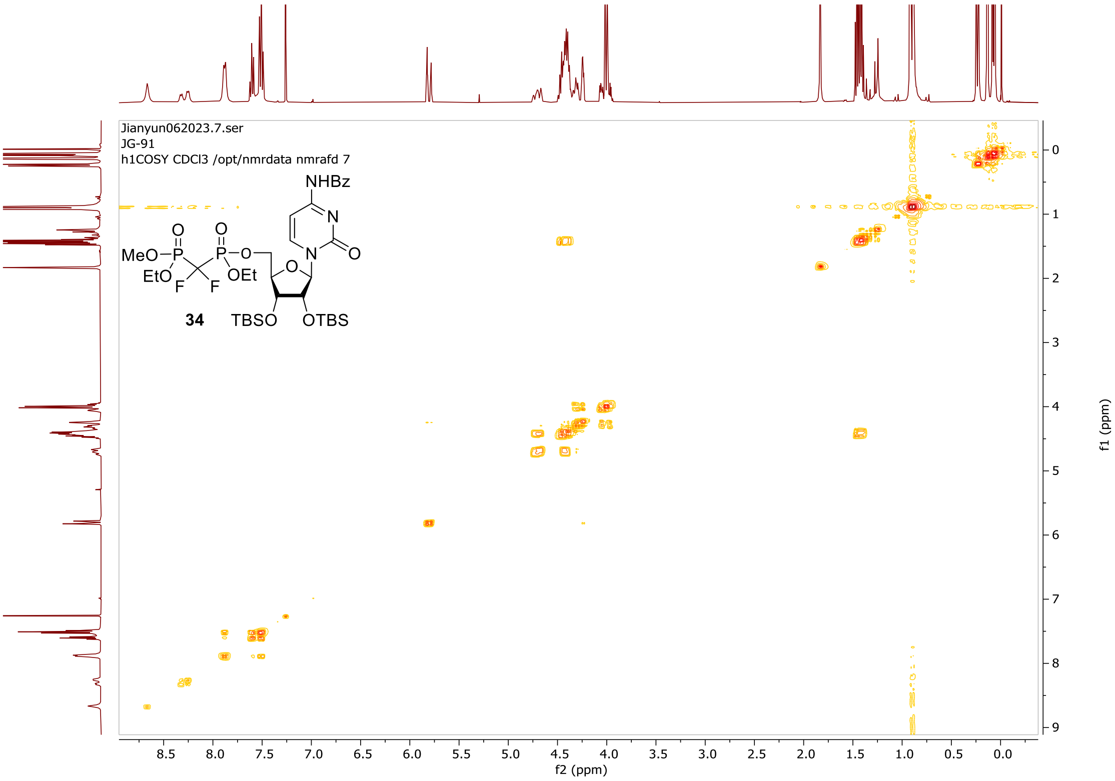

^{1}H - ^{1}H COSY (101 MHz, $CDCl_3$) of compound **34**

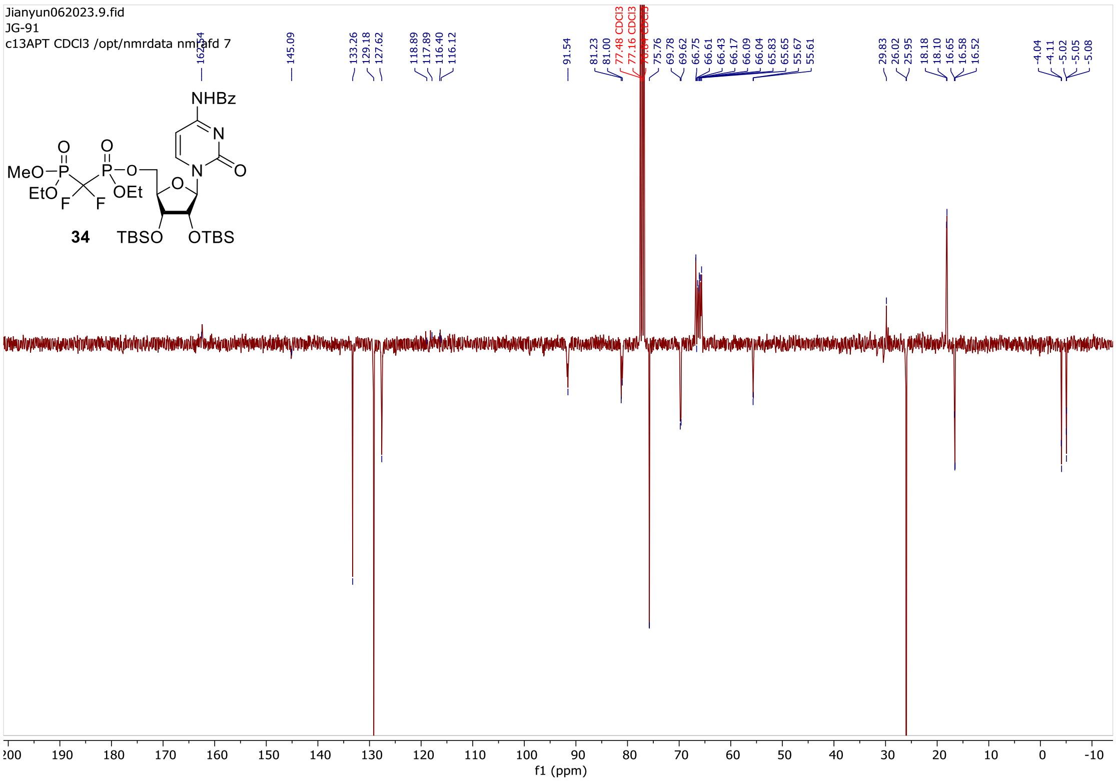

^{13}C NMR (101 MHz, $CDCl_3$) of compound **34**

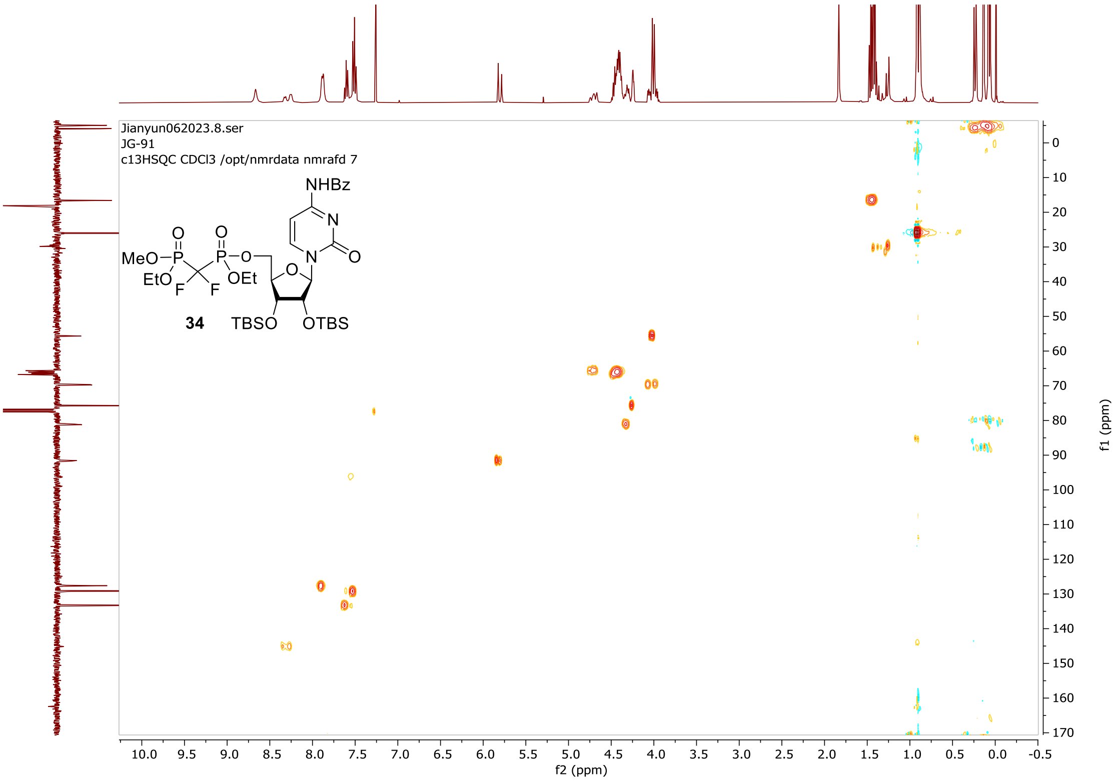

^{1}H - ^{13}C HSQC (400 MHz, $CDCl_3$) of compound **34**

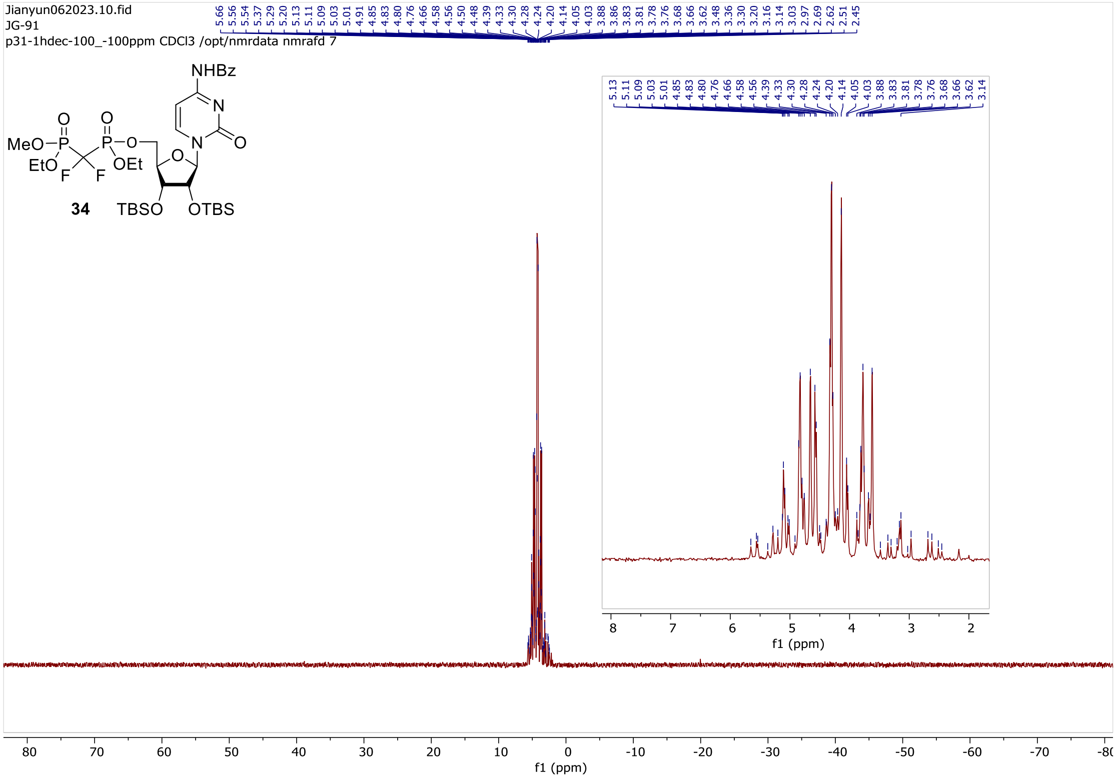

^{31}P NMR (162 MHz, CDCl$_3$) of compound **34**

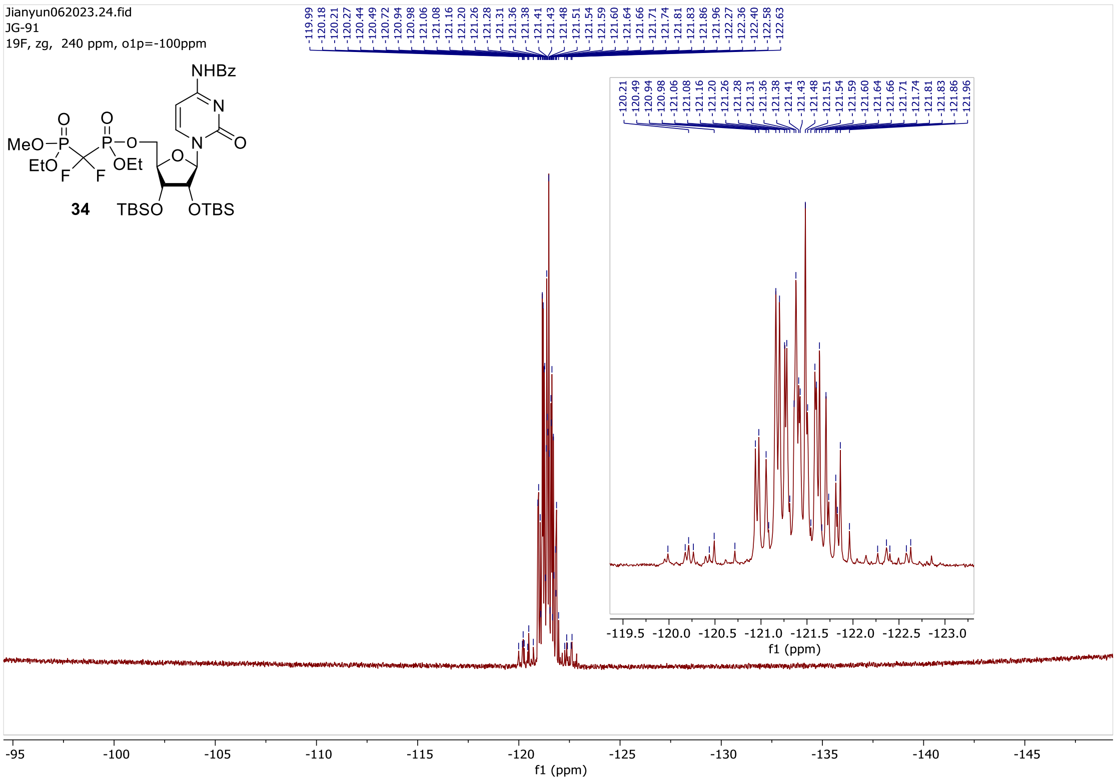

^{19}F NMR (376 MHz, CDCl$_3$) of compound **34**

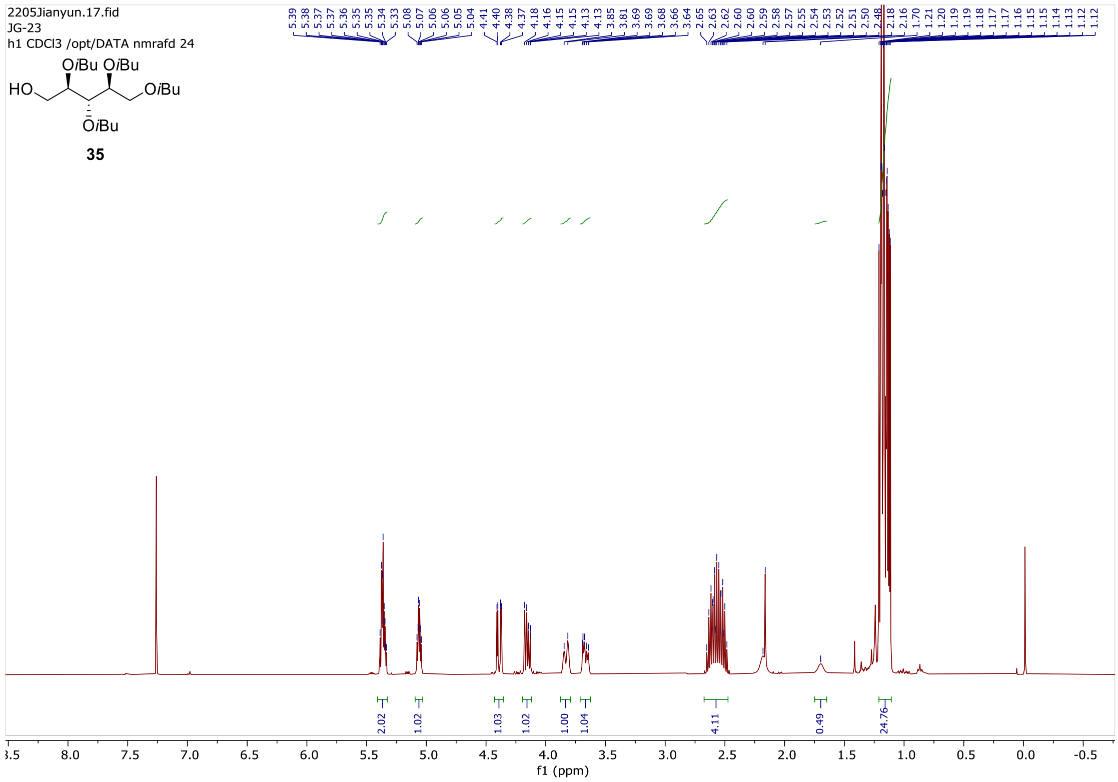

^{1}H NMR (400 MHz, CDCl$_3$) of compound **35**

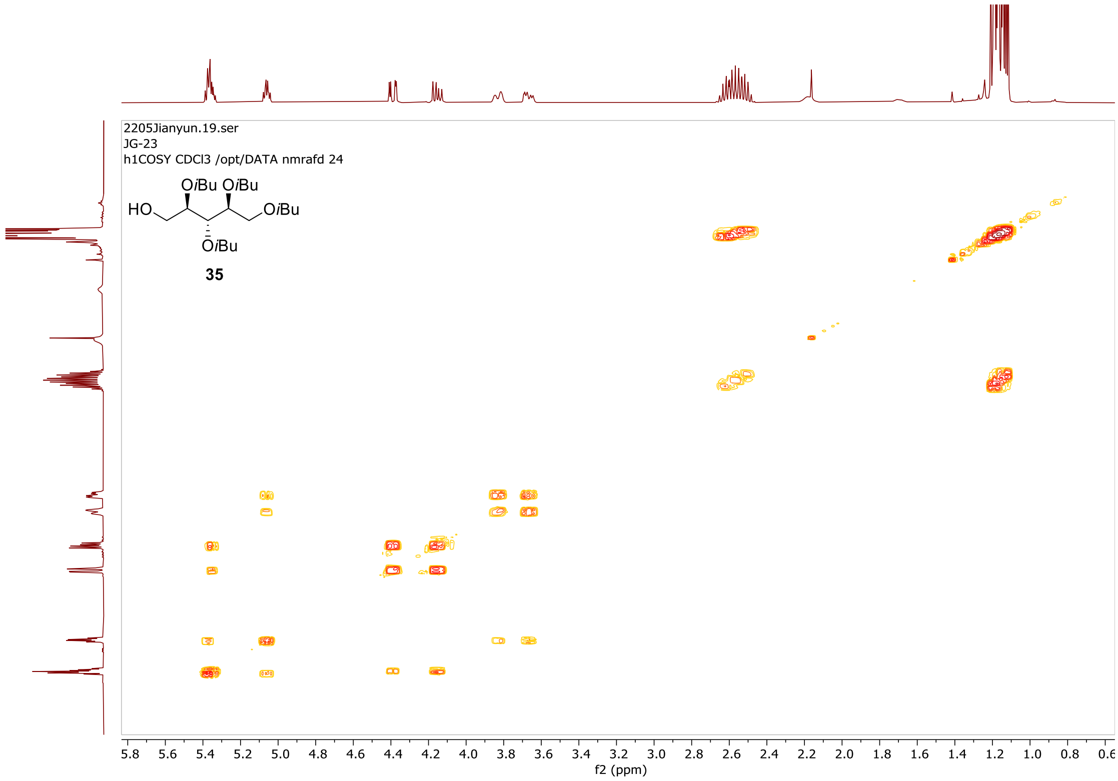

^{1}H - ^{1}H COSY (101 MHz, CDCl$_3$) of compound **35**

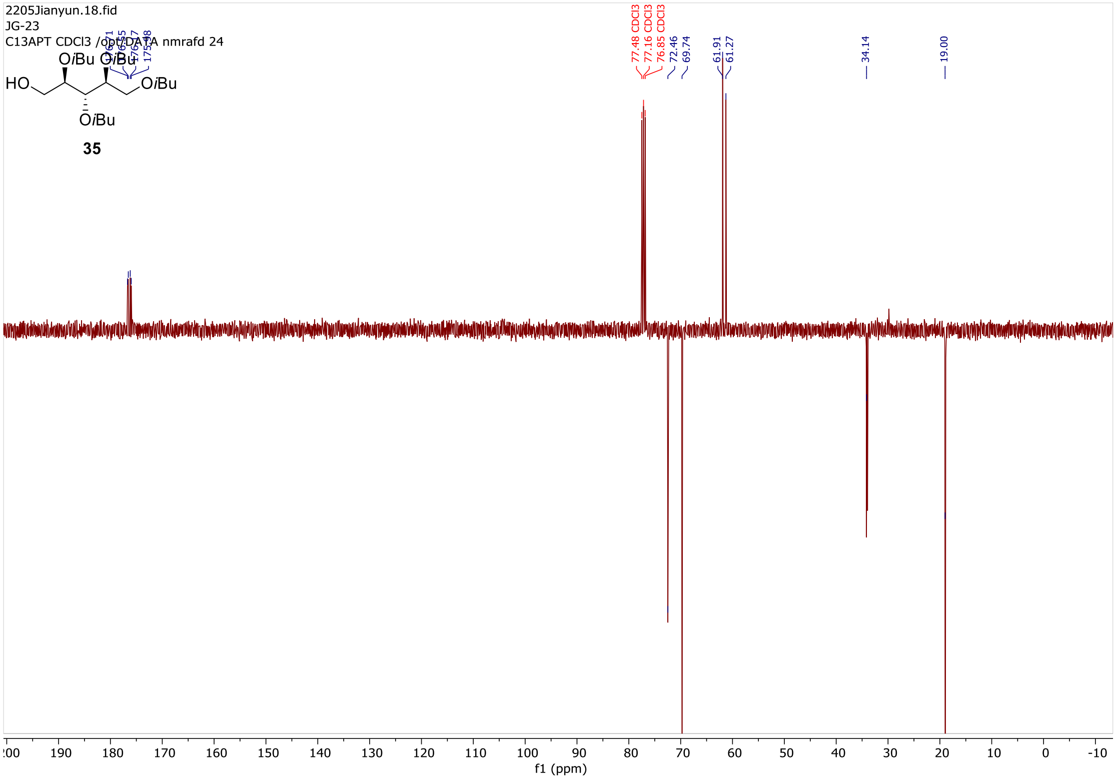

^{13}C NMR (101 MHz, $CDCl_3$) of compound **35**

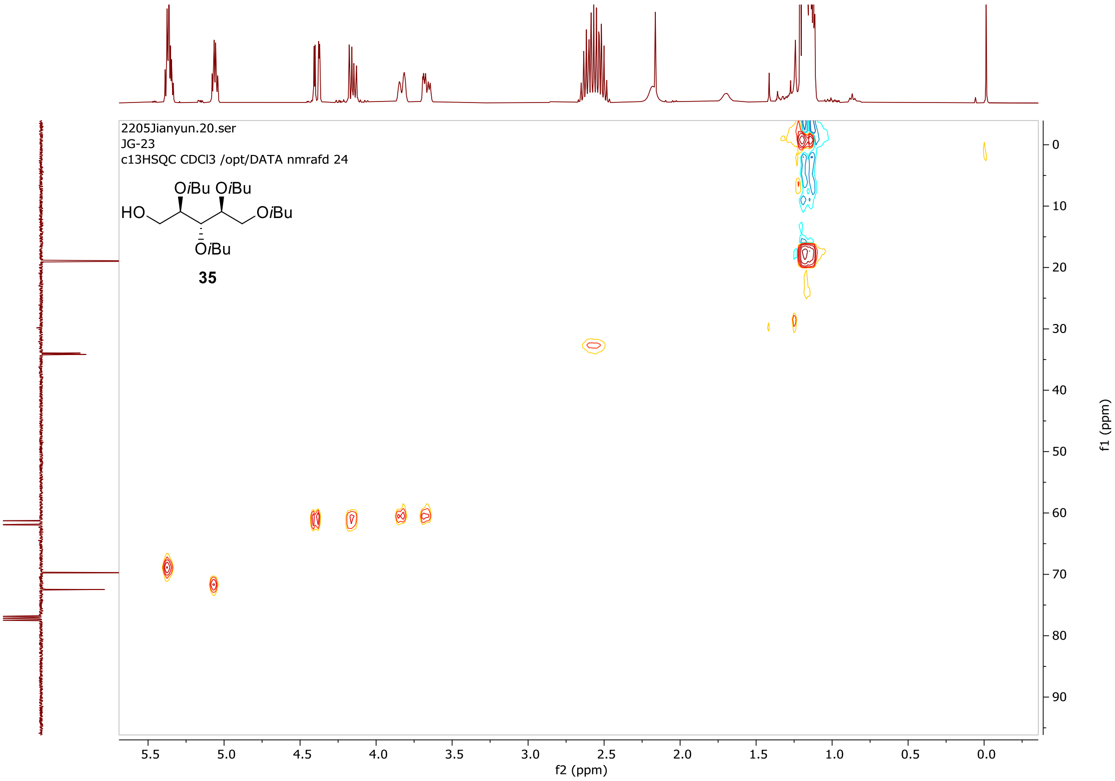

^{1}H - ^{13}C HSQC (400 MHz, $CDCl_3$) of compound **35**

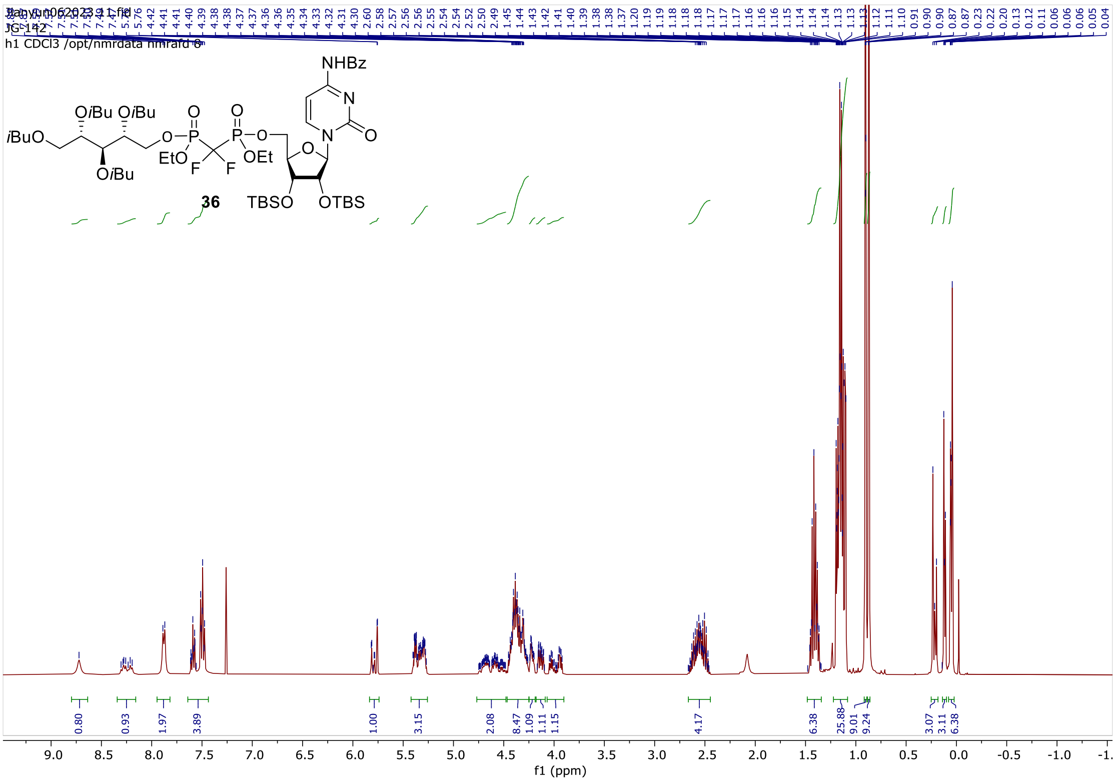

^{1}H NMR (400 MHz, $CDCl_3$) of compound **36**

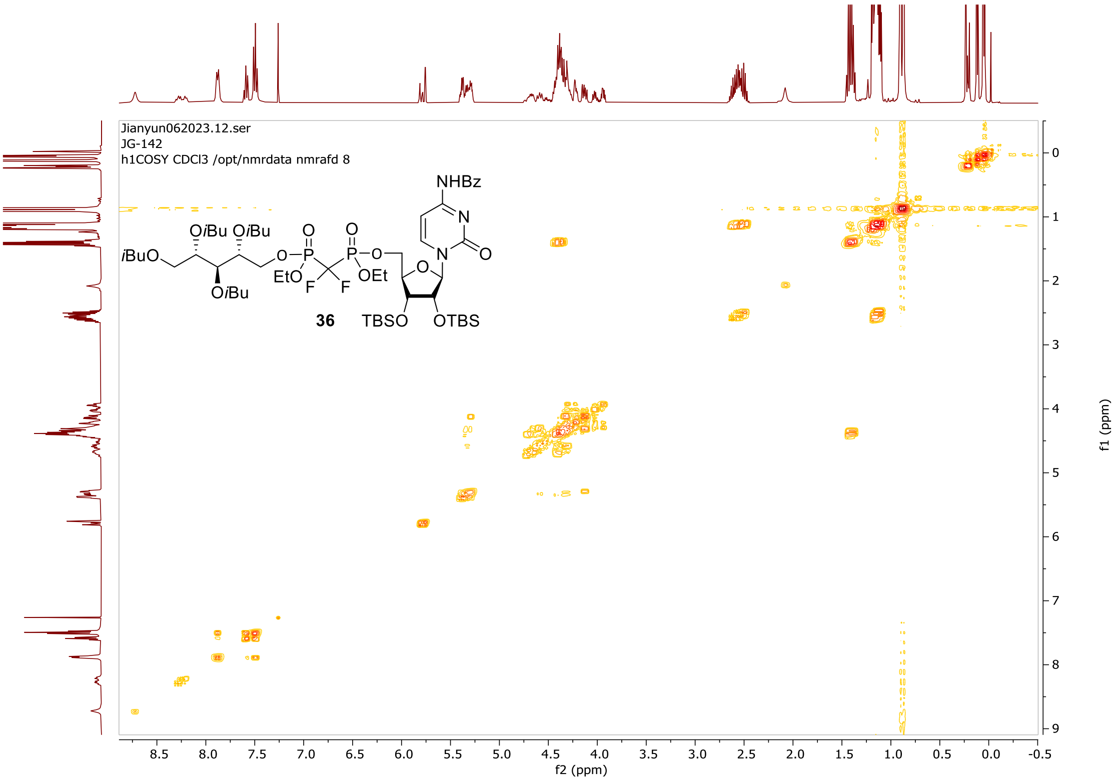

^{1}H - ^{1}H COSY (101 MHz, $CDCl_3$) of compound **36**

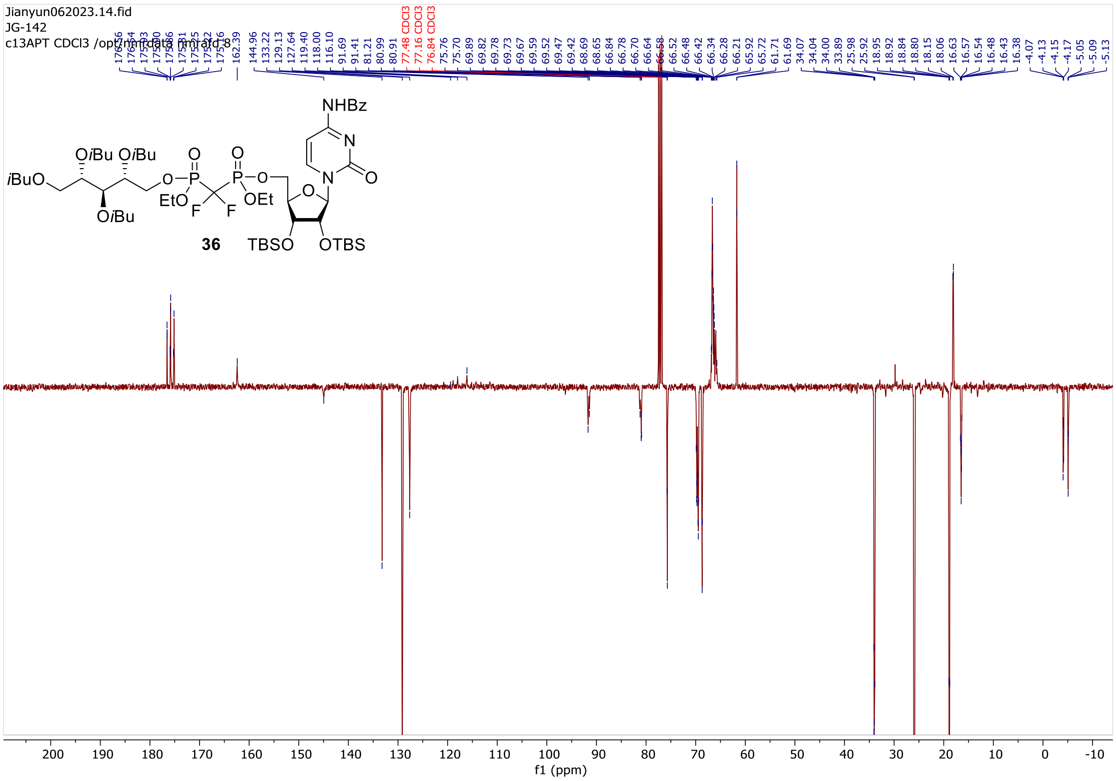

^{13}C NMR (101 MHz, CDCl$_3$) of compound **36**

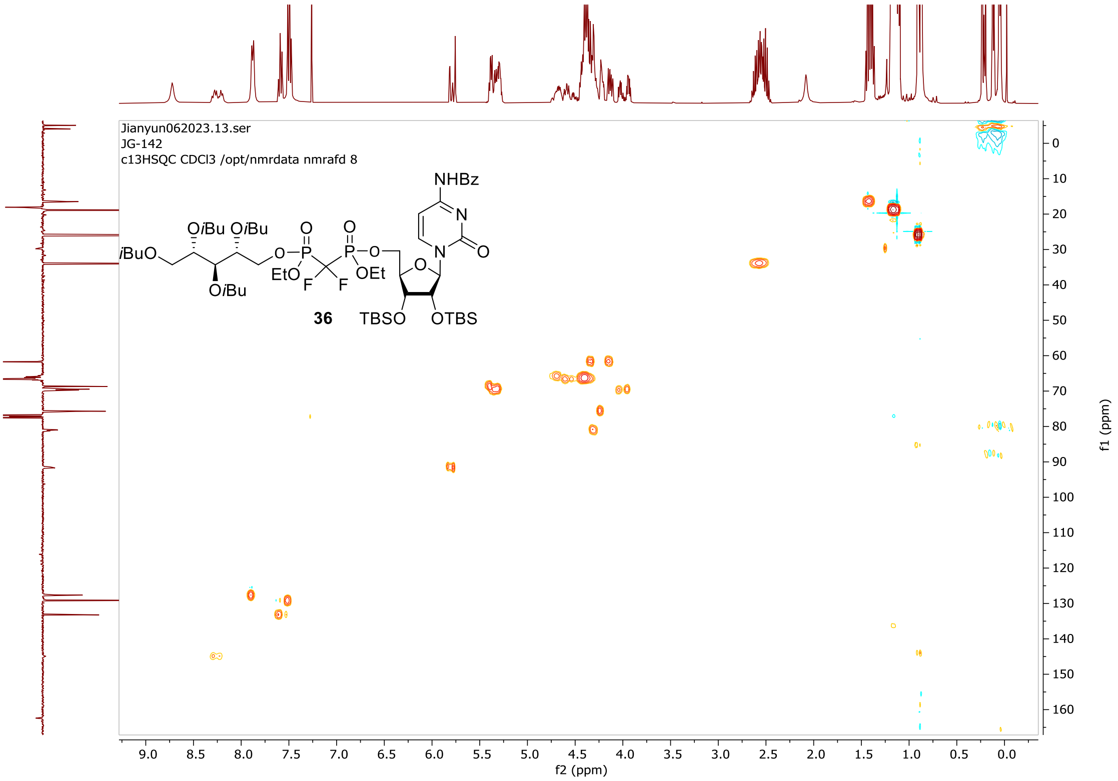

^{1}H - ^{13}C HSQC (400 MHz, CDCl$_3$) of compound **36**

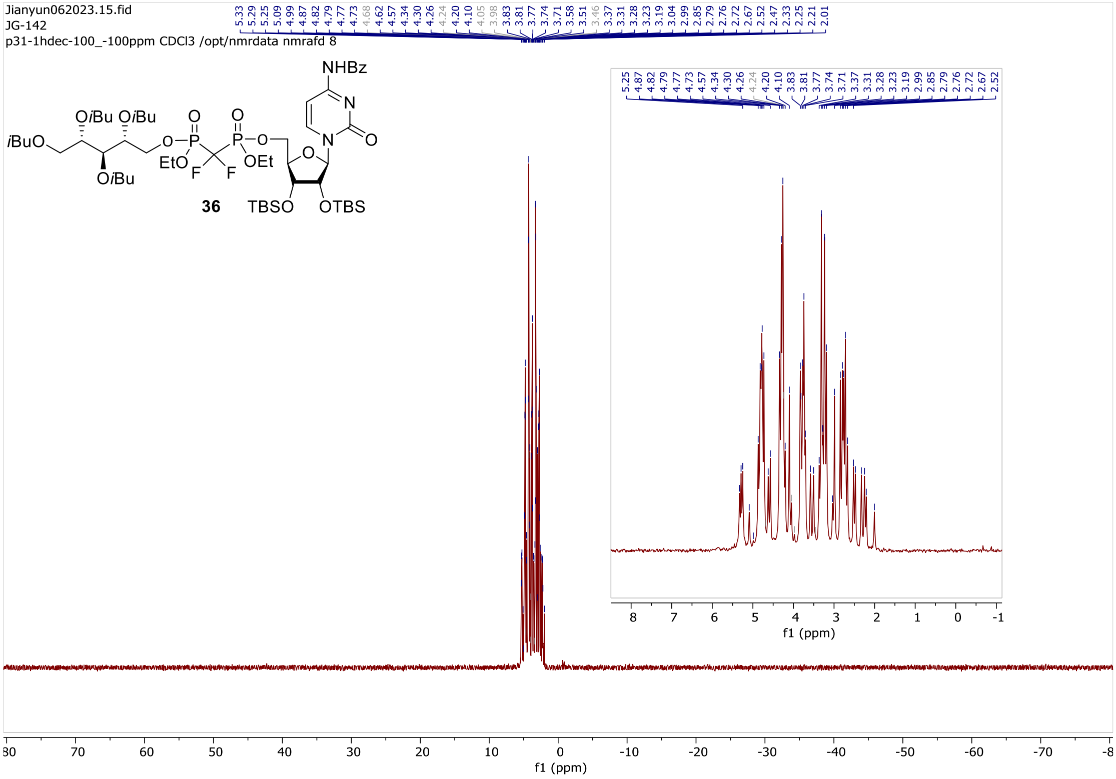

^{31}P NMR (162 MHz, CDCl$_3$) of compound **36**

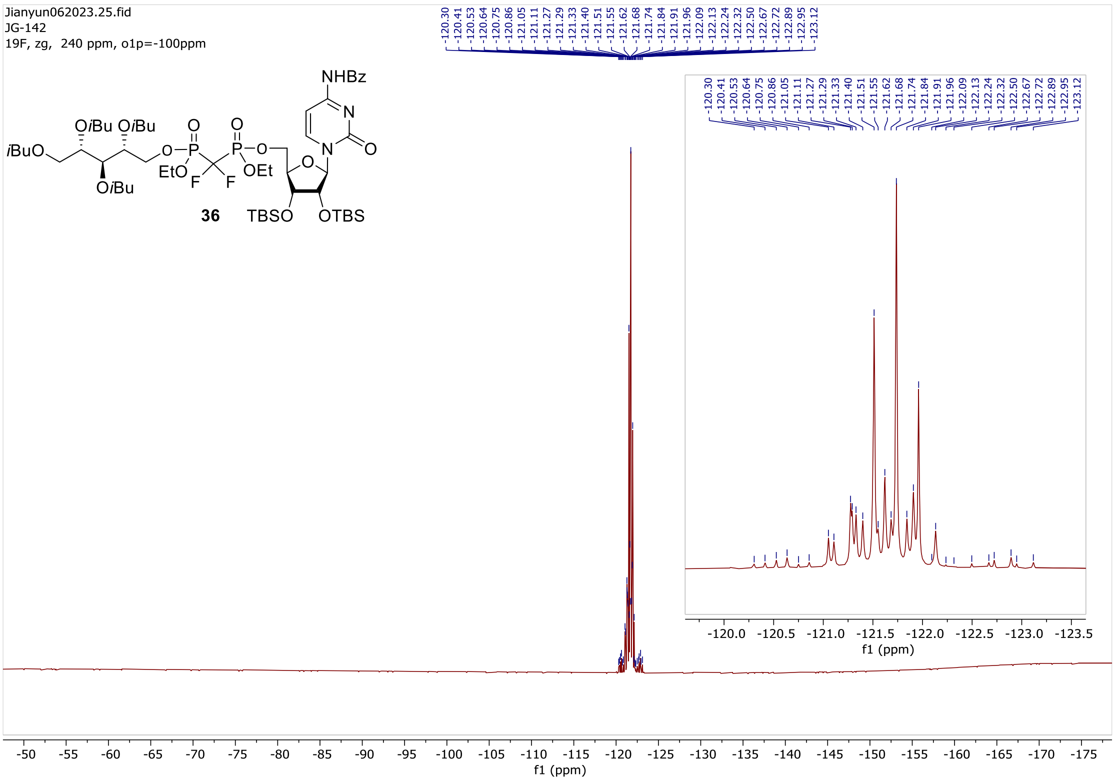

^{19}F NMR (376 MHz, CDCl$_3$) of compound **36**

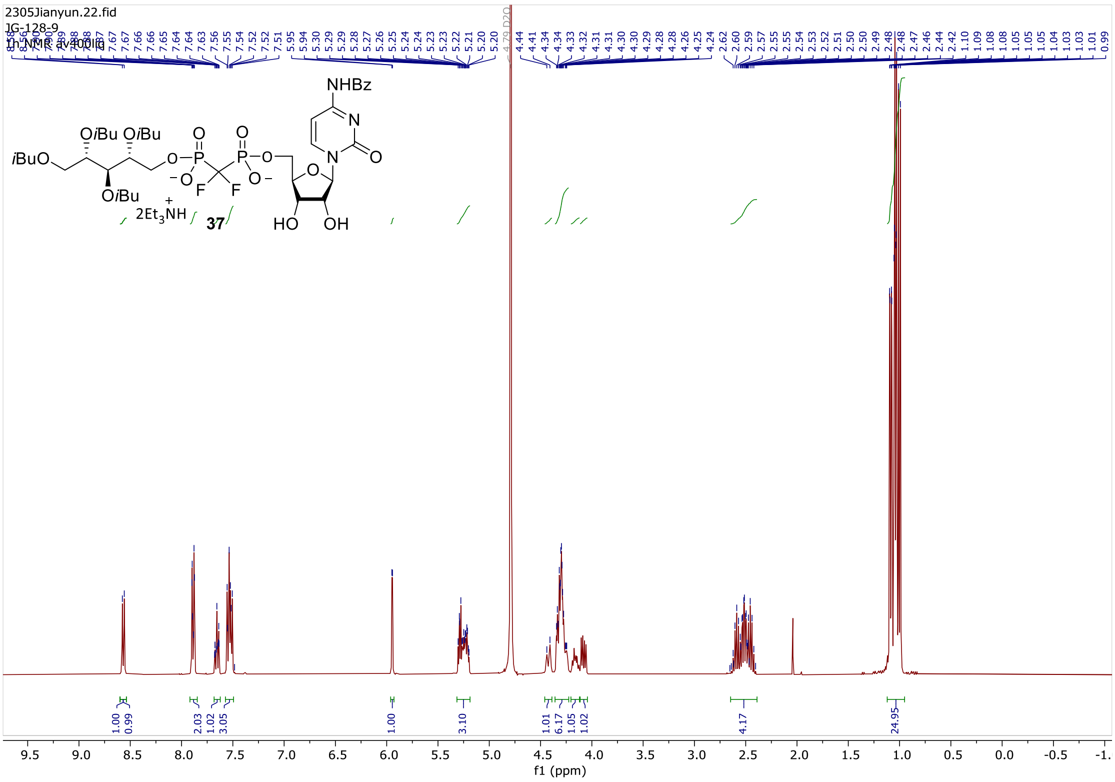

^{1}H NMR (400 MHz, $CDCl_3$) of compound **37**

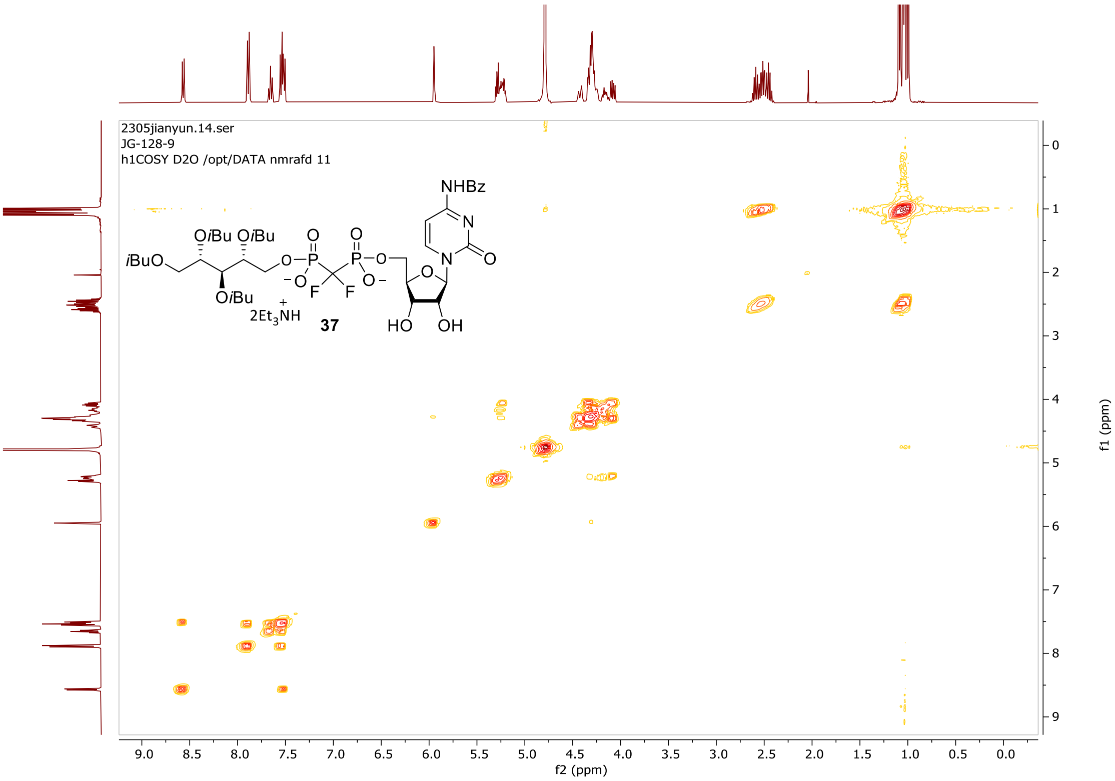

^{1}H - ^{1}H COSY (101 MHz, $CDCl_3$) of compound **37**

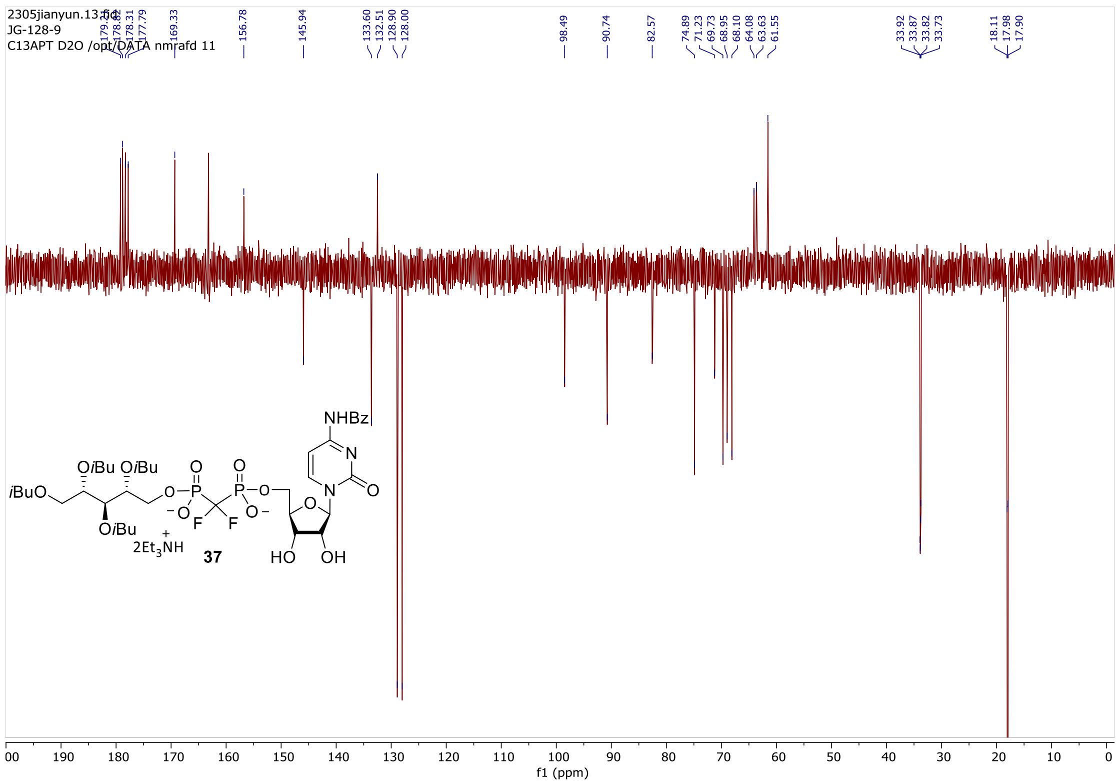

^{13}C NMR (101 MHz, $CDCl_3$) of compound **37**

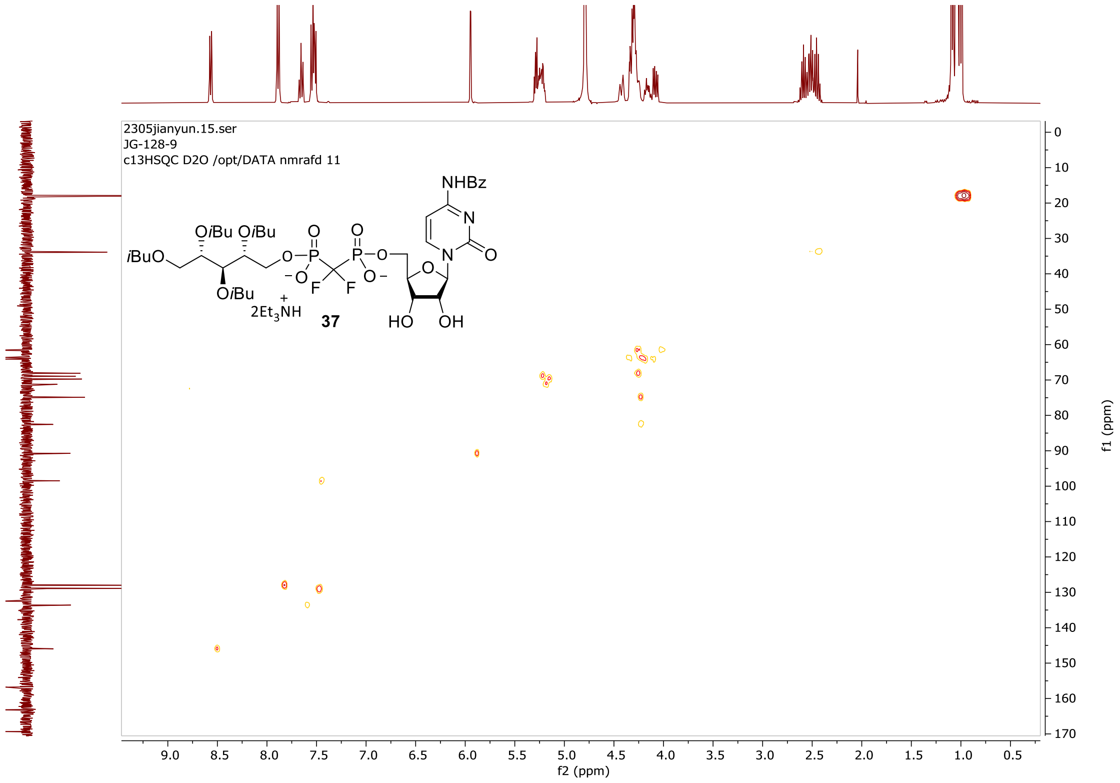

^{1}H - ^{13}C HSQC (400 MHz, $CDCl_3$) of compound **37**

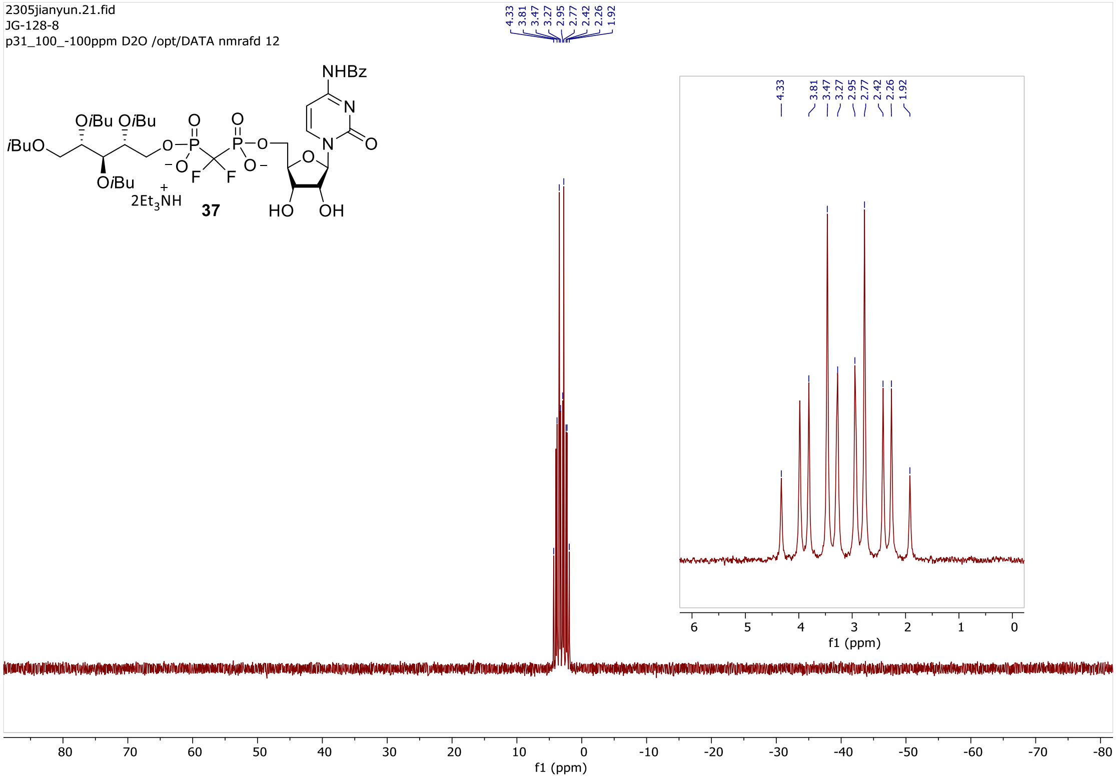

^{31}P NMR (162 MHz, $CDCl_3$) of compound **37**

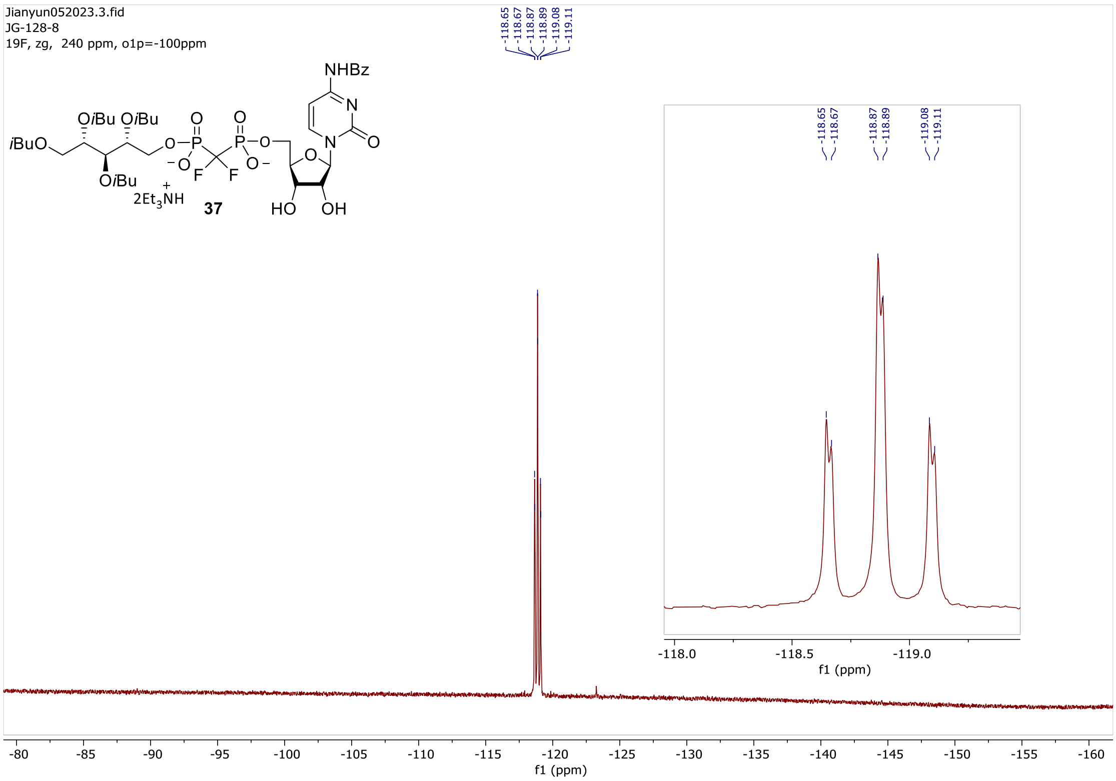

^{19}F NMR (376 MHz, $CDCl_3$) of compound **37**

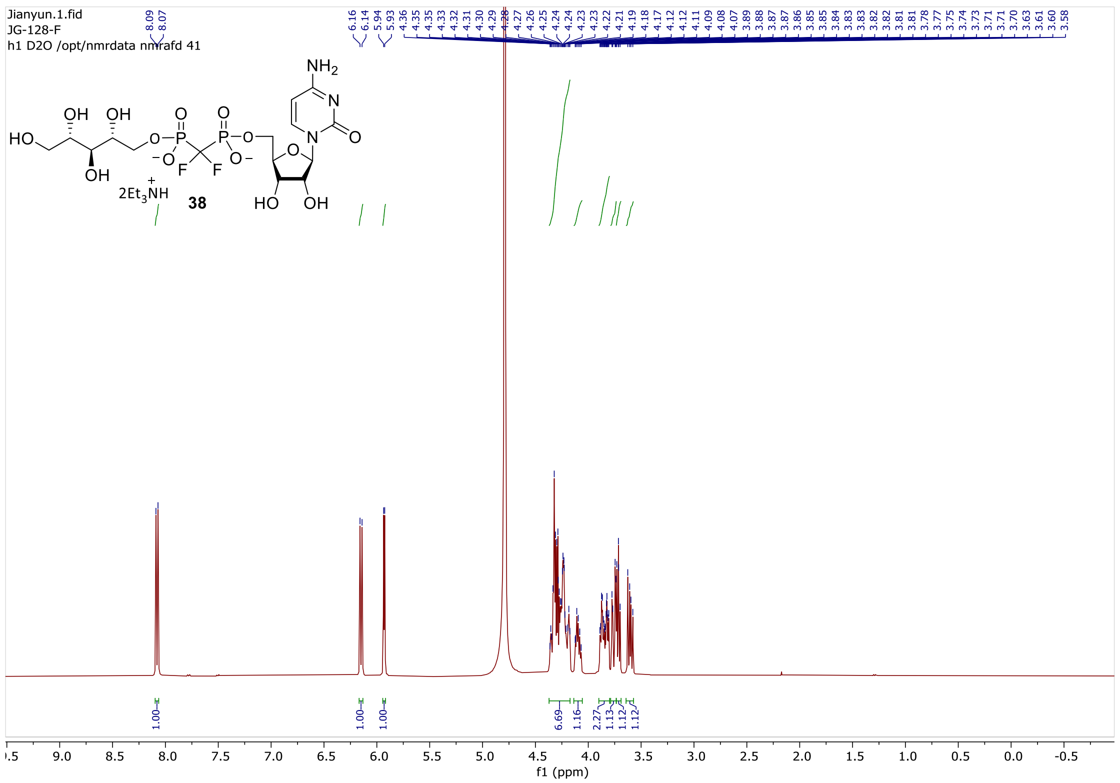

^{1}H NMR (400 MHz, CDCl$_3$) of compound **38**

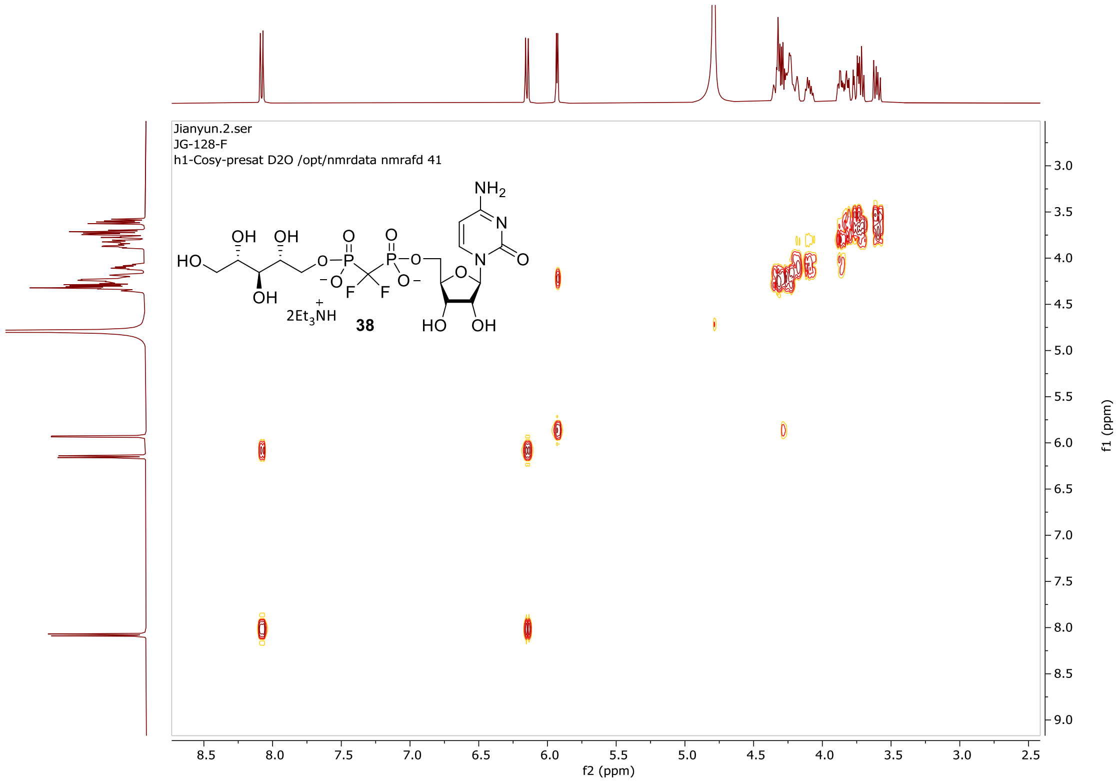

^{1}H - ^{1}H COSY (101 MHz, CDCl$_3$) of compound **38**

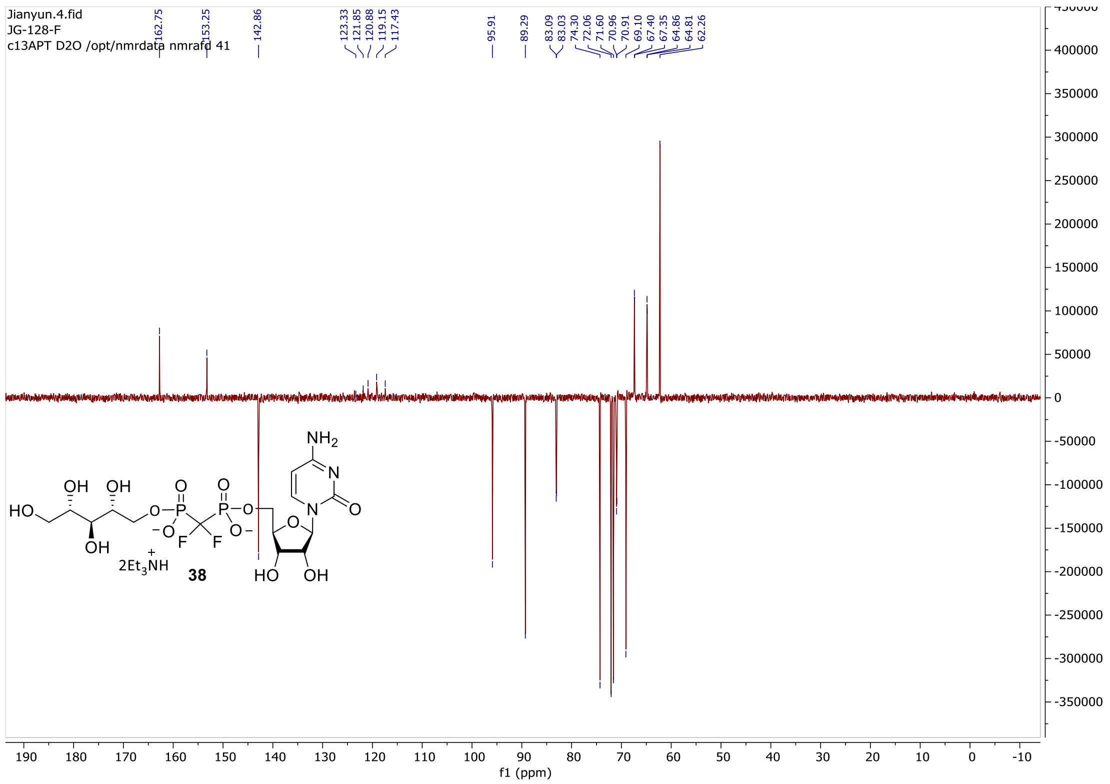

^{13}C NMR (101 MHz, $CDCl_3$) of compound **38**

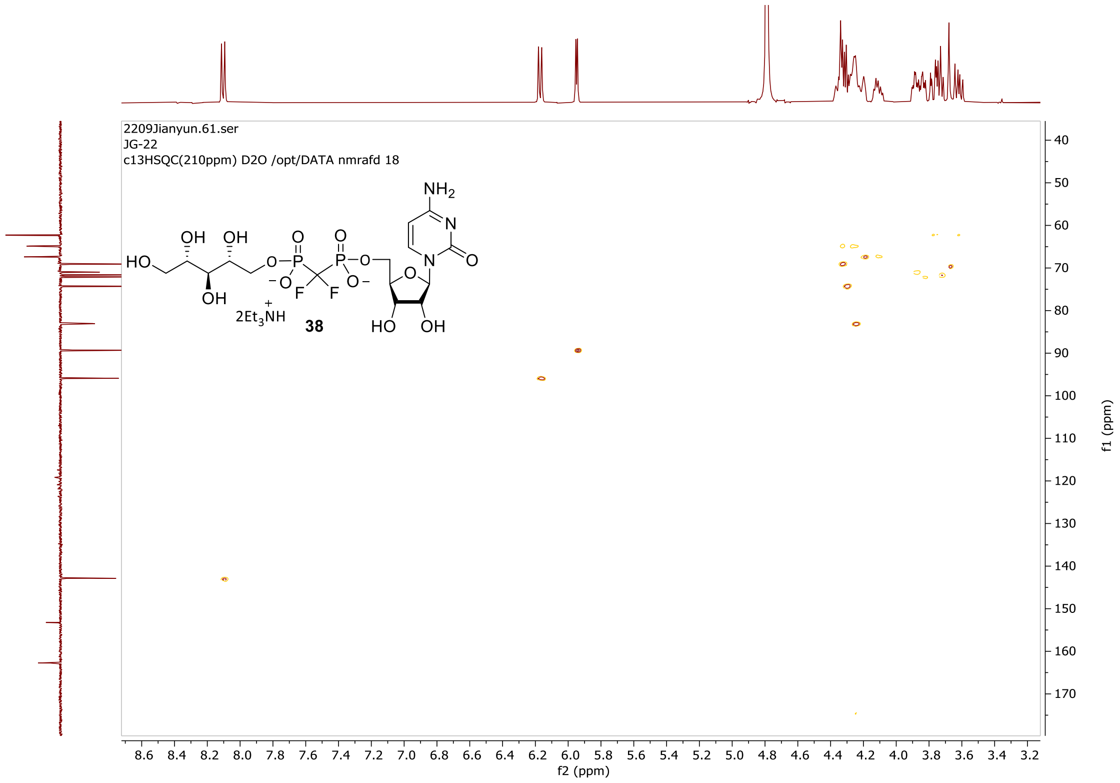

^{1}H - ^{13}C HSQC (400 MHz, $CDCl_3$) of compound **38**

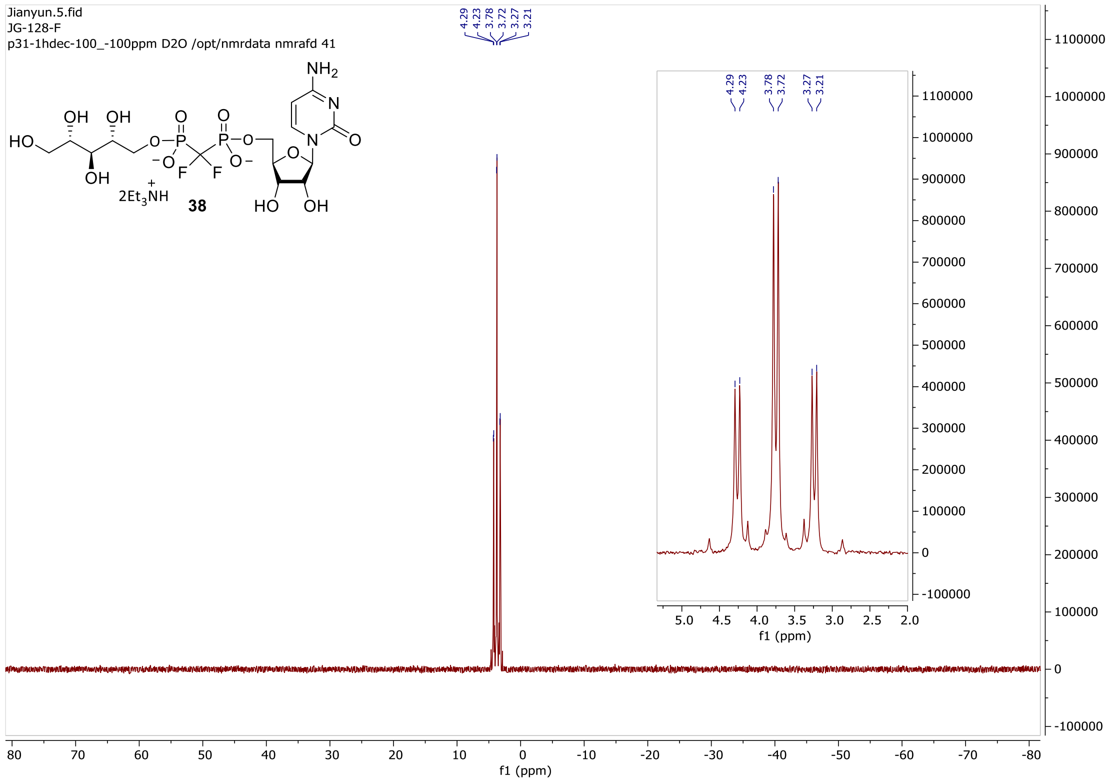

^{31}P NMR (162 MHz, $CDCl_3$) of compound **38**

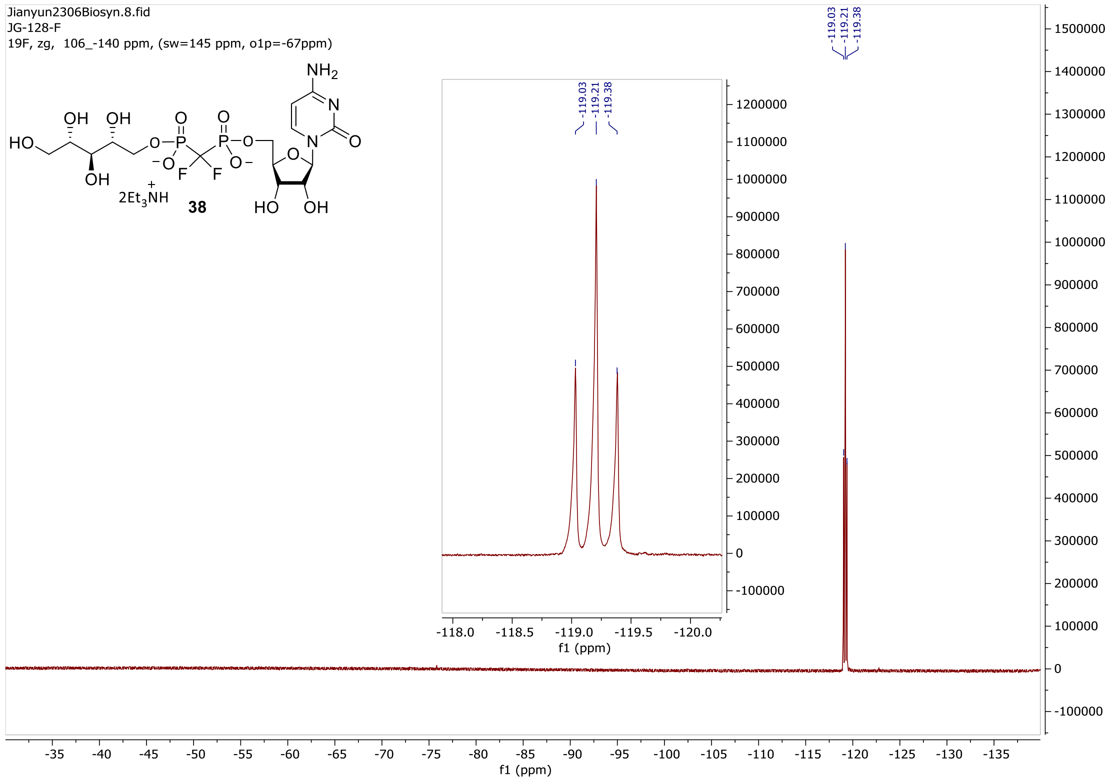

^{19}F NMR (471 MHz, $CDCl_3$) of compound **38**

References

[1] Blackburn, G. M.; Taylor, G. E. Syntheses of some fluorine-containing halomethanephosphonate and methylenebisphosphonate esters. *J. Organomet. Chem.* **1988,** *348*, 55-61.
[2] Davisson, V. J.; Davis, D. R.; Dixit, V. M.; Poulter, C. D. Synthesis of nucleotide 5'-diphosphates from 5'-O-tosyl nucleosides. *J. Org. Chem.* **1987,** *52*, 1794-1801.
[3] Beigelman, L. N.; Mikhailov, S. N. Transient protection in nucleoside synthesis using trityl groups: is it necessary to block hydroxyl groups? *Carbohydr. Res.* **1990,** *203*, 324-329.
[4] Huang, H.-S.; Kong, R.; Zheng, X.-A.; Chen, W.-J.; Han, S.-B.; Zeng, D.-Y.; Gong, S.-S.; Sun, Q. A Practical Method for Regioselective 5′-O-tert-Butyldimethylsilyl Deprotection of Persilylated Nucleosides by Methanolic Phosphomolybdic Acid. *Synlett* **2018,** *29*, 2437-2443.
